# Diagnosing growth in low-grade gliomas with and without longitudinal volume measurements: A retrospective observational study

**DOI:** 10.1371/journal.pmed.1002810

**Published:** 2019-05-28

**Authors:** Hassan M. Fathallah-Shaykh, Andrew DeAtkine, Elizabeth Coffee, Elias Khayat, Asim K. Bag, Xiaosi Han, Paula Province Warren, Markus Bredel, John Fiveash, James Markert, Nidhal Bouaynaya, Louis B. Nabors

**Affiliations:** 1 Department of Neurology, University of Alabama at Birmingham, Birmingham, Alabama, United States of America; 2 Department of Mathematics, University of Alabama at Birmingham, Birmingham, Alabama, United States of America; 3 Department of Diagnostic Imaging, St. Jude Children’s Research Hospital, Memphis, Tennessee, United States of America; 4 Department of Radiation Oncology, University of Alabama at Birmingham, Birmingham, Alabama, United States of America; 5 Department of Neurological Surgery, University of Alabama at Birmingham, Birmingham, Alabama, United States of America; 6 Department of Electrical Engineering, Rowan University, Glassboro, New Jersey, United States of America; Università del Salento, ITALY

## Abstract

**Background:**

Low-grade gliomas cause significant neurological morbidity by brain invasion. There is no universally accepted objective technique available for detection of enlargement of low-grade gliomas in the clinical setting; subjective evaluation by clinicians using visual comparison of longitudinal radiological studies is the gold standard. The aim of this study is to determine whether a computer-assisted diagnosis (CAD) method helps physicians detect earlier growth of low-grade gliomas.

**Methods and findings:**

We reviewed 165 patients diagnosed with grade 2 gliomas, seen at the University of Alabama at Birmingham clinics from 1 July 2017 to 14 May 2018. MRI scans were collected during the spring and summer of 2018. Fifty-six gliomas met the inclusion criteria, including 19 oligodendrogliomas, 26 astrocytomas, and 11 mixed gliomas in 30 males and 26 females with a mean age of 48 years and a range of follow-up of 150.2 months (difference between highest and lowest values). None received radiation therapy. We also studied 7 patients with an imaging abnormality without pathological diagnosis, who were clinically stable at the time of retrospective review (14 May 2018). This study compared growth detection by 7 physicians aided by the CAD method with retrospective clinical reports. The tumors of 63 patients (56 + 7) in 627 MRI scans were digitized, including 34 grade 2 gliomas with radiological progression and 22 radiologically stable grade 2 gliomas. The CAD method consisted of tumor segmentation, computing volumes, and pointing to growth by the online abrupt change-of-point method, which considers only past measurements. Independent scientists have evaluated the segmentation method. In 29 of the 34 patients with progression, the median time to growth detection was only 14 months for CAD compared to 44 months for current standard of care radiological evaluation (*p* < 0.001). Using CAD, accurate detection of tumor enlargement was possible with a median of only 57% change in the tumor volume as compared to a median of 174% change of volume necessary to diagnose tumor growth using standard of care clinical methods (*p* < 0.001). In the radiologically stable group, CAD facilitated growth detection in 13 out of 22 patients. CAD did not detect growth in the imaging abnormality group. The main limitation of this study was its retrospective design; nevertheless, the results depict the current state of a gold standard in clinical practice that allowed a significant increase in tumor volumes from baseline before detection. Such large increases in tumor volume would not be permitted in a prospective design. The number of glioma patients (*n* = 56) is a limitation; however, it is equivalent to the number of patients in phase II clinical trials.

**Conclusions:**

The current practice of visual comparison of longitudinal MRI scans is associated with significant delays in detecting growth of low-grade gliomas. Our findings support the idea that physicians aided by CAD detect growth at significantly smaller volumes than physicians using visual comparison alone. This study does not answer the questions whether to treat or not and which treatment modality is optimal. Nonetheless, early growth detection sets the stage for future clinical studies that address these questions and whether early therapeutic interventions prolong survival and improve quality of life.

## Abstract 2

Hassan Fathallah-Shaykh and colleagues reveal an improved approach for detection of growth in low-grade gliomas.

## Author summary

**Why was this study done?:**

- Low-grade gliomas constitute 15% of all adult brain tumors and cause significant neurological morbidity by brain invasion.
- There is no universally accepted objective technique available for detection of enlargement of low-grade gliomas in the clinical setting.
- The current gold standard is subjective evaluation by clinicians using visual comparison of 2D images from longitudinal radiological studies.
- To improve visual evaluation, a computer-assisted diagnostic procedure that digitizes the tumor and generates volumetric measures could enhance detection of tumor growth by directing the attention of the physician to a change in volume.

**What did the researchers do and find?:**

- We studied the longitudinal radiological studies of 63 patients with a median follow-up period of 33.6 months, and compared detection of growth by 7 physicians aided by the computer-assisted diagnostic procedure to detection of growth by the standard method (visual comparison).
- The computer-assisted method helped physicians detect growth at significantly earlier times and at significantly smaller tumor volumes than the current standard method.
- Physicians aided by the computer-assisted method diagnosed tumor growth in 13 of 22 glioma patients labeled as clinically stable by the standard method.
- Having tumor volume measurements and time series, we were able to identify tumors with nonlinear and nonhomogeneous growth.

**What do these findings mean?:**

- Current clinical practice is associated with significant delays in detecting growth of low-grade gliomas.
- Earlier growth detection and detection at smaller tumor volumes are desirable because smaller tumors are associated with smaller fields of radiation, optimal surgical resections, and longer survival with less neurological morbidity.
- Early growth detection holds the potential of lowering the morbidity, and perhaps mortality, of patients with low-grade gliomas, a possibility that needs to be tested in prospective studies.
- In the future, there could be a policy of classifying and treating patients with low-grade gliomas based on tumor growth rates and direction of tumor growth. The decision to treat would be determined by the rate of growth and proximity to critical areas of the brain, once they have been measured.

## Introduction

Cancer patients are typically monitored with serial imaging of the affected organ; timely detection of tumor recurrence can have profound implications for morbidity and survival. Low-grade gliomas (WHO grade 2) constitute 15% of all adult brain tumors [1–3]. Patients diagnosed with low-grade gliomas are followed by serial magnetic resonance imaging (MRI) of the brain. Fluid-attenuated inversion recovery (FLAIR) is the principle imaging sequence for assessment of growth of low-grade gliomas [4].

At initial diagnosis, low-grade gliomas may be treated by surgery, with or without radiation or chemotherapy [5–7]. More extensive resections of low-grade gliomas are associated with improved overall survival time and progression-free survival time [8–14]. Some studies have reported a correlation between radiation therapy and cognitive impairment in patients with low-grade gliomas [15,16]; however, a recent European Organisation for Research and Treatment of Cancer study found no difference in global cognition in patients treated by radiotherapy versus chemotherapy [17]. At the time of growth detection, low-grade gliomas may remain at the same grade or could have transformed to higher grades [18]; they may again be treated by surgery with or without radiation therapy and chemotherapy [19,20].

Image segmentation and analysis are non-trivial problems because of the unpredictable appearance and shape of brain tumors on MRI. Recently, several artificial intelligence methods and configurations have been applied to brain diseases, including brain tumors [21]. We have developed a method for image segmentation of medical images that extracts object boundaries in computer vision [22]. Our method applies non-negative matrix factorization and a modified level set method; it does not use deep learning, training data, or neural networks. Detection of abrupt changes in the characteristics of physical systems is a fundamental problem in signal processing; applications include fault detection and diagnosis, safety of aircrafts, prediction of earthquakes, and biomedical applications, like electroencephalogram, electromyography, and ECG analysis [23,24].

At present, visual comparison of 2-dimensional (2D) FLAIR images with or without bi-dimensional measurement is the gold standard for surveillance of low-grade gliomas. Physicians compare 2D images from a series of longitudinal studies. Because the overall survival time of low-grade glioma is measured in years, most of the patients have a large longitudinal series of images over several years. Comparison of the current MRI with all prior imaging takes a very long time for image interpretation, which is practically not feasible in the current standard of practice. Furthermore, in a typical cancer center, be it in an academic setting or in a private setting, multiple physicians are involved in assessment of tumor growth, introducing high interobserver variability [25]. We hypothesized that detection of a change in the state of the tumor, i.e., tumor growth, could be improved by a computer-assisted diagnosis (CAD) procedure that digitizes the tumor and directs the attention of the physician to a change in volume. This is important because small tumor size is associated with less neurological morbidity [2,17].

## Methods

### Ethical approval

The Institutional Review Board of the University of Alabama at Birmingham approved the research; waiver of informed consent was granted because the research involved no greater than minimal risk and no procedures for which written consent is normally required outside the research context. This study did not have a protocol.

### Study design

This is a retrospective observational study of the accuracy of the diagnosis of glioma growth by expert physicians who viewed MRI scans in a clinical setting and by 7 expert physicians who, in addition, were provided segmented images, numerical volumes, and a statistical determination of growth by the change-of-point method.

### Patient selection and study size

**Fig 1:**
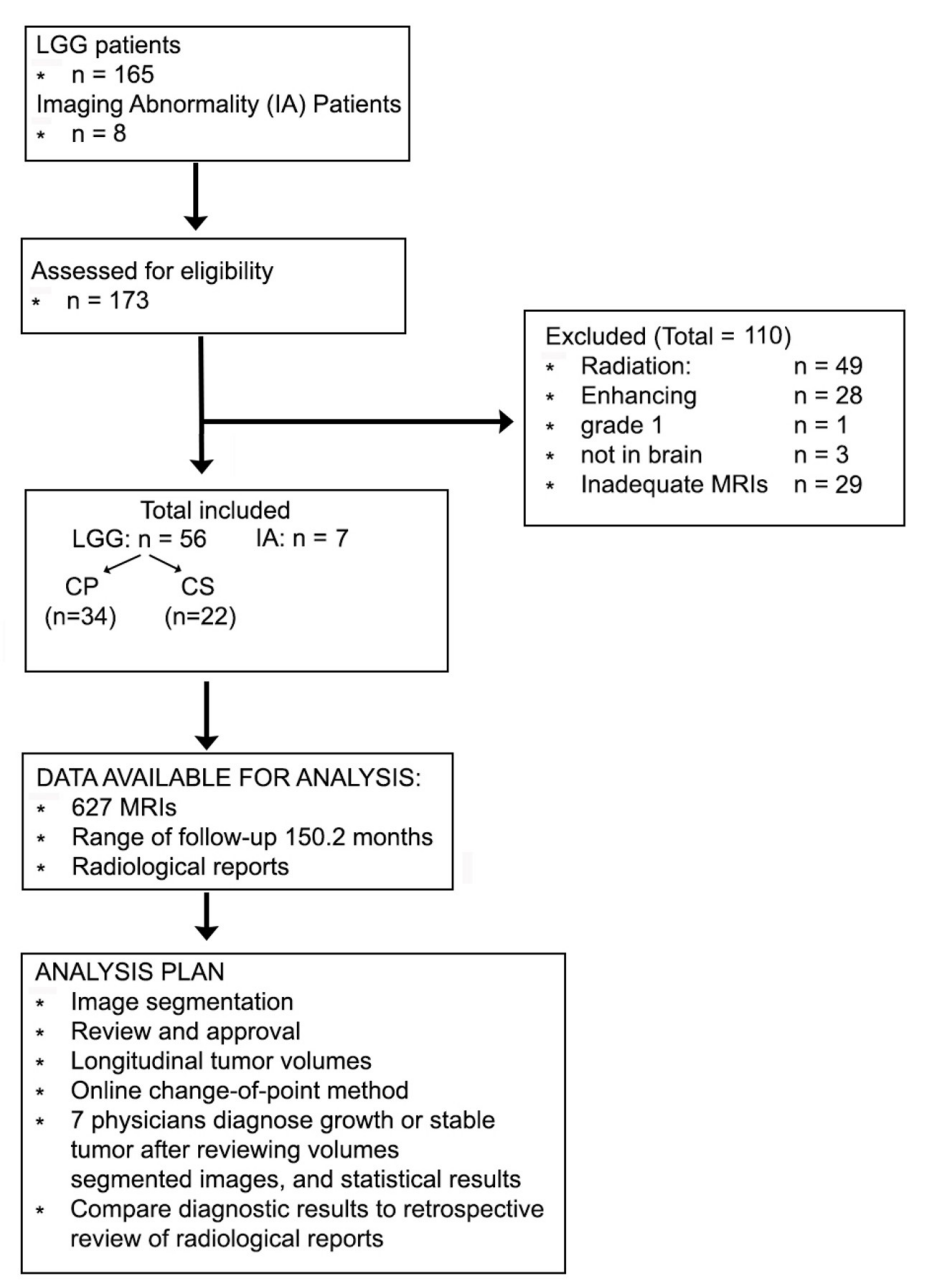
Flow diagram and analysis plan. CP, clinical progression; CS, clinically stable; LLG, low-grade glioma.

We reviewed 165 patients who had been diagnosed with WHO grade 2 gliomas, seen in the neuro-oncology clinics at the University of Alabama at Birmingham from 1 July 2017 to 14 May 2018 (see flow diagram in Fig 1). The MRI scans were collected from the radiology PACS during the spring and summer of 2018.

The inclusion criteria were (1) pathological diagnosis of grade 2 oligodendroglioma (oligo), grade 2 astrocytoma (astro), or grade 2 mixed glioma in the brain excluding the pineal gland and (2) at least 4 MRI scans available for review either after the initial diagnosis or after the completion of chemotherapy with temozolomide (if applicable). The exclusion criteria were (1) treatment with radiation therapy after the initial diagnosis or (2) radiological reports indicating development of new enhancement without an increase in FLAIR signal. Patients treated by radiation therapy were excluded because radiation may confound the results by causing an independent increase in FLAIR signal. We excluded patients whose radiological reports described new enhancing nodules without an increase in FLAIR signal because they are easily detected by visual examination.

A total of 56 gliomas met the inclusion criteria, including 19 oligos, 26 astros, and 11 mixed gliomas; only 2 patients received temozolomide (Table 1). All of the oligos had the 1p/19q co-deletions except for 1 with a single deletion of 19q. At the time of retrospective review (14 May 2018), 34/56 patients had been diagnosed with clinical progression while the remaining 22/56 were diagnosed as being clinically stable by visual comparison of the most recent MRI performed at the last clinic visit. We reviewed the records of 8 patients followed in our clinics for an imaging abnormality without pathological diagnosis; 1 patient was excluded because of lack of follow-up information. All 7 imaging abnormality patients were considered clinically stable at the time of review of this study.

**Table 1:** Patient characteristics.

Pathology	Number of patients	Mean age (years)	Number of males	Number of females	Number treated with temozolomide
Oligodendroglioma	19	47	11	8	1
Astrocytoma	26	46	14	12	1
Mixed glioma	11	53	5	6	0
All	56	48	30	26	2

### Time to growth detected by standard clinical care

Different board-certified neuro-radiologists at the University of Alabama at Birmingham Hospital generated the radiological reports after evaluating each longitudinal MRI scan. We retrospectively calculated the time to growth detection from the impressions of the radiological reports of these patients.

### Tumor segmentation

A total of 627 MRI scans were analyzed. Segmentation of the FLAIR sequence was performed by 2 procedures: First, an automated algorithm classified and contoured the different regions in the image; it applied non-negative matrix factorization and a modified level set method (NMF-LSM) as detailed in Dera et al. [22]. This automated segmentation generated 8 segments for every image (see [22]), which were ranked by their maximal intensities. Second, the final tumor margins were obtained by combining the regions whose maximal intensities were above the level of the gray matter. A physician reviewed and approved the final tumor margins. Detailed information on the segmentation method and combining the segments to compute tumor margins is presented elsewhere [26]. The organizers of the Multimodal Brain Tumor Segmentation (BraTS) Challenge (https://www.med.upenn.edu/sbia/brats2018/data.html) have independently evaluated the accuracy of this method in the segmentation of the hyperintense areas in T2/FLAIR MRI (i.e., whole tumor label) [27]. Tumor volumes were computed by multiplying the sum of the tumor segments in all axial images by the distance between images. The computations were performed at the Cheaha supercomputer of the University of Alabama at Birmingham (https://docs.uabgrid.uab.edu/wiki/cheaha).

### Online abrupt changes of point

To exclude FLAIR changes due to the evolution of post-surgical changes, the baseline volume in the longitudinal series was the first minimum after surgical resection. To identify an abrupt change of volume, we applied the function findchangepts in Matlab (Mathworks), detecting a change in the root-mean-square level at a minimum threshold of 500/(volume at baseline) and a minimum of 2 samples between change points. The number 500 corresponds to 5% of the rounded median of the baseline volume.

In the clinical setting, a physician reviews the current MRI scan and compares it to MRI scans performed on earlier dates. To simulate a clinic visit, the online change of point considers only past measurements. The time to growth detected by the CAD method corresponds to the period of time between the dates of the baseline MRI and the first change of point.

### Review of growth detection by the change-of-point method

Growth detected by the statistical change-of-point method was reviewed by 7 physicians who are board-certified in neuro-radiology (AKB), neuro-imaging (LBN), neuro-oncology (HMF, LBN, PPW, XH), radiation oncology (MB), and neurosurgery (JM). For 63 cases, the physicians were provided with (1) the tumor volumes, (2) determination of growth or stability by the change-of-point method, and (3) the images with segmentation, obtained at (1) baseline (as defined in the previous section), (2) the time point of growth detection by the statistical change-of-point method, if different than the last visit, and (3) the last visit. The images included a red line contouring the tumor margins, delineated by the segmentation method (see S1 Data). The physicians were asked to determine if the tumor had grown compared to baseline or not. These MRI scans and volumes, including the segmentation data, are available in S1 Table and S1 and S2 Datas.

### Mathematical model of gliomas

The authors have recently reported a system of partial differential equations (PDEs) that model glioma growth at the scale of MRI and pathology. The equations include the rates of replication (mitosis), brain invasion, angiogenesis, and a threshold for hypoxia; the numerical methods used to solve the system of PDEs are detailed elsewhere [18,28,29].

### Statistical analysis and curve fitting

The *p*-values were generated by the Mann–Whitney–Wilcoxon 2-tail test. Curve fitting was done in Matlab using the fit function and the poly1 (*y* = *p*1 * *x* + *p*2) and exp1 (*y* = *a* * exp(*b* * *x*)) models. Normalized volumes were computed by subtracting the baseline volume and dividing by the most recent volume. Time intervals from baseline were normalized by dividing by the largest. To identify tumors with exponential model growth, we selected a normalized curve if its nonlinear sum of squares due to error (sse) was less than 0.6 * linear sse (0.6 was chosen as it yields an exponential model fit of *r*^2^ > 0.85 for the normalized data of all the selected curves).

## Results

### Patient description

The CAD method was applied to the longitudinal MRI scans of a total of 63 patients, including 56 patients with gliomas; the mean age and the proportions male and female are shown in Table 1. The range, mean, and median of the follow-up were 150.2, 46.6, and 33.6 months, respectively. There were 3 groups of patients: 34 patients with grade 2 gliomas with a known clinical progression, 22 patients with grade 2 gliomas who were clinically stable by visual comparison, and 7 patients with an imaging abnormality, who were also clinically stable by visual comparison. The clinical progression group included 7 oligos, 18 astros, and 9 mixed gliomas. The clinically stable tumor group included 12 oligos, 8 astros, and 2 mixed gliomas.

### CAD detects growth earlier

**Fig 2:**
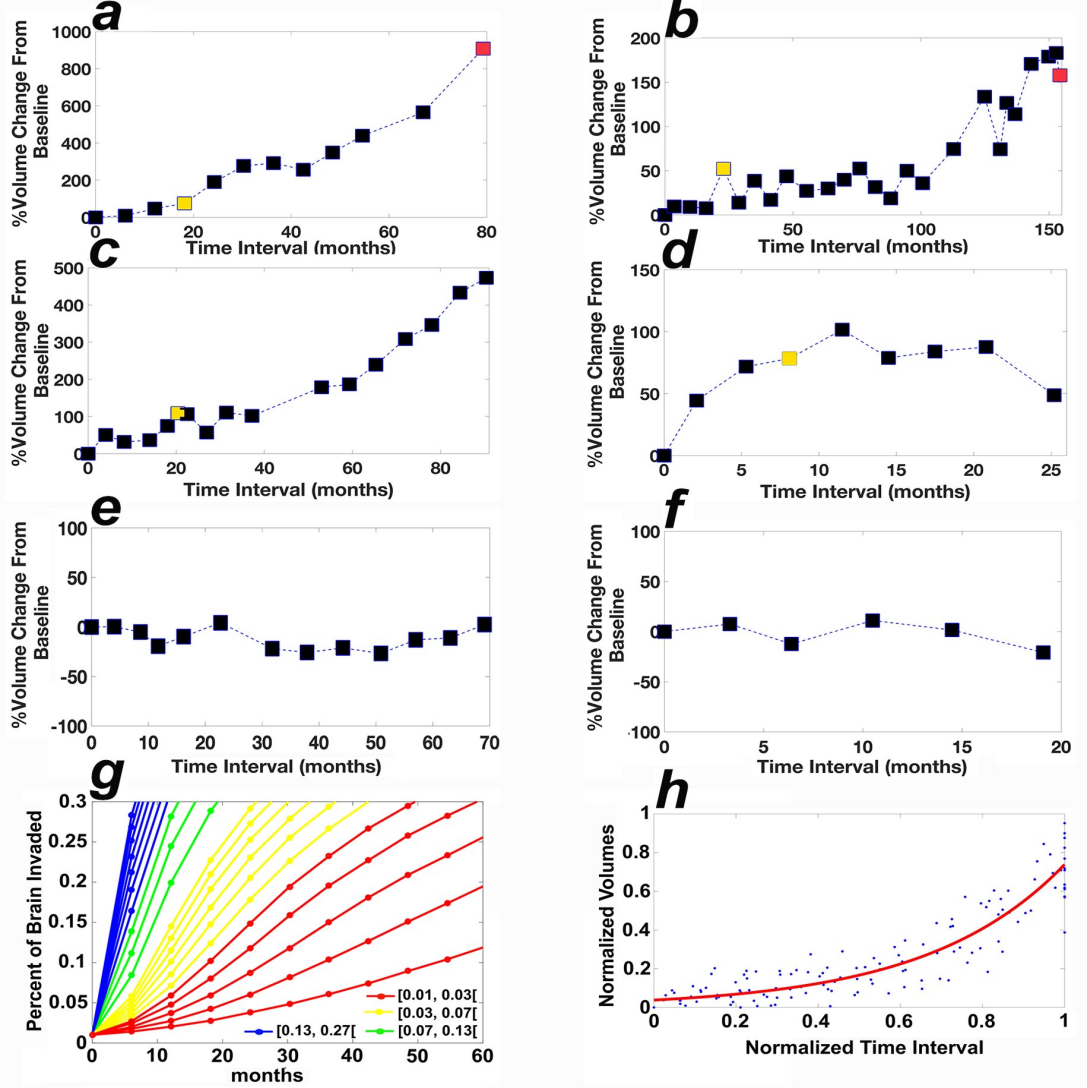
Growth curves of grade 2 gliomas. (a and b) The tumor volumes of 2 patients in the clinical progression group, diagnosed with oligos. (c and d) The tumor volumes of 2 patients in the clinically stable group diagnosed with an oligo and an astro, respectively. The volumes at the time to growth detected by CAD and visual comparison are colored yellow and red, respectively. (e and f) The tumor volumes of 2 patients in the clinically stable group by CAD and visual comparison, diagnosed with an astro and an oligo, respectively. The *x*-axis in (a–f) corresponds to the time interval from the baseline MRI. (g) The results of simulations of the mathematical model for grade 2 gliomas, showing percent of brain invaded by the tumor (*y*-axis) as a function of the parameter for mitotic rate (per hour) in the presence of a low angiogenesis rate (0.1/hour), see Scribner et al [18]. (h) The curve fit of the normalized data of 14 patients with nonlinear growth using the model *f(x)* = *a* * exp(*b* * *x*), coefficients (with 95% confidence bounds): *a* = 0.03751 (0.02759, 0.04743), *b* = 2.98 (2.69, 3.27), sum of squares due to error = 1.3701, *r*^2^ = 0.8580. astro, astrocytoma; CAD, computer-assisted diagnosis; oligo, oligodendroglioma.

In the clinical progression group, the median time to growth detected by visual comparison for the oligos, astros, and mixed gliomas was 79 months, 33 months, and 56 months, respectively. CAD aided the physicians in detecting growth statistically significantly earlier in 7/7 oligos (median = 19 months), 14/18 astros (median = 12 months), and 8/9 mixed gliomas (median: 16 months; Table 2). Furthermore, tumors were significantly larger at the time point of detection when growth was detected by standard of care radiological assessment compared to CAD, with median values of 163% versus 52%, 155% versus 50%, and 286% versus 69% for oligos, astros, and mixed gliomas, respectively (Table 2 and Fig 2).

**Table 2:** Clinical progression glioma group.

Pathology	Number	ΔG	Time to growth (months), median (IQR)			ΔV, median (IQR)		
			CAD	VC	*p-*Value	CAD	VC	*p-*Value
Oligodendroglioma	7	7	19 (13–23)	79 (48–101)	<0.001	52% (36%–72%)	163% (141%–479%)	0.001
Astrocytoma	18	14	12 (10–16)	33 (26–44)	<0.001	50% (37%–76%)	155% (120%–257%)	0.001
Mixed glioma	9	8	16 (10–20)	56 (31–70)	<0.001	69% (46%–116%)	286% (203%–537%)	<0.001
All	34	29	14 (11–20)	44 (30–68)	<0.001	57% (36%–77%)	174% (134%–342%)	<0.001

ΔG: number of patients whose glioma time to growth detected by computer-assisted diagnosis (CAD) was shorter than that detected by visual comparison (VC). ΔV: percent change in tumor volume from baseline to time point of growth detection. The *p*-values were generated by the Mann–Whitney–Wilcoxon 2-tail test. The results of CAD were identical to that of VC in 5 patients.

### Time to growth in clinically stable grade 2 gliomas

**Fig 3:**
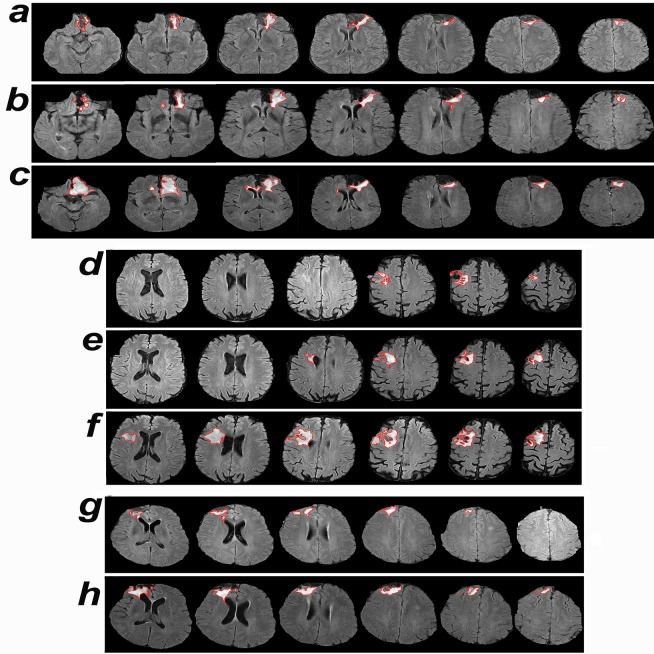
Time to growth detected by computer-assisted-diagnosis and visual comparison. (a–c) Axial FLAIR MRI scans of a patient in the clinical progression group, diagnosed with an oligo, whose tumor volumes are shown in Fig 2B, at baseline (a), the time point of growth detection by computer-assisted diagnosis (b), and the time point of detection by visual comparison (c). (d–f) Axial FLAIR MRI scans of a clinically stable patient diagnosed with a grade 2 oligo, whose tumor volumes are shown in Fig 2C, at baseline (d), the time point of growth detection by computer-assisted diagnosis (e), and the last follow-up MRI, considered to be stable by visual comparison (f). Our panel of physicians reviewed the images and agreed that the tumor had grown. This patient elected to have a resection; the pathological diagnosis revealed grade 3 oligo. (g and h) Axial FLAIR MRI scans of a clinically stable patient diagnosed with an astro, whose tumor volumes are shown in Fig 2D, at baseline (g) and the time point of growth detection by computer-assisted diagnosis (h). astro, astrocytoma; FLAIR, fluid-attenuated inversion recovery; oligo, oligodendroglioma.

In the clinically stable grade 2 glioma group, CAD aided the physicians in detecting growth in 7/12 oligos, 4/8 astros, and 2/2 mixed gliomas; the median period of follow-up was, respectively, 37 months and 19 months for the grade 2 gliomas that exhibited growth versus remained stable by the CAD method (Table 3). The median time to growth detected by the CAD method for oligos, astros, and mixed gliomas was 15 months, 12 months, and 8 months, respectively (Table 3 and Figs 2 and 3).

**Table 3:** Clinically stable glioma group.

Pathology	Number	CAD G	CAD TTG	FU G	FU S	CAD ΔV
Oligodendroglioma	12	7	15 (11–24)	81 (26–86)	19 (16–26)	51% (45%–97%)
Astrocytoma	8	4	12 (8–16)	22 (18–24)	18 (6–50)	78% (64%–126%)
Mixed glioma	2	2	8 (7–9)	87 (73–101)	N.A.	42% (26%–58%)
All	22	13	13 (9–17)	37 (22–88)	19 (8–34)	58% (46%–86%)

CAD G: number of patients in the clinically stable group whose tumors were detected to have grown by computer-assisted diagnosis (CAD). CAD TTG: median (IQR) time to growth (TTG) detected by CAD (months). FU G: median (IQR) time interval between baseline and last MRI scan for patients diagnosed with tumor growth by CAD (months). FU S: median (IQR) time interval between baseline and last MRI scan for patients whose tumors were stable by CAD (months). CAD ΔV: median (IQR) percent change in tumor volume from baseline to growth detection. N.A., not applicable. A statistical analysis comparing CAD TTG and visual comparison (VC) TTG is not applicable here because the latter is not known; nonetheless, VC TTG will be larger than FU G. The Mann–Whitney–Wilcoxon 2-tail test comparing the CAD TTG and FU G of all 13 patients yields *p* < 0.001. The results of CAD were identical to VC in 9 patients.

Three of these 13 gliomas exhibited additional tumor growth during the follow-up period after the time point of growth detection by CAD. In light of the CAD results, the patient whose longitudinal MRI scans are shown in Fig 3D–3F elected to have a resection; pathological examination revealed a WHO grade 3 oligo, a diagnosis that mandates therapeutic intervention. The treating neurosurgeon noted that whereas the current surgical option was a subtotal resection (Fig 3F), a gross total resection would have been a possibility at the time point of growth detection by CAD (Fig 3E).

### Imaging abnormality group

CAD did not detect growth in any of the 7 patients followed for an imaging abnormality. These patients were followed for an average of 79 months (IQR = 68.5 months) after the first MRI.

### Review of predictions of change-of-point method

The 7 expert physicians reviewed and agreed with the determinations of growth (true positives, *n* = 34 + 13) and no growth (true negatives, *n* = 7 + 9) at the times predicted by the statistical online change-of-point method (Fig 3). The MRI data with segmentation results (S1 Data) and volumetric measurements (S2 Data) with corresponding time intervals are available to the reader (see S1 Table).

### Nonlinear stationary growth

The data demonstrate that time to growth detected with the aid of the CAD method can be several years shorter than that detected by visual comparison (Table 2), hence the importance of the rate of tumor growth. Simulations of the mathematical model of gliomas reveal that growth can be either nonlinear or almost linear as a function of the mitotic rate (Fig 2G); small mitotic rates generate nonlinear curves. Using the normalized data of the 47 tumors with growth (*n* = 47; 34 of Table 2 and 13 of Table 3), we identify 14/47 tumors whose normalized growth curves fit a nonlinear exponential model (Fig 2H, *r*^2^ = 0.86). We note that though 22/29 tumors in the clinical progression group continued to grow after the time point of growth detection by the CAD method (Fig 2A), 7 low-grade gliomas remained in a stationary phase of slow growth (S2 Table), which lasted for longer than 3 years in 3 gliomas (Figs 2B and 3A–3C), 18 months in 2 gliomas, 14 months in 1 glioma, and 9 months in 1 glioma.

### 3D growth is nonhomogeneous

**Fig 4:**
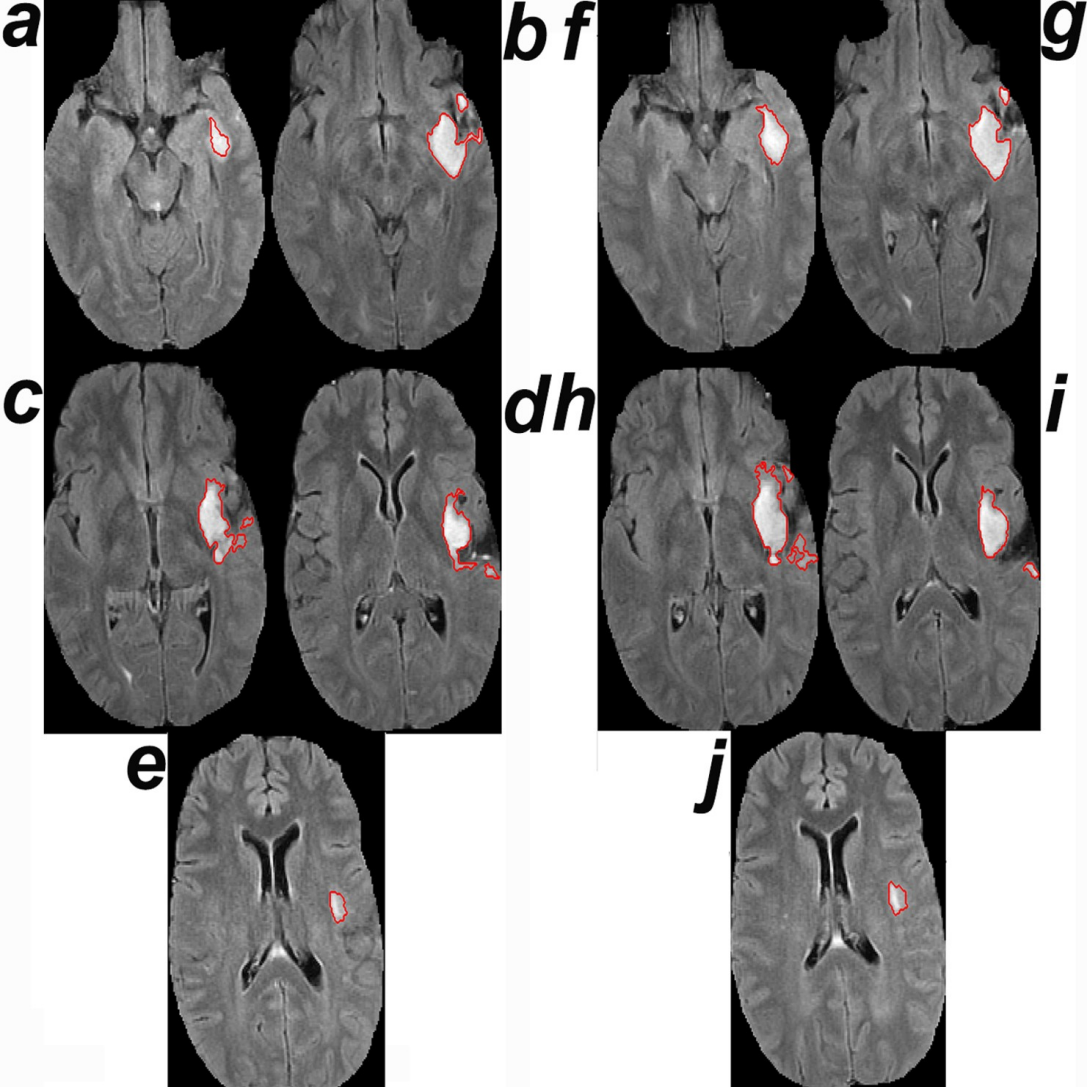
Growth away from the largest tumor section. (a–e) MRI of tumor 4385 (see S1 and S2 Datas) at time 0. (f–j) MRI at the time point of growth detection by CAD. The surface areas of the tumor segments in (a–j) are 268, 1,174, 1,240, 962, 246, 718, 1,262, 1,764, 994, and 282 pixels^2^, respectively. The tumor exhibits larger percent growth from baseline in the third dimension (compare a and f: 268 and 718) away from the section containing the largest tumor component at baseline (compare c and h: 1,240 and 1,764). The second MRI was deemed stable by visual comparison.

Currently, clinical trials compute the size of a glioma as the bi-dimensional product of the 2 largest perpendicular diameters in the 2D section that includes the largest tumor component. This practice assumes that a glioma grows homogeneously in 3D, i.e., it grows at equal rates in all directions. Fig 4 shows a counterexample, where tumor growth is not homogeneous in 3D because the tumor grows faster at sections away from the largest axial tumor component.

## Discussion

Visual comparison of longitudinal radiological studies is widely used in oncology. In all cases, physicians examine 2D computed tomography or magnetic resonance images to diagnose 4-dimensional objects, i.e., a change in volume over time. Here, we screened 165 gliomas and analyzed the data of a total of 63 patients, including 627 MRI scans; unexpectedly, we found large differences in growth detection by visual comparison and by physicians aided by the CAD method. Because low-grade gliomas are followed for several years, physicians are tasked with comparing the current MRI to all previous studies. Reasons for missing growth by visual inspection include (1) the large number of prior studies, which take a very long time for image interpretation, (2) the current practice of comparing the current MRI to a couple of MRI scans immediately preceding it, (3) the lack of determination of the baseline MRI, (4) small changes from one study to the next that add up over time, (5) that comparing single 2-dimensional images misses growth in the third dimension (Fig 4), i.e., in sections away for the largest tumor component (e.g., cases 4384, 4385, 6936, 7492, 7505, and 7736 in S1 Data), and (6) that baseline volume appears to be a factor for detecting growth by visual comparison; for example, the tumor in Fig 2C (case 7504), whose baseline volume is 42% of that of the tumor in Fig 2B (case 7490), was deemed stable after growing 6-fold whereas the growth of the tumor in Fig 2B was detected after it grew by only 2-fold.

The retrospective analysis of radiological reports in this study yields an unaltered view of the landscape of the diagnostic imaging of gliomas at a tertiary brain tumor center. In analyzing longitudinal measurements of tumor volumes, the problem concerns both detecting whether or not a change in tumor volume has occurred and identifying the time of any such change. These questions are addressed by combining tumor segmentation with the change-of-point analysis. Several segmentation methods including computer vision have recently been developed [3]. CAD improves the detection of growth in grade 2 gliomas by contouring the tumor margins and generating a signal that directs the attention of the physician towards a change of point (Fig 3). The method used in this paper is semi-automated, i.e., the final tumor contouring requires human approval. This method has been ranked among the top 3 algorithms, statistically equivalent with 2 other algorithms competing in the BraTS 2016 challenge [27]. Our segmentation method differs from deep learning algorithms as it does not require offline training of a library of reference images. The online change-of-point method is a well-suited statistical method to simulate the clinic visit as it considers only past measurements at each time point. It is better suited than the fixed threshold method because it handles all types of time-ordered data, including data from non-normal distributions and data with outliers [23,24]. To exclude changes caused by surgical intervention, the baseline MRI is taken as the one that corresponds to the first minimal volume after resection.

Analysis of the 117 measurements of the 16 true-negative growth curves, i.e., stable by CAD and confirmed by expert physician review, yields a mean and standard deviation percent volume change of 0.99% and 26.54%, respectively (curves for 2 such cases shown in Fig 2E and 2F). FLAIR images have variable quality; however, lower quality images are more likely to yield underestimation than overestimation of tumor growth because CAD uses a physician-in-the-loop approach whereby a physician must review and confirm both the segmentation results and the determination of growth. Because the reviewing physician eliminates false positive segmentations and outliers, we believe that in cases where consecutive volumes show a large variation, the higher measurement is more accurate than the lower volume. The main objective of the CAD method employed here is to point the attention towards a potential growth event; the physician has the final responsibility to confirm or not. We argue that minimizing false negatives (even at the cost of potential false positives) is prudent and in the best interest of patients. For instance, if we consider a case similar to the one shown in Fig 2A, it is possible that a physician may not confirm the first growth signal. However, the numerous and continuous alerts starting at month 19 would hopefully make it impossible for the tumor to be allowed to grow unchecked until month 80. We did not encounter false positive signals in our datasets as the reviewing physicians in all cases agreed with the change-of-point detection (S1 Data). Nonetheless, if physician-endorsed false positives are frequent, one could increase the stringency of the change-of-point method by varying the threshold or by considering the second or third change of point.

Tumor assessments in 2D and 3D differ with respect to magnitude. This study evaluated and compared longitudinal volumes of low-grade gliomas. In clinical trials, tumor progression is currently assessed by studying 2D sections of the brain that include the largest component of the tumor; progression is determined when the product of 2 perpendicular lines increases by 25%. For example, a 12% increase in each of 2 dimensions generates a 25% increase in the product in 2D (1.12^2^ = 1.25). Multiplying by a third dimensional increase of 12% leads to a 41% increase in the volume (1.12^3^ = 1.41). Similarly, 20% and 25% increases in each dimension produce 73% and 95% increases in volume, respectively. Conversely, a 300% increase in volume can be generated by a 44% increase in each dimension (1.44^3^ = 3).

The numerical growth charts suggest that low-grade gliomas may be distinguished not only by their pathological diagnosis but also by their rate of growth (Fig 2). For example, the tumor shown in Fig 2A grew at a faster rate than the tumors shown in Fig 2B–2D. The volumetric analysis permits the computation of the rate of growth of low-grade gliomas over time, which may turn out to be a biological marker that may enhance tumor classification and guide therapy.

Although the current study demonstrates the utility of CAD in helping physicians detect growth of grade 2 gliomas following initial observation, additional work is likely needed to develop models for progression after some therapies. Though none of the patients in this study had received radiation therapy, radiation therapy and immunotherapy may be associated with new FLAIR signal that does not represent tumor growth.

The main limitation of this study was its retrospective design since the time point of tumor growth detection was determined by retrospective review of the radiological reports. The findings unequivocally point towards shortcomings of the current state of clinical practice that allowed a significant increase in tumor volumes from baseline before growth was detected. Because of ethical considerations, these large magnitudes of tumor growth would not be permitted in a prospective design as coordinators would have to address the signals of growth generated by CAD. Another limitation was the fact that the physicians who validated the volumetric results did not review all the longitudinal MRI scans in the absence of segmentation: They viewed the volumes and the segmented images of the baseline and last MRI scans and the MRI deemed to have tumor growth by CAD (if any); they were offered the option of viewing additional segmented images if they desired. The reason for this design was that the time needed to review 627 MRI scans is both significant and prohibitive; for the same reason, practitioners currently compare the current MRI to a couple of MRI scans immediately preceding it. The number of glioma patients (*n* = 56) is a limitation; however, it is equivalent to the number of patients in phase II clinical trials. Another limitation is the fact that all patients were adults; we plan to study pediatric patients in the future.

It is evident that the volumetric data of the CAD method help physicians detect growth of low-grade gliomas significantly earlier than the current gold standard practice of visual comparison (Tables 2 and 3). However, this study does not address the questions of whether to treat or not and which treatment modality is optimal. Nonetheless, early detection sets the stage for future clinical studies to address these questions and whether early therapeutic interventions prolong survival and improve quality of life. In general, earlier growth detection and detection at smaller tumor volumes are desirable because there is evidence that smaller tumors are associated with smaller fields of radiation, optimal surgical resections (see Fig 3D–3F), and longer survival with less neurological morbidity [8–14,19,20]. We suggest studying early interventions for cases where (1) the new growth is in the proximity of key nonsurgical structures like the corpus callosum, (2) the rate of growth is elevated, or (3) the tumor is sensitive to chemotherapy.

Because low-grade gliomas grow at variable but slow rates, clinicians need to compare a large number of longitudinal images spanning several months or years to detect growth, leading to significant delays in detection of tumor enlargement. Readily available computer-generated tumor outlines combined with longitudinal volumetric data and the identification of a statistically significant change of point aid a rapid diagnosis of tumor enlargement. Hence, CAD could avoid unpredictable delays and improve the determination of efficacy of new therapeutic interventions. Furthermore, early growth detection holds the potential of lowering the morbidity, and perhaps mortality, of patients with low-grade gliomas, a possibility that needs to be tested in prospective studies.

## Supporting information

S1 Data MRI data. The .zip file includes .pdf files labeled as follows, using case number 4224 as an example: 4224_t0.pdf includes the MRI data with segmentation of case number 4224 at baseline. 4224_t1_CAD_G_VC_S.pdf includes the MRI data with segmentation of case number 4224 at time 1, i.e., the time point of growth detection by the change-of-point method when the time point was different from the most recent MRI. 4224_tend_CAD_G_VC_G.pdf includes the MRI data with segmentation of case number 4224 at the most recent MRI. CAD_G and CAD_S mean that the change-of-point method predicted growth (G) or stability (S), respectively. VC_G and VC_S mean that the visual comparison detected growth (G) or stability (S), respectively.
(ZIP)

S2 Data Volumetric measurements. The Excel sheet lists the tumor volumes (pixels^3) and corresponding time intervals from baseline (months) for each case number.
(XLSX)

S1 STROBE checklist (DOC)

S1 Table Summary of the MRI data. The case numbers correspond to the same numbers on the labels of the .pdf files that include the MRI data (S1 Data). Interval to last MRI refers to the interval of time from the baseline MRI to the most recent MRI. CAD Dx and VC Dx refer to the determination of growth or not (i.e., stable) by the CAD method and visual comparison (VC), respectively. Time 1 denotes the time point at which CAD detected growth earlier than the last MRI, if any. Groups 1, 2, and 3 refer to patients with known radiological tumor progression, stable glioma, and imaging abnormality, respectively. As compared to VC, CAD detected earlier growth in 29 group 1 gliomas (blue) and 13 group 2 gliomas (red).
(XLSX)

S2 Table Stationary growth in the clinical progression group. Number of patients in the clinical progression group with stationary/slow growth after the time point of growth detection by CAD lasting for 9 months, 14 months, 18 months, and longer than 3 years.
(XLSX)

## Abbreviations

astro: astrocytoma
BraTs: Multimodal Brain Tumor Segmentation
CAD: computer-assisted diagnosis
FLAIR: fluid-attenuated inversion recovery
oligo: oligodendroglioma
