# Supplementary figures and images for: Diagnosing growth in low-grade gliomas with and without longitudinal volume measurements: A retrospective observational study (part 2 of 2)
Source: PLoS Med. 2019 May 28;16(5):e1002810. doi: 10.1371/journal.pmed.1002810 (PMC6538148; doi:10.1371/journal.pmed.1002810)

Case Number:7751; LAST MRI; CAD --> STABLE; VC --> STABLE; Interval From Baseline =110 months.

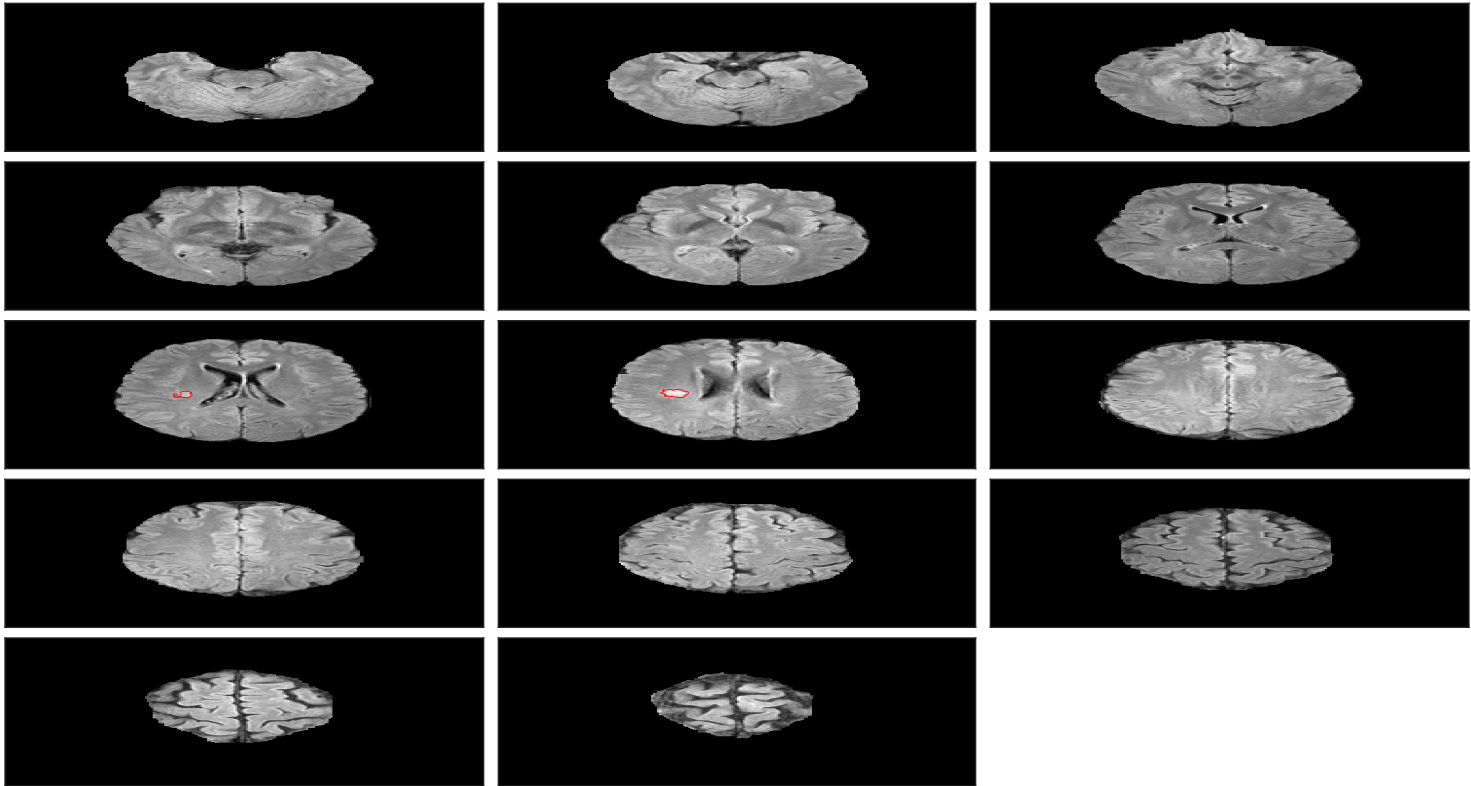

Supplement: S1 Data — The .zip file includes .pdf files labeled as follows, using case number 4224 as an example: 4224_t0.pdf includes the MRI data with segmentation of case number 4224 at baseline. 4224_t1_CAD_G_VC_S.pdf includes the MRI data with segmentation of case number 4224 at time 1, i.e., the time point of growth detection by the change-of-point method when the time point was different from the most recent MRI. 4224_tend_CAD_G_VC_G.pdf includes the MRI data with segmentation of case number 4224 at the most recent MRI. CAD_G and CAD_S mean that the change-of-point method predicted growth (G) or stability (S), respectively. VC_G and VC_S mean that the visual comparison detected growth (G) or stability (S), respectively. (ZIP) [file pmed.1002810.s001.zip › 7751_tend_CAD_S_VC_S.pdf]

Case Number:4388; TIME 1; CAD --> GROWTH; VC --> STABLE; Interval From Baseline =11 months.

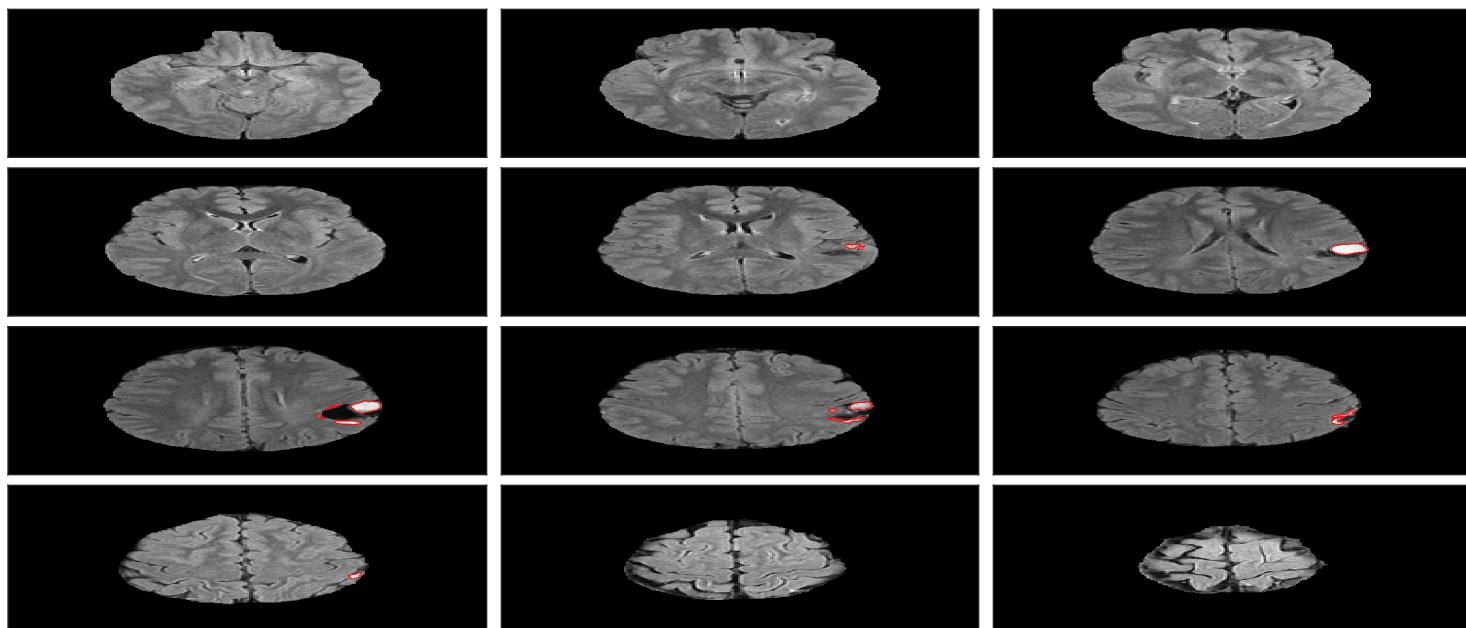

Supplement: S1 Data — The .zip file includes .pdf files labeled as follows, using case number 4224 as an example: 4224_t0.pdf includes the MRI data with segmentation of case number 4224 at baseline. 4224_t1_CAD_G_VC_S.pdf includes the MRI data with segmentation of case number 4224 at time 1, i.e., the time point of growth detection by the change-of-point method when the time point was different from the most recent MRI. 4224_tend_CAD_G_VC_G.pdf includes the MRI data with segmentation of case number 4224 at the most recent MRI. CAD_G and CAD_S mean that the change-of-point method predicted growth (G) or stability (S), respectively. VC_G and VC_S mean that the visual comparison detected growth (G) or stability (S), respectively. (ZIP) [file pmed.1002810.s001.zip › 4388_t1_CAD_G_VC_S.pdf]

Case Number:7732; Time 0 (Baseline).

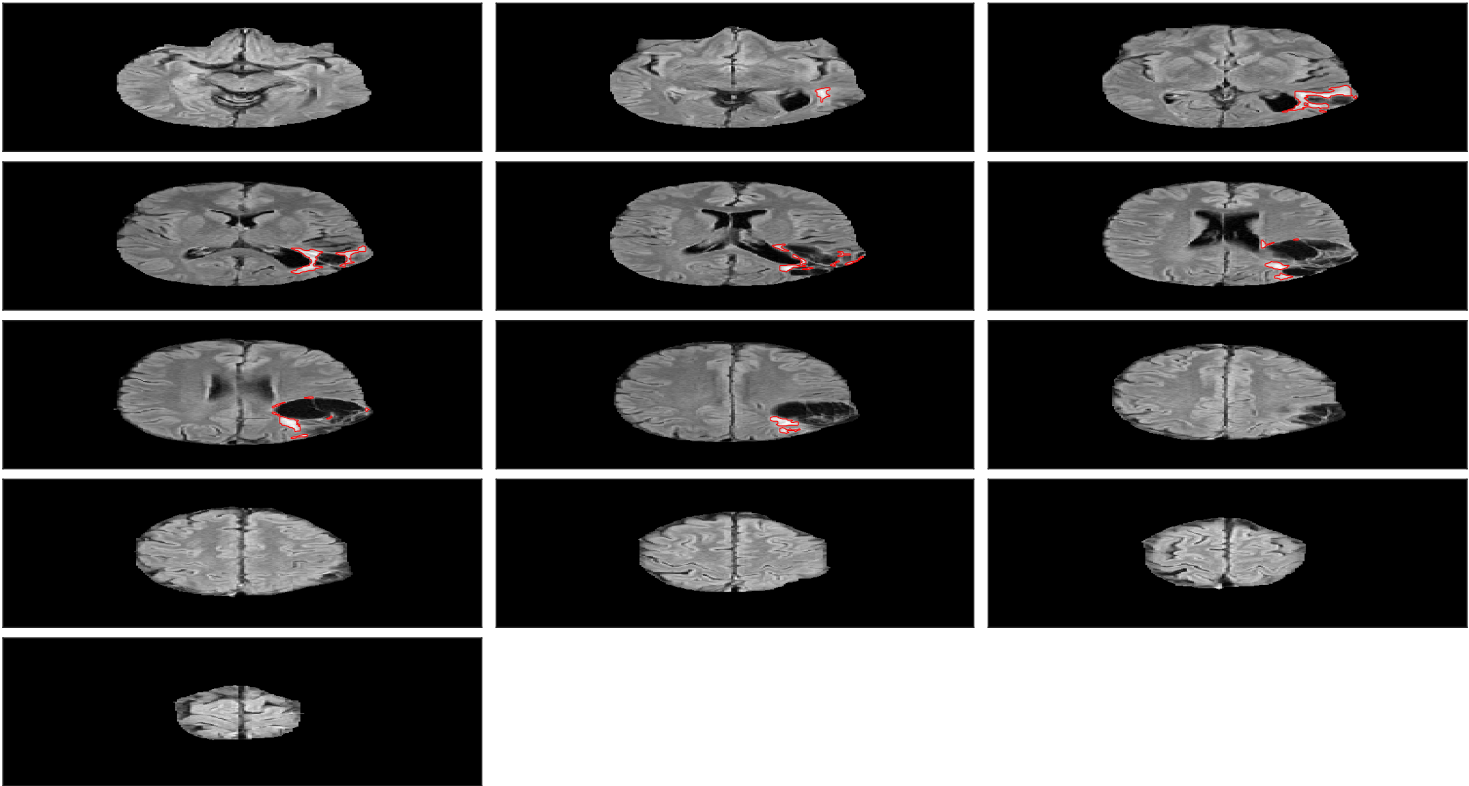

Supplement: S1 Data — The .zip file includes .pdf files labeled as follows, using case number 4224 as an example: 4224_t0.pdf includes the MRI data with segmentation of case number 4224 at baseline. 4224_t1_CAD_G_VC_S.pdf includes the MRI data with segmentation of case number 4224 at time 1, i.e., the time point of growth detection by the change-of-point method when the time point was different from the most recent MRI. 4224_tend_CAD_G_VC_G.pdf includes the MRI data with segmentation of case number 4224 at the most recent MRI. CAD_G and CAD_S mean that the change-of-point method predicted growth (G) or stability (S), respectively. VC_G and VC_S mean that the visual comparison detected growth (G) or stability (S), respectively. (ZIP) [file pmed.1002810.s001.zip › 7732_t0.pdf]

Case Number:4386; Time 0 (Baseline).

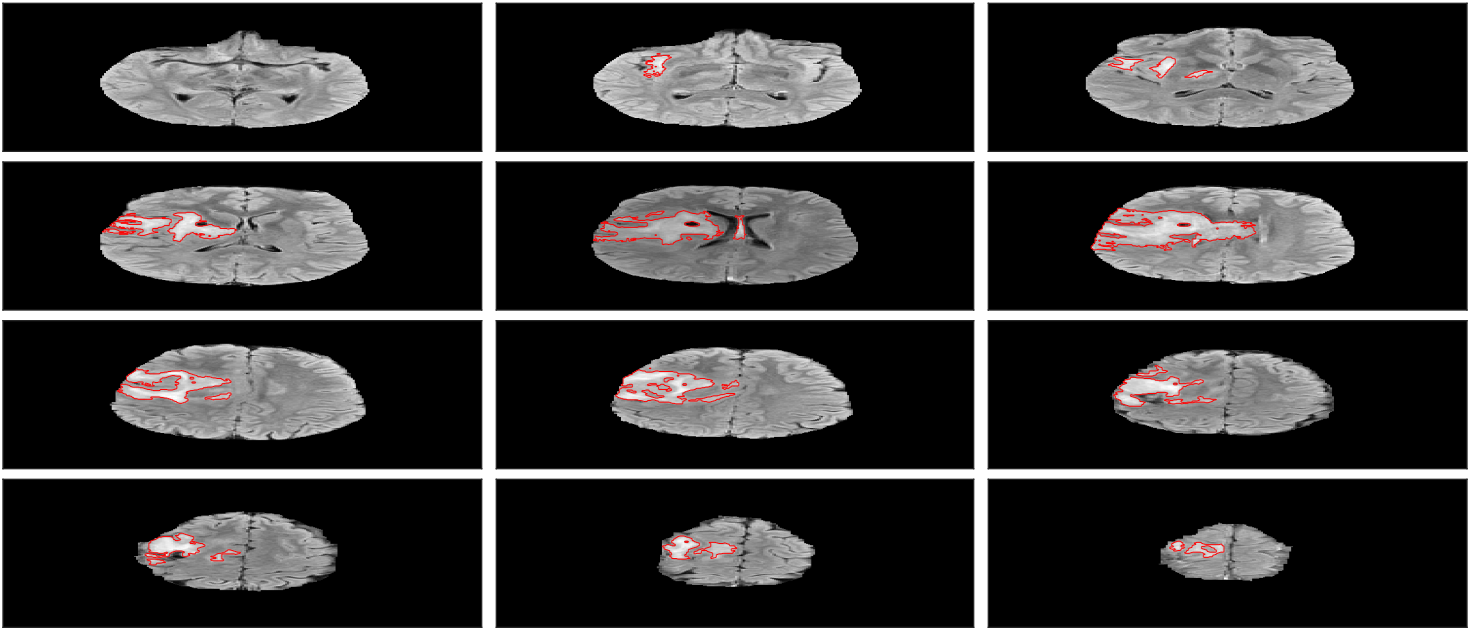

Supplement: S1 Data — The .zip file includes .pdf files labeled as follows, using case number 4224 as an example: 4224_t0.pdf includes the MRI data with segmentation of case number 4224 at baseline. 4224_t1_CAD_G_VC_S.pdf includes the MRI data with segmentation of case number 4224 at time 1, i.e., the time point of growth detection by the change-of-point method when the time point was different from the most recent MRI. 4224_tend_CAD_G_VC_G.pdf includes the MRI data with segmentation of case number 4224 at the most recent MRI. CAD_G and CAD_S mean that the change-of-point method predicted growth (G) or stability (S), respectively. VC_G and VC_S mean that the visual comparison detected growth (G) or stability (S), respectively. (ZIP) [file pmed.1002810.s001.zip › 4386_t0.pdf]

Case Number:7751; Time 0 (Baseline).

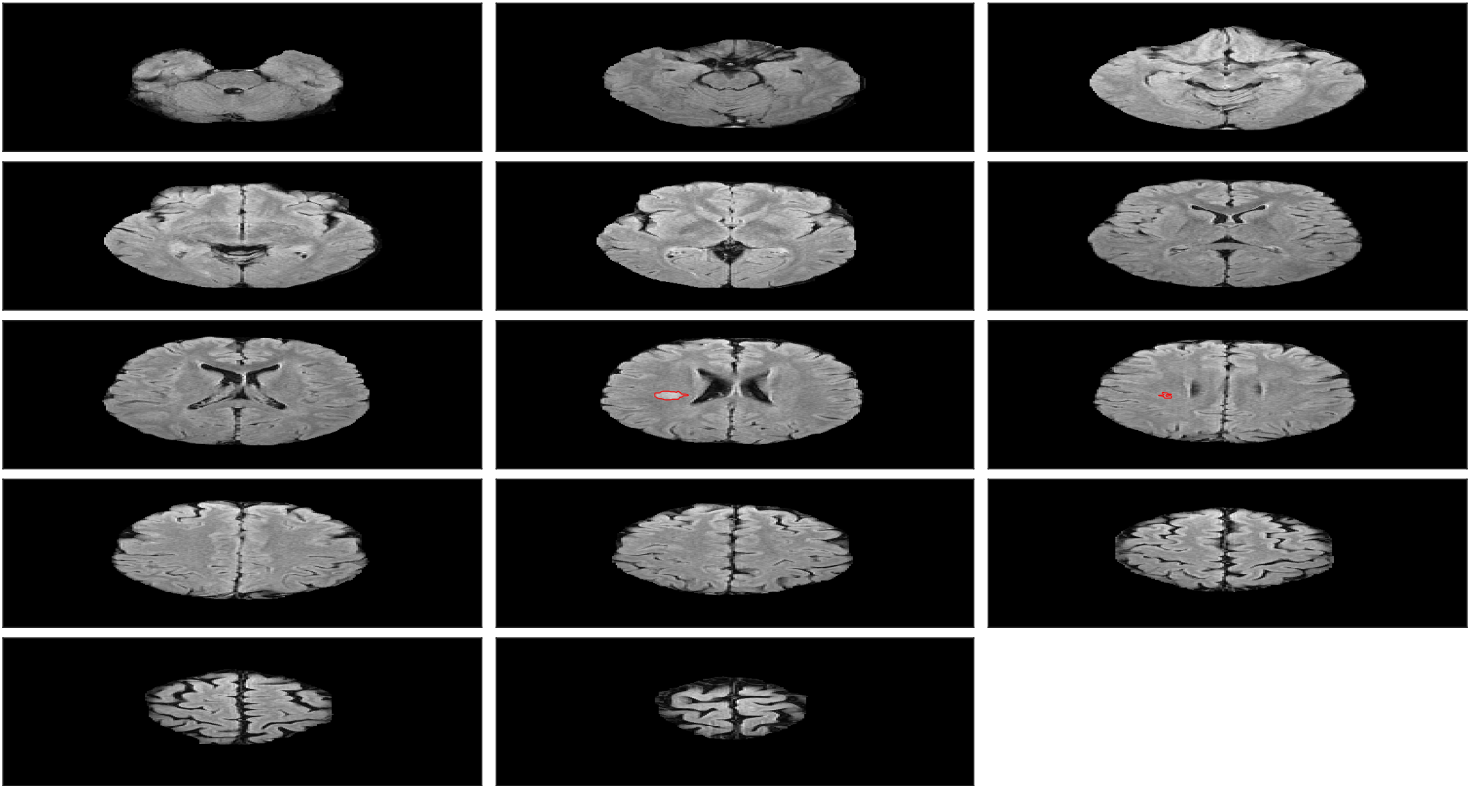

Supplement: S1 Data — The .zip file includes .pdf files labeled as follows, using case number 4224 as an example: 4224_t0.pdf includes the MRI data with segmentation of case number 4224 at baseline. 4224_t1_CAD_G_VC_S.pdf includes the MRI data with segmentation of case number 4224 at time 1, i.e., the time point of growth detection by the change-of-point method when the time point was different from the most recent MRI. 4224_tend_CAD_G_VC_G.pdf includes the MRI data with segmentation of case number 4224 at the most recent MRI. CAD_G and CAD_S mean that the change-of-point method predicted growth (G) or stability (S), respectively. VC_G and VC_S mean that the visual comparison detected growth (G) or stability (S), respectively. (ZIP) [file pmed.1002810.s001.zip › 7751_t0.pdf]

**Case Number:7736; TIME 1; CAD --> GROWTH; VC --> STABLE; Interval From Baseline =25 months.**

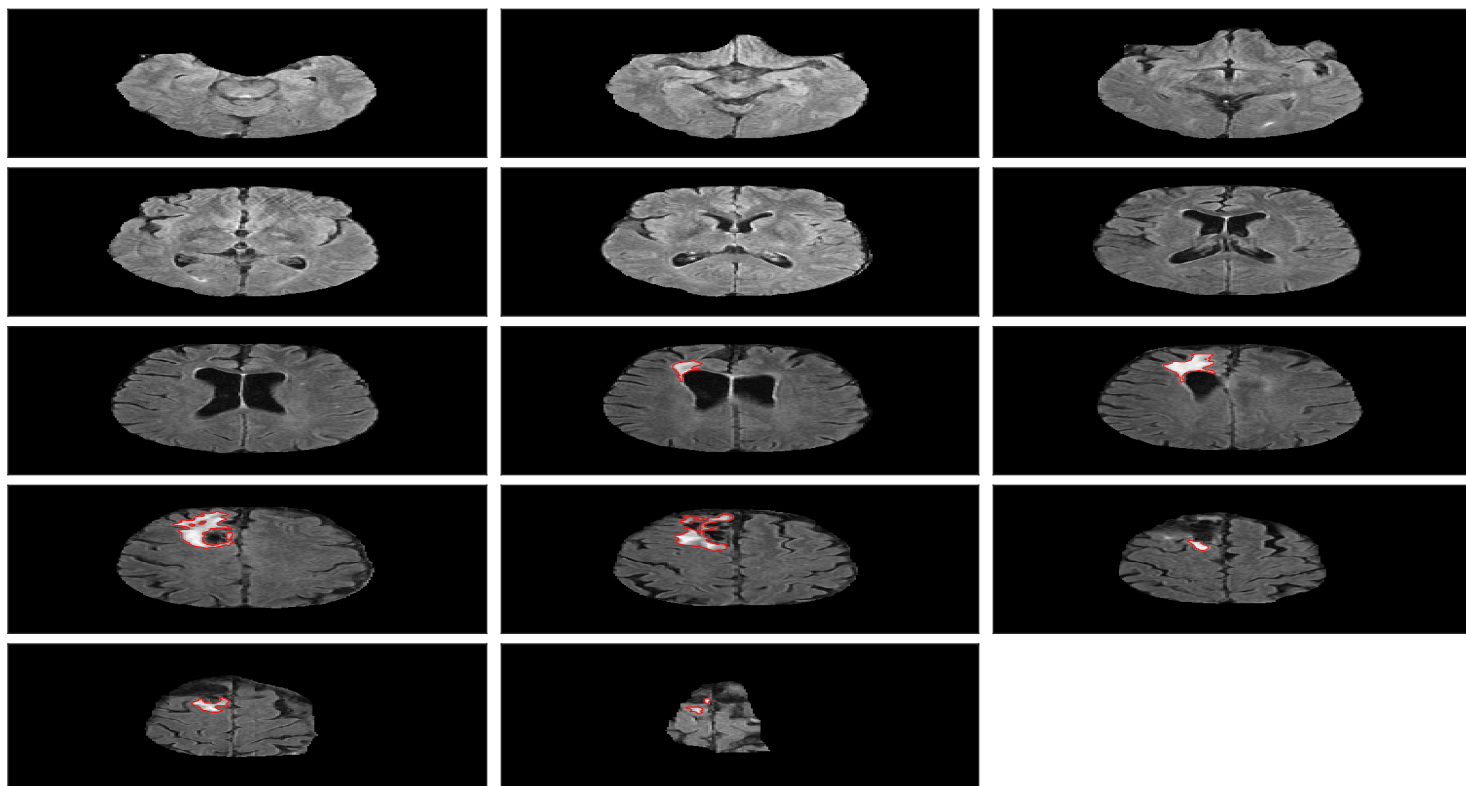

Supplement: S1 Data — The .zip file includes .pdf files labeled as follows, using case number 4224 as an example: 4224_t0.pdf includes the MRI data with segmentation of case number 4224 at baseline. 4224_t1_CAD_G_VC_S.pdf includes the MRI data with segmentation of case number 4224 at time 1, i.e., the time point of growth detection by the change-of-point method when the time point was different from the most recent MRI. 4224_tend_CAD_G_VC_G.pdf includes the MRI data with segmentation of case number 4224 at the most recent MRI. CAD_G and CAD_S mean that the change-of-point method predicted growth (G) or stability (S), respectively. VC_G and VC_S mean that the visual comparison detected growth (G) or stability (S), respectively. (ZIP) [file pmed.1002810.s001.zip › 7736_t1_CAD_G_VC_S.pdf]

Case Number:7489; Time 0 (Baseline).

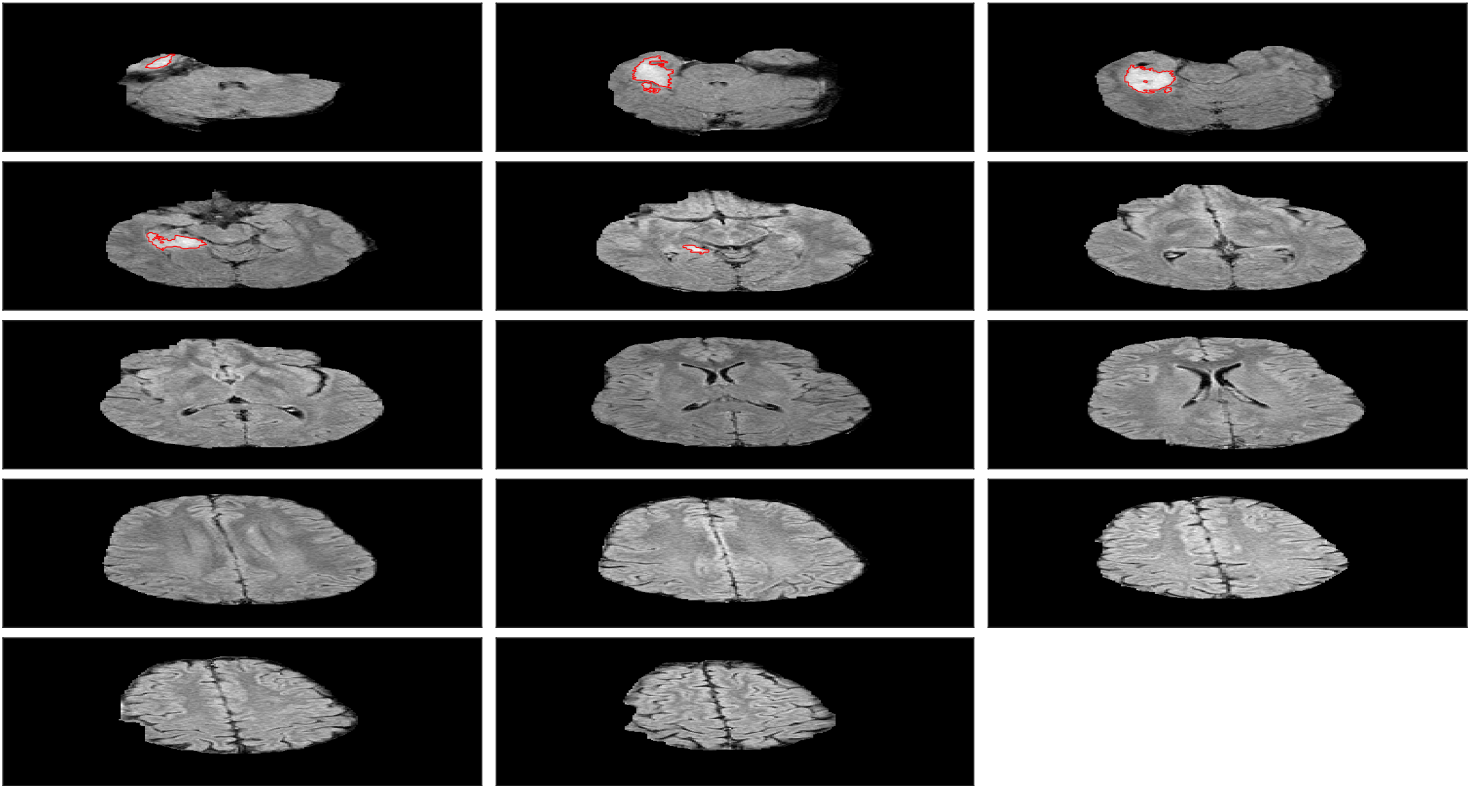

Supplement: S1 Data — The .zip file includes .pdf files labeled as follows, using case number 4224 as an example: 4224_t0.pdf includes the MRI data with segmentation of case number 4224 at baseline. 4224_t1_CAD_G_VC_S.pdf includes the MRI data with segmentation of case number 4224 at time 1, i.e., the time point of growth detection by the change-of-point method when the time point was different from the most recent MRI. 4224_tend_CAD_G_VC_G.pdf includes the MRI data with segmentation of case number 4224 at the most recent MRI. CAD_G and CAD_S mean that the change-of-point method predicted growth (G) or stability (S), respectively. VC_G and VC_S mean that the visual comparison detected growth (G) or stability (S), respectively. (ZIP) [file pmed.1002810.s001.zip › 7489_t0.pdf]

Case Number:7716; Time 0 (Baseline).

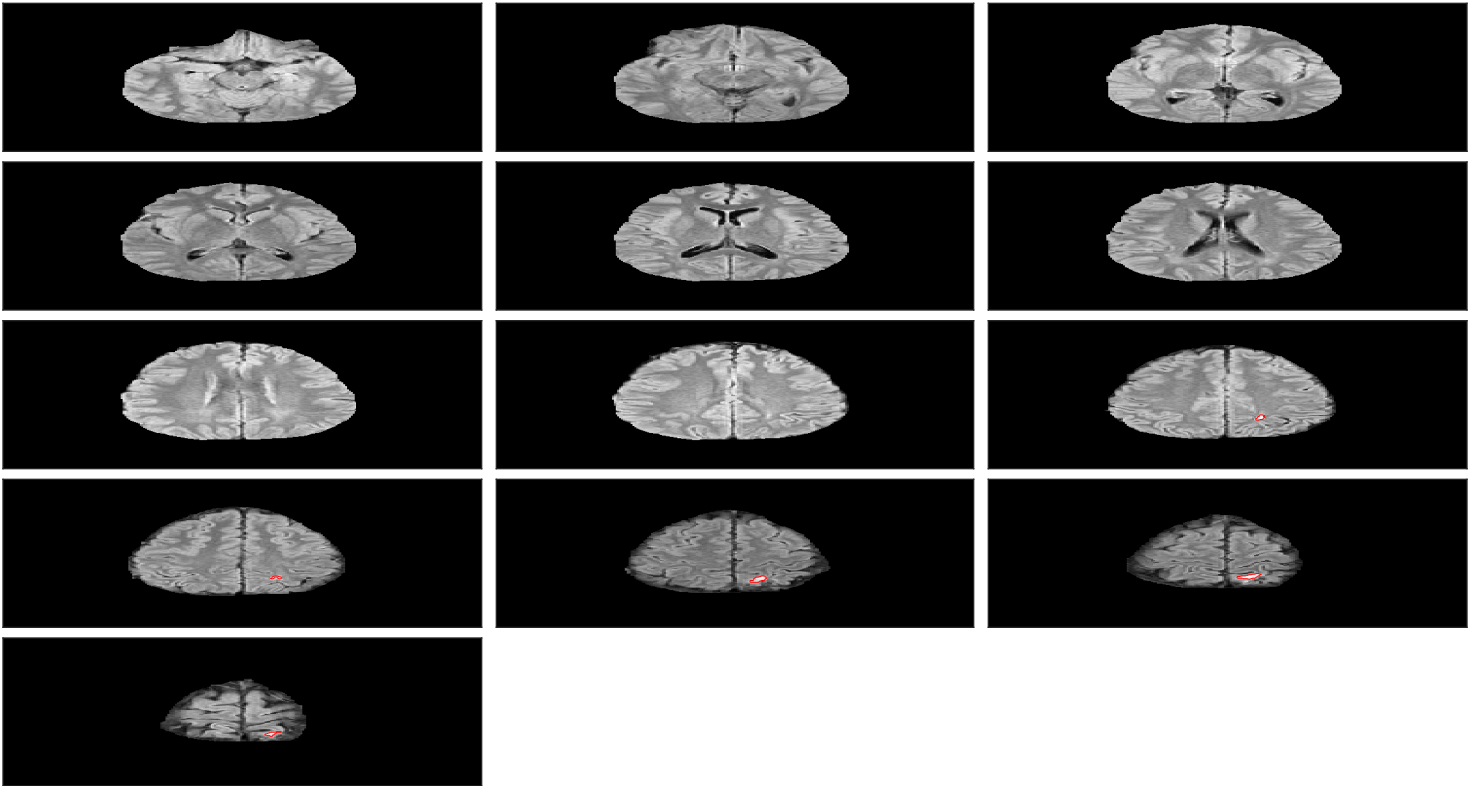

Supplement: S1 Data — The .zip file includes .pdf files labeled as follows, using case number 4224 as an example: 4224_t0.pdf includes the MRI data with segmentation of case number 4224 at baseline. 4224_t1_CAD_G_VC_S.pdf includes the MRI data with segmentation of case number 4224 at time 1, i.e., the time point of growth detection by the change-of-point method when the time point was different from the most recent MRI. 4224_tend_CAD_G_VC_G.pdf includes the MRI data with segmentation of case number 4224 at the most recent MRI. CAD_G and CAD_S mean that the change-of-point method predicted growth (G) or stability (S), respectively. VC_G and VC_S mean that the visual comparison detected growth (G) or stability (S), respectively. (ZIP) [file pmed.1002810.s001.zip › 7716_t0.pdf]

Case Number:7708; Time 0 (Baseline).

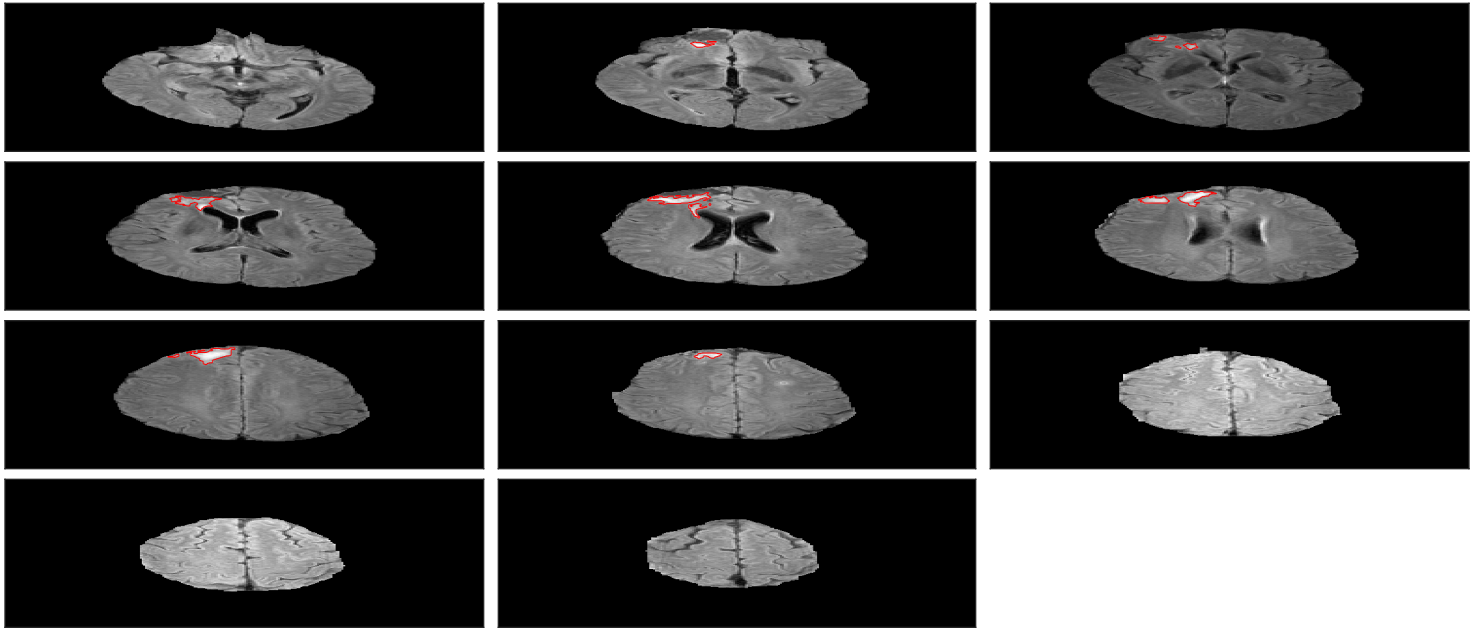

Supplement: S1 Data — The .zip file includes .pdf files labeled as follows, using case number 4224 as an example: 4224_t0.pdf includes the MRI data with segmentation of case number 4224 at baseline. 4224_t1_CAD_G_VC_S.pdf includes the MRI data with segmentation of case number 4224 at time 1, i.e., the time point of growth detection by the change-of-point method when the time point was different from the most recent MRI. 4224_tend_CAD_G_VC_G.pdf includes the MRI data with segmentation of case number 4224 at the most recent MRI. CAD_G and CAD_S mean that the change-of-point method predicted growth (G) or stability (S), respectively. VC_G and VC_S mean that the visual comparison detected growth (G) or stability (S), respectively. (ZIP) [file pmed.1002810.s001.zip › 7708_t0.pdf]

Case Number:7474; LAST MRI; CAD --> GROWTH; VC --> GROWTH; Interval From Baseline =32 months.

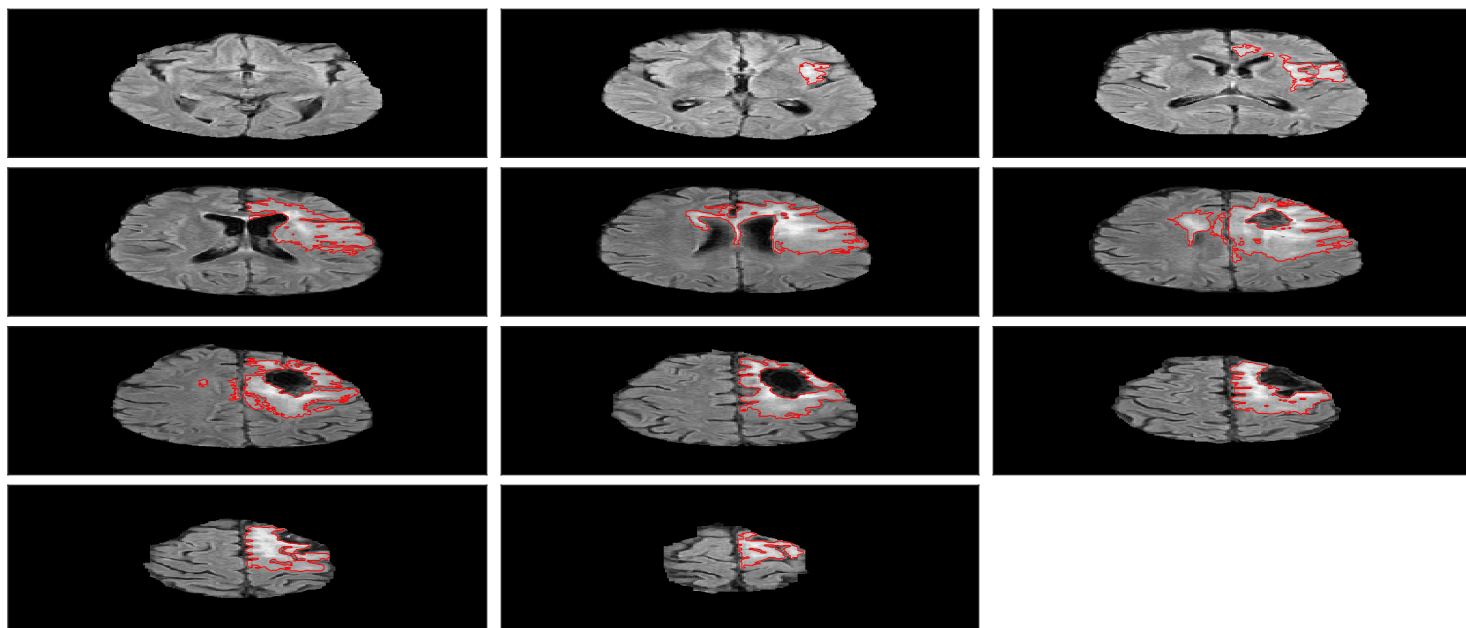

Supplement: S1 Data — The .zip file includes .pdf files labeled as follows, using case number 4224 as an example: 4224_t0.pdf includes the MRI data with segmentation of case number 4224 at baseline. 4224_t1_CAD_G_VC_S.pdf includes the MRI data with segmentation of case number 4224 at time 1, i.e., the time point of growth detection by the change-of-point method when the time point was different from the most recent MRI. 4224_tend_CAD_G_VC_G.pdf includes the MRI data with segmentation of case number 4224 at the most recent MRI. CAD_G and CAD_S mean that the change-of-point method predicted growth (G) or stability (S), respectively. VC_G and VC_S mean that the visual comparison detected growth (G) or stability (S), respectively. (ZIP) [file pmed.1002810.s001.zip › 7474_tend_CAD_G_VC_G.pdf]

Case Number:7749; LAST MRI; CAD --> STABLE; VC --> STABLE; Interval From Baseline =33 months.

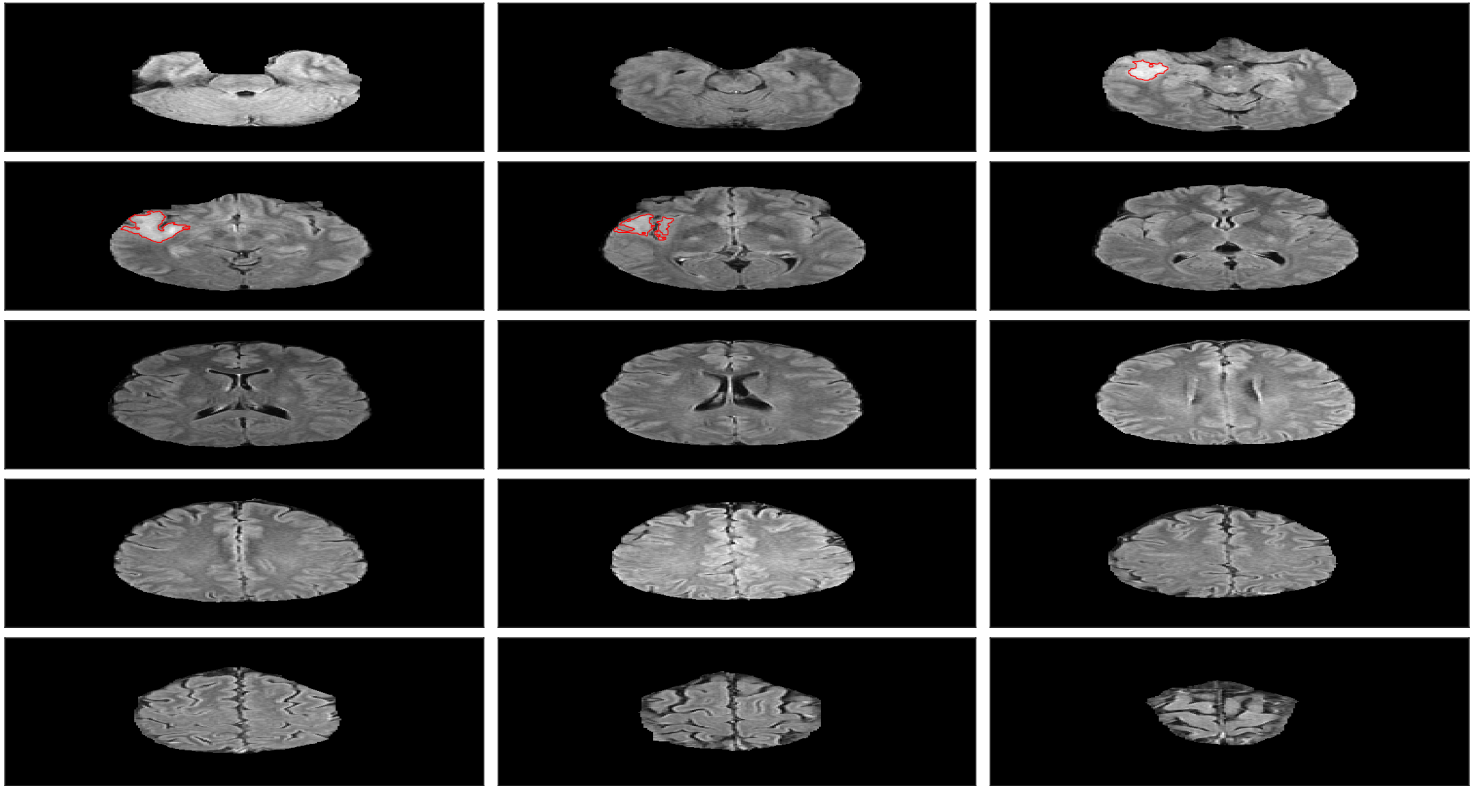

Supplement: S1 Data — The .zip file includes .pdf files labeled as follows, using case number 4224 as an example: 4224_t0.pdf includes the MRI data with segmentation of case number 4224 at baseline. 4224_t1_CAD_G_VC_S.pdf includes the MRI data with segmentation of case number 4224 at time 1, i.e., the time point of growth detection by the change-of-point method when the time point was different from the most recent MRI. 4224_tend_CAD_G_VC_G.pdf includes the MRI data with segmentation of case number 4224 at the most recent MRI. CAD_G and CAD_S mean that the change-of-point method predicted growth (G) or stability (S), respectively. VC_G and VC_S mean that the visual comparison detected growth (G) or stability (S), respectively. (ZIP) [file pmed.1002810.s001.zip › 7749_tend_CAD_S_VC_S.pdf]

Case Number:7707; LAST MRI; CAD --> GROWTH; VC --> GROWTH; Interval From Baseline =10 months.

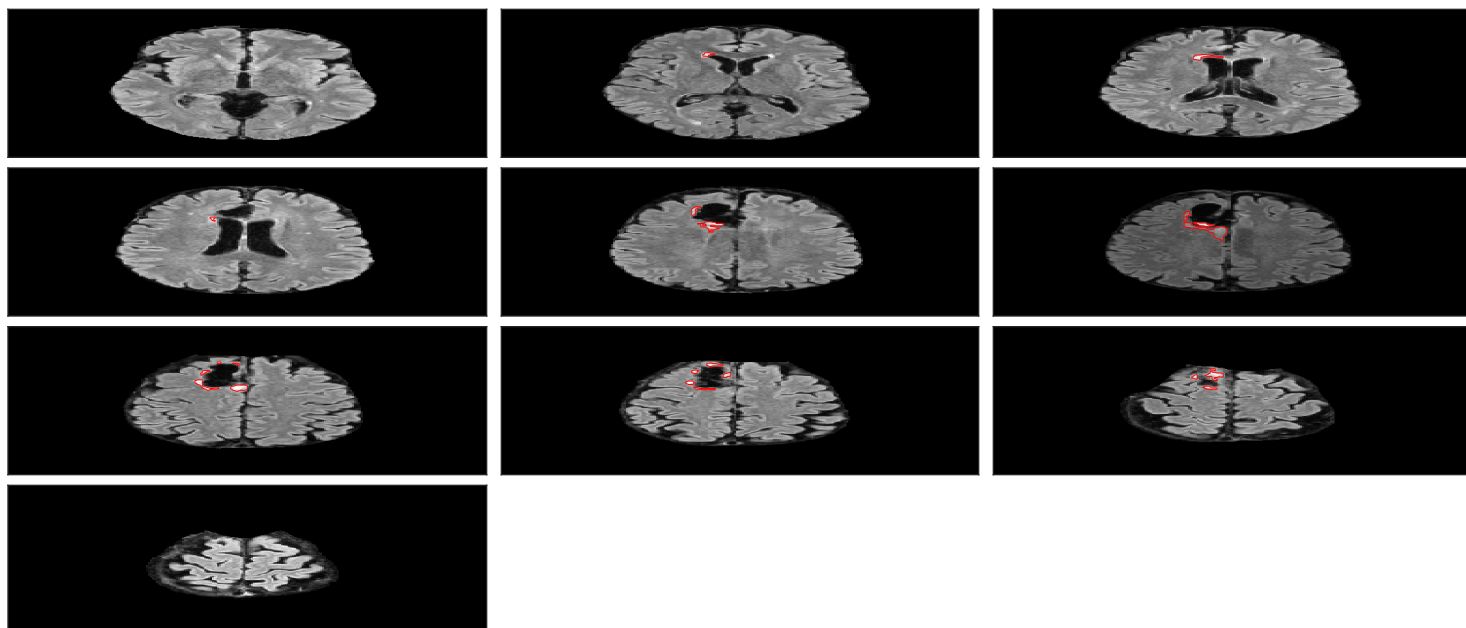

Supplement: S1 Data — The .zip file includes .pdf files labeled as follows, using case number 4224 as an example: 4224_t0.pdf includes the MRI data with segmentation of case number 4224 at baseline. 4224_t1_CAD_G_VC_S.pdf includes the MRI data with segmentation of case number 4224 at time 1, i.e., the time point of growth detection by the change-of-point method when the time point was different from the most recent MRI. 4224_tend_CAD_G_VC_G.pdf includes the MRI data with segmentation of case number 4224 at the most recent MRI. CAD_G and CAD_S mean that the change-of-point method predicted growth (G) or stability (S), respectively. VC_G and VC_S mean that the visual comparison detected growth (G) or stability (S), respectively. (ZIP) [file pmed.1002810.s001.zip › 7707_tend_CAD_G_VC_G.pdf]

Case Number:6937; TIME 1; CAD --> GROWTH; VC --> STABLE; Interval From Baseline =16 months.

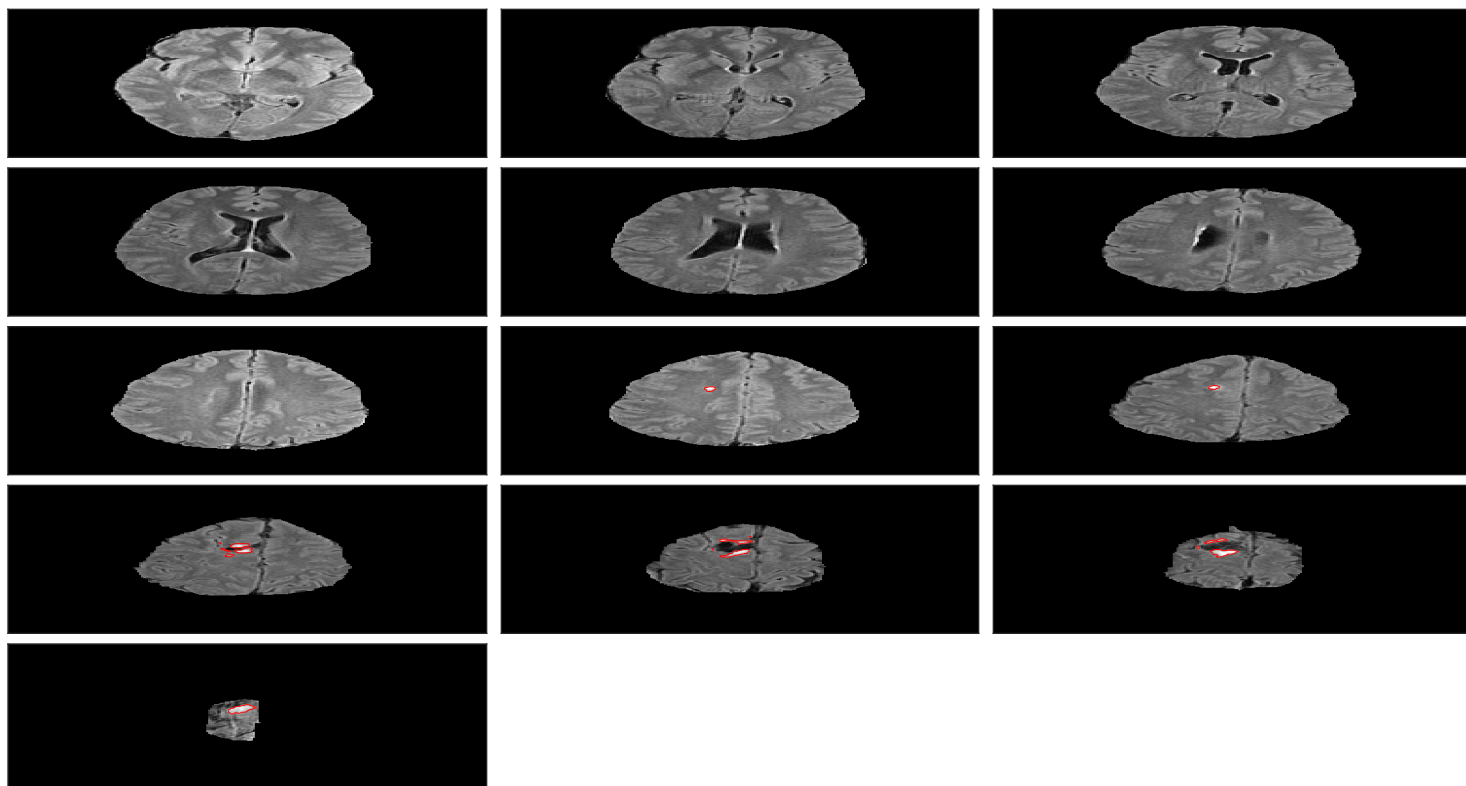

Supplement: S1 Data — The .zip file includes .pdf files labeled as follows, using case number 4224 as an example: 4224_t0.pdf includes the MRI data with segmentation of case number 4224 at baseline. 4224_t1_CAD_G_VC_S.pdf includes the MRI data with segmentation of case number 4224 at time 1, i.e., the time point of growth detection by the change-of-point method when the time point was different from the most recent MRI. 4224_tend_CAD_G_VC_G.pdf includes the MRI data with segmentation of case number 4224 at the most recent MRI. CAD_G and CAD_S mean that the change-of-point method predicted growth (G) or stability (S), respectively. VC_G and VC_S mean that the visual comparison detected growth (G) or stability (S), respectively. (ZIP) [file pmed.1002810.s001.zip › 6937_t1_CAD_G_VC_S.pdf]

Case Number:7506; LAST MRI; CAD --> STABLE; VC --> STABLE; Interval From Baseline =19 months.

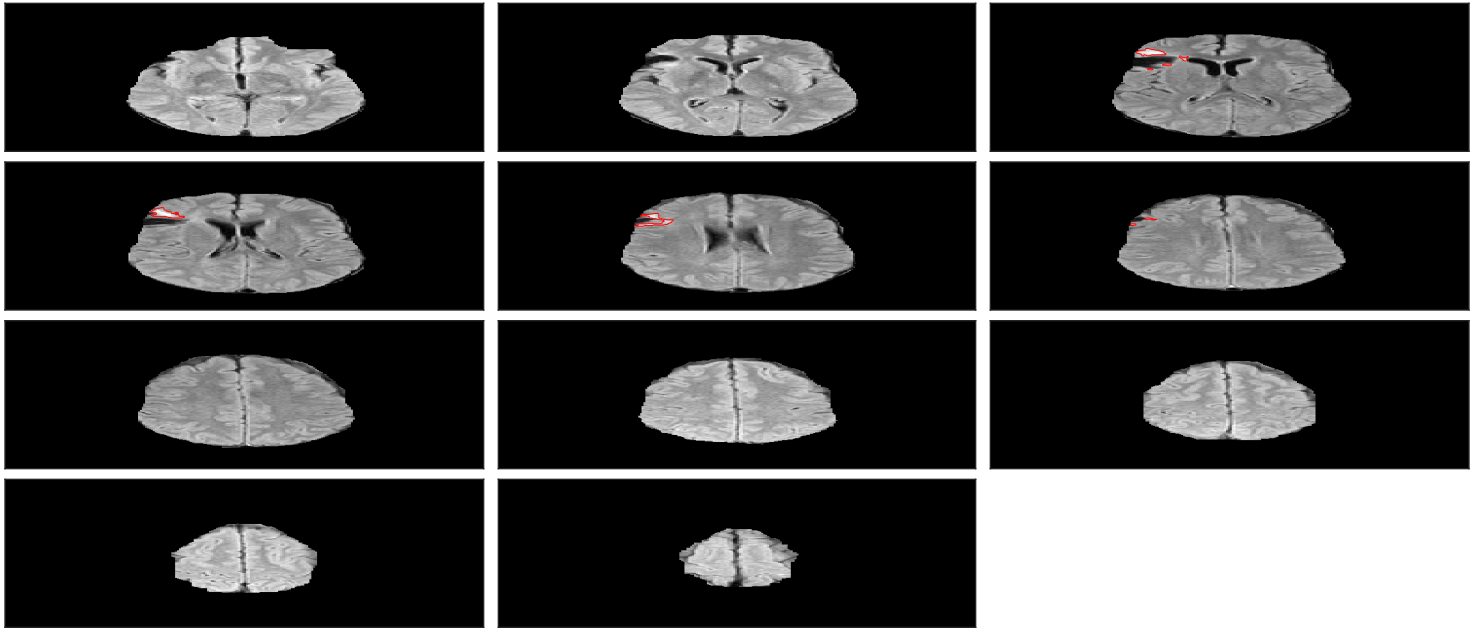

Supplement: S1 Data — The .zip file includes .pdf files labeled as follows, using case number 4224 as an example: 4224_t0.pdf includes the MRI data with segmentation of case number 4224 at baseline. 4224_t1_CAD_G_VC_S.pdf includes the MRI data with segmentation of case number 4224 at time 1, i.e., the time point of growth detection by the change-of-point method when the time point was different from the most recent MRI. 4224_tend_CAD_G_VC_G.pdf includes the MRI data with segmentation of case number 4224 at the most recent MRI. CAD_G and CAD_S mean that the change-of-point method predicted growth (G) or stability (S), respectively. VC_G and VC_S mean that the visual comparison detected growth (G) or stability (S), respectively. (ZIP) [file pmed.1002810.s001.zip › 7506_tend_CAD_S_VC_S.pdf]

Case Number:7094; LAST MRI; CAD --> GROWTH; VC --> GROWTH; Interval From Baseline =51 months.

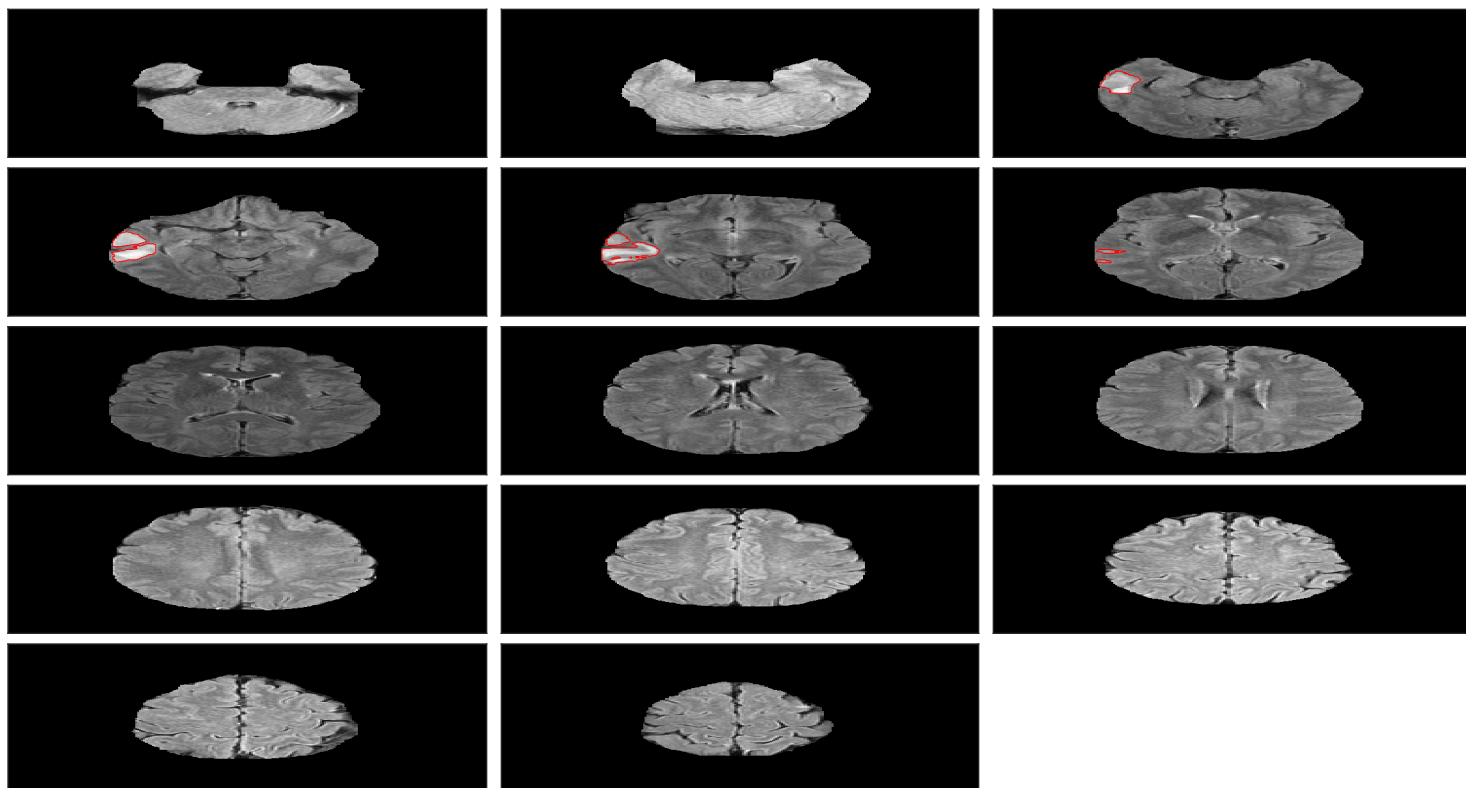

Supplement: S1 Data — The .zip file includes .pdf files labeled as follows, using case number 4224 as an example: 4224_t0.pdf includes the MRI data with segmentation of case number 4224 at baseline. 4224_t1_CAD_G_VC_S.pdf includes the MRI data with segmentation of case number 4224 at time 1, i.e., the time point of growth detection by the change-of-point method when the time point was different from the most recent MRI. 4224_tend_CAD_G_VC_G.pdf includes the MRI data with segmentation of case number 4224 at the most recent MRI. CAD_G and CAD_S mean that the change-of-point method predicted growth (G) or stability (S), respectively. VC_G and VC_S mean that the visual comparison detected growth (G) or stability (S), respectively. (ZIP) [file pmed.1002810.s001.zip › 7094_tend_CAD_G_VC_G.pdf]

Case Number:7335; Time 0 (Baseline).

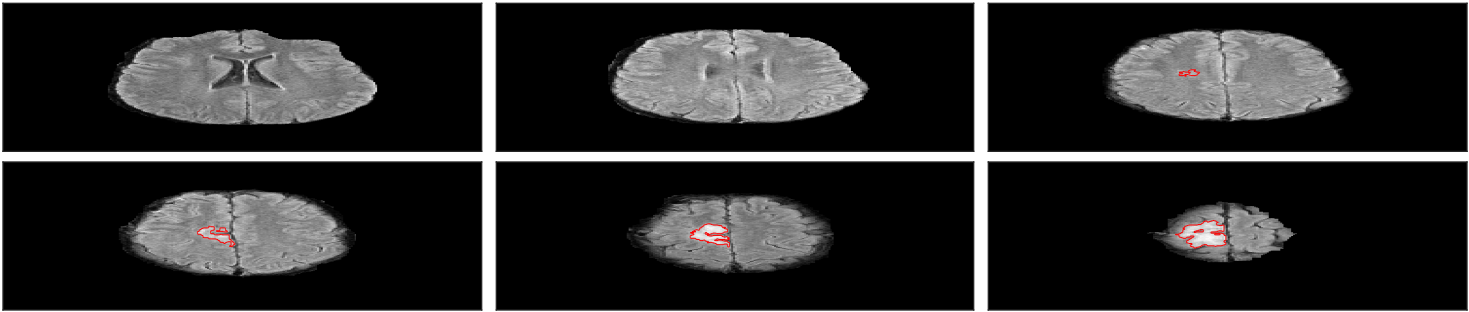

Supplement: S1 Data — The .zip file includes .pdf files labeled as follows, using case number 4224 as an example: 4224_t0.pdf includes the MRI data with segmentation of case number 4224 at baseline. 4224_t1_CAD_G_VC_S.pdf includes the MRI data with segmentation of case number 4224 at time 1, i.e., the time point of growth detection by the change-of-point method when the time point was different from the most recent MRI. 4224_tend_CAD_G_VC_G.pdf includes the MRI data with segmentation of case number 4224 at the most recent MRI. CAD_G and CAD_S mean that the change-of-point method predicted growth (G) or stability (S), respectively. VC_G and VC_S mean that the visual comparison detected growth (G) or stability (S), respectively. (ZIP) [file pmed.1002810.s001.zip › 7335_t0.pdf]

Case Number:7477; LAST MRI; CAD --> GROWTH; VC --> GROWTH; Interval From Baseline =26 months.

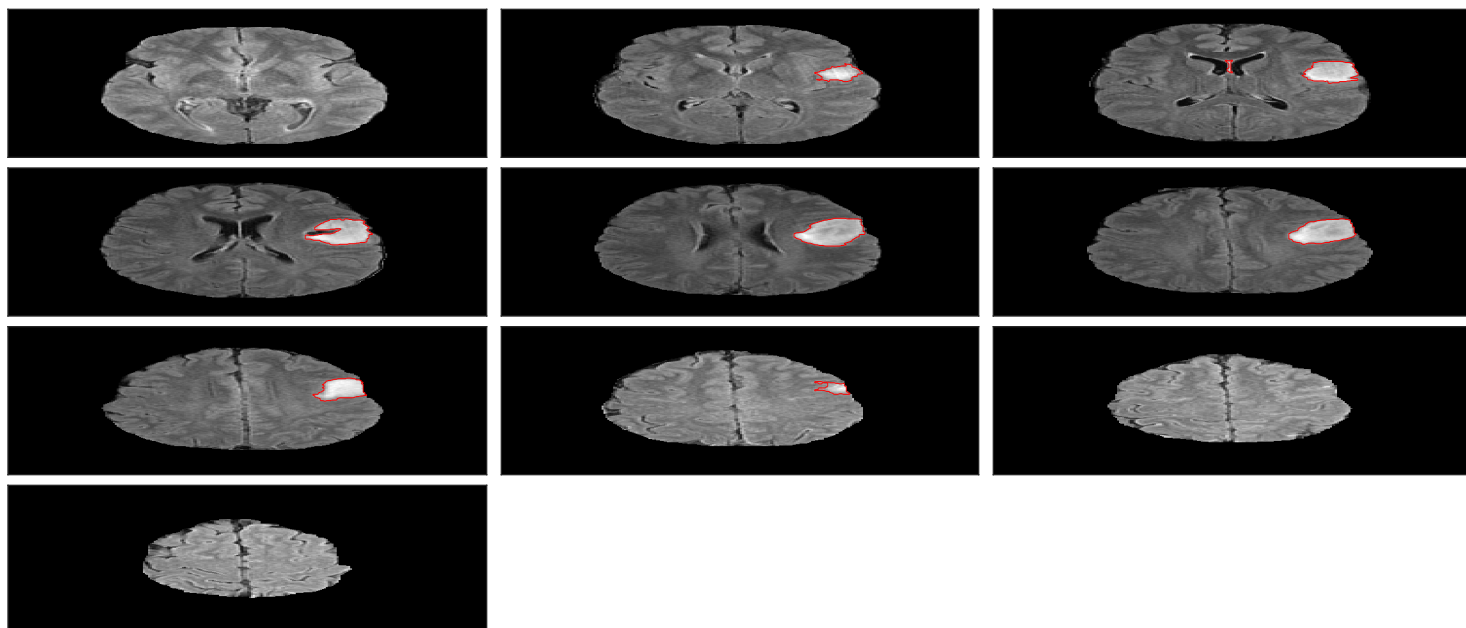

Supplement: S1 Data — The .zip file includes .pdf files labeled as follows, using case number 4224 as an example: 4224_t0.pdf includes the MRI data with segmentation of case number 4224 at baseline. 4224_t1_CAD_G_VC_S.pdf includes the MRI data with segmentation of case number 4224 at time 1, i.e., the time point of growth detection by the change-of-point method when the time point was different from the most recent MRI. 4224_tend_CAD_G_VC_G.pdf includes the MRI data with segmentation of case number 4224 at the most recent MRI. CAD_G and CAD_S mean that the change-of-point method predicted growth (G) or stability (S), respectively. VC_G and VC_S mean that the visual comparison detected growth (G) or stability (S), respectively. (ZIP) [file pmed.1002810.s001.zip › 7477_tend_CAD_G_VC_G.pdf]

Case Number:7753; LAST MRI; CAD --> STABLE; VC --> STABLE; Interval From Baseline =99 months.

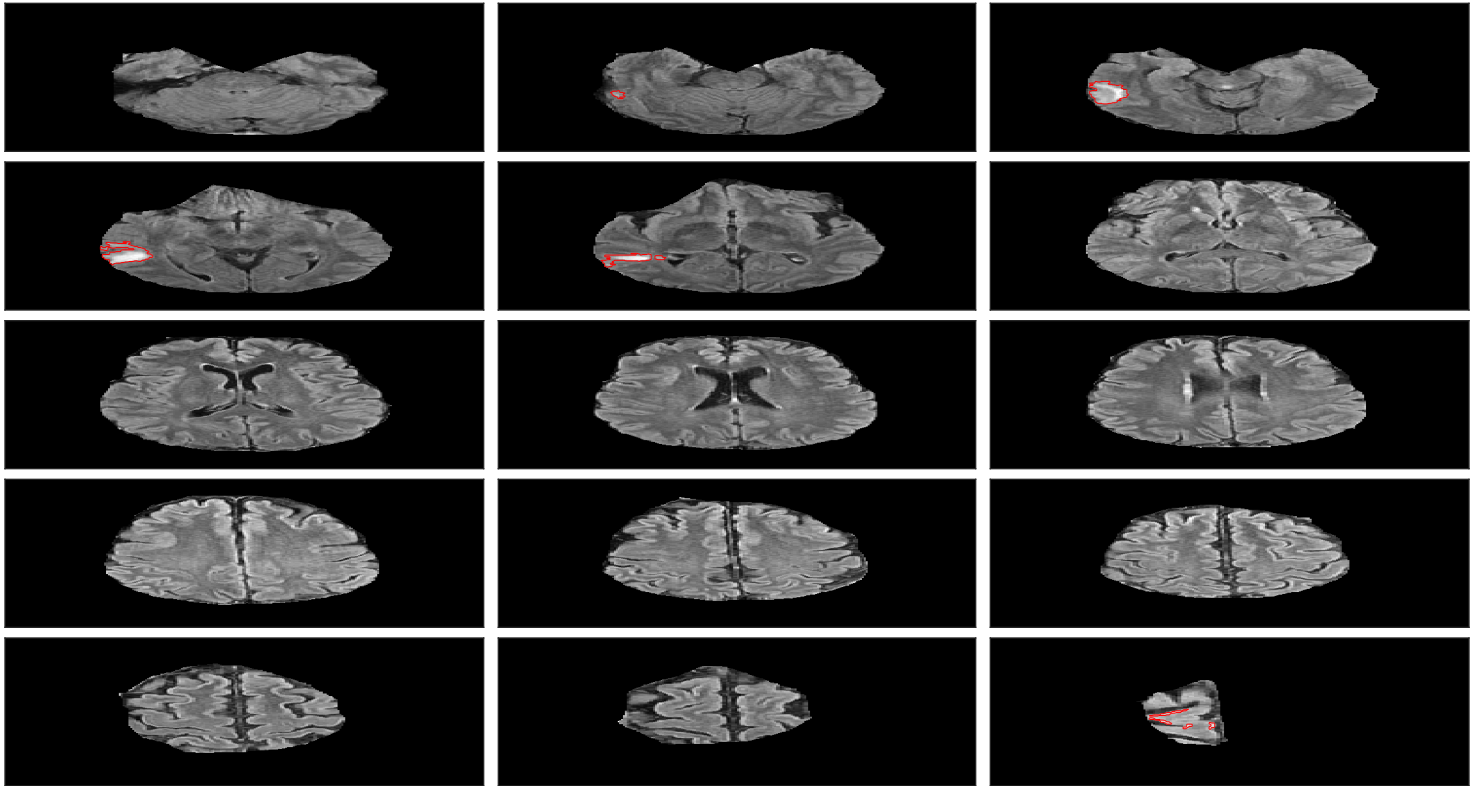

Supplement: S1 Data — The .zip file includes .pdf files labeled as follows, using case number 4224 as an example: 4224_t0.pdf includes the MRI data with segmentation of case number 4224 at baseline. 4224_t1_CAD_G_VC_S.pdf includes the MRI data with segmentation of case number 4224 at time 1, i.e., the time point of growth detection by the change-of-point method when the time point was different from the most recent MRI. 4224_tend_CAD_G_VC_G.pdf includes the MRI data with segmentation of case number 4224 at the most recent MRI. CAD_G and CAD_S mean that the change-of-point method predicted growth (G) or stability (S), respectively. VC_G and VC_S mean that the visual comparison detected growth (G) or stability (S), respectively. (ZIP) [file pmed.1002810.s001.zip › 7753_tend_CAD_S_VC_S.pdf]

Case Number:7476; Time 0 (Baseline).

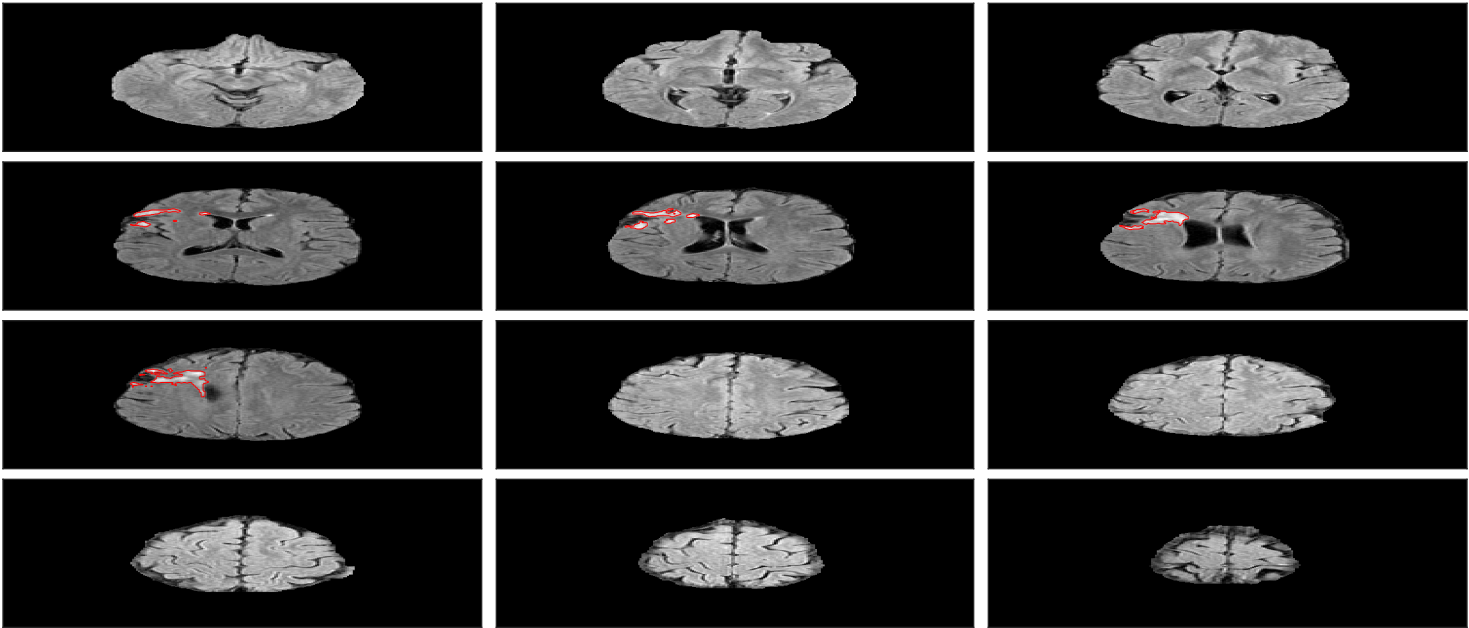

Supplement: S1 Data — The .zip file includes .pdf files labeled as follows, using case number 4224 as an example: 4224_t0.pdf includes the MRI data with segmentation of case number 4224 at baseline. 4224_t1_CAD_G_VC_S.pdf includes the MRI data with segmentation of case number 4224 at time 1, i.e., the time point of growth detection by the change-of-point method when the time point was different from the most recent MRI. 4224_tend_CAD_G_VC_G.pdf includes the MRI data with segmentation of case number 4224 at the most recent MRI. CAD_G and CAD_S mean that the change-of-point method predicted growth (G) or stability (S), respectively. VC_G and VC_S mean that the visual comparison detected growth (G) or stability (S), respectively. (ZIP) [file pmed.1002810.s001.zip › 7476_t0.pdf]

Case Number:7733; TIME 1; CAD --> GROWTH; VC --> STABLE; Interval From Baseline =15 months.

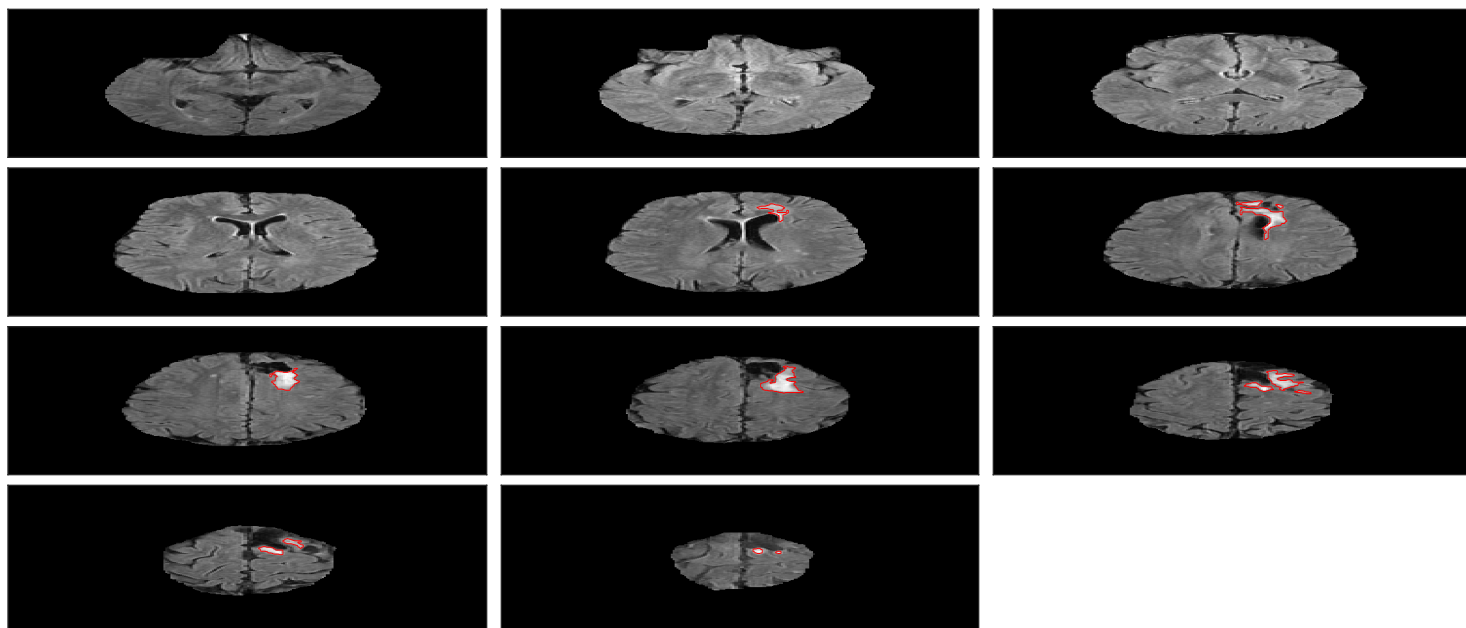

Supplement: S1 Data — The .zip file includes .pdf files labeled as follows, using case number 4224 as an example: 4224_t0.pdf includes the MRI data with segmentation of case number 4224 at baseline. 4224_t1_CAD_G_VC_S.pdf includes the MRI data with segmentation of case number 4224 at time 1, i.e., the time point of growth detection by the change-of-point method when the time point was different from the most recent MRI. 4224_tend_CAD_G_VC_G.pdf includes the MRI data with segmentation of case number 4224 at the most recent MRI. CAD_G and CAD_S mean that the change-of-point method predicted growth (G) or stability (S), respectively. VC_G and VC_S mean that the visual comparison detected growth (G) or stability (S), respectively. (ZIP) [file pmed.1002810.s001.zip › 7733_t1_CAD_G_VC_S.pdf]

Case Number:7478; TIME 1; CAD --> GROWTH; VC --> STABLE; Interval From Baseline =20 months.

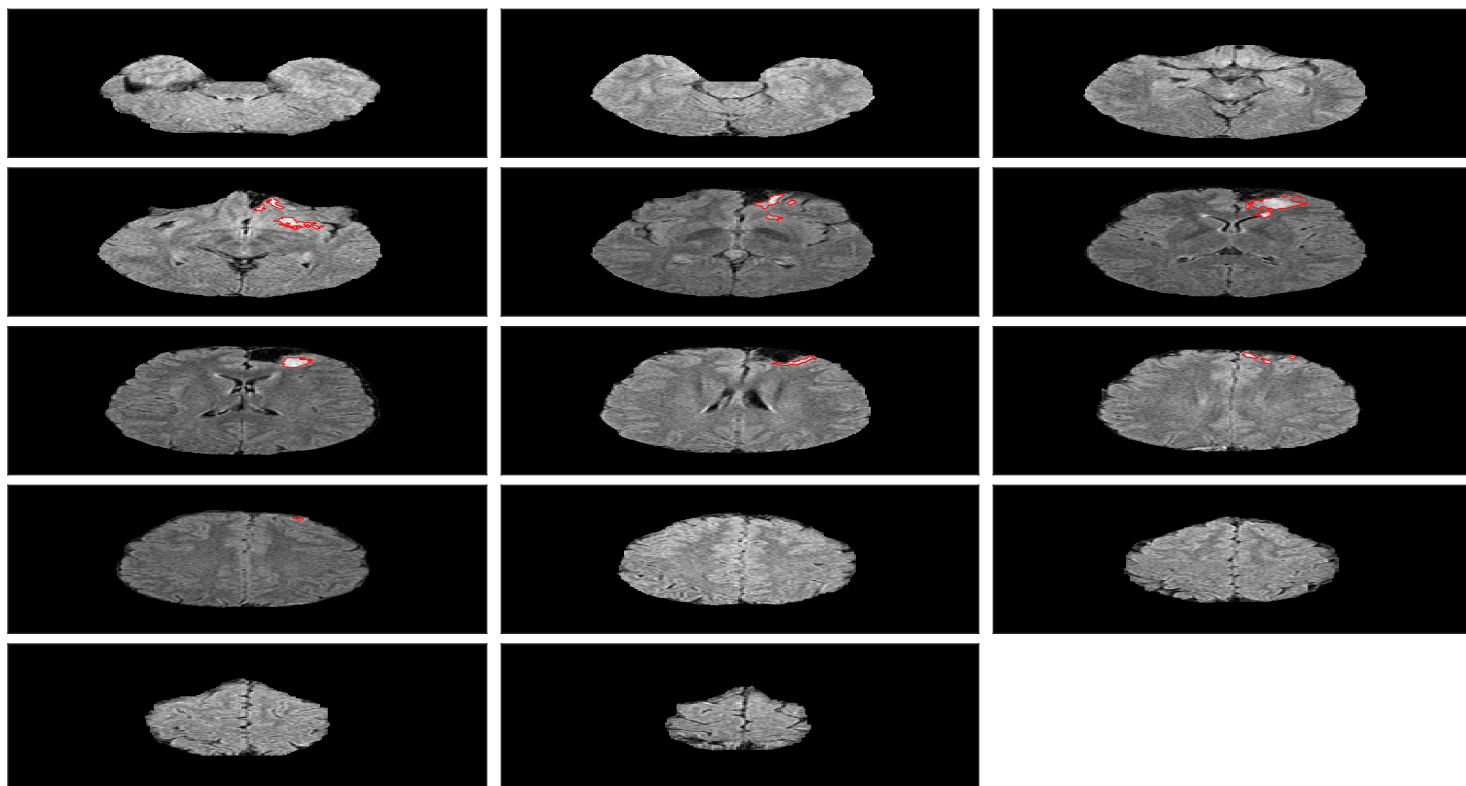

Supplement: S1 Data — The .zip file includes .pdf files labeled as follows, using case number 4224 as an example: 4224_t0.pdf includes the MRI data with segmentation of case number 4224 at baseline. 4224_t1_CAD_G_VC_S.pdf includes the MRI data with segmentation of case number 4224 at time 1, i.e., the time point of growth detection by the change-of-point method when the time point was different from the most recent MRI. 4224_tend_CAD_G_VC_G.pdf includes the MRI data with segmentation of case number 4224 at the most recent MRI. CAD_G and CAD_S mean that the change-of-point method predicted growth (G) or stability (S), respectively. VC_G and VC_S mean that the visual comparison detected growth (G) or stability (S), respectively. (ZIP) [file pmed.1002810.s001.zip › 7478_t1_CAD_G_VC_S.pdf]

Case Number:7752; LAST MRI; CAD --> STABLE; VC --> STABLE; Interval From Baseline =25 months.

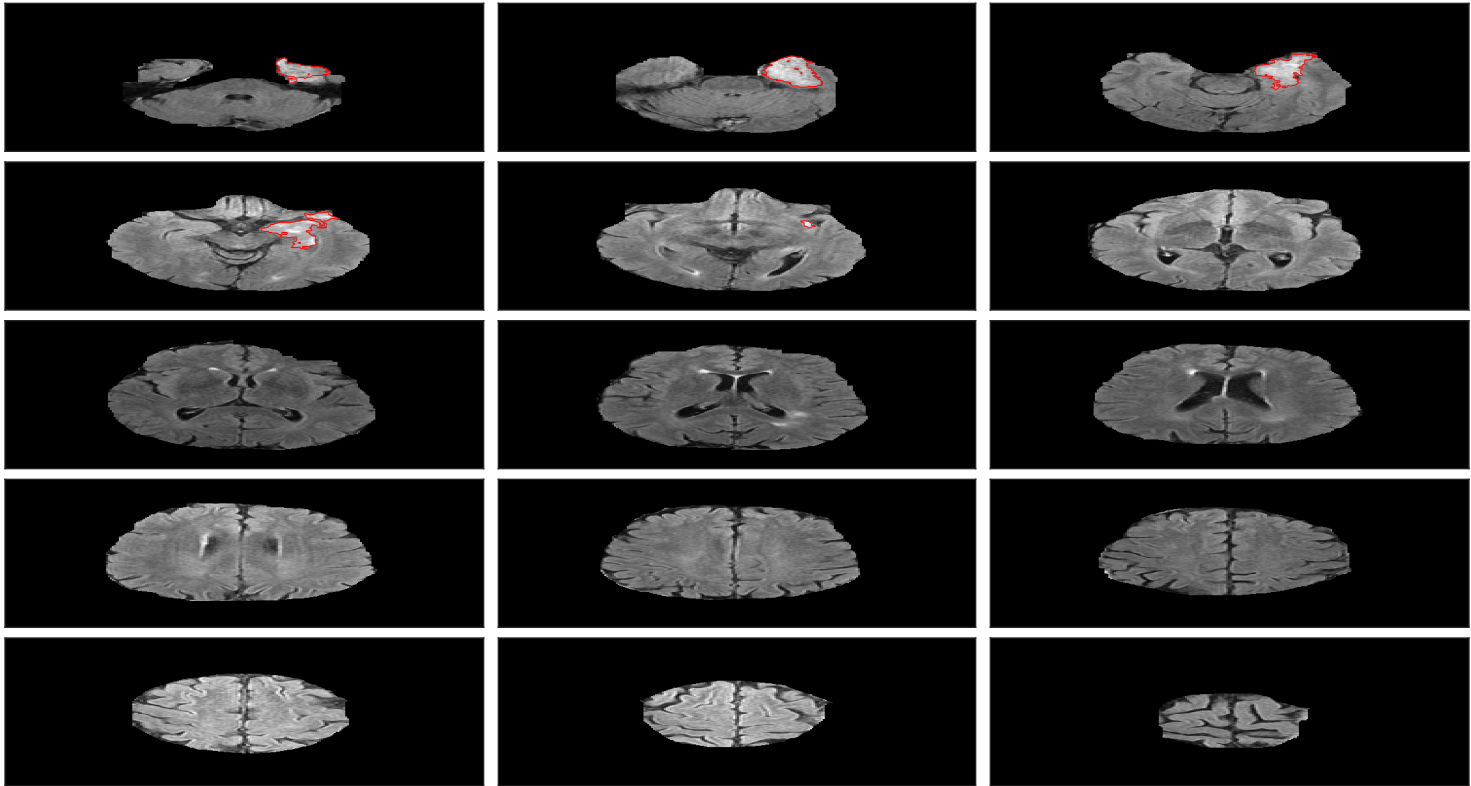

Supplement: S1 Data — The .zip file includes .pdf files labeled as follows, using case number 4224 as an example: 4224_t0.pdf includes the MRI data with segmentation of case number 4224 at baseline. 4224_t1_CAD_G_VC_S.pdf includes the MRI data with segmentation of case number 4224 at time 1, i.e., the time point of growth detection by the change-of-point method when the time point was different from the most recent MRI. 4224_tend_CAD_G_VC_G.pdf includes the MRI data with segmentation of case number 4224 at the most recent MRI. CAD_G and CAD_S mean that the change-of-point method predicted growth (G) or stability (S), respectively. VC_G and VC_S mean that the visual comparison detected growth (G) or stability (S), respectively. (ZIP) [file pmed.1002810.s001.zip › 7752_tend_CAD_S_VC_S.pdf]

Case Number:7476; LAST MRI; CAD --> GROWTH; VC --> GROWTH; Interval From Baseline =28 months.

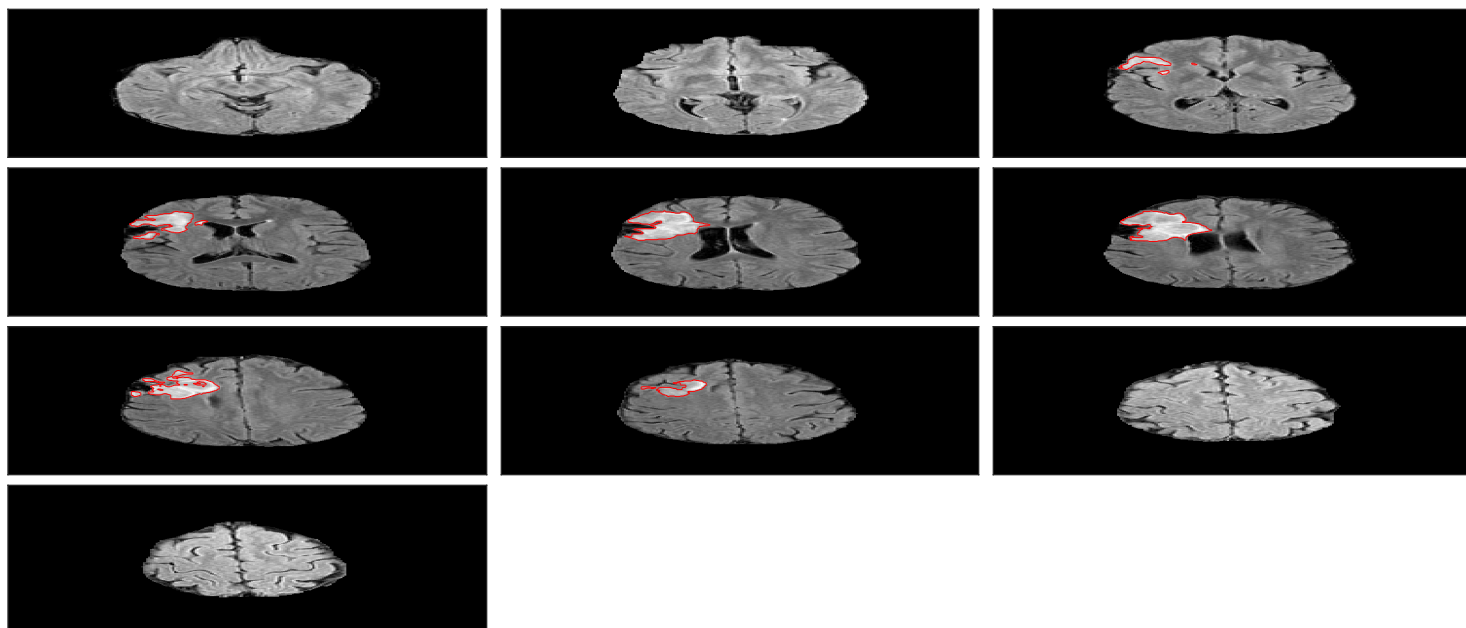

Supplement: S1 Data — The .zip file includes .pdf files labeled as follows, using case number 4224 as an example: 4224_t0.pdf includes the MRI data with segmentation of case number 4224 at baseline. 4224_t1_CAD_G_VC_S.pdf includes the MRI data with segmentation of case number 4224 at time 1, i.e., the time point of growth detection by the change-of-point method when the time point was different from the most recent MRI. 4224_tend_CAD_G_VC_G.pdf includes the MRI data with segmentation of case number 4224 at the most recent MRI. CAD_G and CAD_S mean that the change-of-point method predicted growth (G) or stability (S), respectively. VC_G and VC_S mean that the visual comparison detected growth (G) or stability (S), respectively. (ZIP) [file pmed.1002810.s001.zip › 7476_tend_CAD_G_VC_G.pdf]

Case Number:7493; LAST MRI; CAD --> GROWTH; VC --> STABLE; Interval From Baseline =22 months.

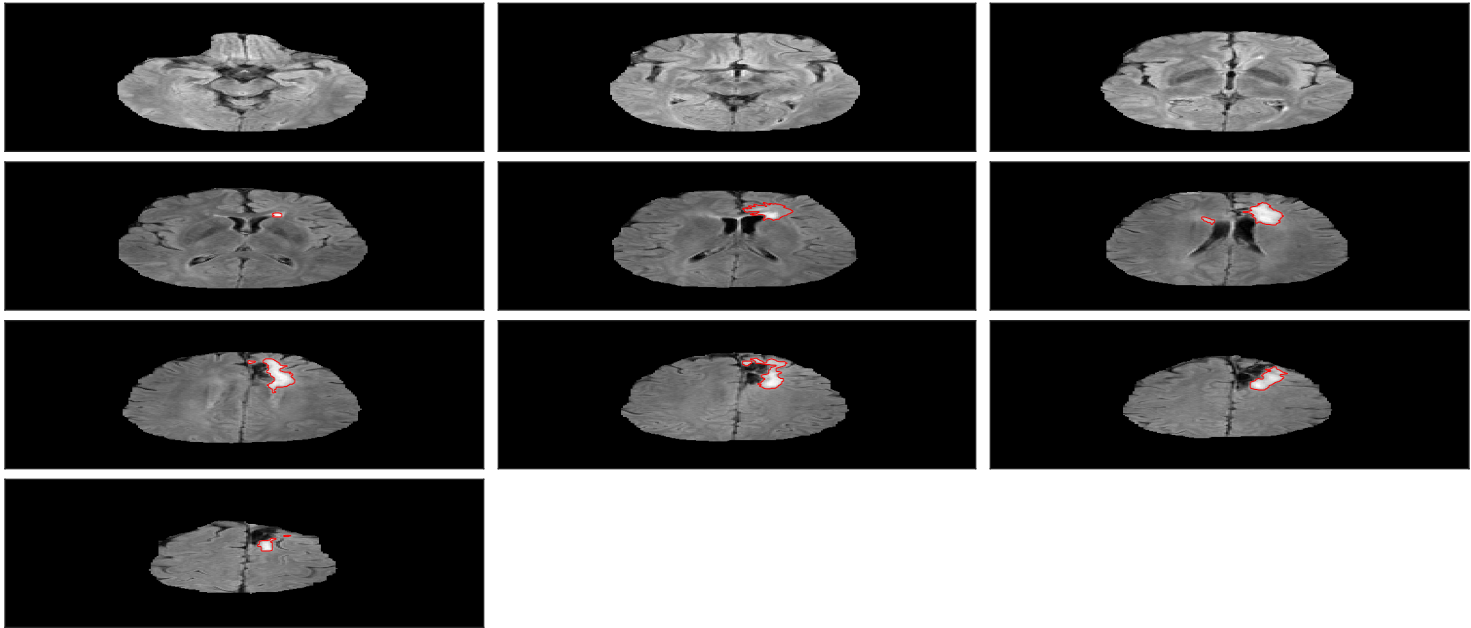

Supplement: S1 Data — The .zip file includes .pdf files labeled as follows, using case number 4224 as an example: 4224_t0.pdf includes the MRI data with segmentation of case number 4224 at baseline. 4224_t1_CAD_G_VC_S.pdf includes the MRI data with segmentation of case number 4224 at time 1, i.e., the time point of growth detection by the change-of-point method when the time point was different from the most recent MRI. 4224_tend_CAD_G_VC_G.pdf includes the MRI data with segmentation of case number 4224 at the most recent MRI. CAD_G and CAD_S mean that the change-of-point method predicted growth (G) or stability (S), respectively. VC_G and VC_S mean that the visual comparison detected growth (G) or stability (S), respectively. (ZIP) [file pmed.1002810.s001.zip › 7493_tend_CAD_G_VC_S.pdf]

Case Number:7711; LAST MRI; CAD --> GROWTH; VC --> GROWTH; Interval From Baseline =9 months.

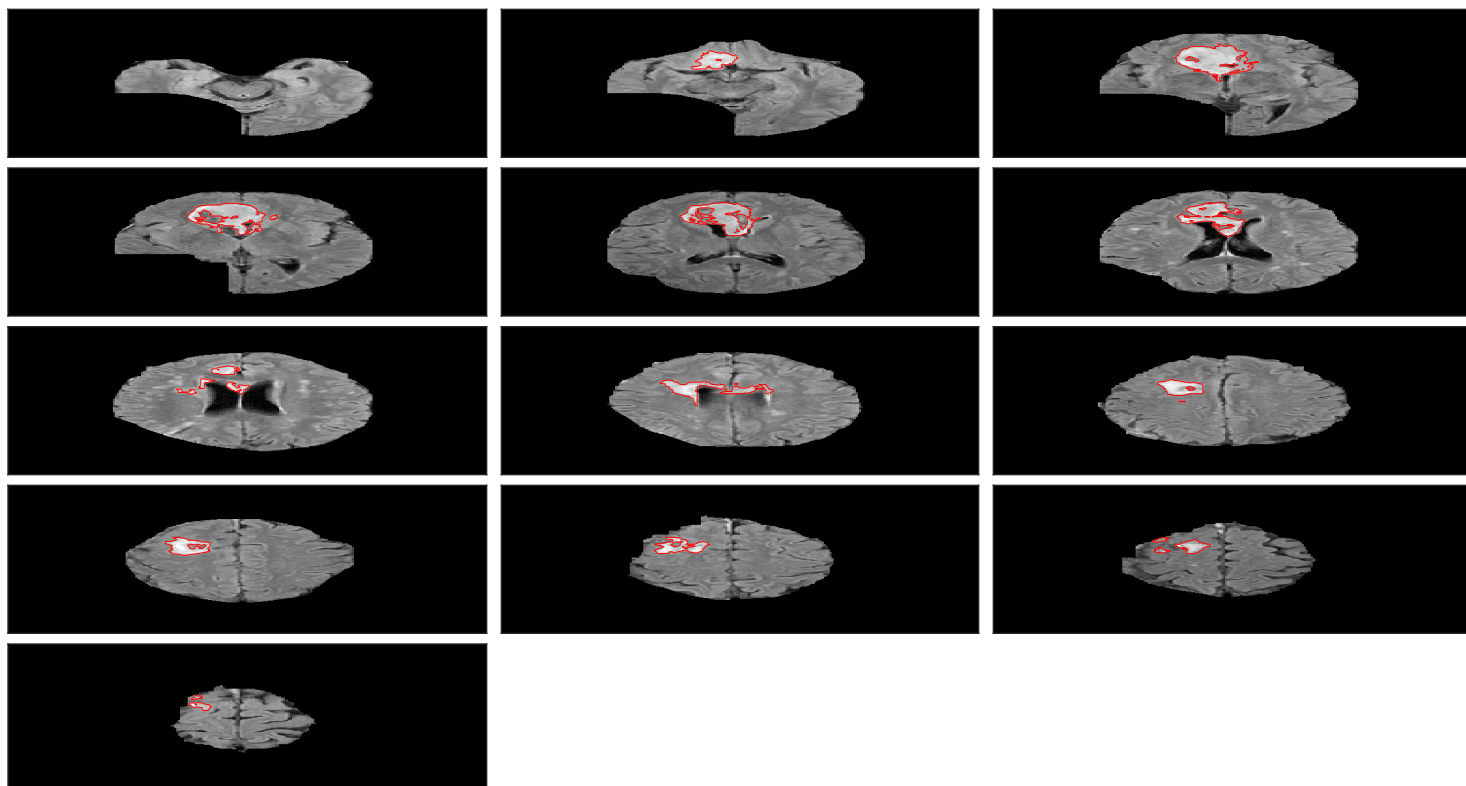

Supplement: S1 Data — The .zip file includes .pdf files labeled as follows, using case number 4224 as an example: 4224_t0.pdf includes the MRI data with segmentation of case number 4224 at baseline. 4224_t1_CAD_G_VC_S.pdf includes the MRI data with segmentation of case number 4224 at time 1, i.e., the time point of growth detection by the change-of-point method when the time point was different from the most recent MRI. 4224_tend_CAD_G_VC_G.pdf includes the MRI data with segmentation of case number 4224 at the most recent MRI. CAD_G and CAD_S mean that the change-of-point method predicted growth (G) or stability (S), respectively. VC_G and VC_S mean that the visual comparison detected growth (G) or stability (S), respectively. (ZIP) [file pmed.1002810.s001.zip › 7711_tend_CAD_G_VC_G.pdf]

Case Number:7713; LAST MRI; CAD --> GROWTH; VC --> STABLE; Interval From Baseline =21 months.

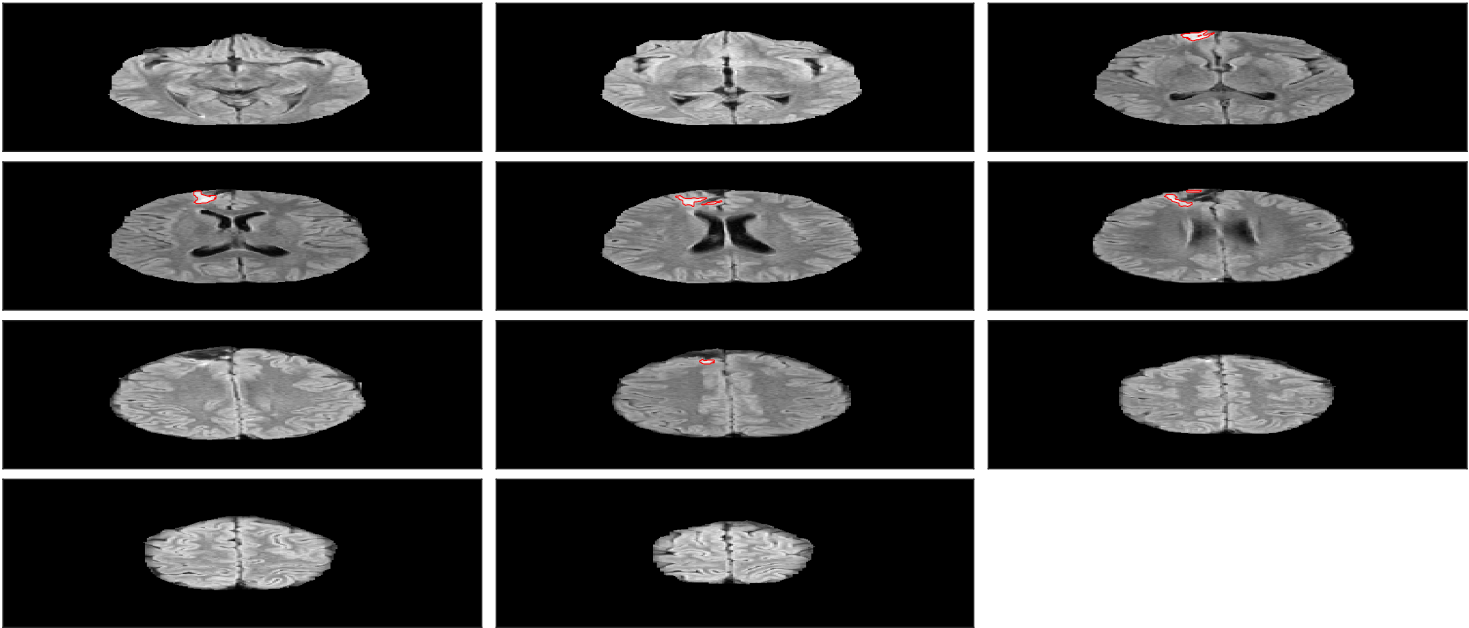

Supplement: S1 Data — The .zip file includes .pdf files labeled as follows, using case number 4224 as an example: 4224_t0.pdf includes the MRI data with segmentation of case number 4224 at baseline. 4224_t1_CAD_G_VC_S.pdf includes the MRI data with segmentation of case number 4224 at time 1, i.e., the time point of growth detection by the change-of-point method when the time point was different from the most recent MRI. 4224_tend_CAD_G_VC_G.pdf includes the MRI data with segmentation of case number 4224 at the most recent MRI. CAD_G and CAD_S mean that the change-of-point method predicted growth (G) or stability (S), respectively. VC_G and VC_S mean that the visual comparison detected growth (G) or stability (S), respectively. (ZIP) [file pmed.1002810.s001.zip › 7713_tend_CAD_G_VC_S.pdf]

Case Number:7491; LAST MRI; CAD --> GROWTH; VC --> GROWTH; Interval From Baseline =46 months.

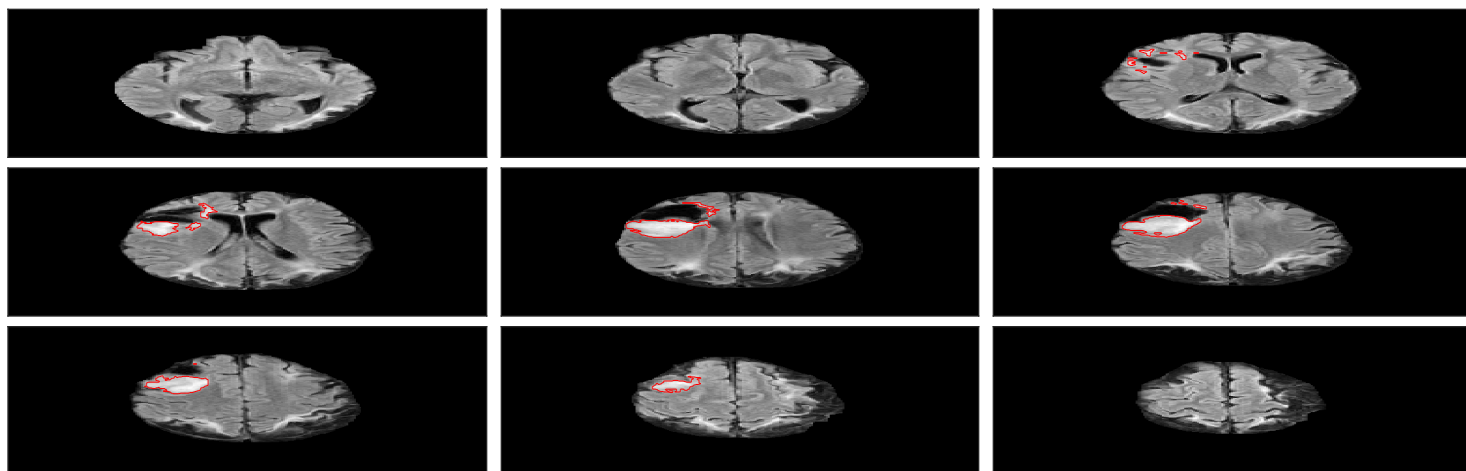

Supplement: S1 Data — The .zip file includes .pdf files labeled as follows, using case number 4224 as an example: 4224_t0.pdf includes the MRI data with segmentation of case number 4224 at baseline. 4224_t1_CAD_G_VC_S.pdf includes the MRI data with segmentation of case number 4224 at time 1, i.e., the time point of growth detection by the change-of-point method when the time point was different from the most recent MRI. 4224_tend_CAD_G_VC_G.pdf includes the MRI data with segmentation of case number 4224 at the most recent MRI. CAD_G and CAD_S mean that the change-of-point method predicted growth (G) or stability (S), respectively. VC_G and VC_S mean that the visual comparison detected growth (G) or stability (S), respectively. (ZIP) [file pmed.1002810.s001.zip › 7491_tend_CAD_G_VC_G.pdf]

Case Number:7504; TIME 1; CAD --> GROWTH; VC --> STABLE; Interval From Baseline =20 months.

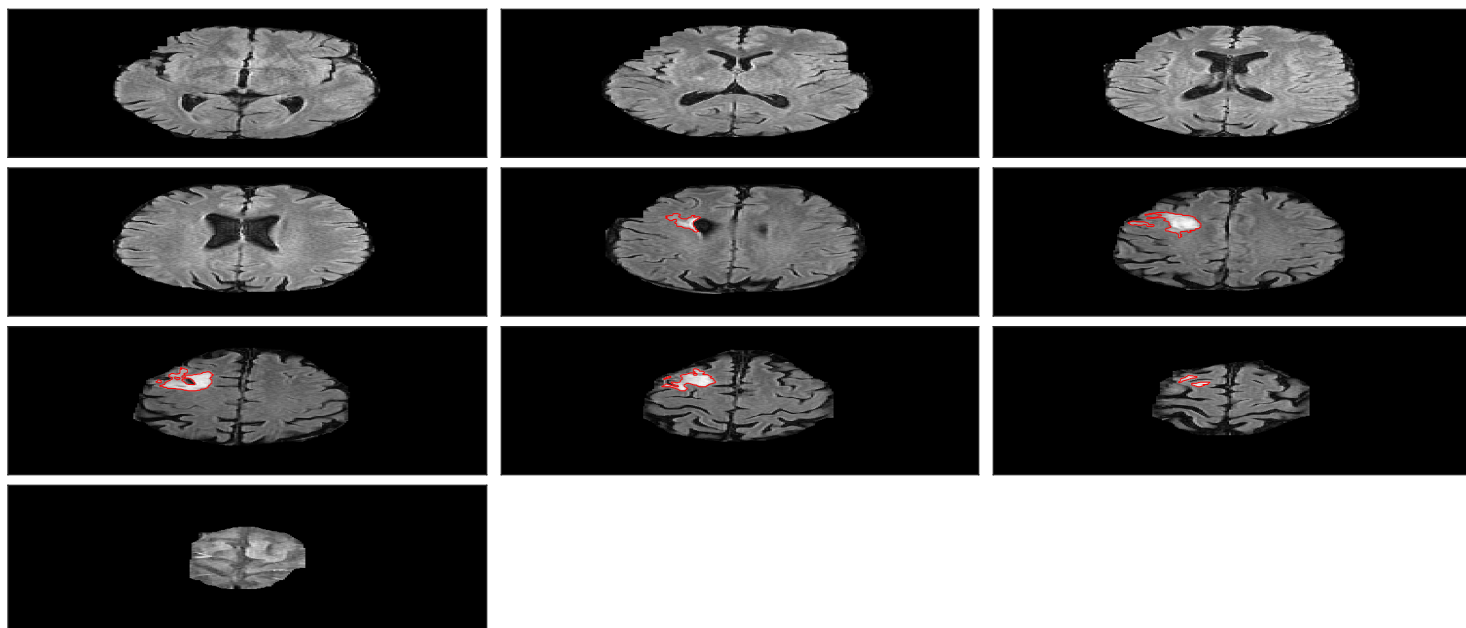

Supplement: S1 Data — The .zip file includes .pdf files labeled as follows, using case number 4224 as an example: 4224_t0.pdf includes the MRI data with segmentation of case number 4224 at baseline. 4224_t1_CAD_G_VC_S.pdf includes the MRI data with segmentation of case number 4224 at time 1, i.e., the time point of growth detection by the change-of-point method when the time point was different from the most recent MRI. 4224_tend_CAD_G_VC_G.pdf includes the MRI data with segmentation of case number 4224 at the most recent MRI. CAD_G and CAD_S mean that the change-of-point method predicted growth (G) or stability (S), respectively. VC_G and VC_S mean that the visual comparison detected growth (G) or stability (S), respectively. (ZIP) [file pmed.1002810.s001.zip › 7504_t1_CAD_G_VC_S.pdf]

Case Number:7493; Time 0 (Baseline).

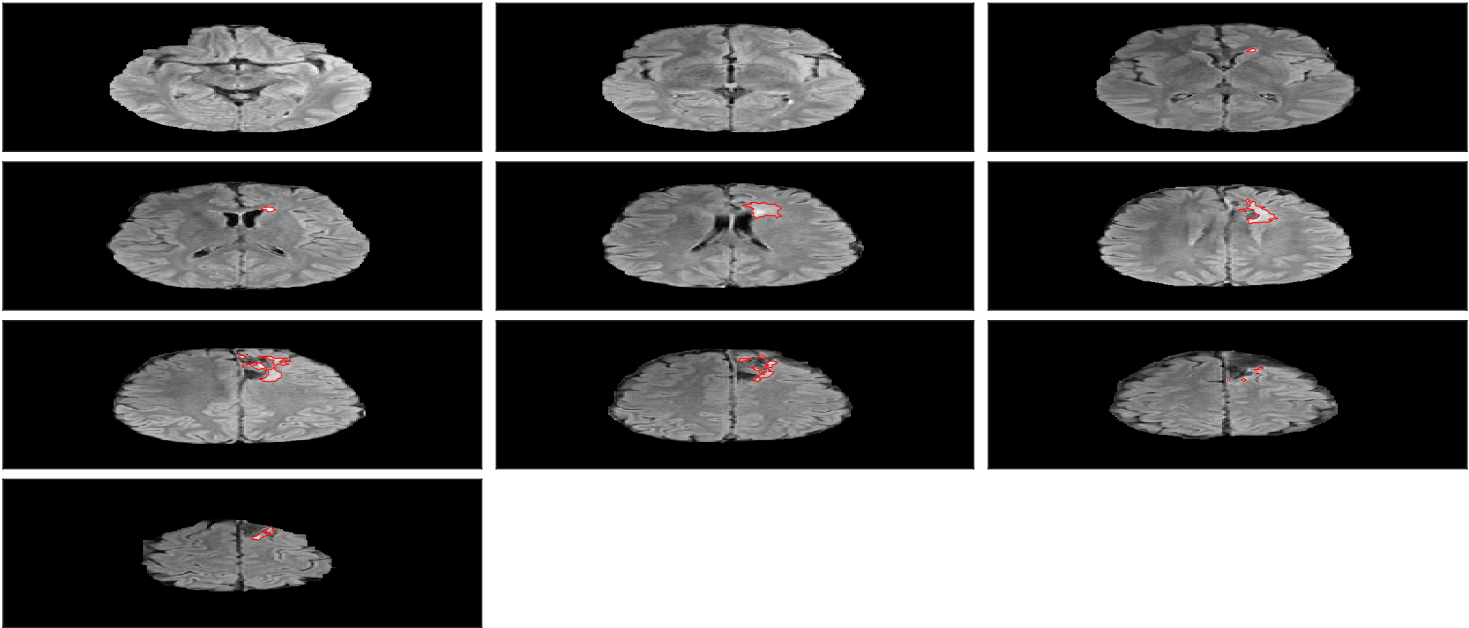

Supplement: S1 Data — The .zip file includes .pdf files labeled as follows, using case number 4224 as an example: 4224_t0.pdf includes the MRI data with segmentation of case number 4224 at baseline. 4224_t1_CAD_G_VC_S.pdf includes the MRI data with segmentation of case number 4224 at time 1, i.e., the time point of growth detection by the change-of-point method when the time point was different from the most recent MRI. 4224_tend_CAD_G_VC_G.pdf includes the MRI data with segmentation of case number 4224 at the most recent MRI. CAD_G and CAD_S mean that the change-of-point method predicted growth (G) or stability (S), respectively. VC_G and VC_S mean that the visual comparison detected growth (G) or stability (S), respectively. (ZIP) [file pmed.1002810.s001.zip › 7493_t0.pdf]

Case Number:7489; LAST MRI; CAD --> GROWTH; VC --> GROWTH; Interval From Baseline =51 months.

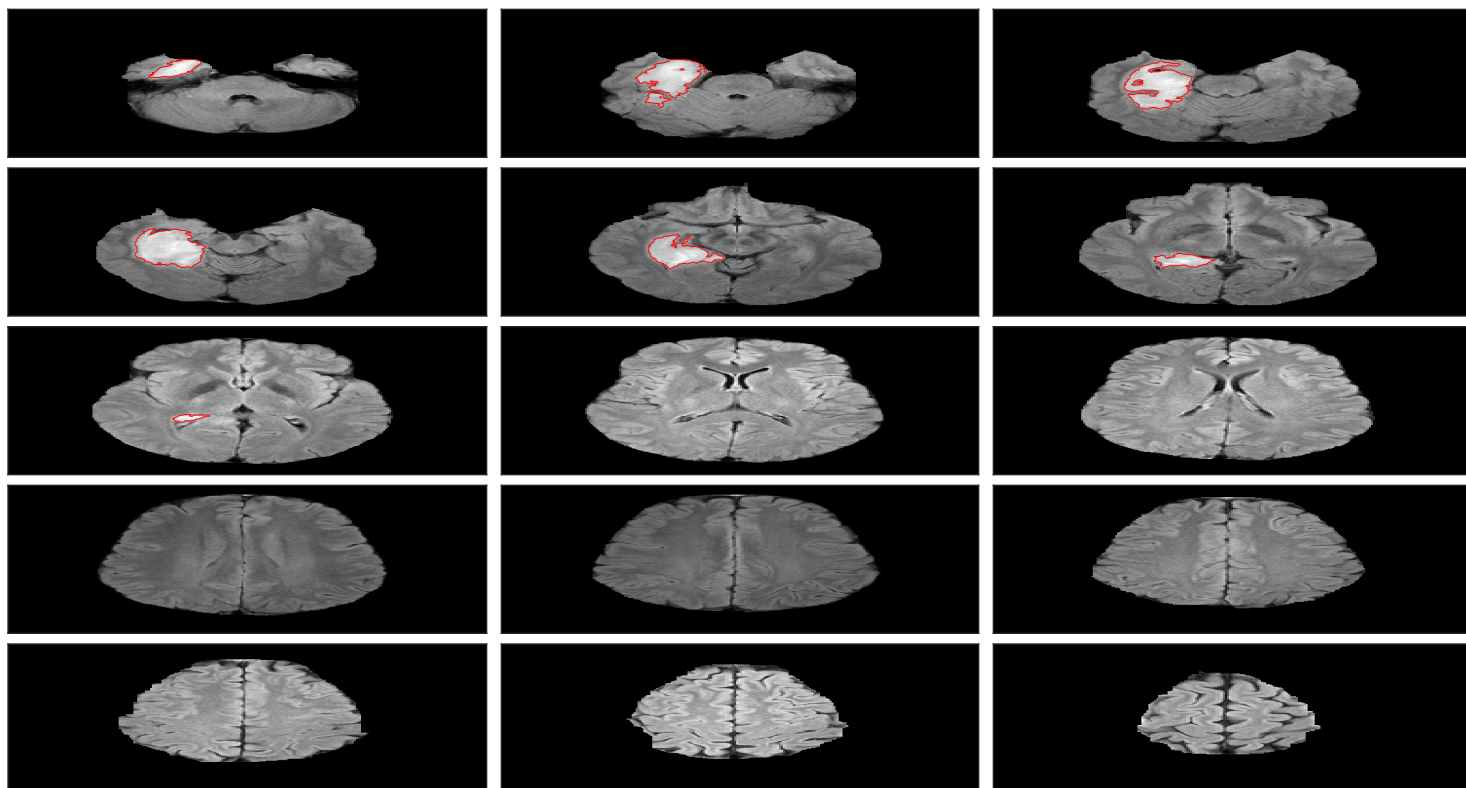

Supplement: S1 Data — The .zip file includes .pdf files labeled as follows, using case number 4224 as an example: 4224_t0.pdf includes the MRI data with segmentation of case number 4224 at baseline. 4224_t1_CAD_G_VC_S.pdf includes the MRI data with segmentation of case number 4224 at time 1, i.e., the time point of growth detection by the change-of-point method when the time point was different from the most recent MRI. 4224_tend_CAD_G_VC_G.pdf includes the MRI data with segmentation of case number 4224 at the most recent MRI. CAD_G and CAD_S mean that the change-of-point method predicted growth (G) or stability (S), respectively. VC_G and VC_S mean that the visual comparison detected growth (G) or stability (S), respectively. (ZIP) [file pmed.1002810.s001.zip › 7489_tend_CAD_G_VC_G.pdf]

Case Number:7492; LAST MRI; CAD --> GROWTH; VC --> STABLE; Interval From Baseline =16 months.

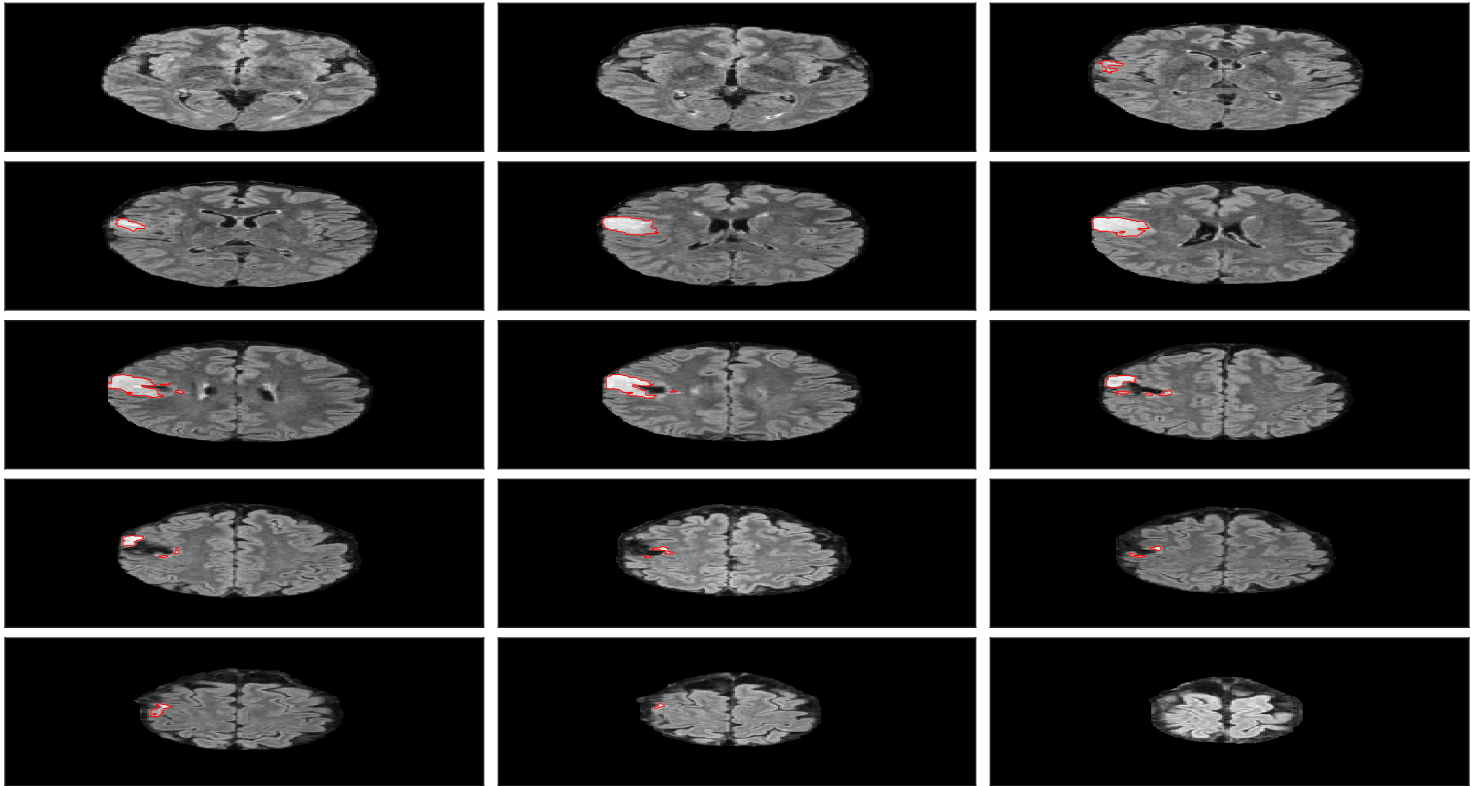

Supplement: S1 Data — The .zip file includes .pdf files labeled as follows, using case number 4224 as an example: 4224_t0.pdf includes the MRI data with segmentation of case number 4224 at baseline. 4224_t1_CAD_G_VC_S.pdf includes the MRI data with segmentation of case number 4224 at time 1, i.e., the time point of growth detection by the change-of-point method when the time point was different from the most recent MRI. 4224_tend_CAD_G_VC_G.pdf includes the MRI data with segmentation of case number 4224 at the most recent MRI. CAD_G and CAD_S mean that the change-of-point method predicted growth (G) or stability (S), respectively. VC_G and VC_S mean that the visual comparison detected growth (G) or stability (S), respectively. (ZIP) [file pmed.1002810.s001.zip › 7492_tend_CAD_G_VC_S.pdf]

Case Number:7736; Time 0 (Baseline).

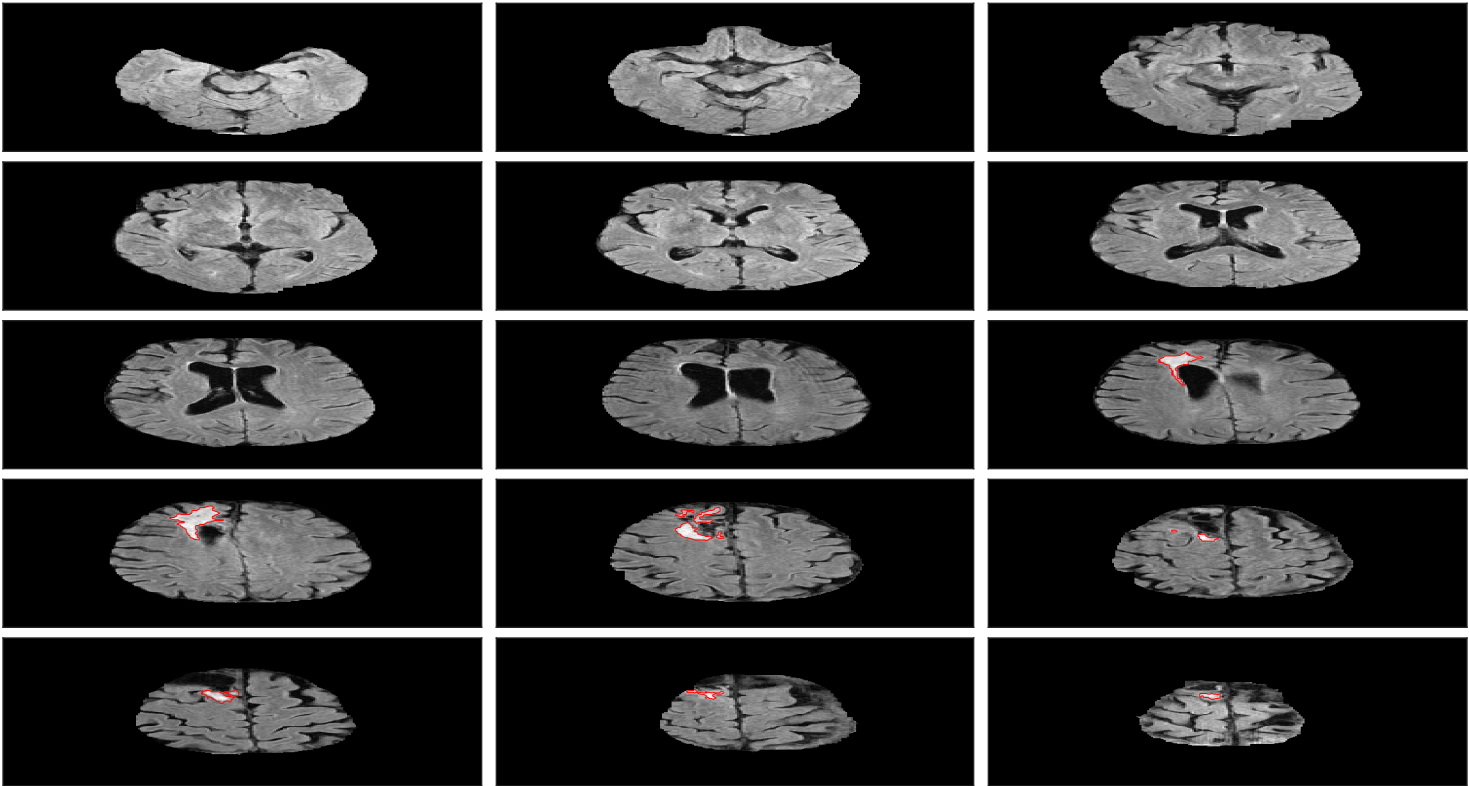

Supplement: S1 Data — The .zip file includes .pdf files labeled as follows, using case number 4224 as an example: 4224_t0.pdf includes the MRI data with segmentation of case number 4224 at baseline. 4224_t1_CAD_G_VC_S.pdf includes the MRI data with segmentation of case number 4224 at time 1, i.e., the time point of growth detection by the change-of-point method when the time point was different from the most recent MRI. 4224_tend_CAD_G_VC_G.pdf includes the MRI data with segmentation of case number 4224 at the most recent MRI. CAD_G and CAD_S mean that the change-of-point method predicted growth (G) or stability (S), respectively. VC_G and VC_S mean that the visual comparison detected growth (G) or stability (S), respectively. (ZIP) [file pmed.1002810.s001.zip › 7736_t0.pdf]

Case Number:7490; LAST MRI; CAD --> GROWTH; VC --> GROWTH; Interval From Baseline =154 months.

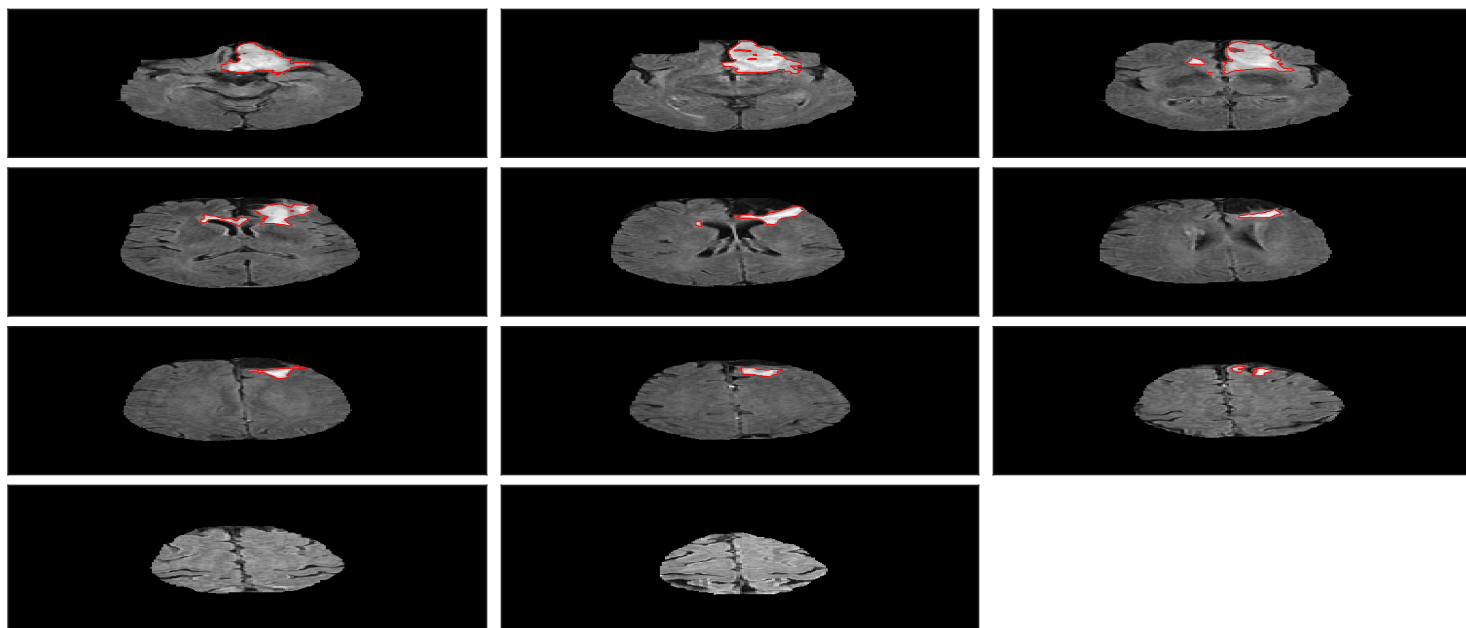

Supplement: S1 Data — The .zip file includes .pdf files labeled as follows, using case number 4224 as an example: 4224_t0.pdf includes the MRI data with segmentation of case number 4224 at baseline. 4224_t1_CAD_G_VC_S.pdf includes the MRI data with segmentation of case number 4224 at time 1, i.e., the time point of growth detection by the change-of-point method when the time point was different from the most recent MRI. 4224_tend_CAD_G_VC_G.pdf includes the MRI data with segmentation of case number 4224 at the most recent MRI. CAD_G and CAD_S mean that the change-of-point method predicted growth (G) or stability (S), respectively. VC_G and VC_S mean that the visual comparison detected growth (G) or stability (S), respectively. (ZIP) [file pmed.1002810.s001.zip › 7490_tend_CAD_G_VC_G.pdf]

Case Number:7755; Time 0 (Baseline).

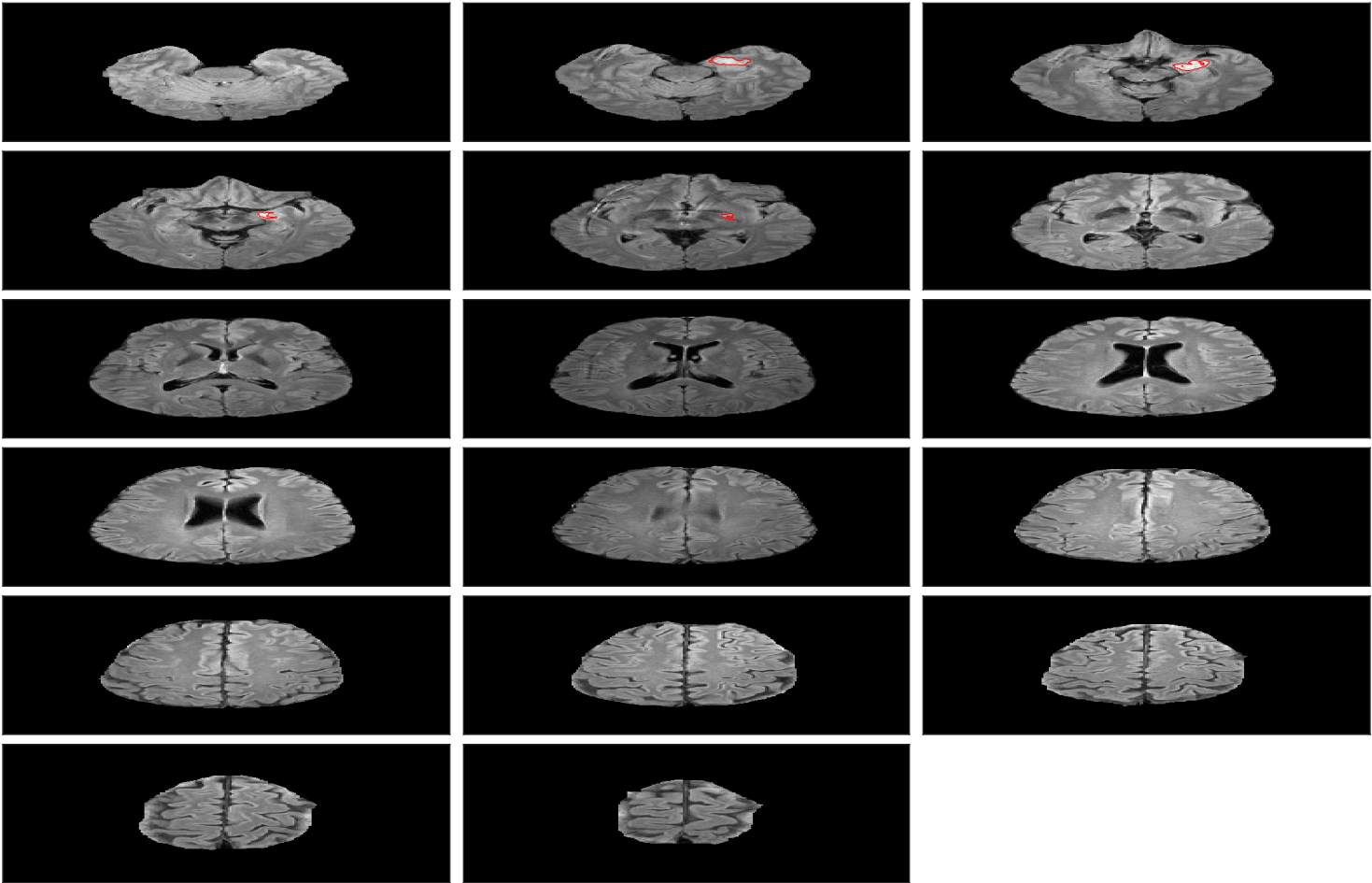

Supplement: S1 Data — The .zip file includes .pdf files labeled as follows, using case number 4224 as an example: 4224_t0.pdf includes the MRI data with segmentation of case number 4224 at baseline. 4224_t1_CAD_G_VC_S.pdf includes the MRI data with segmentation of case number 4224 at time 1, i.e., the time point of growth detection by the change-of-point method when the time point was different from the most recent MRI. 4224_tend_CAD_G_VC_G.pdf includes the MRI data with segmentation of case number 4224 at the most recent MRI. CAD_G and CAD_S mean that the change-of-point method predicted growth (G) or stability (S), respectively. VC_G and VC_S mean that the visual comparison detected growth (G) or stability (S), respectively. (ZIP) [file pmed.1002810.s001.zip › 7755_t0.pdf]

Case Number:7709; LAST MRI; CAD --> GROWTH; VC --> GROWTH; Interval From Baseline =38 months.

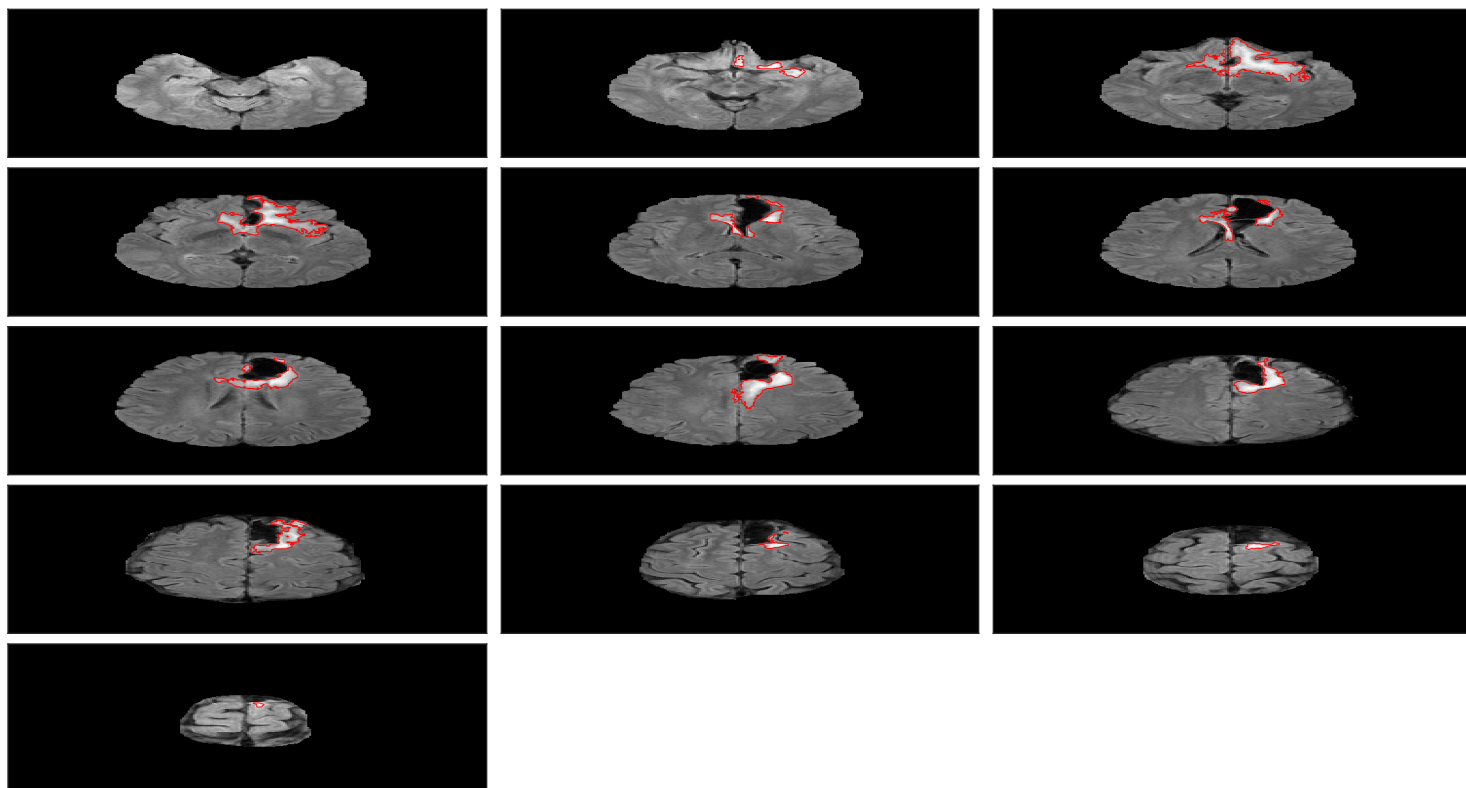

Supplement: S1 Data — The .zip file includes .pdf files labeled as follows, using case number 4224 as an example: 4224_t0.pdf includes the MRI data with segmentation of case number 4224 at baseline. 4224_t1_CAD_G_VC_S.pdf includes the MRI data with segmentation of case number 4224 at time 1, i.e., the time point of growth detection by the change-of-point method when the time point was different from the most recent MRI. 4224_tend_CAD_G_VC_G.pdf includes the MRI data with segmentation of case number 4224 at the most recent MRI. CAD_G and CAD_S mean that the change-of-point method predicted growth (G) or stability (S), respectively. VC_G and VC_S mean that the visual comparison detected growth (G) or stability (S), respectively. (ZIP) [file pmed.1002810.s001.zip › 7709_tend_CAD_G_VC_G.pdf]

Case Number:7718; TIME 1; CAD --> GROWTH; VC --> STABLE; Interval From Baseline =7 months.

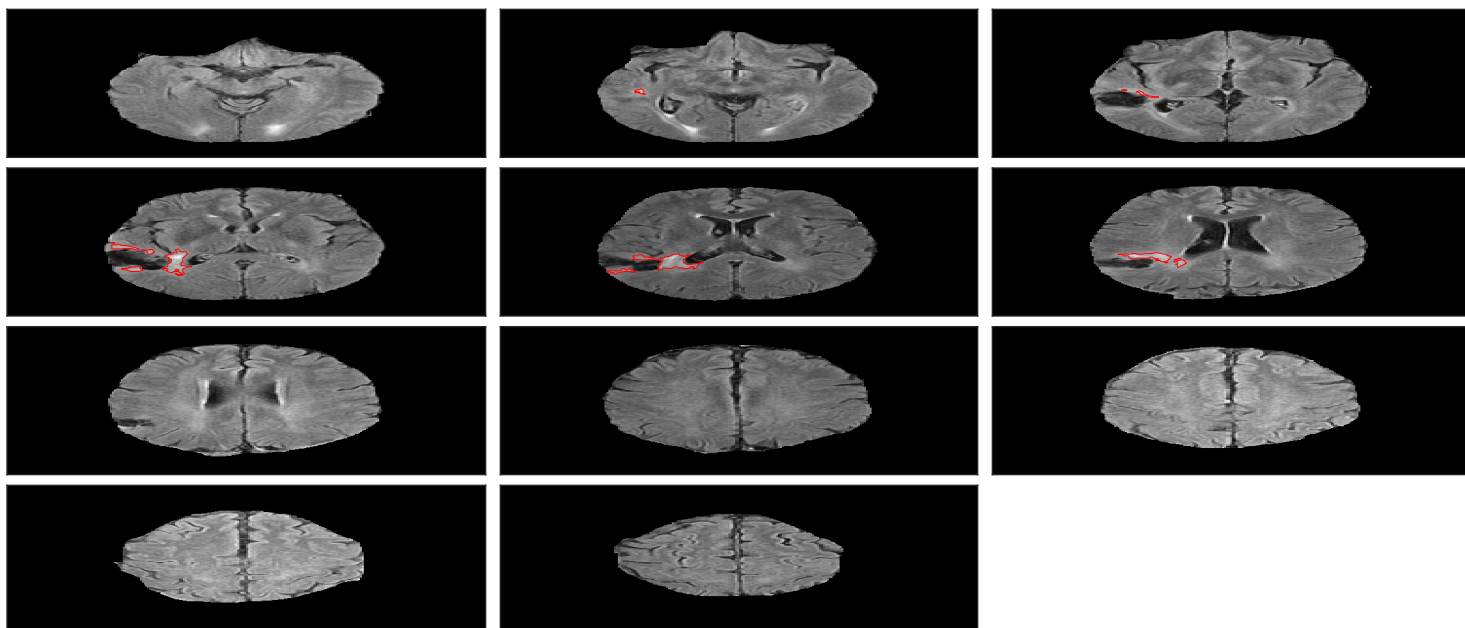

Supplement: S1 Data — The .zip file includes .pdf files labeled as follows, using case number 4224 as an example: 4224_t0.pdf includes the MRI data with segmentation of case number 4224 at baseline. 4224_t1_CAD_G_VC_S.pdf includes the MRI data with segmentation of case number 4224 at time 1, i.e., the time point of growth detection by the change-of-point method when the time point was different from the most recent MRI. 4224_tend_CAD_G_VC_G.pdf includes the MRI data with segmentation of case number 4224 at the most recent MRI. CAD_G and CAD_S mean that the change-of-point method predicted growth (G) or stability (S), respectively. VC_G and VC_S mean that the visual comparison detected growth (G) or stability (S), respectively. (ZIP) [file pmed.1002810.s001.zip › 7718_t1_CAD_G_VC_S.pdf]

Case Number:7715; LAST MRI; CAD --> STABLE; VC --> STABLE; Interval From Baseline =7 months.

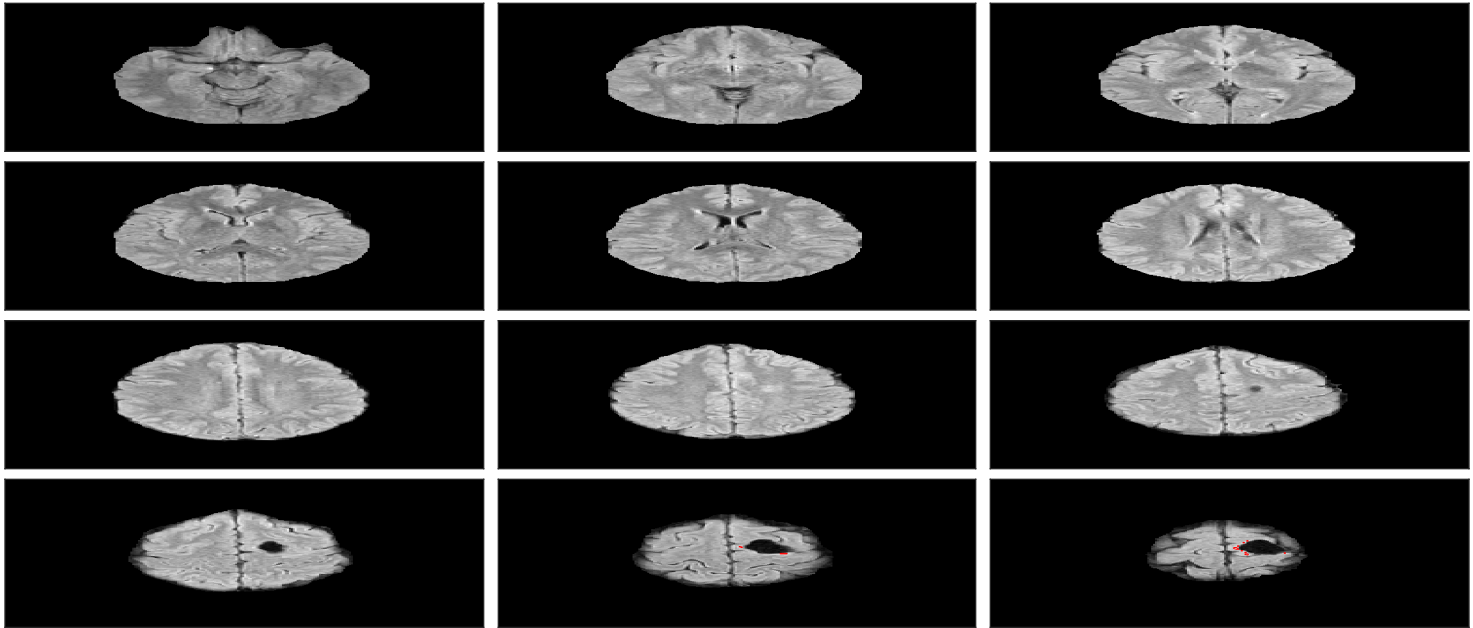

Supplement: S1 Data — The .zip file includes .pdf files labeled as follows, using case number 4224 as an example: 4224_t0.pdf includes the MRI data with segmentation of case number 4224 at baseline. 4224_t1_CAD_G_VC_S.pdf includes the MRI data with segmentation of case number 4224 at time 1, i.e., the time point of growth detection by the change-of-point method when the time point was different from the most recent MRI. 4224_tend_CAD_G_VC_G.pdf includes the MRI data with segmentation of case number 4224 at the most recent MRI. CAD_G and CAD_S mean that the change-of-point method predicted growth (G) or stability (S), respectively. VC_G and VC_S mean that the visual comparison detected growth (G) or stability (S), respectively. (ZIP) [file pmed.1002810.s001.zip › 7715_tend_CAD_S_VC_S.pdf]

Case Number:4224; TIME 1; CAD --> GROWTH; VC --> STABLE; Interval From Baseline =21 months.

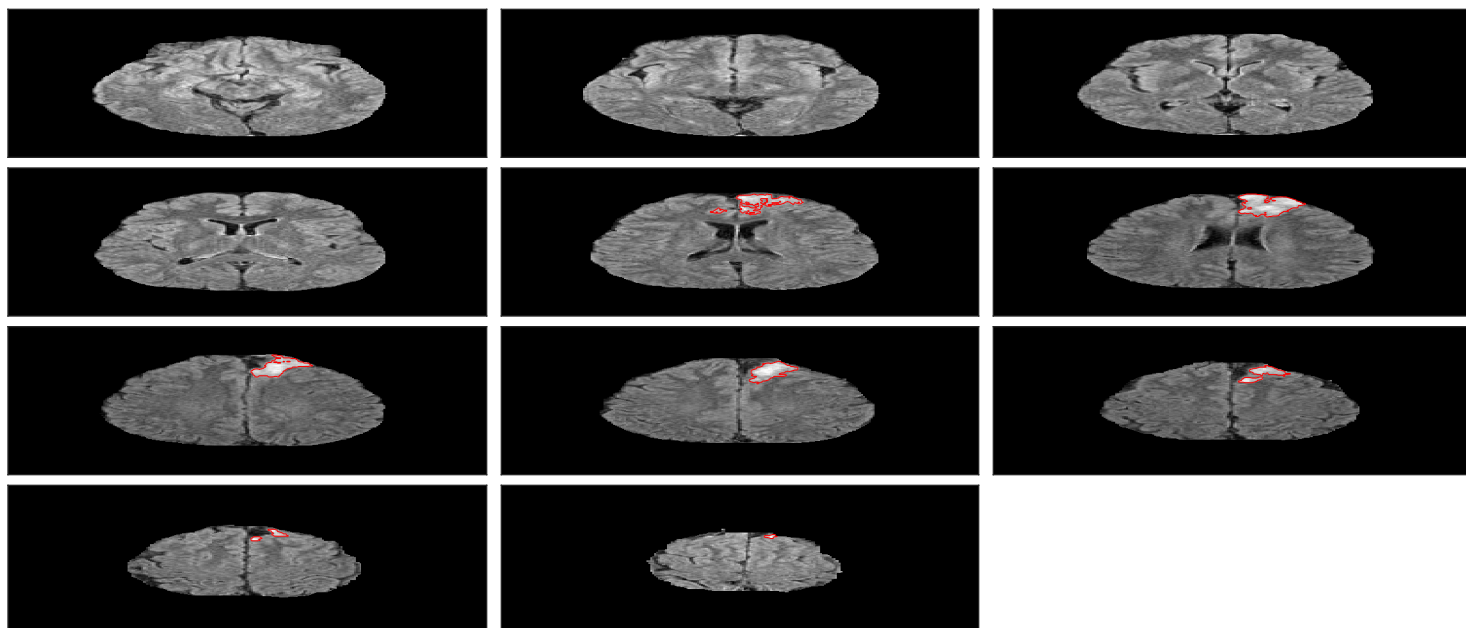

Supplement: S1 Data — The .zip file includes .pdf files labeled as follows, using case number 4224 as an example: 4224_t0.pdf includes the MRI data with segmentation of case number 4224 at baseline. 4224_t1_CAD_G_VC_S.pdf includes the MRI data with segmentation of case number 4224 at time 1, i.e., the time point of growth detection by the change-of-point method when the time point was different from the most recent MRI. 4224_tend_CAD_G_VC_G.pdf includes the MRI data with segmentation of case number 4224 at the most recent MRI. CAD_G and CAD_S mean that the change-of-point method predicted growth (G) or stability (S), respectively. VC_G and VC_S mean that the visual comparison detected growth (G) or stability (S), respectively. (ZIP) [file pmed.1002810.s001.zip › 4224_t1_CAD_G_VC_S.pdf]

Case Number:7069; LAST MRI; CAD --> GROWTH; VC --> GROWTH; Interval From Baseline =25 months.

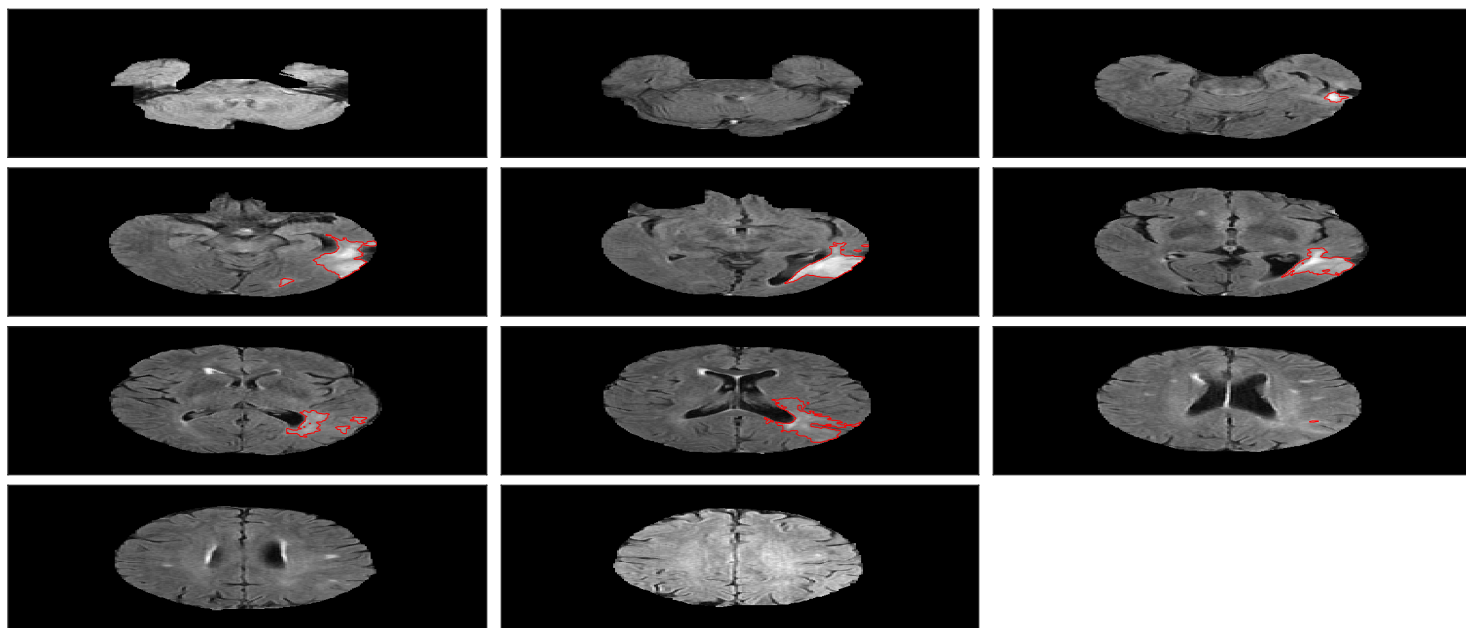

Supplement: S1 Data — The .zip file includes .pdf files labeled as follows, using case number 4224 as an example: 4224_t0.pdf includes the MRI data with segmentation of case number 4224 at baseline. 4224_t1_CAD_G_VC_S.pdf includes the MRI data with segmentation of case number 4224 at time 1, i.e., the time point of growth detection by the change-of-point method when the time point was different from the most recent MRI. 4224_tend_CAD_G_VC_G.pdf includes the MRI data with segmentation of case number 4224 at the most recent MRI. CAD_G and CAD_S mean that the change-of-point method predicted growth (G) or stability (S), respectively. VC_G and VC_S mean that the visual comparison detected growth (G) or stability (S), respectively. (ZIP) [file pmed.1002810.s001.zip › 7069_tend_CAD_G_VC_G.pdf]

Case Number:6934; TIME 1; CAD --> GROWTH; VC --> STABLE; Interval From Baseline =18 months.

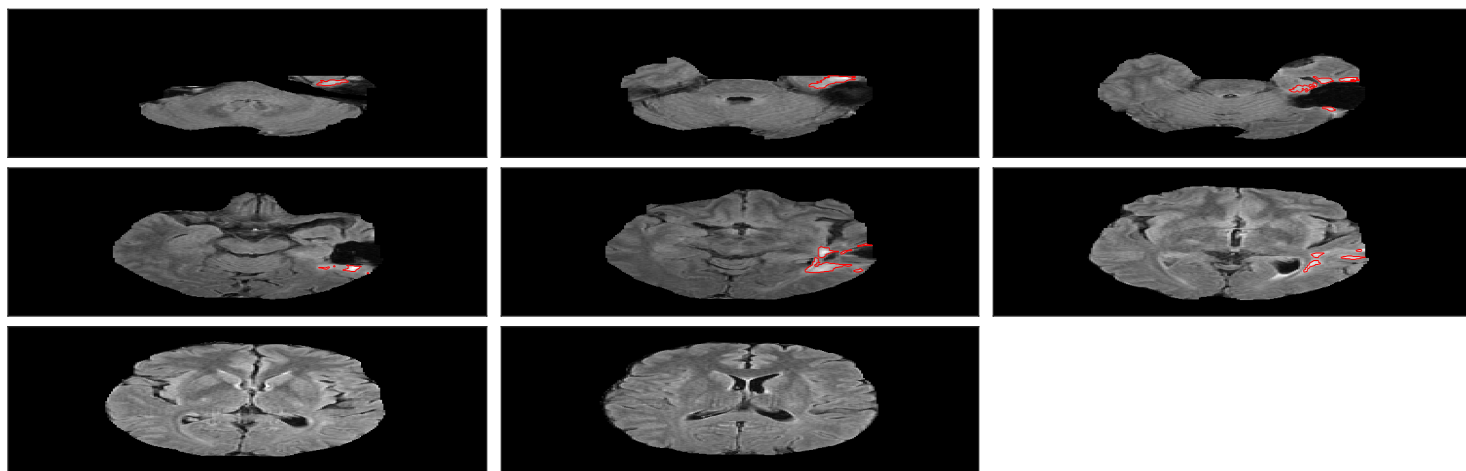

Supplement: S1 Data — The .zip file includes .pdf files labeled as follows, using case number 4224 as an example: 4224_t0.pdf includes the MRI data with segmentation of case number 4224 at baseline. 4224_t1_CAD_G_VC_S.pdf includes the MRI data with segmentation of case number 4224 at time 1, i.e., the time point of growth detection by the change-of-point method when the time point was different from the most recent MRI. 4224_tend_CAD_G_VC_G.pdf includes the MRI data with segmentation of case number 4224 at the most recent MRI. CAD_G and CAD_S mean that the change-of-point method predicted growth (G) or stability (S), respectively. VC_G and VC_S mean that the visual comparison detected growth (G) or stability (S), respectively. (ZIP) [file pmed.1002810.s001.zip › 6934_t1_CAD_G_VC_S.pdf]

Case Number:7070; LAST MRI; CAD --> GROWTH; VC --> GROWTH; Interval From Baseline =108 months.

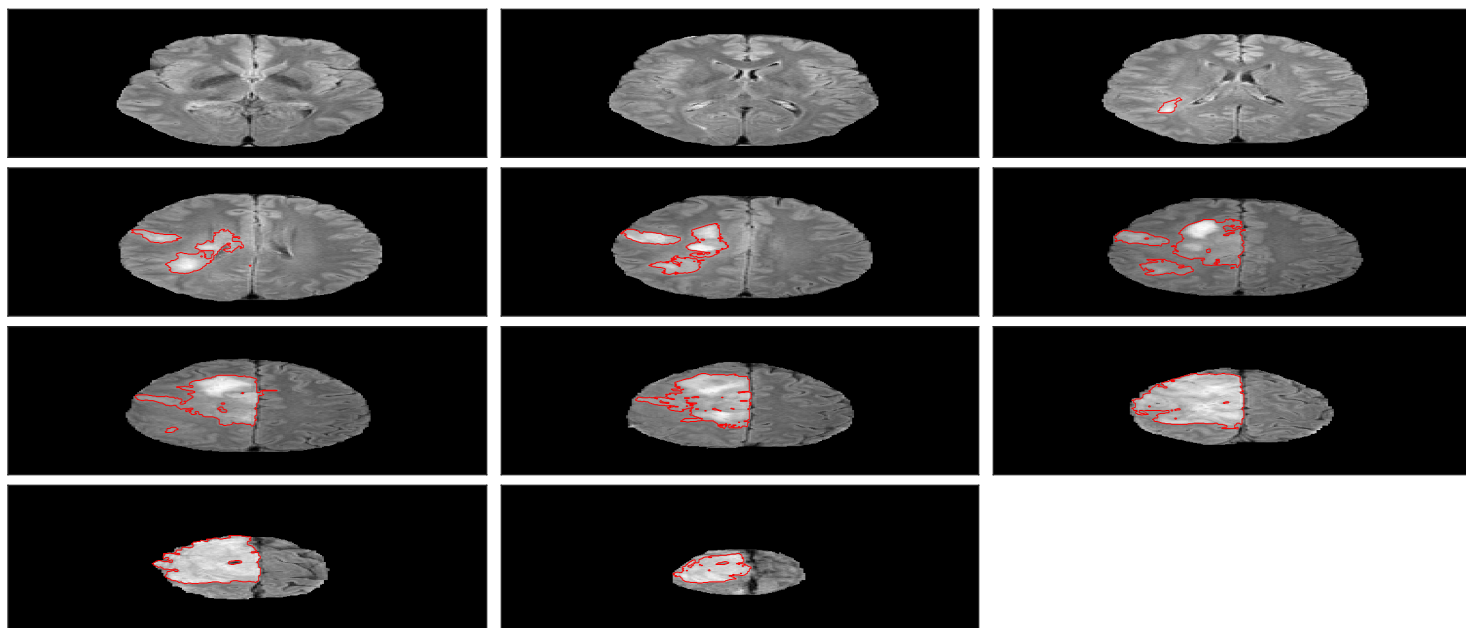

Supplement: S1 Data — The .zip file includes .pdf files labeled as follows, using case number 4224 as an example: 4224_t0.pdf includes the MRI data with segmentation of case number 4224 at baseline. 4224_t1_CAD_G_VC_S.pdf includes the MRI data with segmentation of case number 4224 at time 1, i.e., the time point of growth detection by the change-of-point method when the time point was different from the most recent MRI. 4224_tend_CAD_G_VC_G.pdf includes the MRI data with segmentation of case number 4224 at the most recent MRI. CAD_G and CAD_S mean that the change-of-point method predicted growth (G) or stability (S), respectively. VC_G and VC_S mean that the visual comparison detected growth (G) or stability (S), respectively. (ZIP) [file pmed.1002810.s001.zip › 7070_tend_CAD_G_VC_G.pdf]

Case Number:7708; LAST MRI; CAD --> GROWTH; VC --> STABLE; Interval From Baseline =25 months.

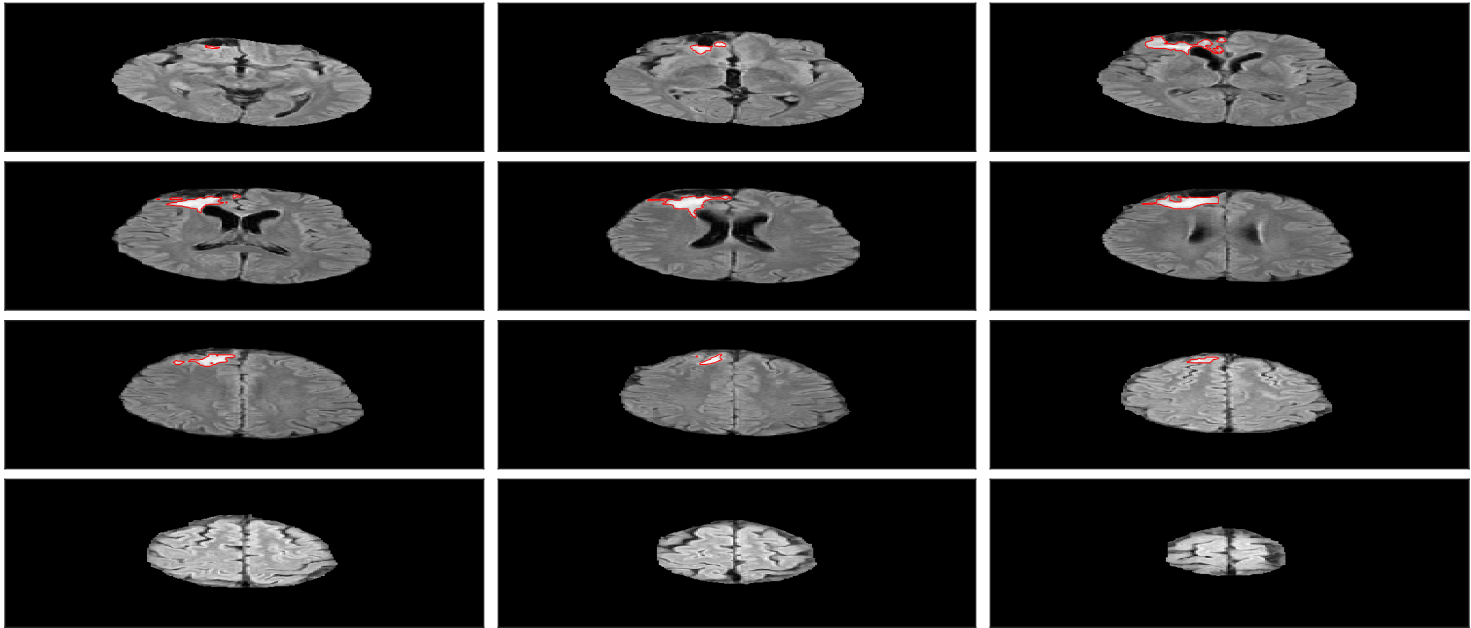

Supplement: S1 Data — The .zip file includes .pdf files labeled as follows, using case number 4224 as an example: 4224_t0.pdf includes the MRI data with segmentation of case number 4224 at baseline. 4224_t1_CAD_G_VC_S.pdf includes the MRI data with segmentation of case number 4224 at time 1, i.e., the time point of growth detection by the change-of-point method when the time point was different from the most recent MRI. 4224_tend_CAD_G_VC_G.pdf includes the MRI data with segmentation of case number 4224 at the most recent MRI. CAD_G and CAD_S mean that the change-of-point method predicted growth (G) or stability (S), respectively. VC_G and VC_S mean that the visual comparison detected growth (G) or stability (S), respectively. (ZIP) [file pmed.1002810.s001.zip › 7708_tend_CAD_G_VC_S.pdf]

Case Number:6937; Time 0 (Baseline).

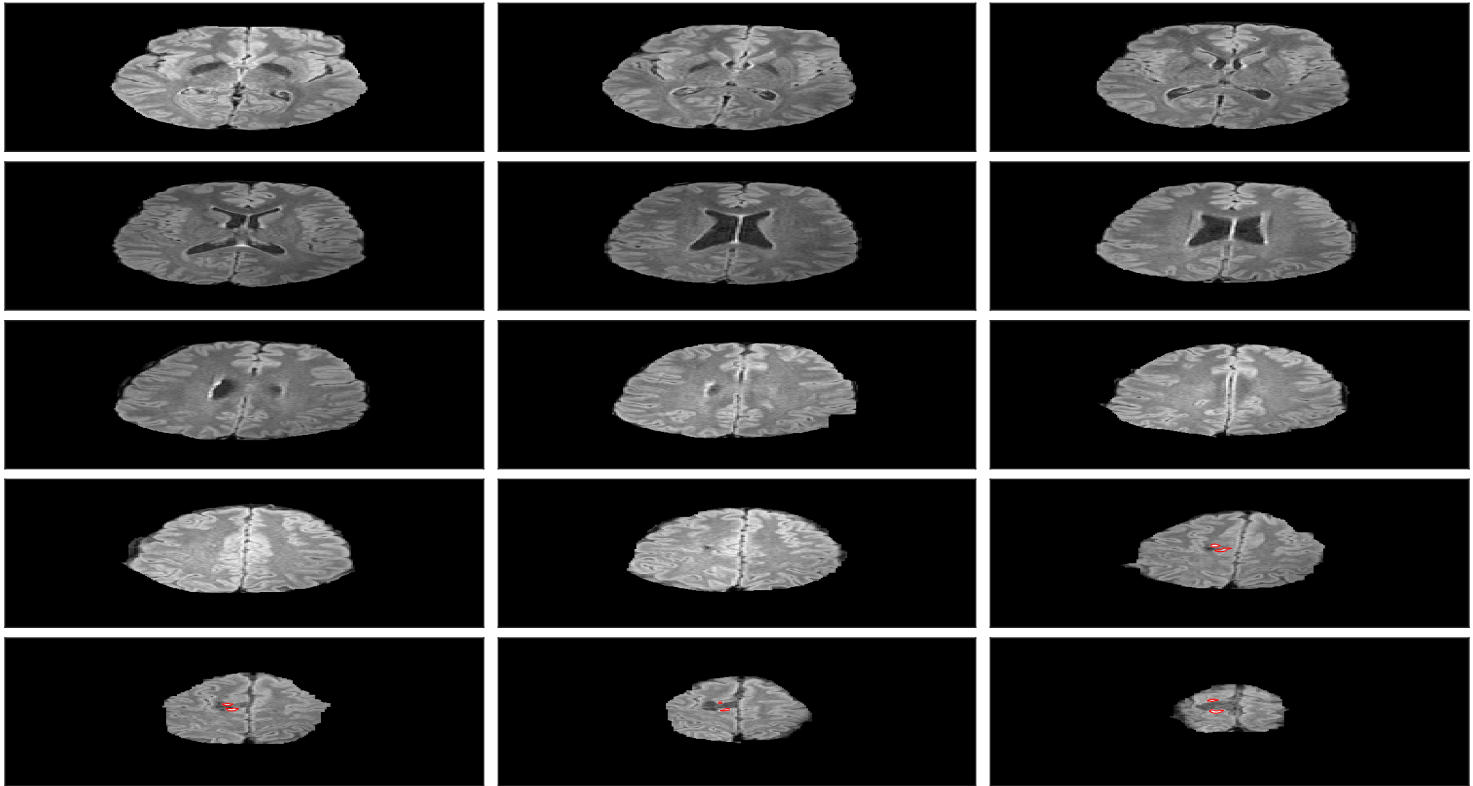

Supplement: S1 Data — The .zip file includes .pdf files labeled as follows, using case number 4224 as an example: 4224_t0.pdf includes the MRI data with segmentation of case number 4224 at baseline. 4224_t1_CAD_G_VC_S.pdf includes the MRI data with segmentation of case number 4224 at time 1, i.e., the time point of growth detection by the change-of-point method when the time point was different from the most recent MRI. 4224_tend_CAD_G_VC_G.pdf includes the MRI data with segmentation of case number 4224 at the most recent MRI. CAD_G and CAD_S mean that the change-of-point method predicted growth (G) or stability (S), respectively. VC_G and VC_S mean that the visual comparison detected growth (G) or stability (S), respectively. (ZIP) [file pmed.1002810.s001.zip › 6937_t0.pdf]

Case Number:4224; Time 0 (Baseline).

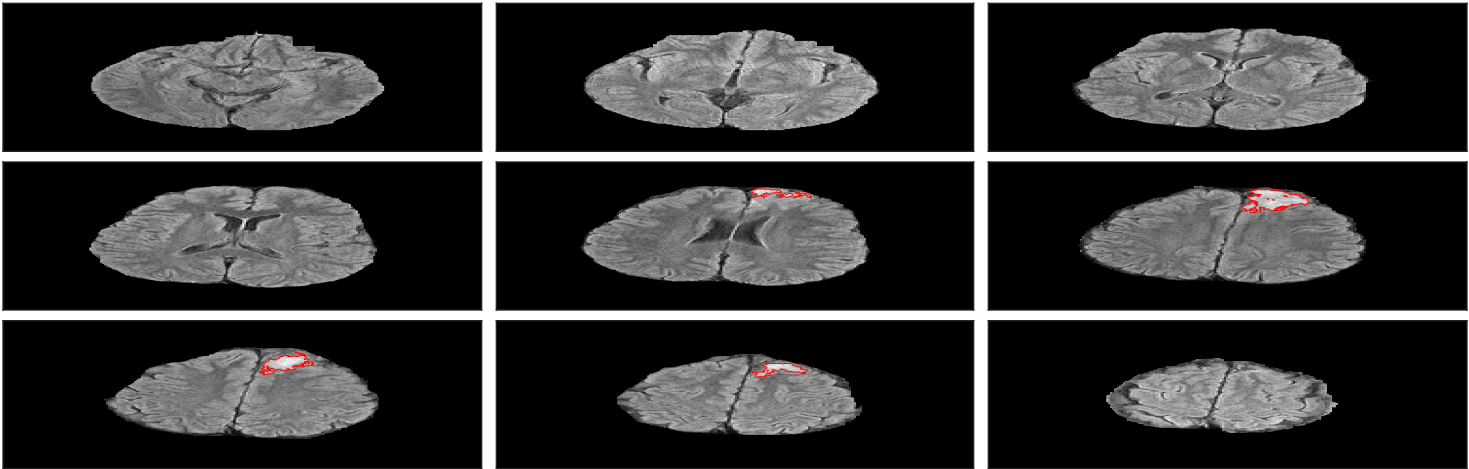

Supplement: S1 Data — The .zip file includes .pdf files labeled as follows, using case number 4224 as an example: 4224_t0.pdf includes the MRI data with segmentation of case number 4224 at baseline. 4224_t1_CAD_G_VC_S.pdf includes the MRI data with segmentation of case number 4224 at time 1, i.e., the time point of growth detection by the change-of-point method when the time point was different from the most recent MRI. 4224_tend_CAD_G_VC_G.pdf includes the MRI data with segmentation of case number 4224 at the most recent MRI. CAD_G and CAD_S mean that the change-of-point method predicted growth (G) or stability (S), respectively. VC_G and VC_S mean that the visual comparison detected growth (G) or stability (S), respectively. (ZIP) [file pmed.1002810.s001.zip › 4224_t0.pdf]

Case Number:7741; LAST MRI; CAD --> GROWTH; VC --> STABLE; Interval From Baseline =81 months.

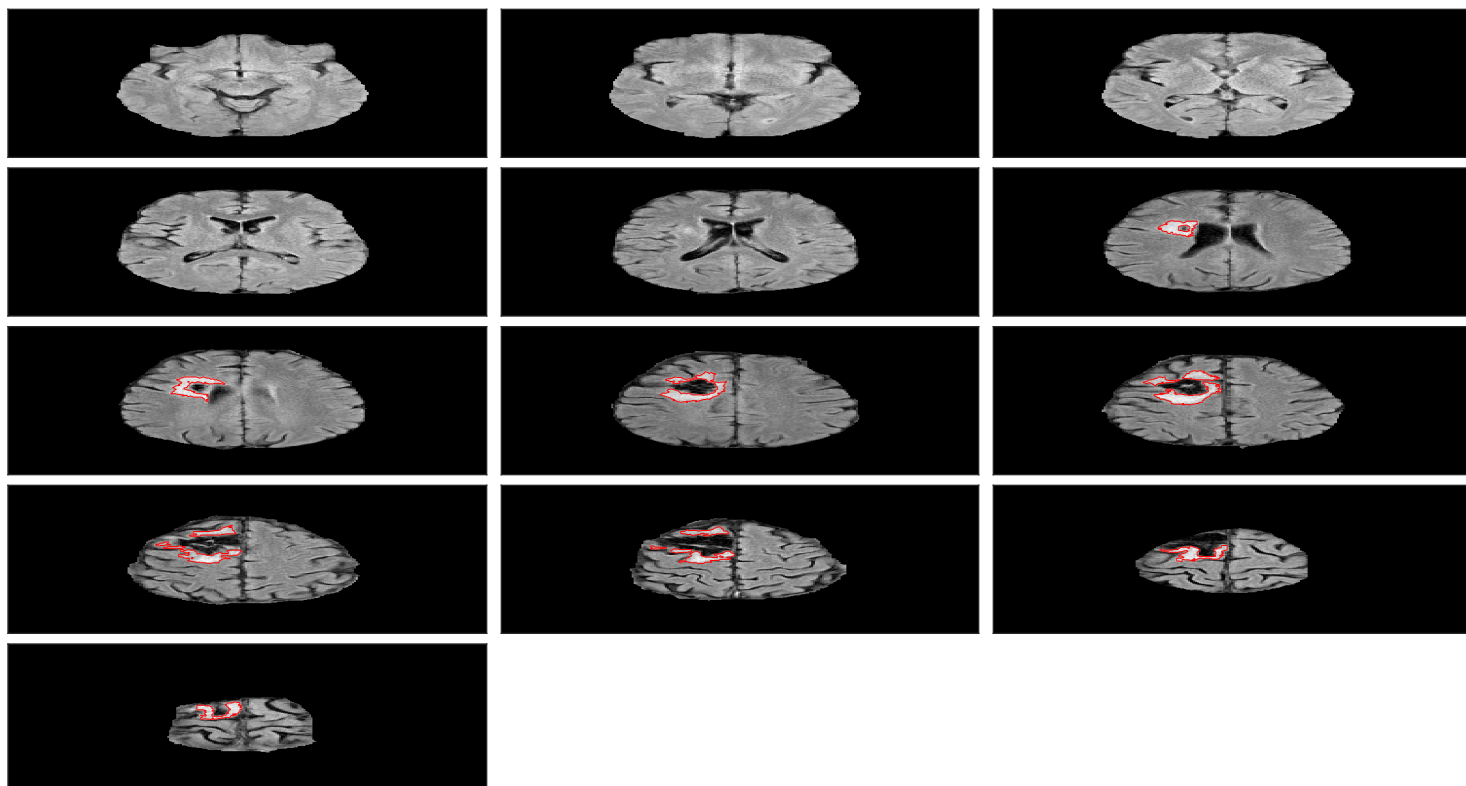

Supplement: S1 Data — The .zip file includes .pdf files labeled as follows, using case number 4224 as an example: 4224_t0.pdf includes the MRI data with segmentation of case number 4224 at baseline. 4224_t1_CAD_G_VC_S.pdf includes the MRI data with segmentation of case number 4224 at time 1, i.e., the time point of growth detection by the change-of-point method when the time point was different from the most recent MRI. 4224_tend_CAD_G_VC_G.pdf includes the MRI data with segmentation of case number 4224 at the most recent MRI. CAD_G and CAD_S mean that the change-of-point method predicted growth (G) or stability (S), respectively. VC_G and VC_S mean that the visual comparison detected growth (G) or stability (S), respectively. (ZIP) [file pmed.1002810.s001.zip › 7741_tend_CAD_G_VC_S.pdf]

Case Number:7714; LAST MRI; CAD --> STABLE; VC --> STABLE; Interval From Baseline =69 months.

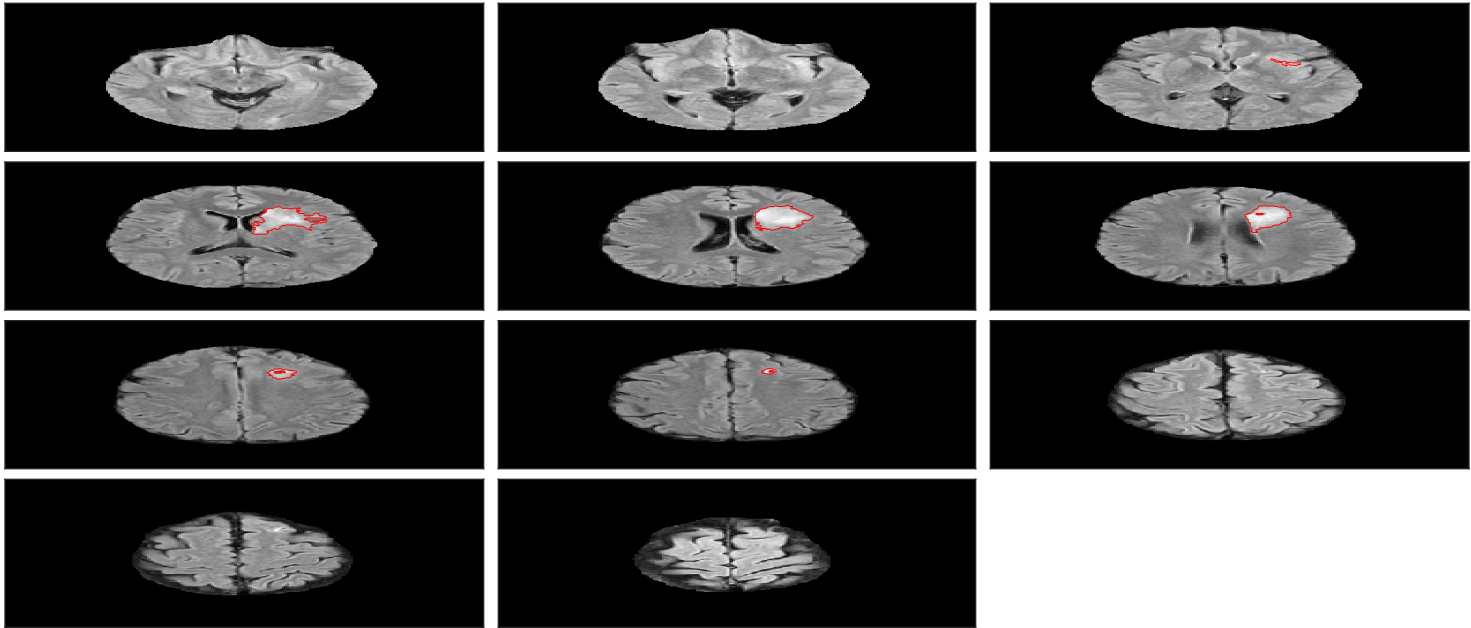

Supplement: S1 Data — The .zip file includes .pdf files labeled as follows, using case number 4224 as an example: 4224_t0.pdf includes the MRI data with segmentation of case number 4224 at baseline. 4224_t1_CAD_G_VC_S.pdf includes the MRI data with segmentation of case number 4224 at time 1, i.e., the time point of growth detection by the change-of-point method when the time point was different from the most recent MRI. 4224_tend_CAD_G_VC_G.pdf includes the MRI data with segmentation of case number 4224 at the most recent MRI. CAD_G and CAD_S mean that the change-of-point method predicted growth (G) or stability (S), respectively. VC_G and VC_S mean that the visual comparison detected growth (G) or stability (S), respectively. (ZIP) [file pmed.1002810.s001.zip › 7714_tend_CAD_S_VC_S.pdf]

Case Number:7472; Time 0 (Baseline).

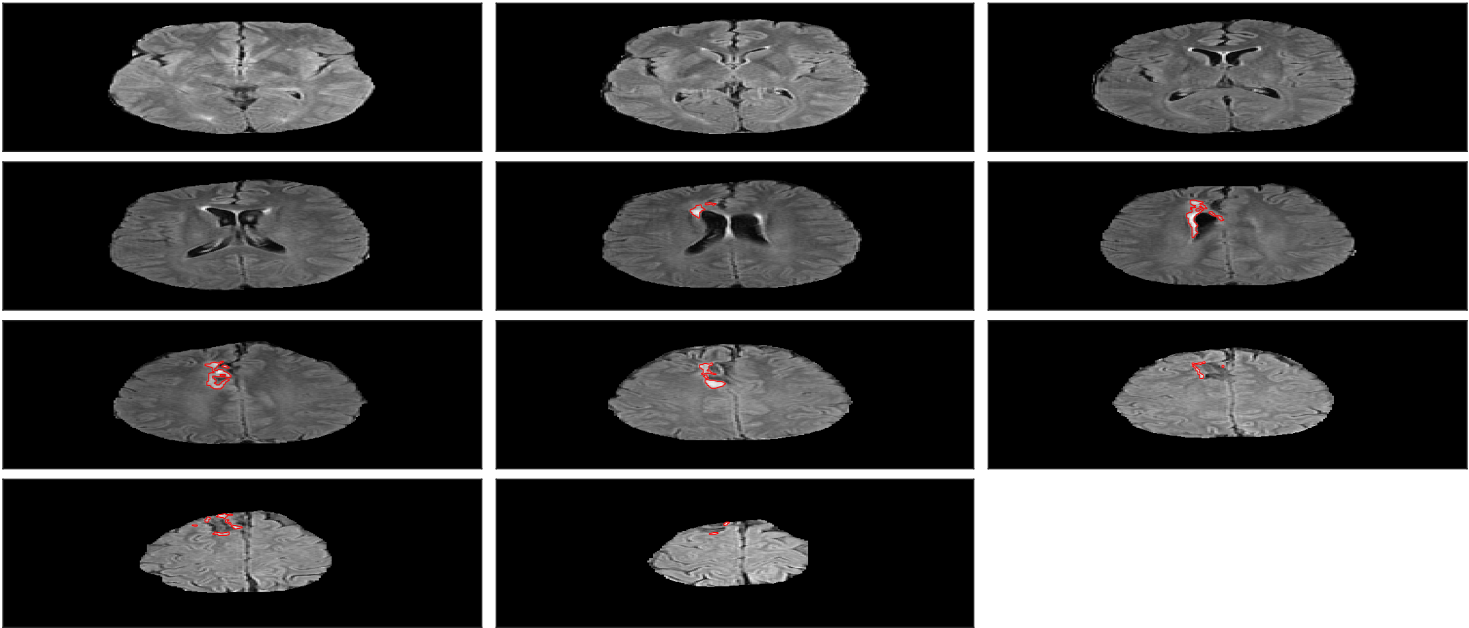

Supplement: S1 Data — The .zip file includes .pdf files labeled as follows, using case number 4224 as an example: 4224_t0.pdf includes the MRI data with segmentation of case number 4224 at baseline. 4224_t1_CAD_G_VC_S.pdf includes the MRI data with segmentation of case number 4224 at time 1, i.e., the time point of growth detection by the change-of-point method when the time point was different from the most recent MRI. 4224_tend_CAD_G_VC_G.pdf includes the MRI data with segmentation of case number 4224 at the most recent MRI. CAD_G and CAD_S mean that the change-of-point method predicted growth (G) or stability (S), respectively. VC_G and VC_S mean that the visual comparison detected growth (G) or stability (S), respectively. (ZIP) [file pmed.1002810.s001.zip › 7472_t0.pdf]

Case Number:7094; Time 0 (Baseline).

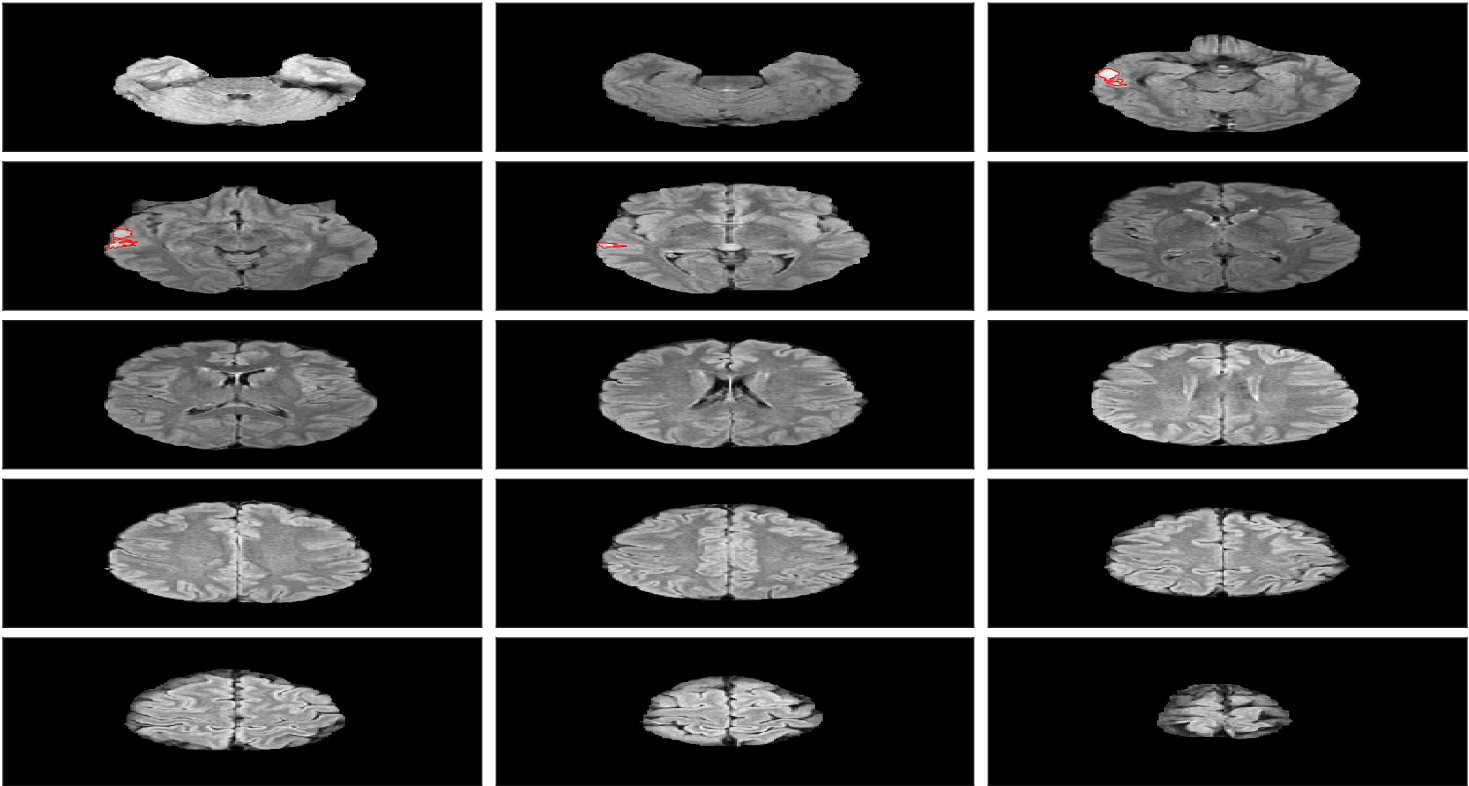

Supplement: S1 Data — The .zip file includes .pdf files labeled as follows, using case number 4224 as an example: 4224_t0.pdf includes the MRI data with segmentation of case number 4224 at baseline. 4224_t1_CAD_G_VC_S.pdf includes the MRI data with segmentation of case number 4224 at time 1, i.e., the time point of growth detection by the change-of-point method when the time point was different from the most recent MRI. 4224_tend_CAD_G_VC_G.pdf includes the MRI data with segmentation of case number 4224 at the most recent MRI. CAD_G and CAD_S mean that the change-of-point method predicted growth (G) or stability (S), respectively. VC_G and VC_S mean that the visual comparison detected growth (G) or stability (S), respectively. (ZIP) [file pmed.1002810.s001.zip › 7094_t0.pdf]

Case Number:7478; LAST MRI; CAD --> GROWTH; VC --> GROWTH; Interval From Baseline =65 months.

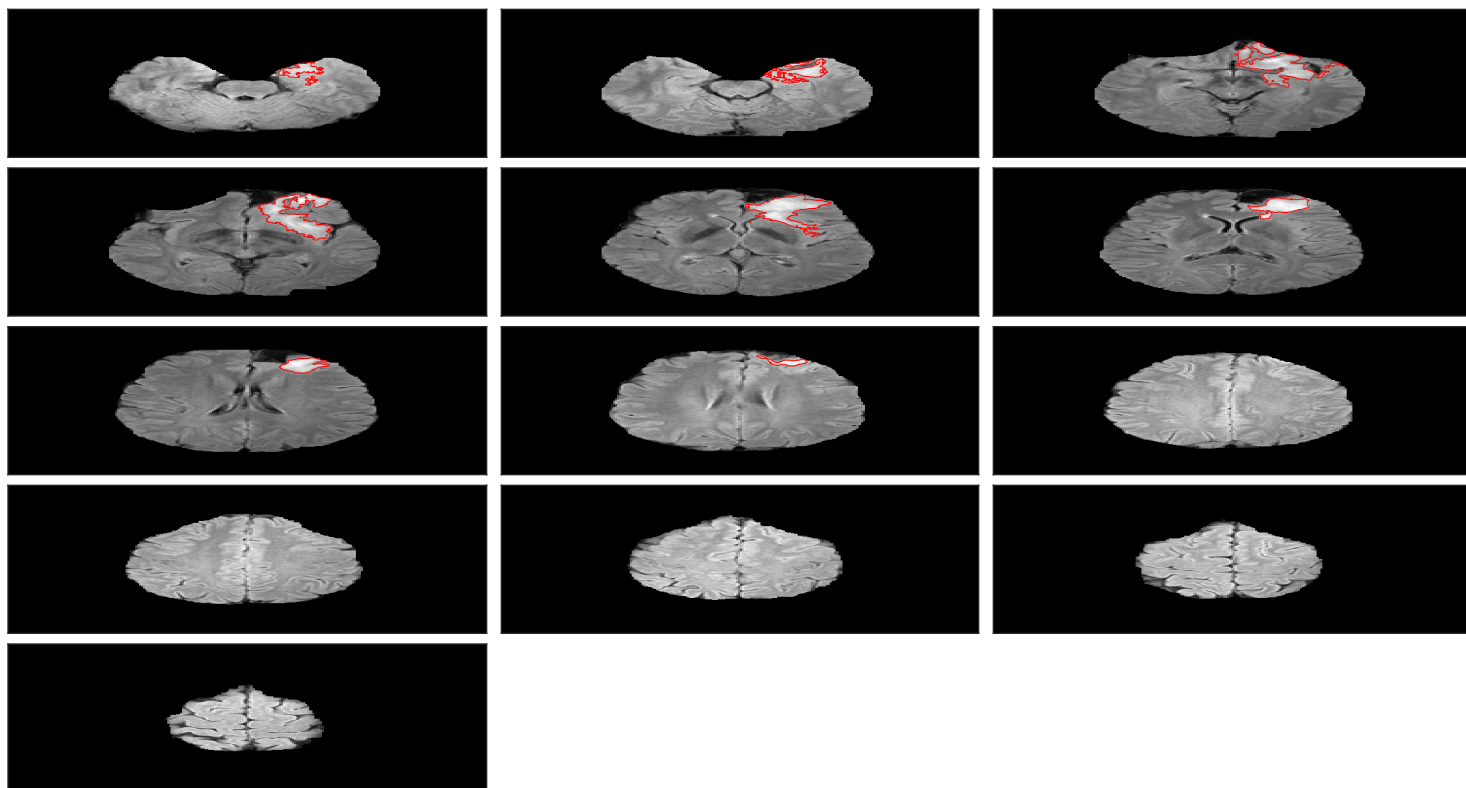

Supplement: S1 Data — The .zip file includes .pdf files labeled as follows, using case number 4224 as an example: 4224_t0.pdf includes the MRI data with segmentation of case number 4224 at baseline. 4224_t1_CAD_G_VC_S.pdf includes the MRI data with segmentation of case number 4224 at time 1, i.e., the time point of growth detection by the change-of-point method when the time point was different from the most recent MRI. 4224_tend_CAD_G_VC_G.pdf includes the MRI data with segmentation of case number 4224 at the most recent MRI. CAD_G and CAD_S mean that the change-of-point method predicted growth (G) or stability (S), respectively. VC_G and VC_S mean that the visual comparison detected growth (G) or stability (S), respectively. (ZIP) [file pmed.1002810.s001.zip › 7478_tend_CAD_G_VC_G.pdf]

Case Number:6935; Time 0 (Baseline).

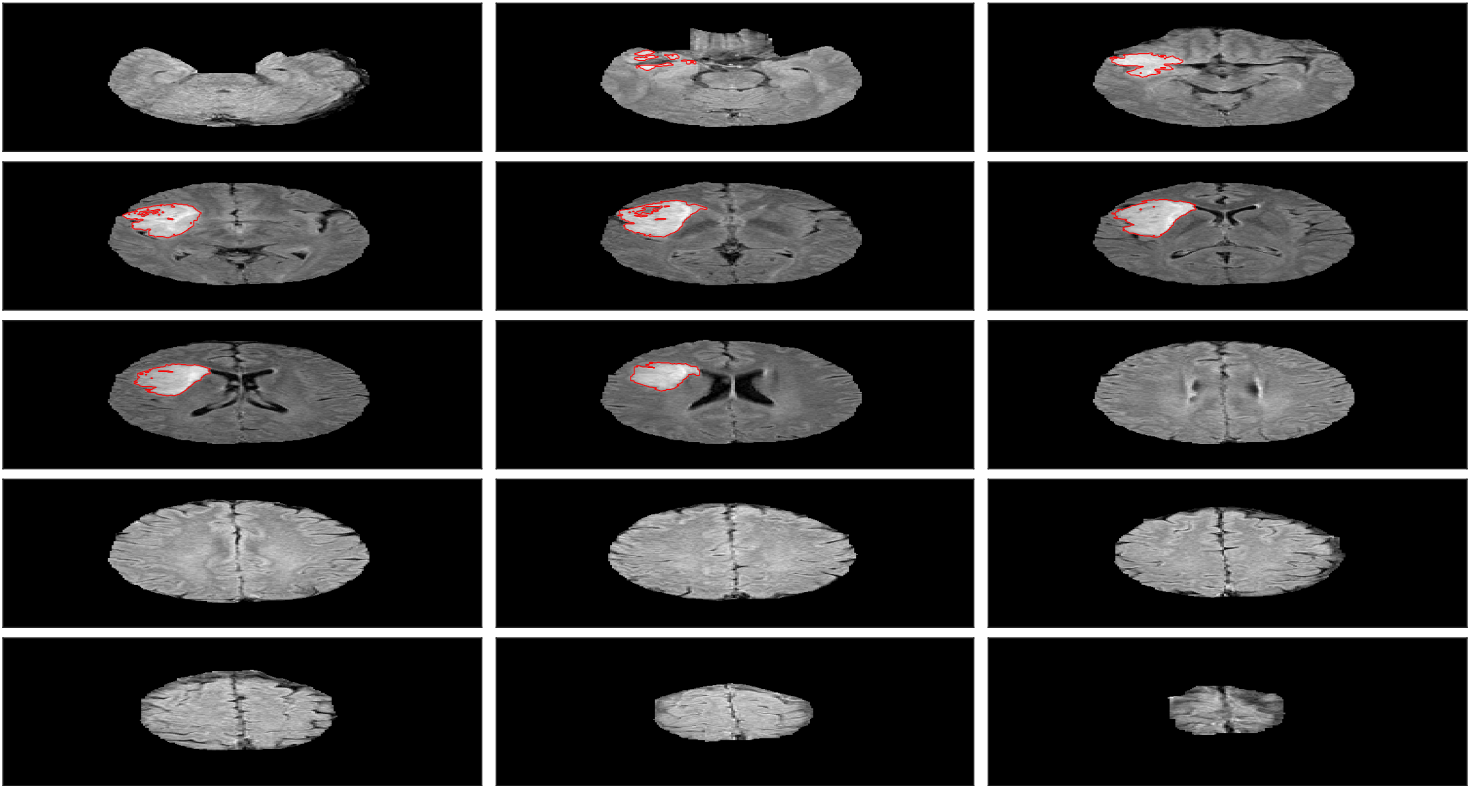

Supplement: S1 Data — The .zip file includes .pdf files labeled as follows, using case number 4224 as an example: 4224_t0.pdf includes the MRI data with segmentation of case number 4224 at baseline. 4224_t1_CAD_G_VC_S.pdf includes the MRI data with segmentation of case number 4224 at time 1, i.e., the time point of growth detection by the change-of-point method when the time point was different from the most recent MRI. 4224_tend_CAD_G_VC_G.pdf includes the MRI data with segmentation of case number 4224 at the most recent MRI. CAD_G and CAD_S mean that the change-of-point method predicted growth (G) or stability (S), respectively. VC_G and VC_S mean that the visual comparison detected growth (G) or stability (S), respectively. (ZIP) [file pmed.1002810.s001.zip › 6935_t0.pdf]

Case Number:7716; LAST MRI; CAD --> GROWTH; VC --> STABLE; Interval From Baseline =15 months.

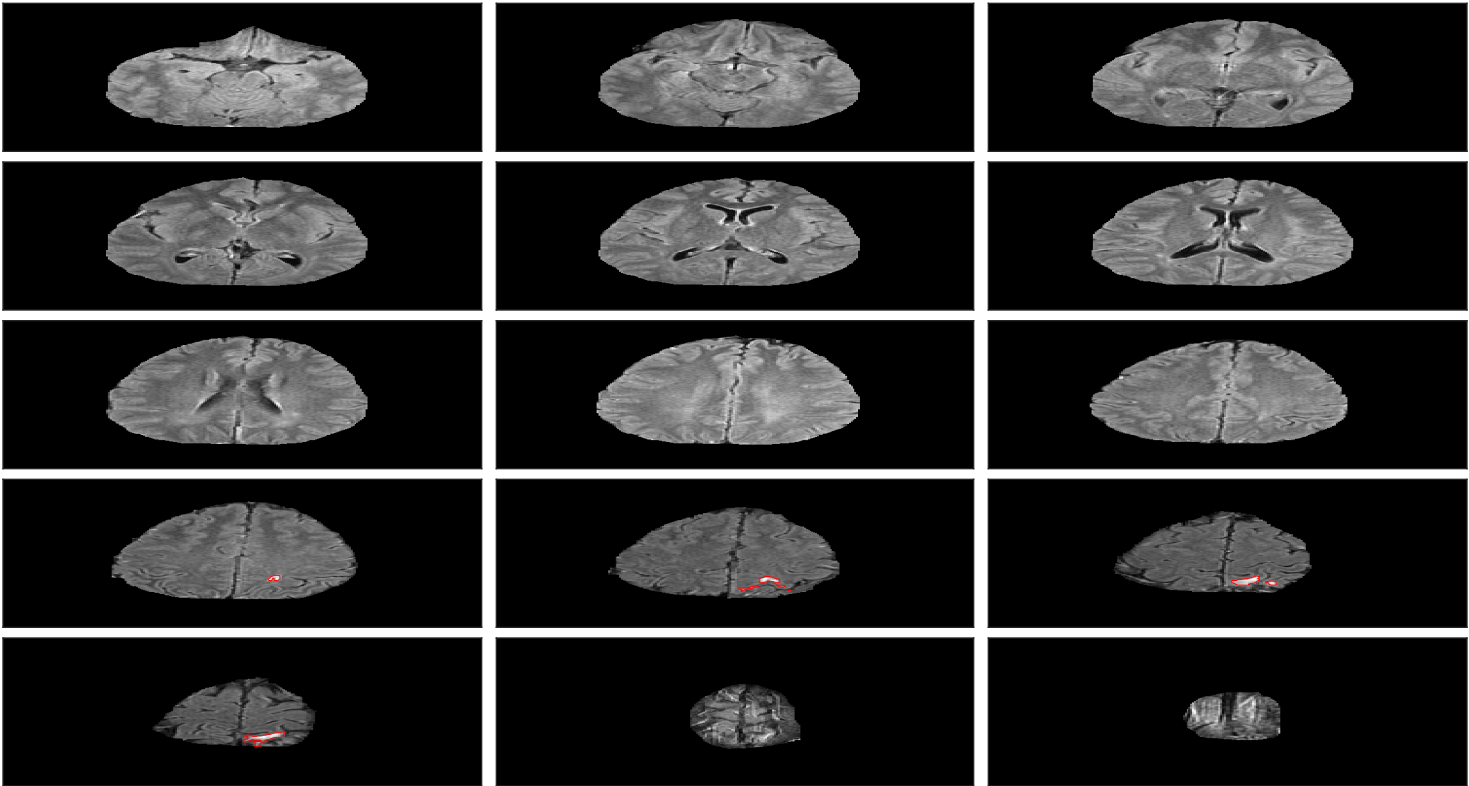

Supplement: S1 Data — The .zip file includes .pdf files labeled as follows, using case number 4224 as an example: 4224_t0.pdf includes the MRI data with segmentation of case number 4224 at baseline. 4224_t1_CAD_G_VC_S.pdf includes the MRI data with segmentation of case number 4224 at time 1, i.e., the time point of growth detection by the change-of-point method when the time point was different from the most recent MRI. 4224_tend_CAD_G_VC_G.pdf includes the MRI data with segmentation of case number 4224 at the most recent MRI. CAD_G and CAD_S mean that the change-of-point method predicted growth (G) or stability (S), respectively. VC_G and VC_S mean that the visual comparison detected growth (G) or stability (S), respectively. (ZIP) [file pmed.1002810.s001.zip › 7716_tend_CAD_G_VC_S.pdf]

Case Number:4225; LAST MRI; CAD --> GROWTH; VC --> GROWTH; Interval From Baseline =80 months.

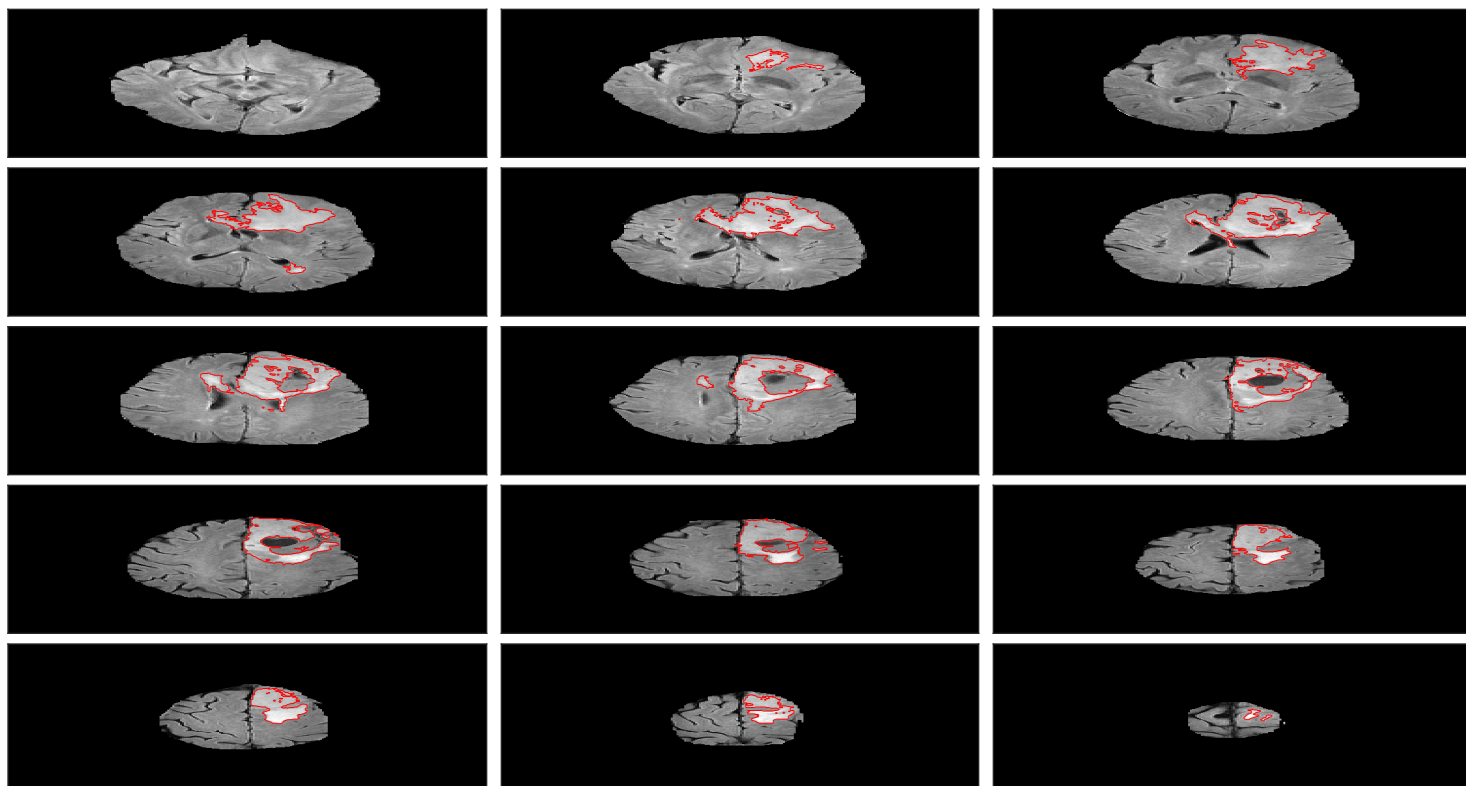

Supplement: S1 Data — The .zip file includes .pdf files labeled as follows, using case number 4224 as an example: 4224_t0.pdf includes the MRI data with segmentation of case number 4224 at baseline. 4224_t1_CAD_G_VC_S.pdf includes the MRI data with segmentation of case number 4224 at time 1, i.e., the time point of growth detection by the change-of-point method when the time point was different from the most recent MRI. 4224_tend_CAD_G_VC_G.pdf includes the MRI data with segmentation of case number 4224 at the most recent MRI. CAD_G and CAD_S mean that the change-of-point method predicted growth (G) or stability (S), respectively. VC_G and VC_S mean that the visual comparison detected growth (G) or stability (S), respectively. (ZIP) [file pmed.1002810.s001.zip › 4225_tend_CAD_G_VC_G.pdf]

Case Number:7333; Time 0 (Baseline).

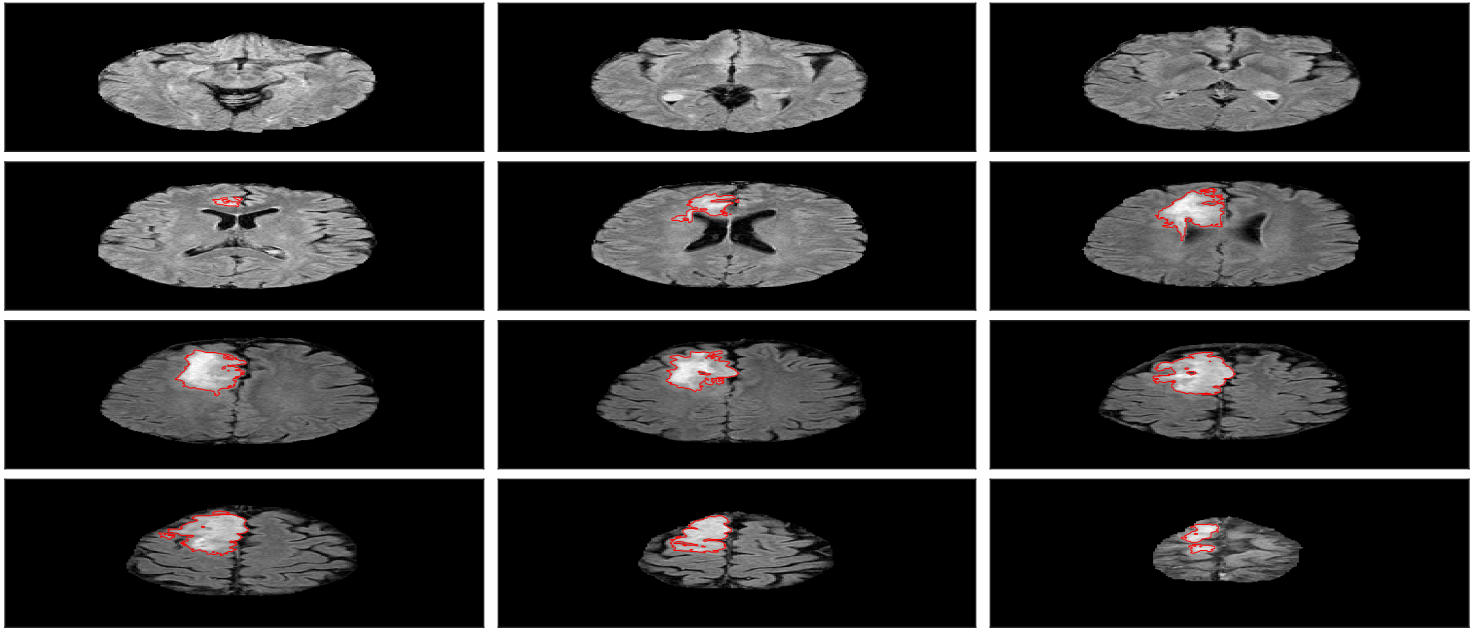

Supplement: S1 Data — The .zip file includes .pdf files labeled as follows, using case number 4224 as an example: 4224_t0.pdf includes the MRI data with segmentation of case number 4224 at baseline. 4224_t1_CAD_G_VC_S.pdf includes the MRI data with segmentation of case number 4224 at time 1, i.e., the time point of growth detection by the change-of-point method when the time point was different from the most recent MRI. 4224_tend_CAD_G_VC_G.pdf includes the MRI data with segmentation of case number 4224 at the most recent MRI. CAD_G and CAD_S mean that the change-of-point method predicted growth (G) or stability (S), respectively. VC_G and VC_S mean that the visual comparison detected growth (G) or stability (S), respectively. (ZIP) [file pmed.1002810.s001.zip › 7333_t0.pdf]

**Case Number:7505; TIME 1; CAD --> GROWTH; VC --> STABLE; Interval From Baseline =19 months.**

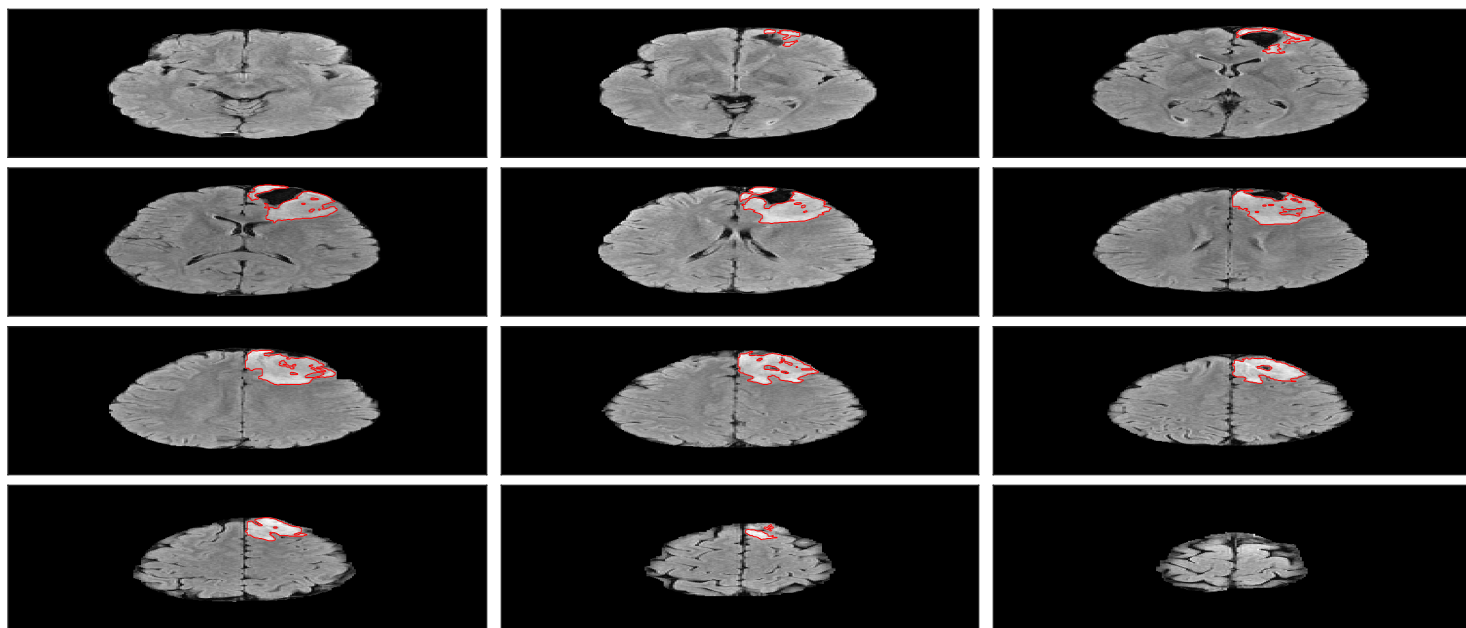

Supplement: S1 Data — The .zip file includes .pdf files labeled as follows, using case number 4224 as an example: 4224_t0.pdf includes the MRI data with segmentation of case number 4224 at baseline. 4224_t1_CAD_G_VC_S.pdf includes the MRI data with segmentation of case number 4224 at time 1, i.e., the time point of growth detection by the change-of-point method when the time point was different from the most recent MRI. 4224_tend_CAD_G_VC_G.pdf includes the MRI data with segmentation of case number 4224 at the most recent MRI. CAD_G and CAD_S mean that the change-of-point method predicted growth (G) or stability (S), respectively. VC_G and VC_S mean that the visual comparison detected growth (G) or stability (S), respectively. (ZIP) [file pmed.1002810.s001.zip › 7505_t1_CAD_G_VC_S.pdf]

Case Number:7335; TIME 1; CAD --> GROWTH; VC --> STABLE; Interval From Baseline =12 months.

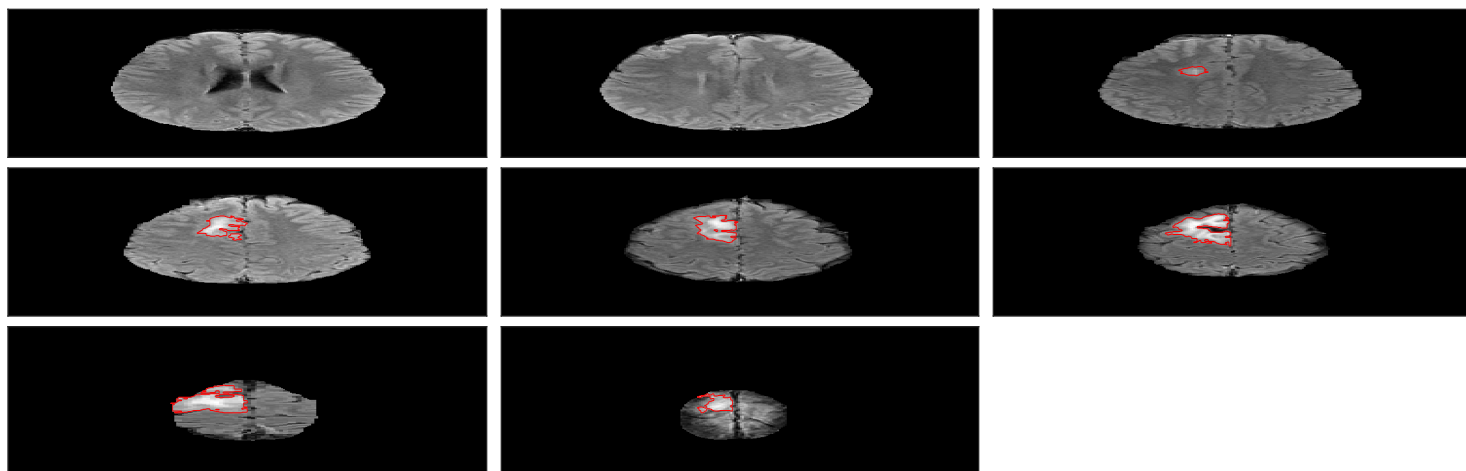

Supplement: S1 Data — The .zip file includes .pdf files labeled as follows, using case number 4224 as an example: 4224_t0.pdf includes the MRI data with segmentation of case number 4224 at baseline. 4224_t1_CAD_G_VC_S.pdf includes the MRI data with segmentation of case number 4224 at time 1, i.e., the time point of growth detection by the change-of-point method when the time point was different from the most recent MRI. 4224_tend_CAD_G_VC_G.pdf includes the MRI data with segmentation of case number 4224 at the most recent MRI. CAD_G and CAD_S mean that the change-of-point method predicted growth (G) or stability (S), respectively. VC_G and VC_S mean that the visual comparison detected growth (G) or stability (S), respectively. (ZIP) [file pmed.1002810.s001.zip › 7335_t1_CAD_G_VC_S.pdf]

Case Number:4224; LAST MRI; CAD --> GROWTH; VC --> GROWTH; Interval From Baseline =108 months.

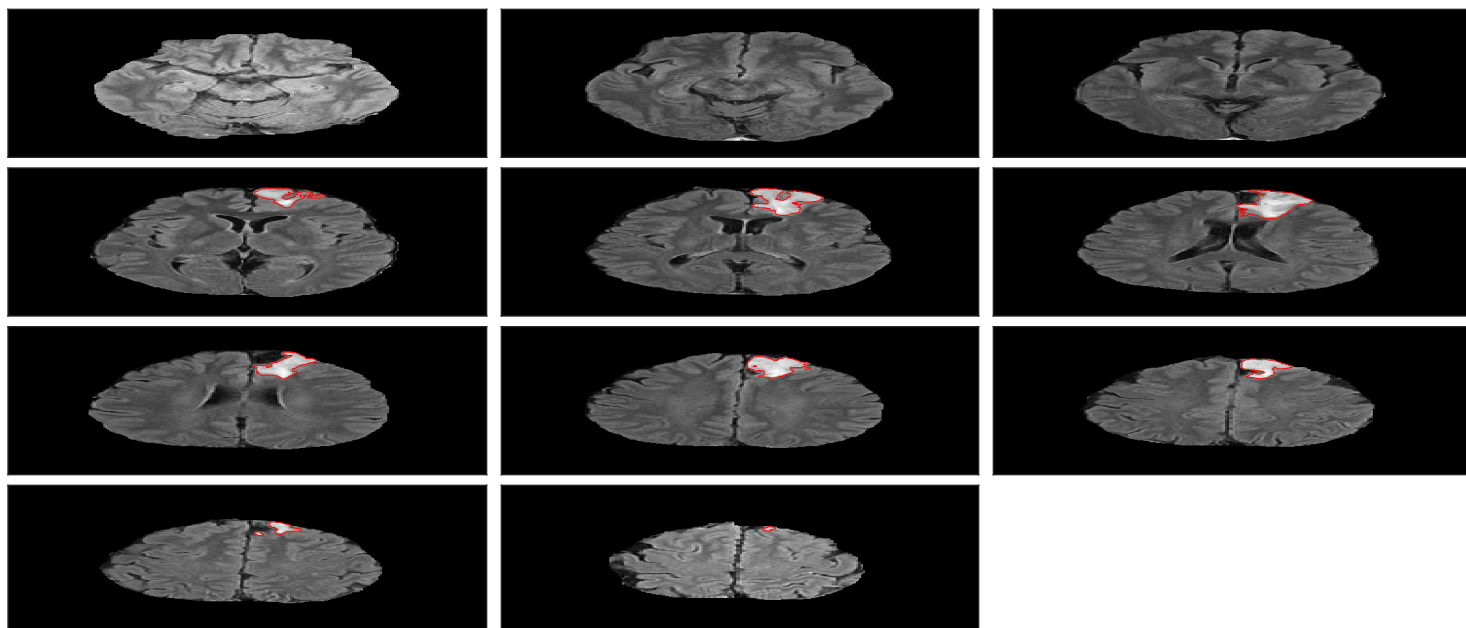

Supplement: S1 Data — The .zip file includes .pdf files labeled as follows, using case number 4224 as an example: 4224_t0.pdf includes the MRI data with segmentation of case number 4224 at baseline. 4224_t1_CAD_G_VC_S.pdf includes the MRI data with segmentation of case number 4224 at time 1, i.e., the time point of growth detection by the change-of-point method when the time point was different from the most recent MRI. 4224_tend_CAD_G_VC_G.pdf includes the MRI data with segmentation of case number 4224 at the most recent MRI. CAD_G and CAD_S mean that the change-of-point method predicted growth (G) or stability (S), respectively. VC_G and VC_S mean that the visual comparison detected growth (G) or stability (S), respectively. (ZIP) [file pmed.1002810.s001.zip › 4224_tend_CAD_G_VC_G.pdf]

Case Number:7470; Time 0 (Baseline).

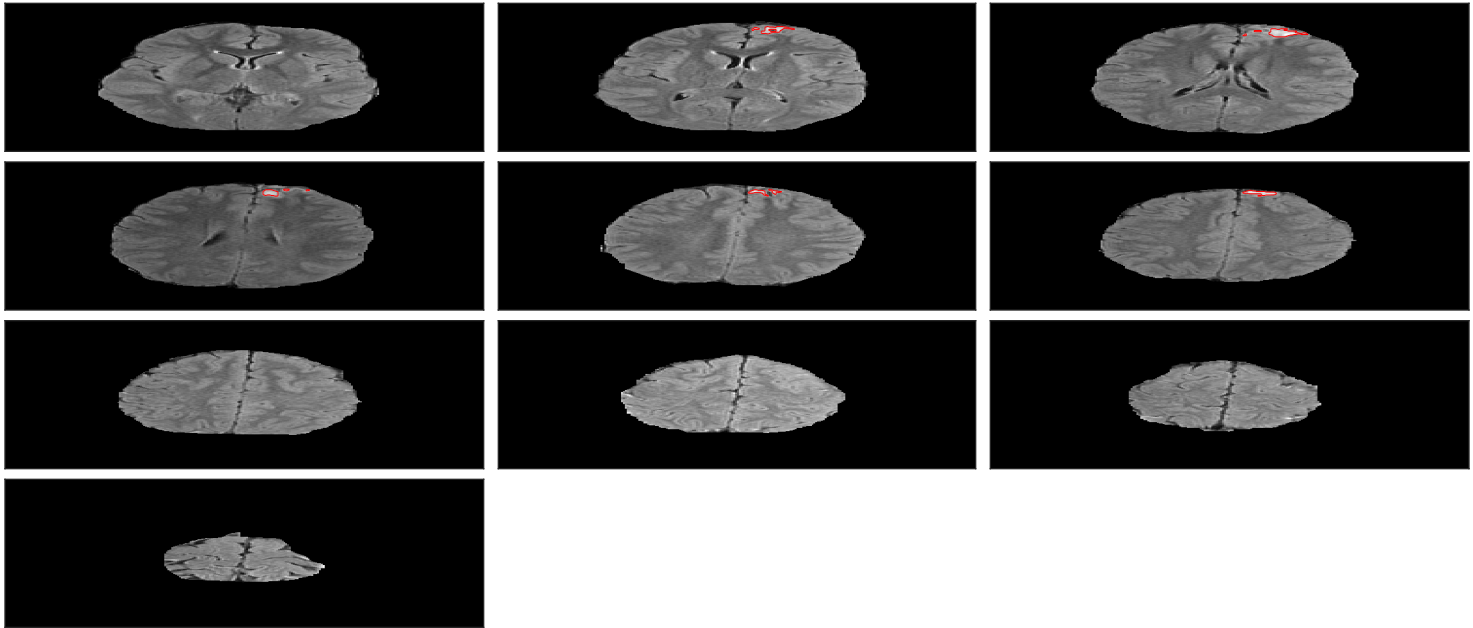

Supplement: S1 Data — The .zip file includes .pdf files labeled as follows, using case number 4224 as an example: 4224_t0.pdf includes the MRI data with segmentation of case number 4224 at baseline. 4224_t1_CAD_G_VC_S.pdf includes the MRI data with segmentation of case number 4224 at time 1, i.e., the time point of growth detection by the change-of-point method when the time point was different from the most recent MRI. 4224_tend_CAD_G_VC_G.pdf includes the MRI data with segmentation of case number 4224 at the most recent MRI. CAD_G and CAD_S mean that the change-of-point method predicted growth (G) or stability (S), respectively. VC_G and VC_S mean that the visual comparison detected growth (G) or stability (S), respectively. (ZIP) [file pmed.1002810.s001.zip › 7470_t0.pdf]

Case Number:7494; LAST MRI; CAD --> GROWTH; VC --> STABLE; Interval From Baseline =37 months.

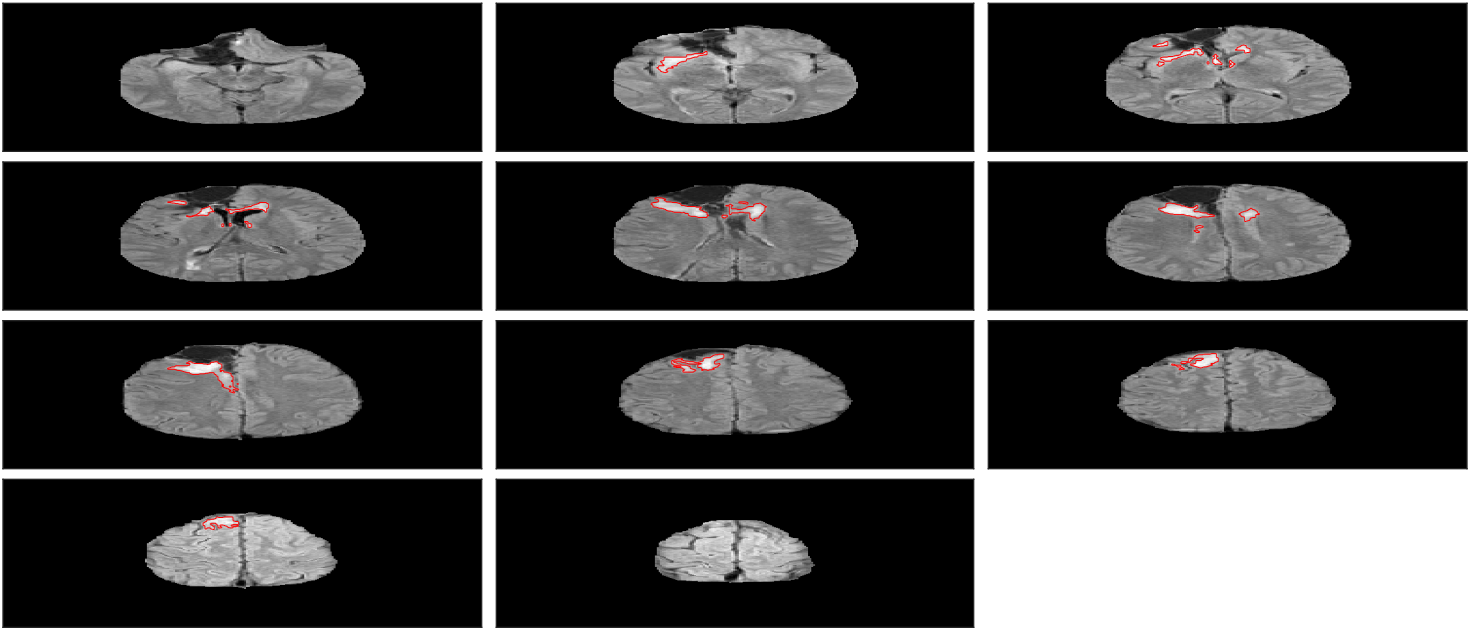

Supplement: S1 Data — The .zip file includes .pdf files labeled as follows, using case number 4224 as an example: 4224_t0.pdf includes the MRI data with segmentation of case number 4224 at baseline. 4224_t1_CAD_G_VC_S.pdf includes the MRI data with segmentation of case number 4224 at time 1, i.e., the time point of growth detection by the change-of-point method when the time point was different from the most recent MRI. 4224_tend_CAD_G_VC_G.pdf includes the MRI data with segmentation of case number 4224 at the most recent MRI. CAD_G and CAD_S mean that the change-of-point method predicted growth (G) or stability (S), respectively. VC_G and VC_S mean that the visual comparison detected growth (G) or stability (S), respectively. (ZIP) [file pmed.1002810.s001.zip › 7494_tend_CAD_G_VC_S.pdf]

Case Number:4225; TIME 1; CAD --> GROWTH; VC --> STABLE; Interval From Baseline =8 months.

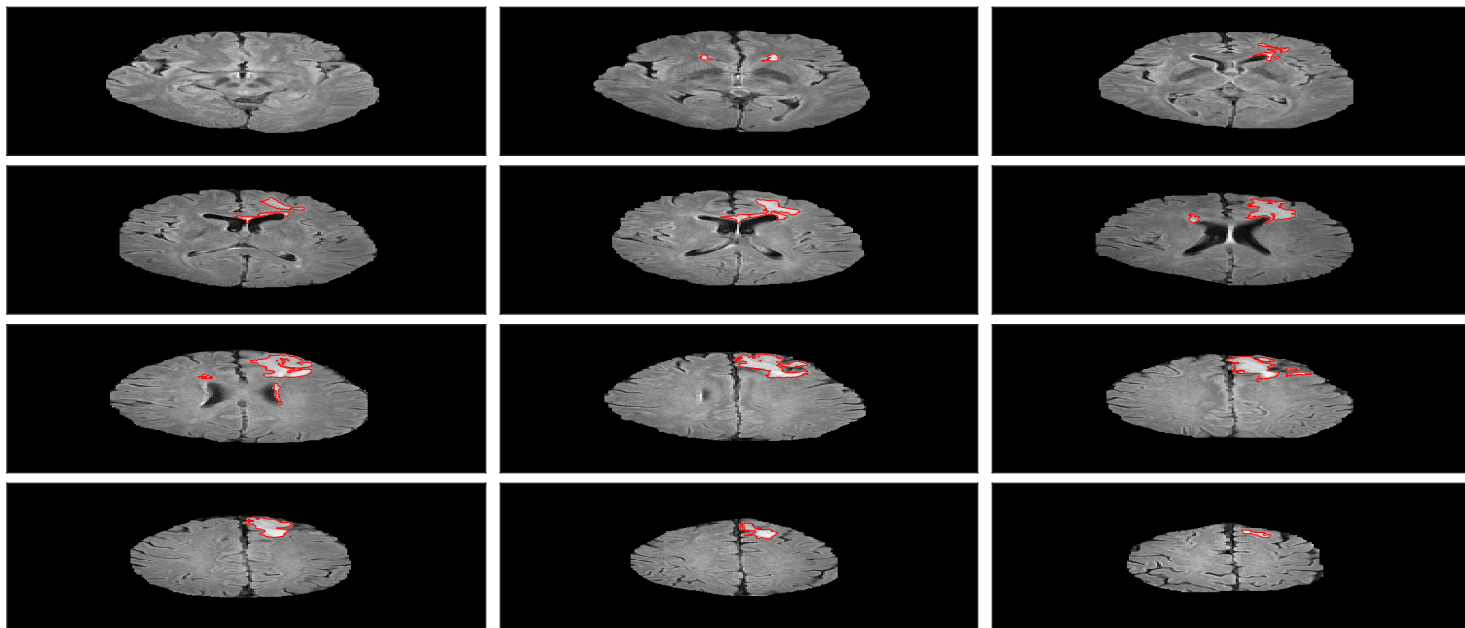

Supplement: S1 Data — The .zip file includes .pdf files labeled as follows, using case number 4224 as an example: 4224_t0.pdf includes the MRI data with segmentation of case number 4224 at baseline. 4224_t1_CAD_G_VC_S.pdf includes the MRI data with segmentation of case number 4224 at time 1, i.e., the time point of growth detection by the change-of-point method when the time point was different from the most recent MRI. 4224_tend_CAD_G_VC_G.pdf includes the MRI data with segmentation of case number 4224 at the most recent MRI. CAD_G and CAD_S mean that the change-of-point method predicted growth (G) or stability (S), respectively. VC_G and VC_S mean that the visual comparison detected growth (G) or stability (S), respectively. (ZIP) [file pmed.1002810.s001.zip › 4225_t1_CAD_G_VC_S.pdf]

Case Number:7710; Time 0 (Baseline).

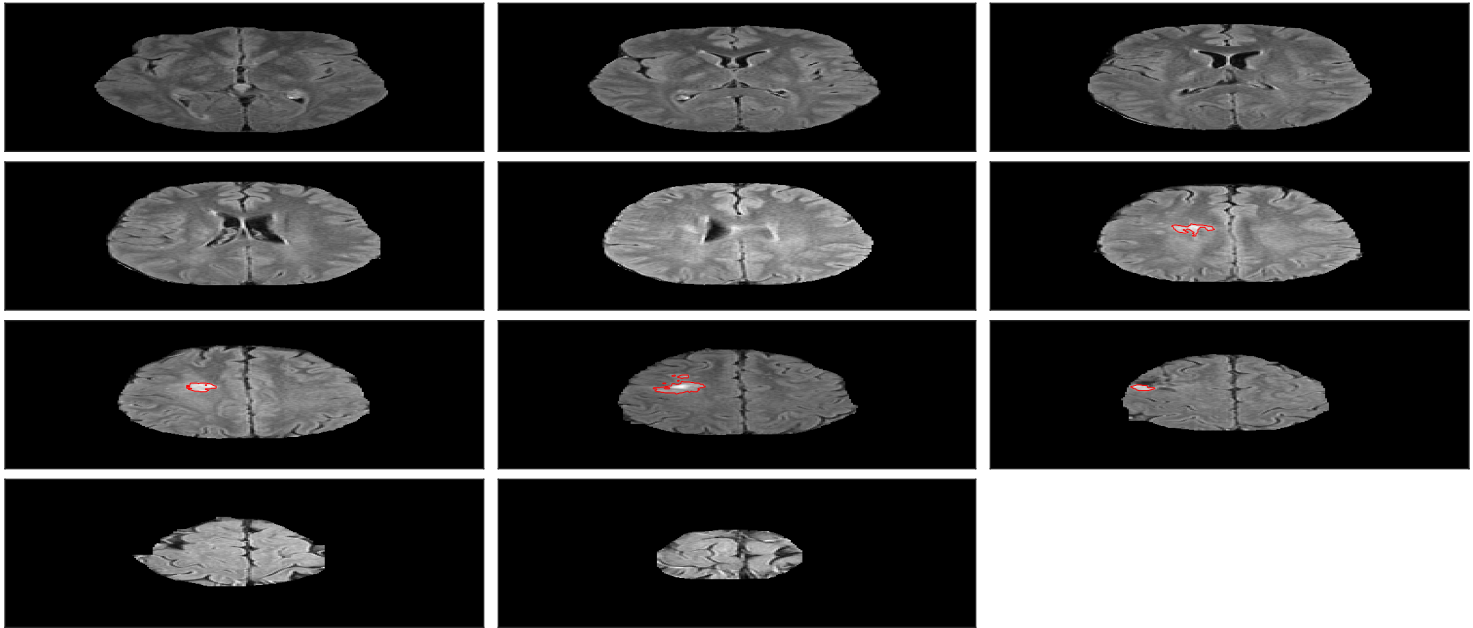

Supplement: S1 Data — The .zip file includes .pdf files labeled as follows, using case number 4224 as an example: 4224_t0.pdf includes the MRI data with segmentation of case number 4224 at baseline. 4224_t1_CAD_G_VC_S.pdf includes the MRI data with segmentation of case number 4224 at time 1, i.e., the time point of growth detection by the change-of-point method when the time point was different from the most recent MRI. 4224_tend_CAD_G_VC_G.pdf includes the MRI data with segmentation of case number 4224 at the most recent MRI. CAD_G and CAD_S mean that the change-of-point method predicted growth (G) or stability (S), respectively. VC_G and VC_S mean that the visual comparison detected growth (G) or stability (S), respectively. (ZIP) [file pmed.1002810.s001.zip › 7710_t0.pdf]

Case Number:6935; TIME 1; CAD --> GROWTH; VC --> STABLE; Interval From Baseline =31 months.

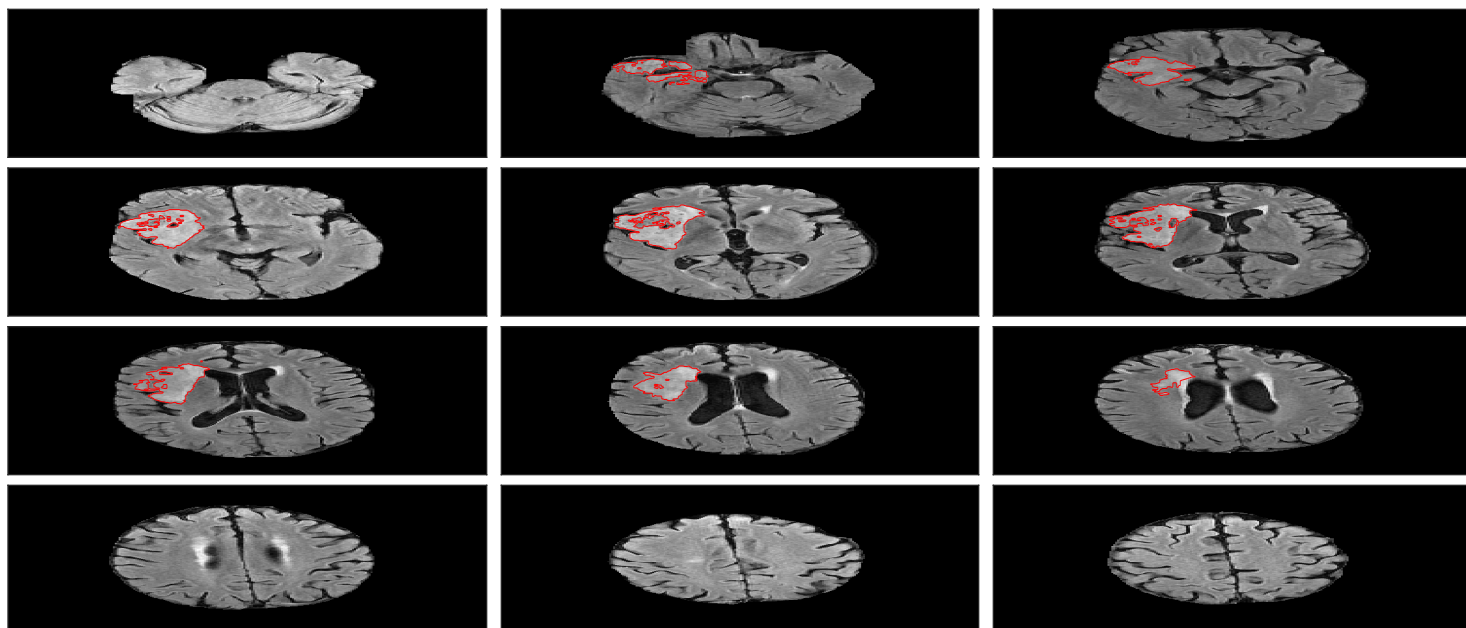

Supplement: S1 Data — The .zip file includes .pdf files labeled as follows, using case number 4224 as an example: 4224_t0.pdf includes the MRI data with segmentation of case number 4224 at baseline. 4224_t1_CAD_G_VC_S.pdf includes the MRI data with segmentation of case number 4224 at time 1, i.e., the time point of growth detection by the change-of-point method when the time point was different from the most recent MRI. 4224_tend_CAD_G_VC_G.pdf includes the MRI data with segmentation of case number 4224 at the most recent MRI. CAD_G and CAD_S mean that the change-of-point method predicted growth (G) or stability (S), respectively. VC_G and VC_S mean that the visual comparison detected growth (G) or stability (S), respectively. (ZIP) [file pmed.1002810.s001.zip › 6935_t1_CAD_G_VC_S.pdf]

**Case Number:7710; LAST MRI; CAD --> STABLE; VC --> STABLE; Interval From Baseline =4 months.**

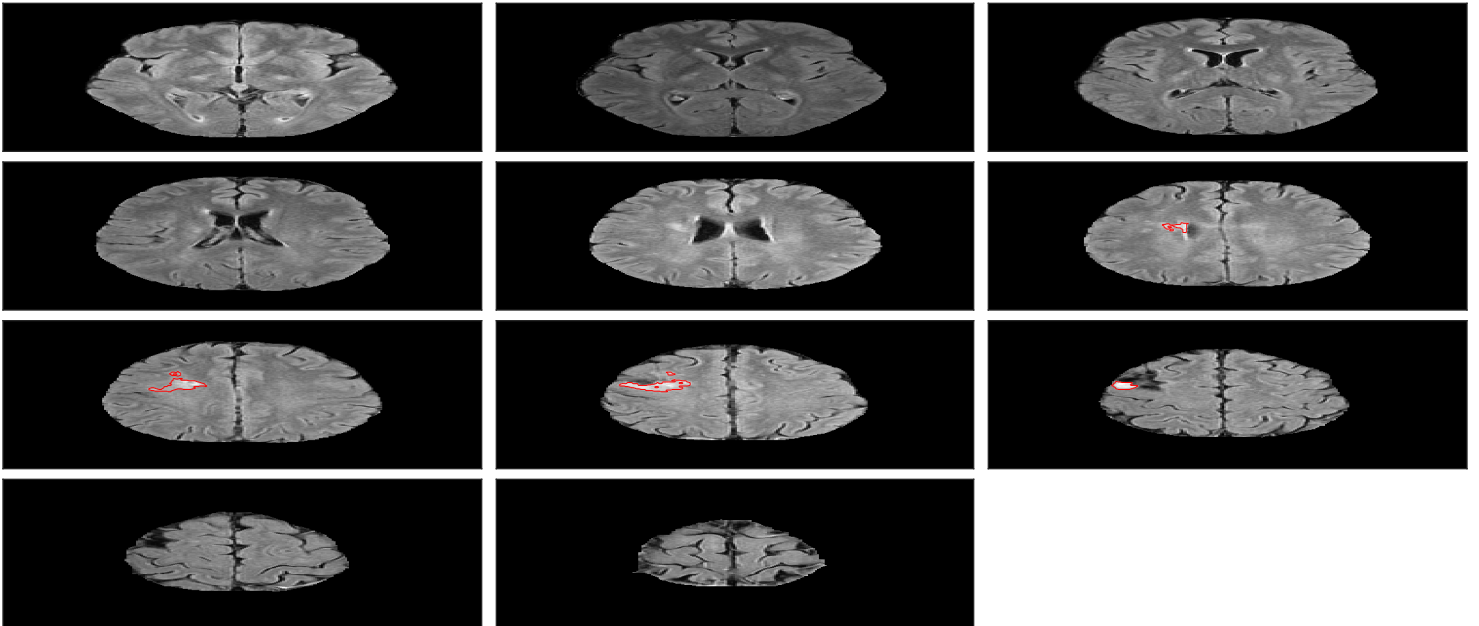

Supplement: S1 Data — The .zip file includes .pdf files labeled as follows, using case number 4224 as an example: 4224_t0.pdf includes the MRI data with segmentation of case number 4224 at baseline. 4224_t1_CAD_G_VC_S.pdf includes the MRI data with segmentation of case number 4224 at time 1, i.e., the time point of growth detection by the change-of-point method when the time point was different from the most recent MRI. 4224_tend_CAD_G_VC_G.pdf includes the MRI data with segmentation of case number 4224 at the most recent MRI. CAD_G and CAD_S mean that the change-of-point method predicted growth (G) or stability (S), respectively. VC_G and VC_S mean that the visual comparison detected growth (G) or stability (S), respectively. (ZIP) [file pmed.1002810.s001.zip › 7710_tend_CAD_S_VC_S.pdf]

Case Number:7717; LAST MRI; CAD --> GROWTH; VC --> GROWTH; Interval From Baseline =66 months.

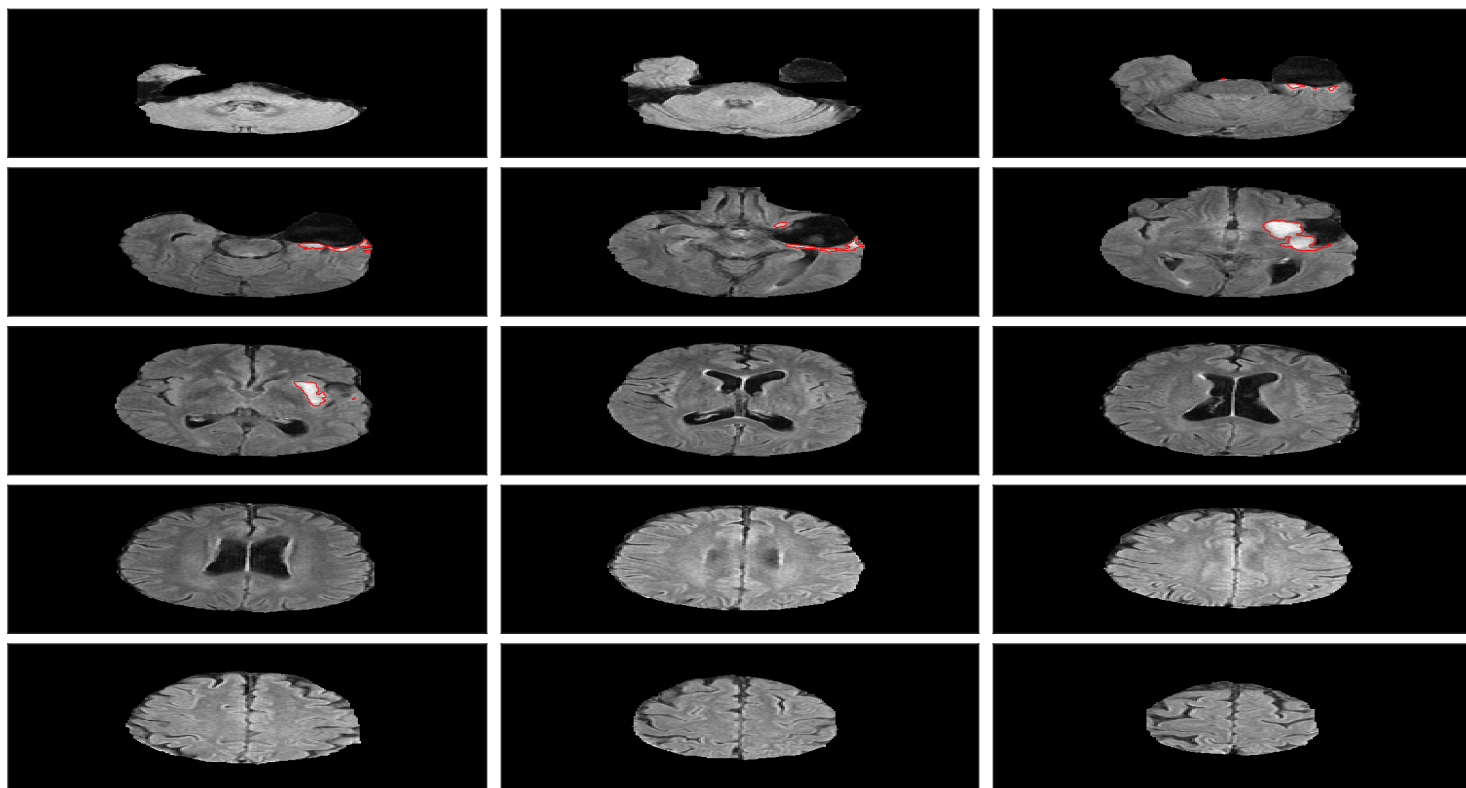

Supplement: S1 Data — The .zip file includes .pdf files labeled as follows, using case number 4224 as an example: 4224_t0.pdf includes the MRI data with segmentation of case number 4224 at baseline. 4224_t1_CAD_G_VC_S.pdf includes the MRI data with segmentation of case number 4224 at time 1, i.e., the time point of growth detection by the change-of-point method when the time point was different from the most recent MRI. 4224_tend_CAD_G_VC_G.pdf includes the MRI data with segmentation of case number 4224 at the most recent MRI. CAD_G and CAD_S mean that the change-of-point method predicted growth (G) or stability (S), respectively. VC_G and VC_S mean that the visual comparison detected growth (G) or stability (S), respectively. (ZIP) [file pmed.1002810.s001.zip › 7717_tend_CAD_G_VC_G.pdf]

Case Number:7749; Time 0 (Baseline).

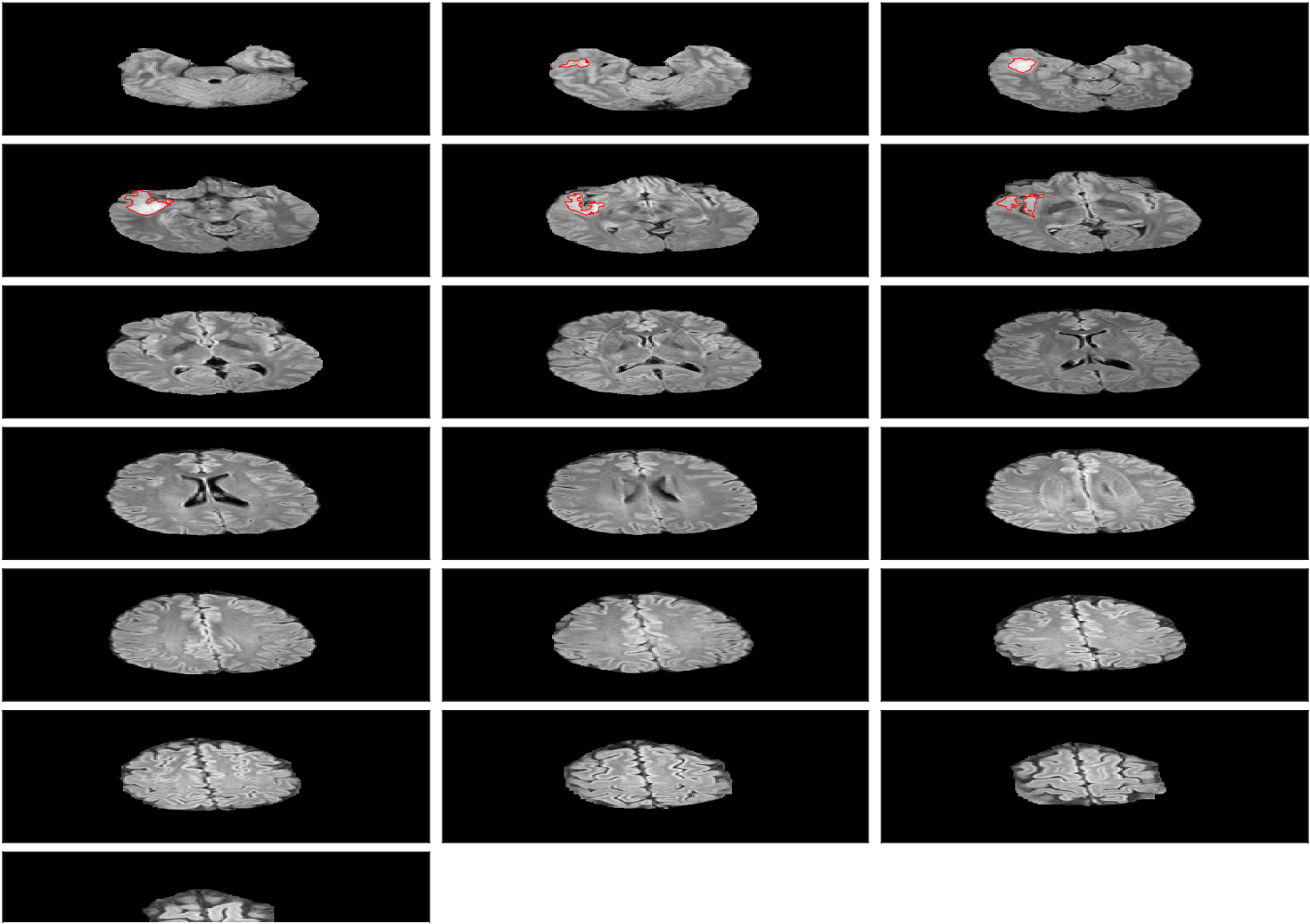

Supplement: S1 Data — The .zip file includes .pdf files labeled as follows, using case number 4224 as an example: 4224_t0.pdf includes the MRI data with segmentation of case number 4224 at baseline. 4224_t1_CAD_G_VC_S.pdf includes the MRI data with segmentation of case number 4224 at time 1, i.e., the time point of growth detection by the change-of-point method when the time point was different from the most recent MRI. 4224_tend_CAD_G_VC_G.pdf includes the MRI data with segmentation of case number 4224 at the most recent MRI. CAD_G and CAD_S mean that the change-of-point method predicted growth (G) or stability (S), respectively. VC_G and VC_S mean that the visual comparison detected growth (G) or stability (S), respectively. (ZIP) [file pmed.1002810.s001.zip › 7749_t0.pdf]

Case Number:7734; Time 0 (Baseline).

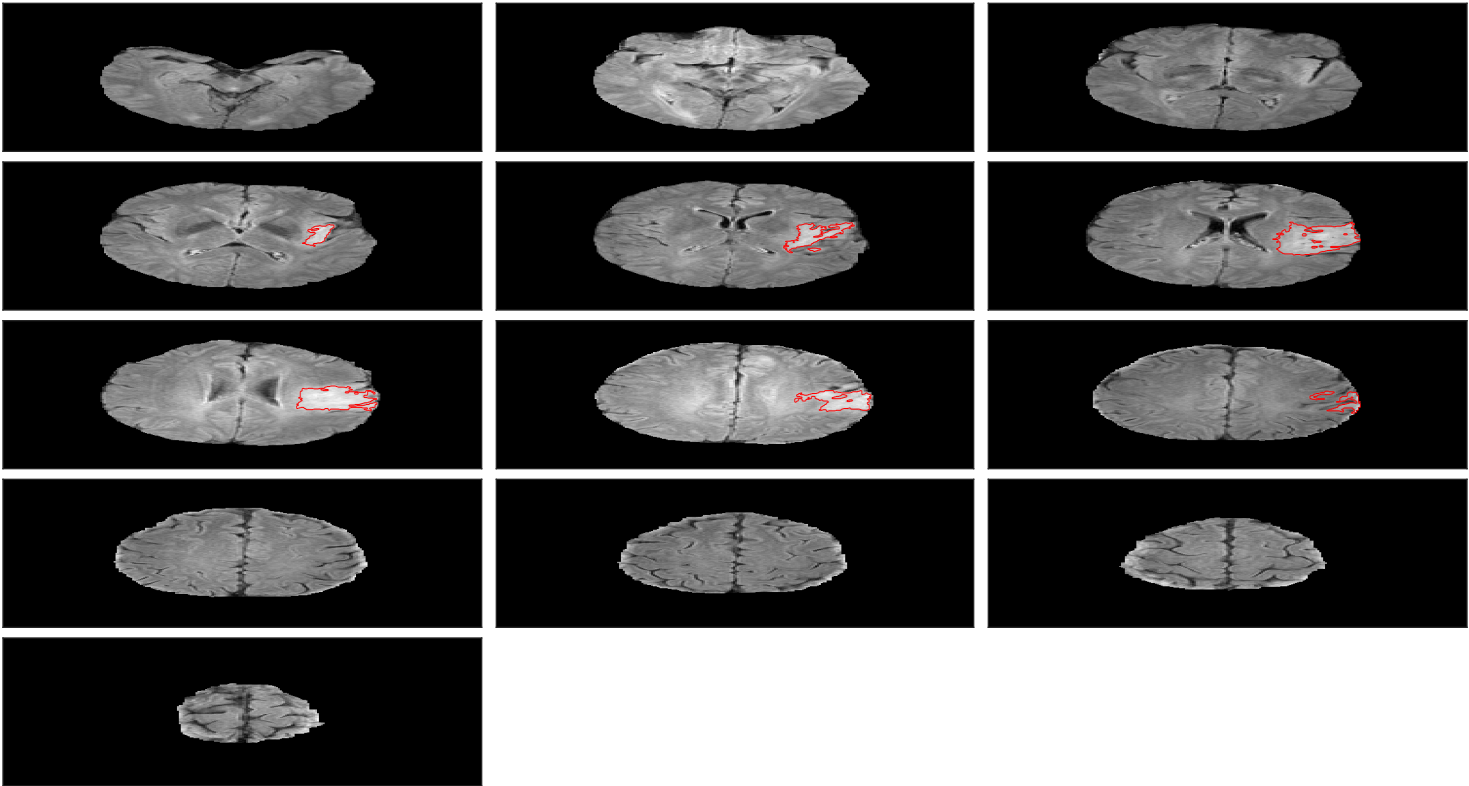

Supplement: S1 Data — The .zip file includes .pdf files labeled as follows, using case number 4224 as an example: 4224_t0.pdf includes the MRI data with segmentation of case number 4224 at baseline. 4224_t1_CAD_G_VC_S.pdf includes the MRI data with segmentation of case number 4224 at time 1, i.e., the time point of growth detection by the change-of-point method when the time point was different from the most recent MRI. 4224_tend_CAD_G_VC_G.pdf includes the MRI data with segmentation of case number 4224 at the most recent MRI. CAD_G and CAD_S mean that the change-of-point method predicted growth (G) or stability (S), respectively. VC_G and VC_S mean that the visual comparison detected growth (G) or stability (S), respectively. (ZIP) [file pmed.1002810.s001.zip › 7734_t0.pdf]

Case Number:7069; Time 0 (Baseline).

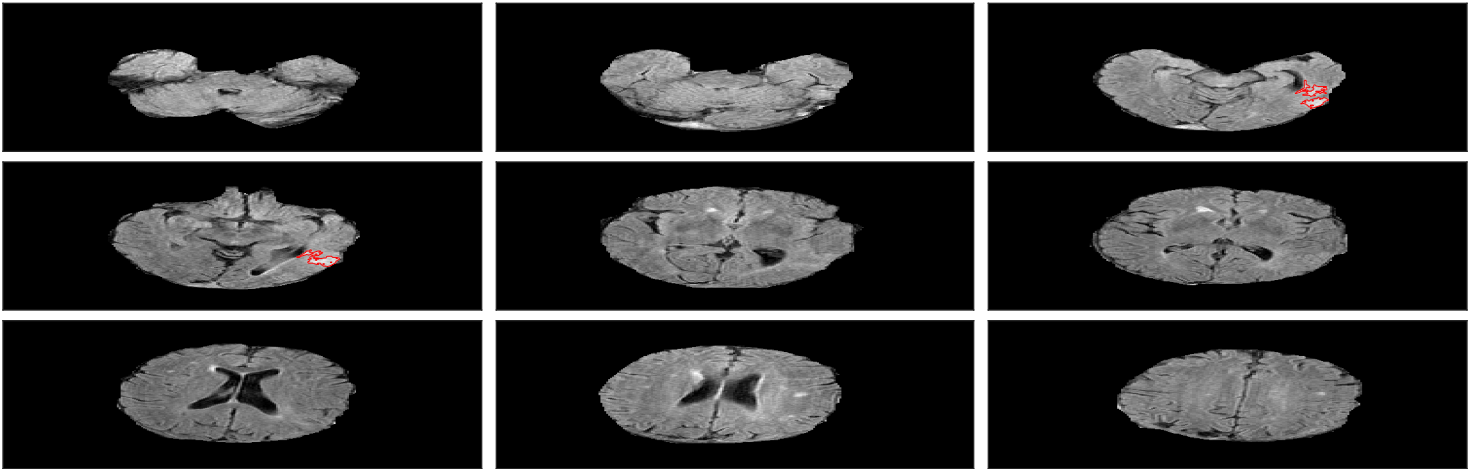

Supplement: S1 Data — The .zip file includes .pdf files labeled as follows, using case number 4224 as an example: 4224_t0.pdf includes the MRI data with segmentation of case number 4224 at baseline. 4224_t1_CAD_G_VC_S.pdf includes the MRI data with segmentation of case number 4224 at time 1, i.e., the time point of growth detection by the change-of-point method when the time point was different from the most recent MRI. 4224_tend_CAD_G_VC_G.pdf includes the MRI data with segmentation of case number 4224 at the most recent MRI. CAD_G and CAD_S mean that the change-of-point method predicted growth (G) or stability (S), respectively. VC_G and VC_S mean that the visual comparison detected growth (G) or stability (S), respectively. (ZIP) [file pmed.1002810.s001.zip › 7069_t0.pdf]

Case Number:7491; Time 0 (Baseline).

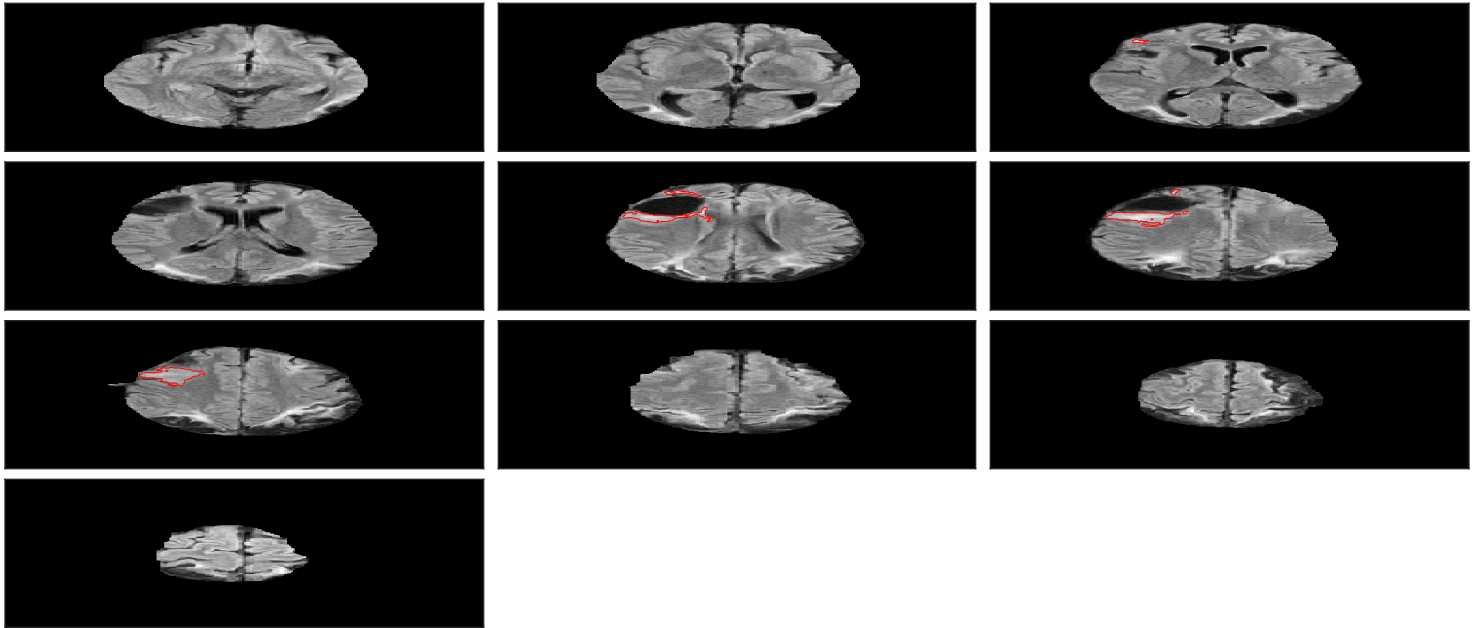

Supplement: S1 Data — The .zip file includes .pdf files labeled as follows, using case number 4224 as an example: 4224_t0.pdf includes the MRI data with segmentation of case number 4224 at baseline. 4224_t1_CAD_G_VC_S.pdf includes the MRI data with segmentation of case number 4224 at time 1, i.e., the time point of growth detection by the change-of-point method when the time point was different from the most recent MRI. 4224_tend_CAD_G_VC_G.pdf includes the MRI data with segmentation of case number 4224 at the most recent MRI. CAD_G and CAD_S mean that the change-of-point method predicted growth (G) or stability (S), respectively. VC_G and VC_S mean that the visual comparison detected growth (G) or stability (S), respectively. (ZIP) [file pmed.1002810.s001.zip › 7491_t0.pdf]
