# Supplementary figures and images for: Diagnosing growth in low-grade gliomas with and without longitudinal volume measurements: A retrospective observational study (part 1 of 2)
Source: PLoS Med. 2019 May 28;16(5):e1002810. doi: 10.1371/journal.pmed.1002810 (PMC6538148; doi:10.1371/journal.pmed.1002810)

Case Number:7727; LAST MRI; CAD --> GROWTH; VC --> GROWTH; Interval From Baseline =9 months.

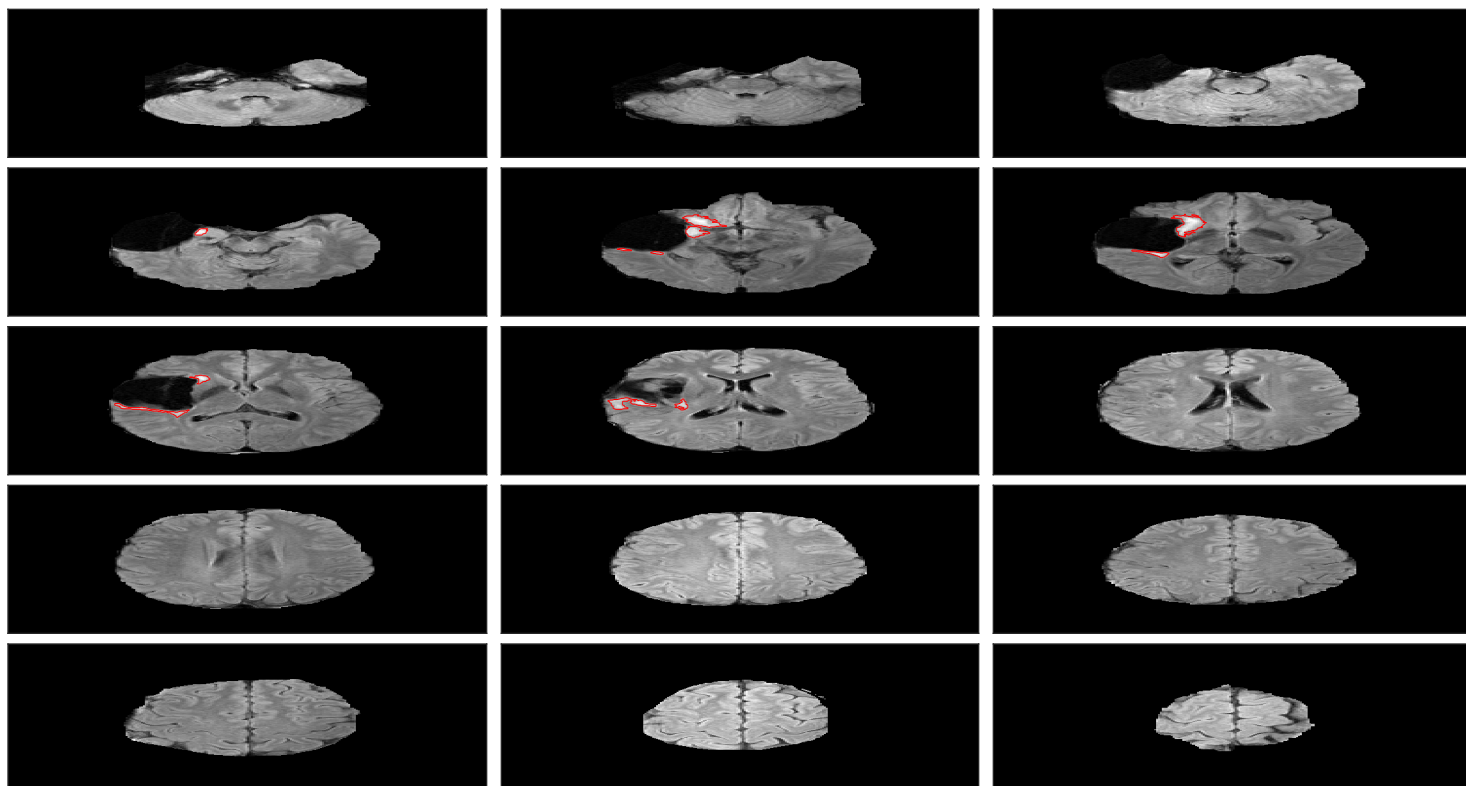

Supplement: S1 Data — The .zip file includes .pdf files labeled as follows, using case number 4224 as an example: 4224_t0.pdf includes the MRI data with segmentation of case number 4224 at baseline. 4224_t1_CAD_G_VC_S.pdf includes the MRI data with segmentation of case number 4224 at time 1, i.e., the time point of growth detection by the change-of-point method when the time point was different from the most recent MRI. 4224_tend_CAD_G_VC_G.pdf includes the MRI data with segmentation of case number 4224 at the most recent MRI. CAD_G and CAD_S mean that the change-of-point method predicted growth (G) or stability (S), respectively. VC_G and VC_S mean that the visual comparison detected growth (G) or stability (S), respectively. (ZIP) [file pmed.1002810.s001.zip › 7727_tend_CAD_G_VC_G.pdf]

Case Number:7707; Time 0 (Baseline).

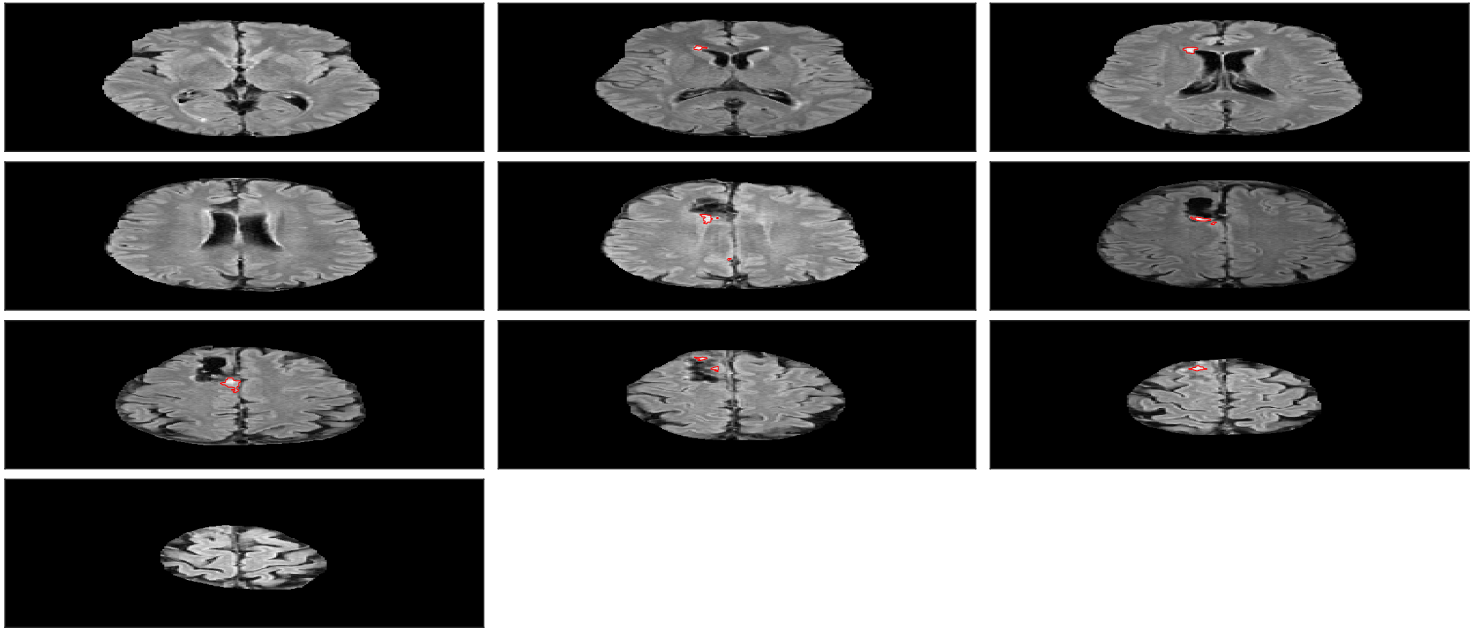

Supplement: S1 Data — The .zip file includes .pdf files labeled as follows, using case number 4224 as an example: 4224_t0.pdf includes the MRI data with segmentation of case number 4224 at baseline. 4224_t1_CAD_G_VC_S.pdf includes the MRI data with segmentation of case number 4224 at time 1, i.e., the time point of growth detection by the change-of-point method when the time point was different from the most recent MRI. 4224_tend_CAD_G_VC_G.pdf includes the MRI data with segmentation of case number 4224 at the most recent MRI. CAD_G and CAD_S mean that the change-of-point method predicted growth (G) or stability (S), respectively. VC_G and VC_S mean that the visual comparison detected growth (G) or stability (S), respectively. (ZIP) [file pmed.1002810.s001.zip › 7707_t0.pdf]

Case Number:7715; Time 0 (Baseline).

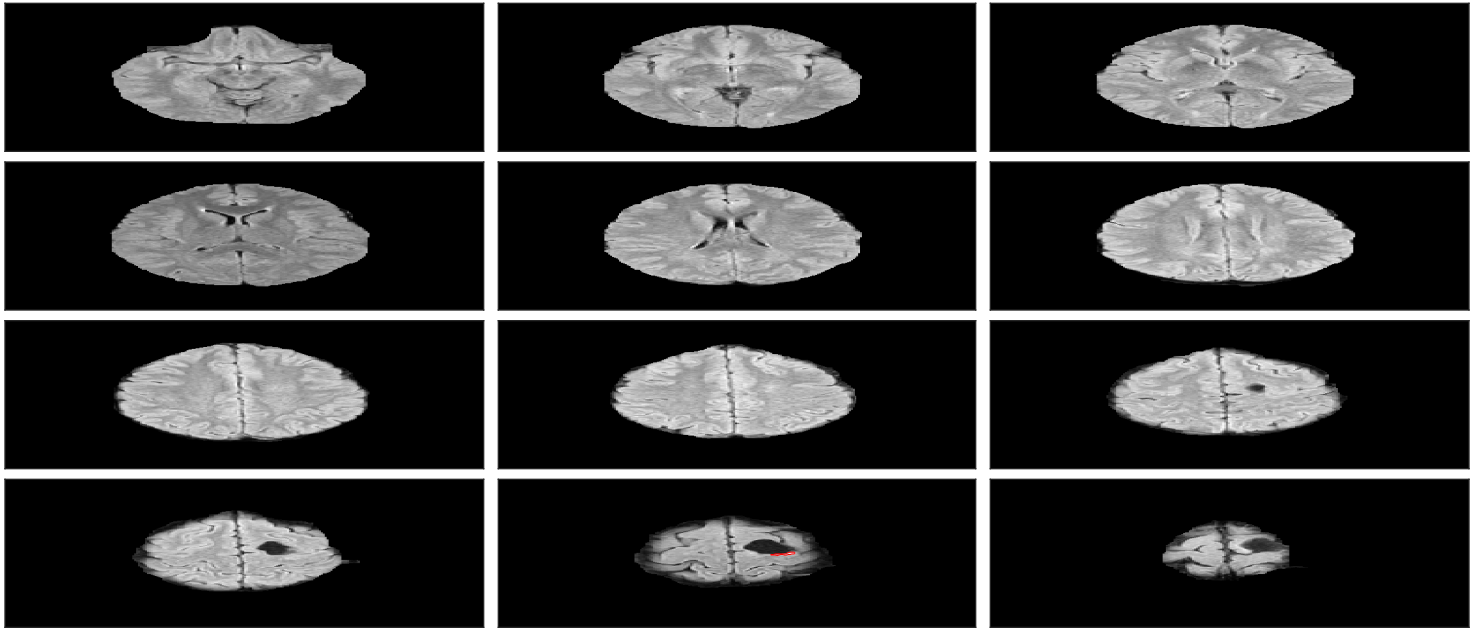

Supplement: S1 Data — The .zip file includes .pdf files labeled as follows, using case number 4224 as an example: 4224_t0.pdf includes the MRI data with segmentation of case number 4224 at baseline. 4224_t1_CAD_G_VC_S.pdf includes the MRI data with segmentation of case number 4224 at time 1, i.e., the time point of growth detection by the change-of-point method when the time point was different from the most recent MRI. 4224_tend_CAD_G_VC_G.pdf includes the MRI data with segmentation of case number 4224 at the most recent MRI. CAD_G and CAD_S mean that the change-of-point method predicted growth (G) or stability (S), respectively. VC_G and VC_S mean that the visual comparison detected growth (G) or stability (S), respectively. (ZIP) [file pmed.1002810.s001.zip › 7715_t0.pdf]

Case Number:4386; TIME 1; CAD --> GROWTH; VC --> STABLE; Interval From Baseline =11 months.

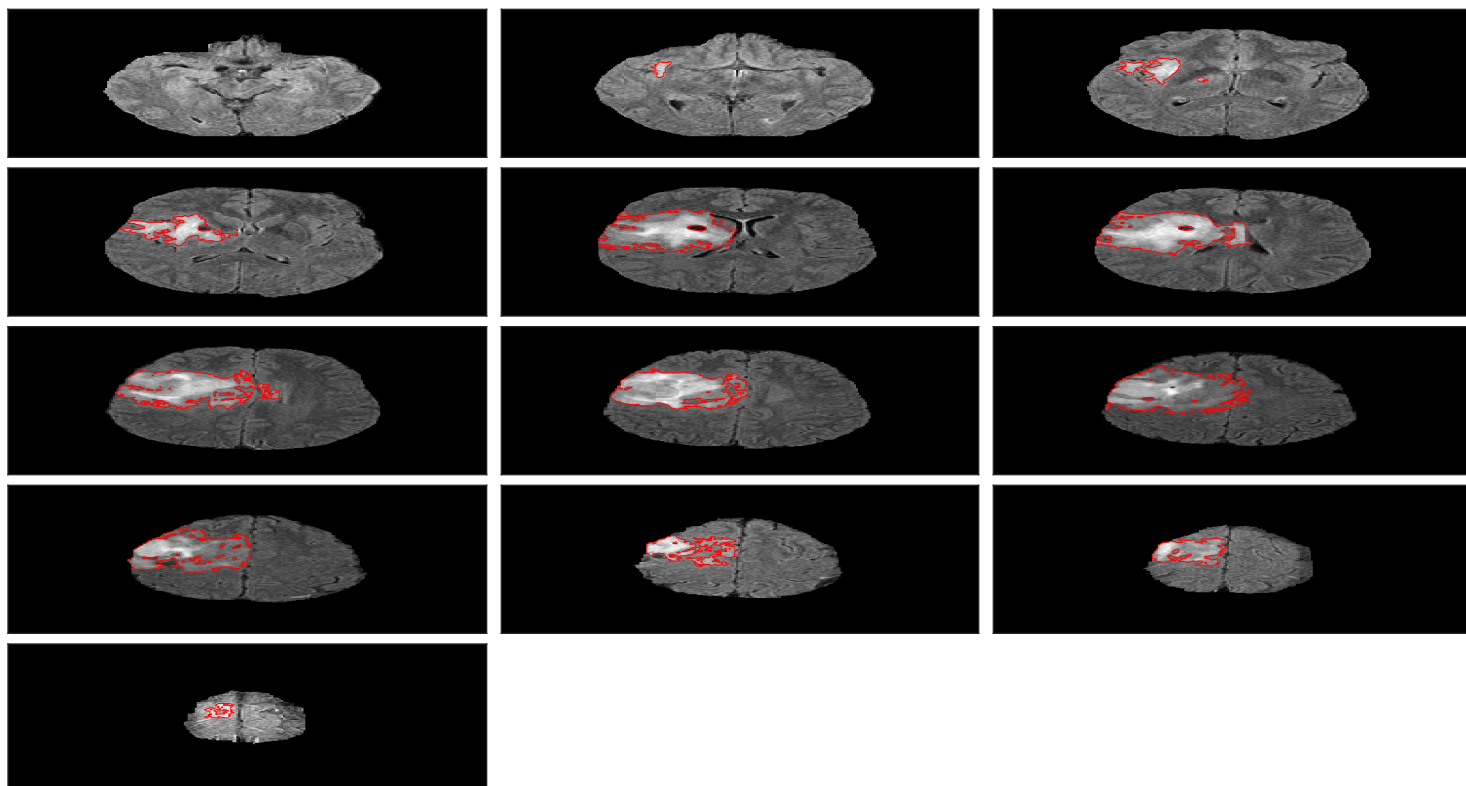

Supplement: S1 Data — The .zip file includes .pdf files labeled as follows, using case number 4224 as an example: 4224_t0.pdf includes the MRI data with segmentation of case number 4224 at baseline. 4224_t1_CAD_G_VC_S.pdf includes the MRI data with segmentation of case number 4224 at time 1, i.e., the time point of growth detection by the change-of-point method when the time point was different from the most recent MRI. 4224_tend_CAD_G_VC_G.pdf includes the MRI data with segmentation of case number 4224 at the most recent MRI. CAD_G and CAD_S mean that the change-of-point method predicted growth (G) or stability (S), respectively. VC_G and VC_S mean that the visual comparison detected growth (G) or stability (S), respectively. (ZIP) [file pmed.1002810.s001.zip › 4386_t1_CAD_G_VC_S.pdf]

Case Number:7494; Time 0 (Baseline).

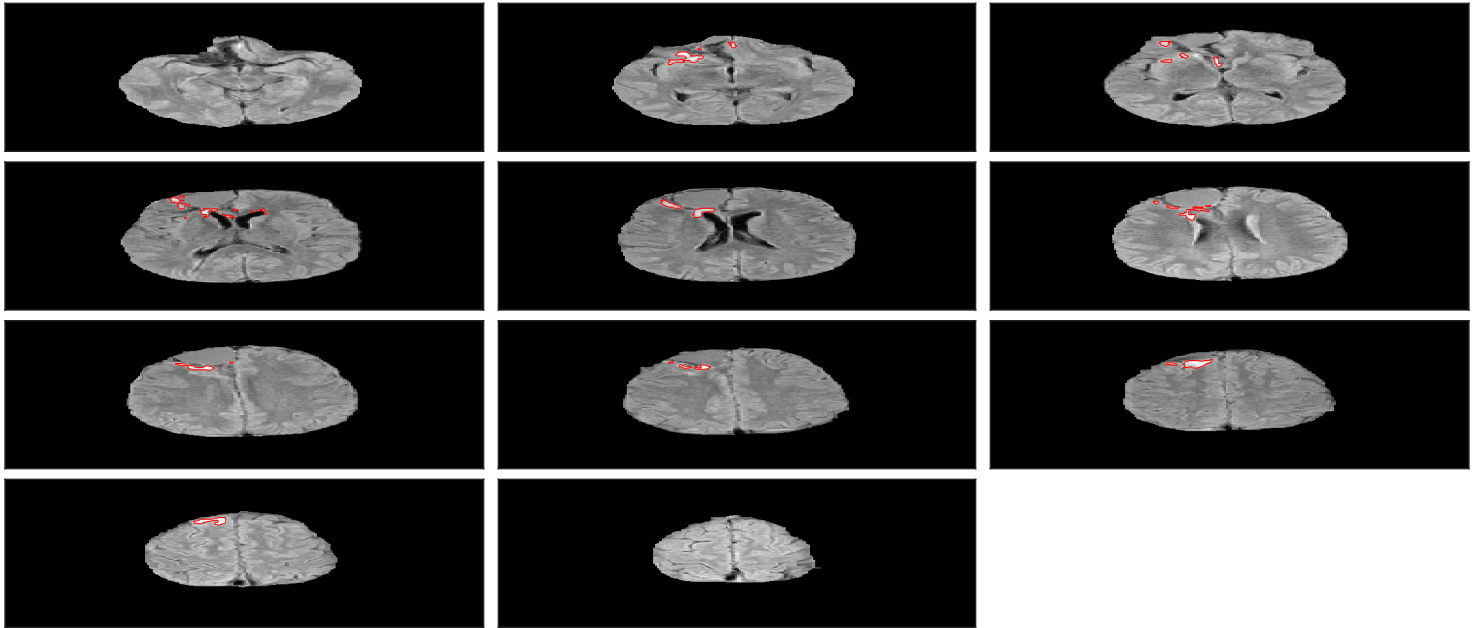

Supplement: S1 Data — The .zip file includes .pdf files labeled as follows, using case number 4224 as an example: 4224_t0.pdf includes the MRI data with segmentation of case number 4224 at baseline. 4224_t1_CAD_G_VC_S.pdf includes the MRI data with segmentation of case number 4224 at time 1, i.e., the time point of growth detection by the change-of-point method when the time point was different from the most recent MRI. 4224_tend_CAD_G_VC_G.pdf includes the MRI data with segmentation of case number 4224 at the most recent MRI. CAD_G and CAD_S mean that the change-of-point method predicted growth (G) or stability (S), respectively. VC_G and VC_S mean that the visual comparison detected growth (G) or stability (S), respectively. (ZIP) [file pmed.1002810.s001.zip › 7494_t0.pdf]

Case Number:4389; Time 0 (Baseline).

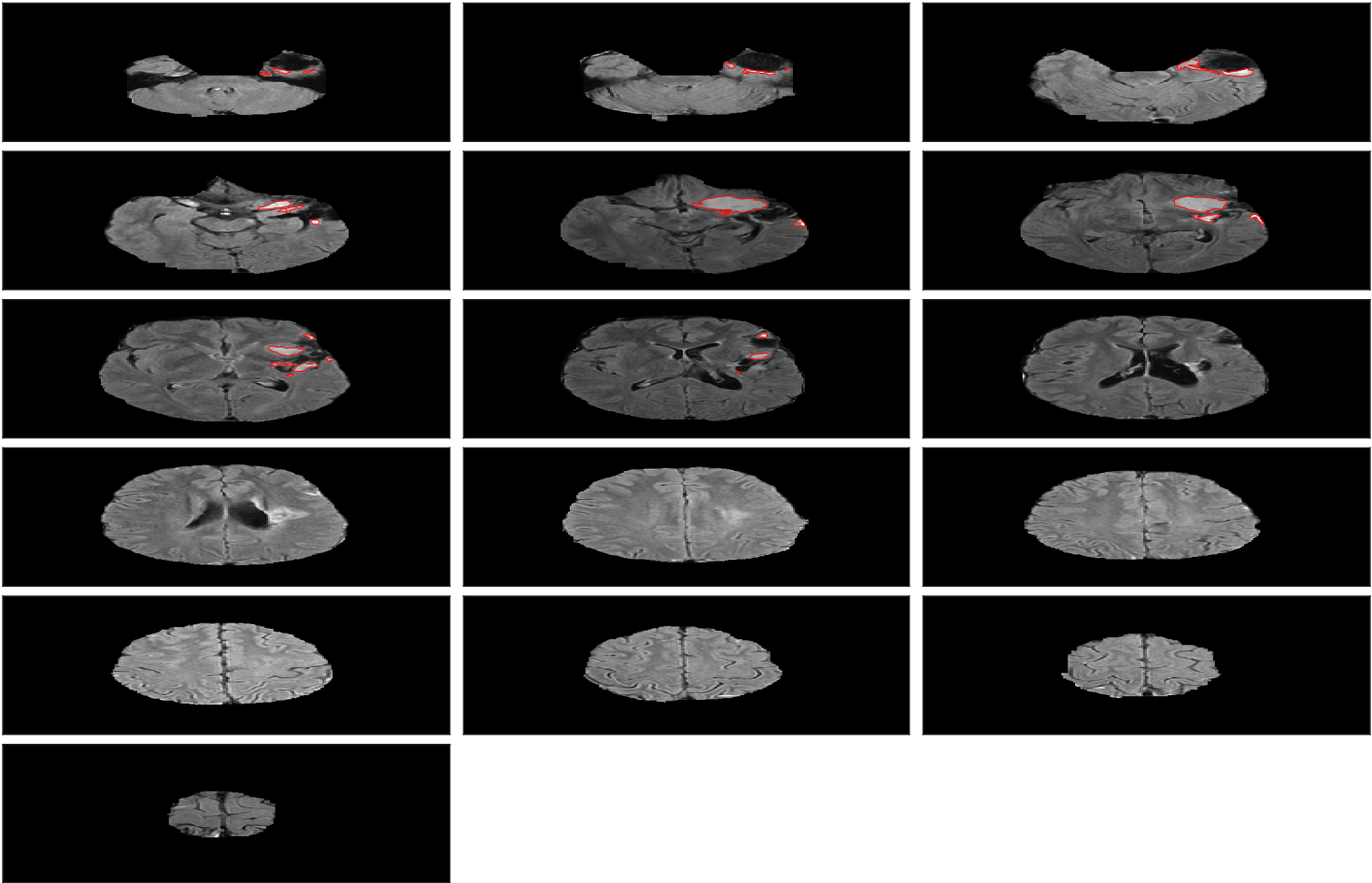

Supplement: S1 Data — The .zip file includes .pdf files labeled as follows, using case number 4224 as an example: 4224_t0.pdf includes the MRI data with segmentation of case number 4224 at baseline. 4224_t1_CAD_G_VC_S.pdf includes the MRI data with segmentation of case number 4224 at time 1, i.e., the time point of growth detection by the change-of-point method when the time point was different from the most recent MRI. 4224_tend_CAD_G_VC_G.pdf includes the MRI data with segmentation of case number 4224 at the most recent MRI. CAD_G and CAD_S mean that the change-of-point method predicted growth (G) or stability (S), respectively. VC_G and VC_S mean that the visual comparison detected growth (G) or stability (S), respectively. (ZIP) [file pmed.1002810.s001.zip › 4389_t0.pdf]

Case Number:7752; Time 0 (Baseline).

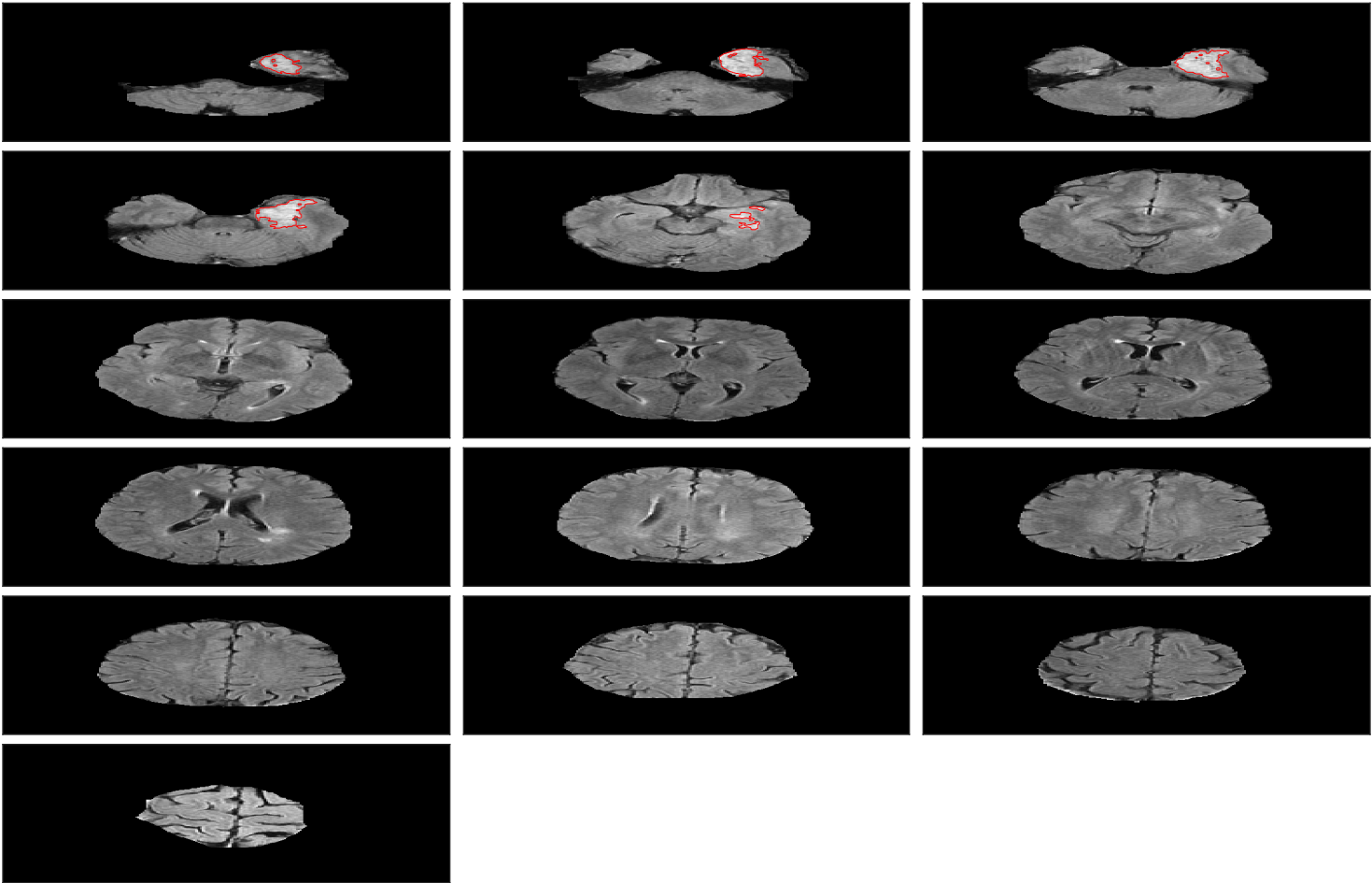

Supplement: S1 Data — The .zip file includes .pdf files labeled as follows, using case number 4224 as an example: 4224_t0.pdf includes the MRI data with segmentation of case number 4224 at baseline. 4224_t1_CAD_G_VC_S.pdf includes the MRI data with segmentation of case number 4224 at time 1, i.e., the time point of growth detection by the change-of-point method when the time point was different from the most recent MRI. 4224_tend_CAD_G_VC_G.pdf includes the MRI data with segmentation of case number 4224 at the most recent MRI. CAD_G and CAD_S mean that the change-of-point method predicted growth (G) or stability (S), respectively. VC_G and VC_S mean that the visual comparison detected growth (G) or stability (S), respectively. (ZIP) [file pmed.1002810.s001.zip › 7752_t0.pdf]

Case Number:7336; LAST MRI; CAD --> GROWTH; VC --> GROWTH; Interval From Baseline =34 months.

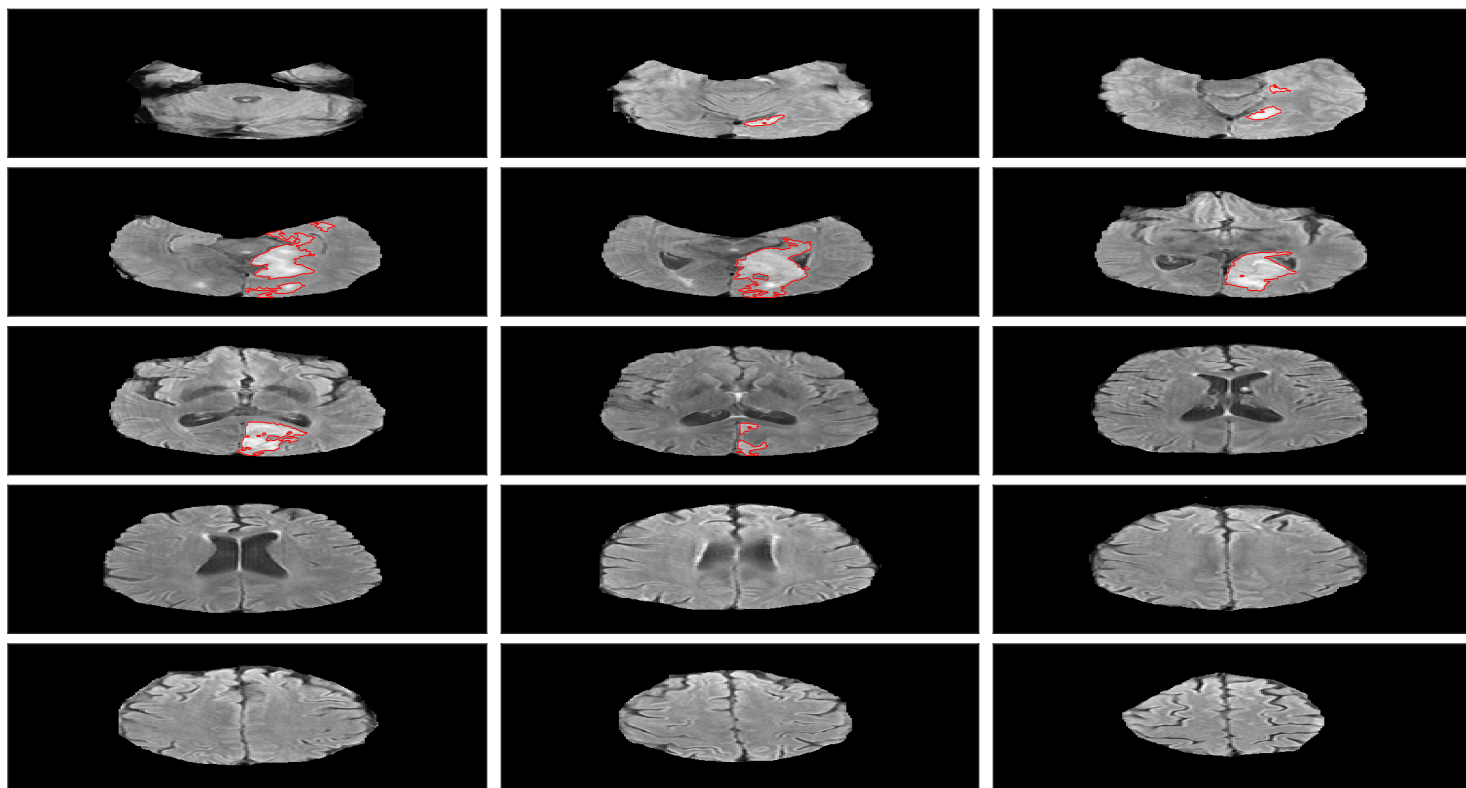

Supplement: S1 Data — The .zip file includes .pdf files labeled as follows, using case number 4224 as an example: 4224_t0.pdf includes the MRI data with segmentation of case number 4224 at baseline. 4224_t1_CAD_G_VC_S.pdf includes the MRI data with segmentation of case number 4224 at time 1, i.e., the time point of growth detection by the change-of-point method when the time point was different from the most recent MRI. 4224_tend_CAD_G_VC_G.pdf includes the MRI data with segmentation of case number 4224 at the most recent MRI. CAD_G and CAD_S mean that the change-of-point method predicted growth (G) or stability (S), respectively. VC_G and VC_S mean that the visual comparison detected growth (G) or stability (S), respectively. (ZIP) [file pmed.1002810.s001.zip › 7336_tend_CAD_G_VC_G.pdf]

Case Number:4385; Time 0 (Baseline).

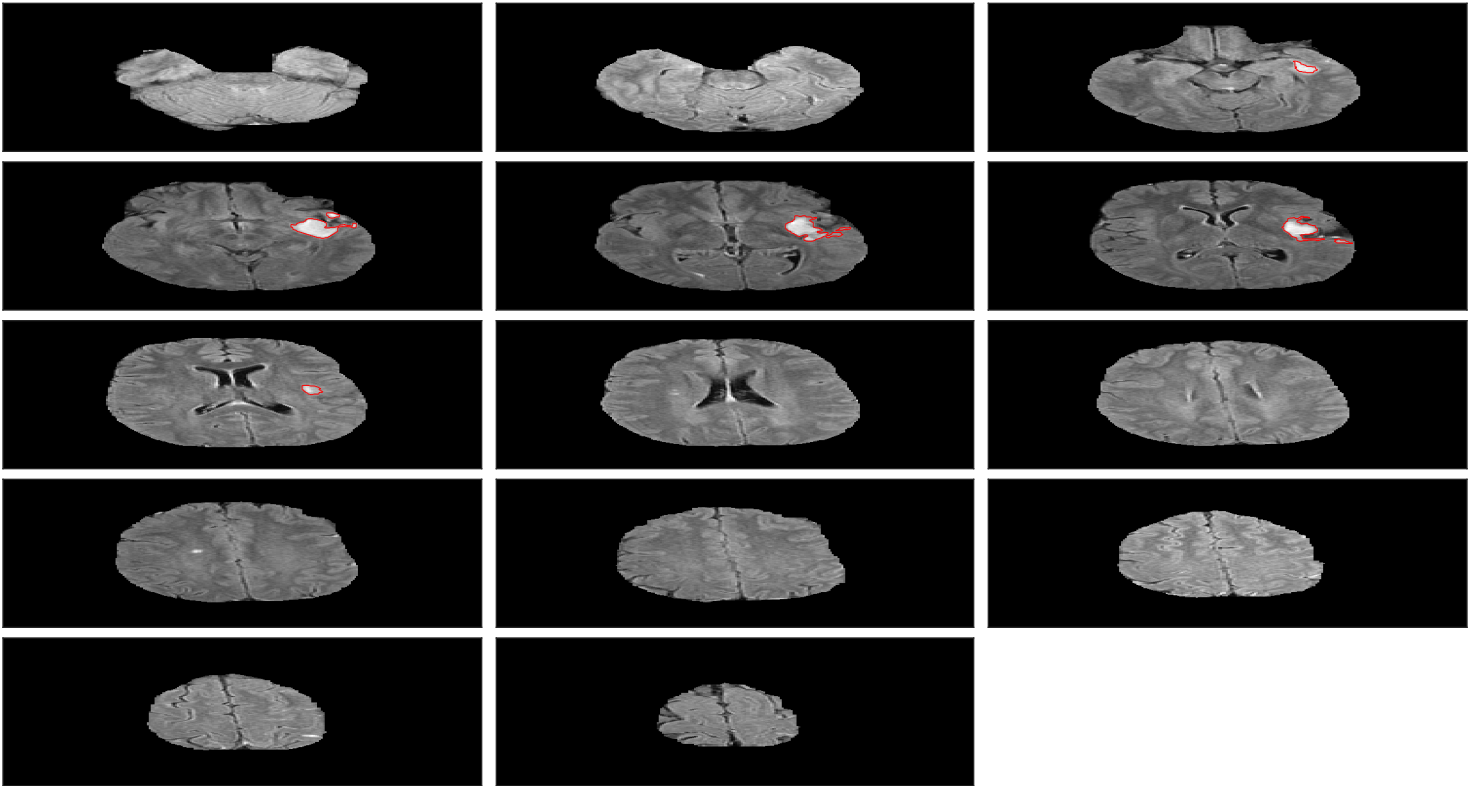

Supplement: S1 Data — The .zip file includes .pdf files labeled as follows, using case number 4224 as an example: 4224_t0.pdf includes the MRI data with segmentation of case number 4224 at baseline. 4224_t1_CAD_G_VC_S.pdf includes the MRI data with segmentation of case number 4224 at time 1, i.e., the time point of growth detection by the change-of-point method when the time point was different from the most recent MRI. 4224_tend_CAD_G_VC_G.pdf includes the MRI data with segmentation of case number 4224 at the most recent MRI. CAD_G and CAD_S mean that the change-of-point method predicted growth (G) or stability (S), respectively. VC_G and VC_S mean that the visual comparison detected growth (G) or stability (S), respectively. (ZIP) [file pmed.1002810.s001.zip › 4385_t0.pdf]

Case Number:4389; LAST MRI; CAD --> GROWTH; VC --> GROWTH; Interval From Baseline =26 months.

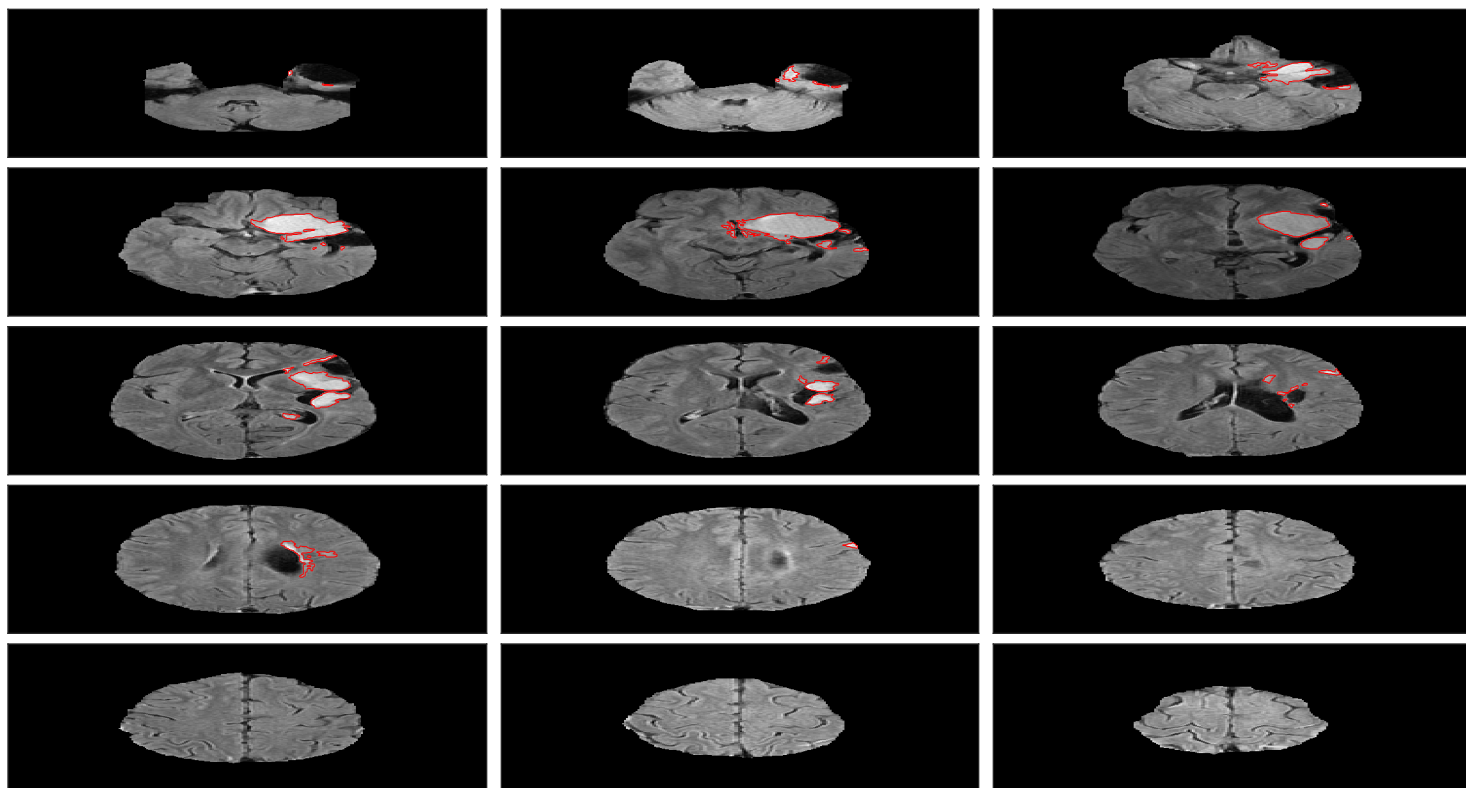

Supplement: S1 Data — The .zip file includes .pdf files labeled as follows, using case number 4224 as an example: 4224_t0.pdf includes the MRI data with segmentation of case number 4224 at baseline. 4224_t1_CAD_G_VC_S.pdf includes the MRI data with segmentation of case number 4224 at time 1, i.e., the time point of growth detection by the change-of-point method when the time point was different from the most recent MRI. 4224_tend_CAD_G_VC_G.pdf includes the MRI data with segmentation of case number 4224 at the most recent MRI. CAD_G and CAD_S mean that the change-of-point method predicted growth (G) or stability (S), respectively. VC_G and VC_S mean that the visual comparison detected growth (G) or stability (S), respectively. (ZIP) [file pmed.1002810.s001.zip › 4389_tend_CAD_G_VC_G.pdf]

**Case Number:7335; LAST MRI; CAD --> GROWTH; VC --> GROWTH; Interval From Baseline =34 months.**

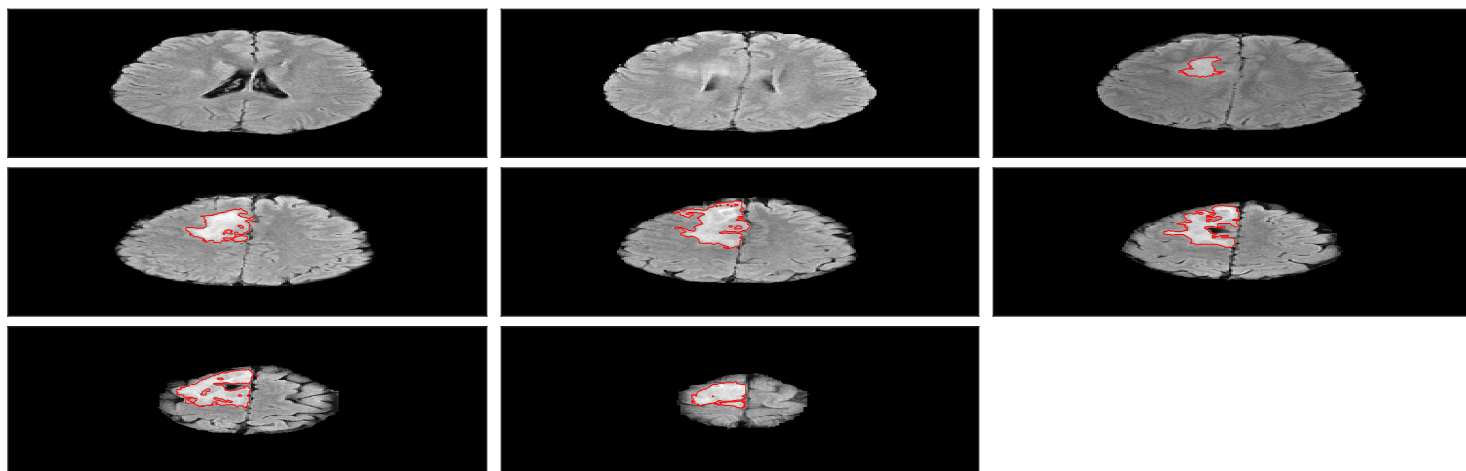

Supplement: S1 Data — The .zip file includes .pdf files labeled as follows, using case number 4224 as an example: 4224_t0.pdf includes the MRI data with segmentation of case number 4224 at baseline. 4224_t1_CAD_G_VC_S.pdf includes the MRI data with segmentation of case number 4224 at time 1, i.e., the time point of growth detection by the change-of-point method when the time point was different from the most recent MRI. 4224_tend_CAD_G_VC_G.pdf includes the MRI data with segmentation of case number 4224 at the most recent MRI. CAD_G and CAD_S mean that the change-of-point method predicted growth (G) or stability (S), respectively. VC_G and VC_S mean that the visual comparison detected growth (G) or stability (S), respectively. (ZIP) [file pmed.1002810.s001.zip › 7335_tend_CAD_G_VC_G.pdf]

Case Number:7492; TIME 1; CAD --> GROWTH; VC --> STABLE; Interval From Baseline =9 months.

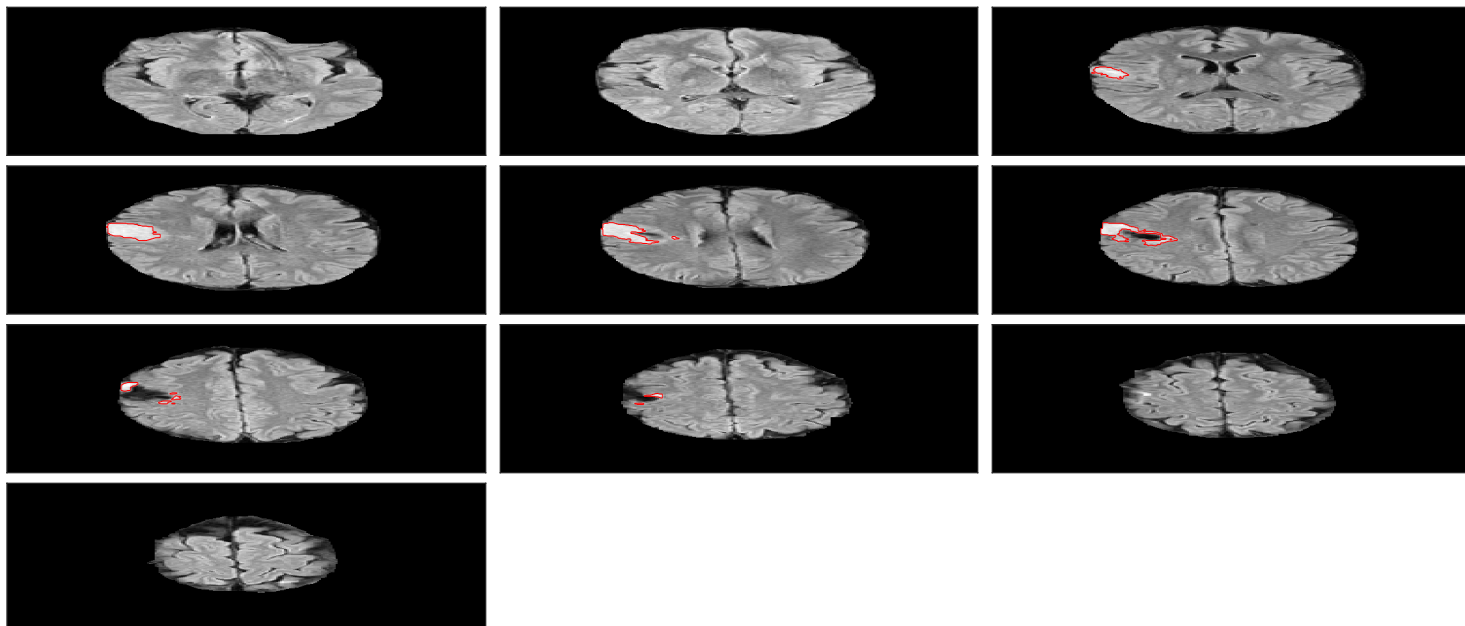

Supplement: S1 Data — The .zip file includes .pdf files labeled as follows, using case number 4224 as an example: 4224_t0.pdf includes the MRI data with segmentation of case number 4224 at baseline. 4224_t1_CAD_G_VC_S.pdf includes the MRI data with segmentation of case number 4224 at time 1, i.e., the time point of growth detection by the change-of-point method when the time point was different from the most recent MRI. 4224_tend_CAD_G_VC_G.pdf includes the MRI data with segmentation of case number 4224 at the most recent MRI. CAD_G and CAD_S mean that the change-of-point method predicted growth (G) or stability (S), respectively. VC_G and VC_S mean that the visual comparison detected growth (G) or stability (S), respectively. (ZIP) [file pmed.1002810.s001.zip › 7492_t1_CAD_G_VC_S.pdf]

Case Number:4388; LAST MRI; CAD --> GROWTH; VC --> GROWTH; Interval From Baseline =14 months.

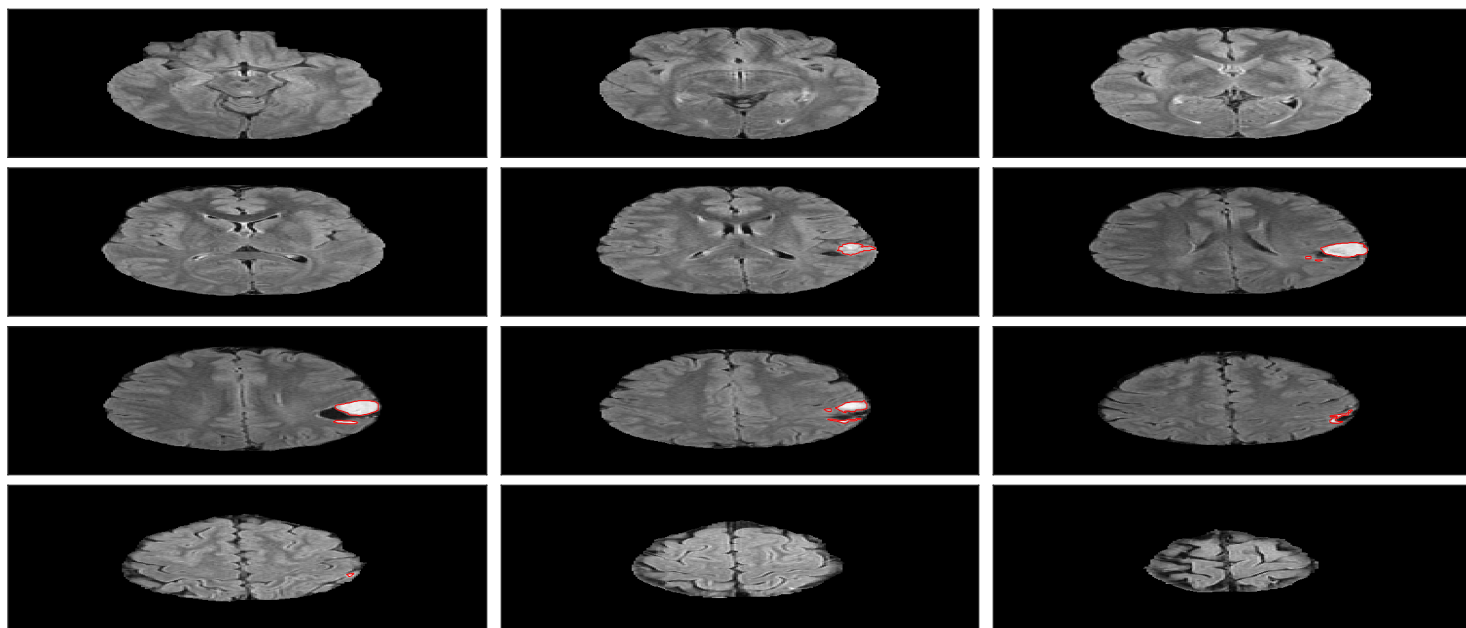

Supplement: S1 Data — The .zip file includes .pdf files labeled as follows, using case number 4224 as an example: 4224_t0.pdf includes the MRI data with segmentation of case number 4224 at baseline. 4224_t1_CAD_G_VC_S.pdf includes the MRI data with segmentation of case number 4224 at time 1, i.e., the time point of growth detection by the change-of-point method when the time point was different from the most recent MRI. 4224_tend_CAD_G_VC_G.pdf includes the MRI data with segmentation of case number 4224 at the most recent MRI. CAD_G and CAD_S mean that the change-of-point method predicted growth (G) or stability (S), respectively. VC_G and VC_S mean that the visual comparison detected growth (G) or stability (S), respectively. (ZIP) [file pmed.1002810.s001.zip › 4388_tend_CAD_G_VC_G.pdf]

Case Number:7709; TIME 1; CAD --> GROWTH; VC --> STABLE; Interval From Baseline =14 months.

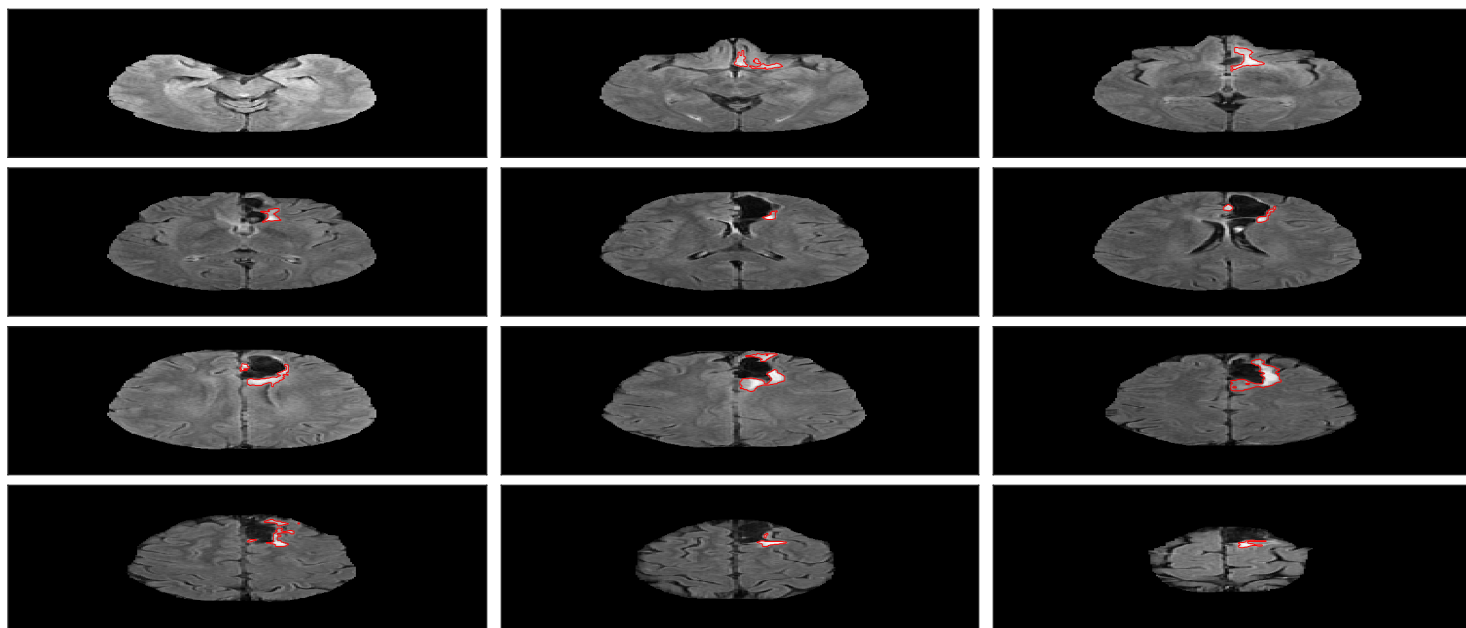

Supplement: S1 Data — The .zip file includes .pdf files labeled as follows, using case number 4224 as an example: 4224_t0.pdf includes the MRI data with segmentation of case number 4224 at baseline. 4224_t1_CAD_G_VC_S.pdf includes the MRI data with segmentation of case number 4224 at time 1, i.e., the time point of growth detection by the change-of-point method when the time point was different from the most recent MRI. 4224_tend_CAD_G_VC_G.pdf includes the MRI data with segmentation of case number 4224 at the most recent MRI. CAD_G and CAD_S mean that the change-of-point method predicted growth (G) or stability (S), respectively. VC_G and VC_S mean that the visual comparison detected growth (G) or stability (S), respectively. (ZIP) [file pmed.1002810.s001.zip › 7709_t1_CAD_G_VC_S.pdf]

Case Number:7334; LAST MRI; CAD --> GROWTH; VC --> GROWTH; Interval From Baseline =11 months.

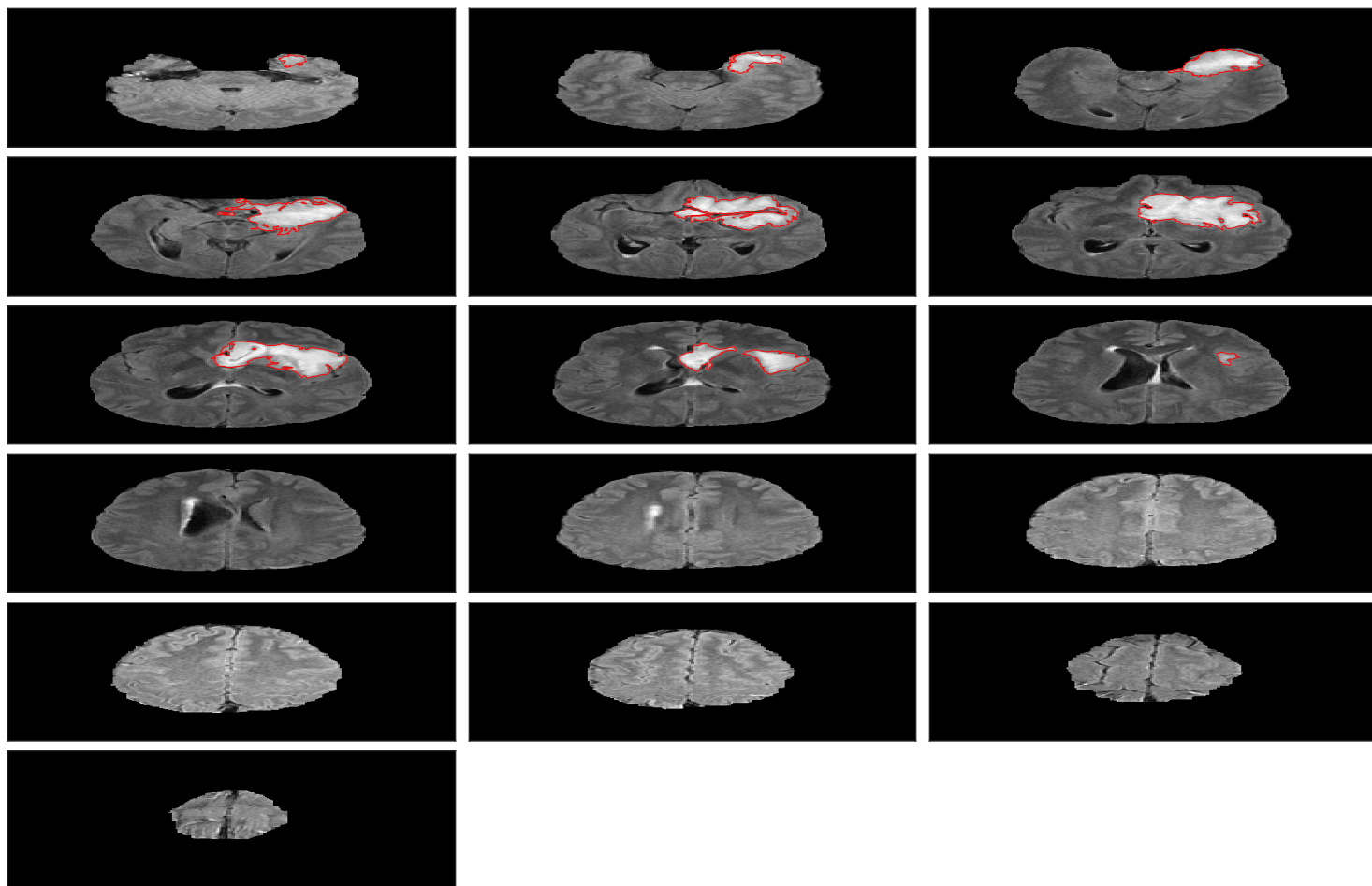

Supplement: S1 Data — The .zip file includes .pdf files labeled as follows, using case number 4224 as an example: 4224_t0.pdf includes the MRI data with segmentation of case number 4224 at baseline. 4224_t1_CAD_G_VC_S.pdf includes the MRI data with segmentation of case number 4224 at time 1, i.e., the time point of growth detection by the change-of-point method when the time point was different from the most recent MRI. 4224_tend_CAD_G_VC_G.pdf includes the MRI data with segmentation of case number 4224 at the most recent MRI. CAD_G and CAD_S mean that the change-of-point method predicted growth (G) or stability (S), respectively. VC_G and VC_S mean that the visual comparison detected growth (G) or stability (S), respectively. (ZIP) [file pmed.1002810.s001.zip › 7334_tend_CAD_G_VC_G.pdf]

Case Number:7504; Time 0 (Baseline).

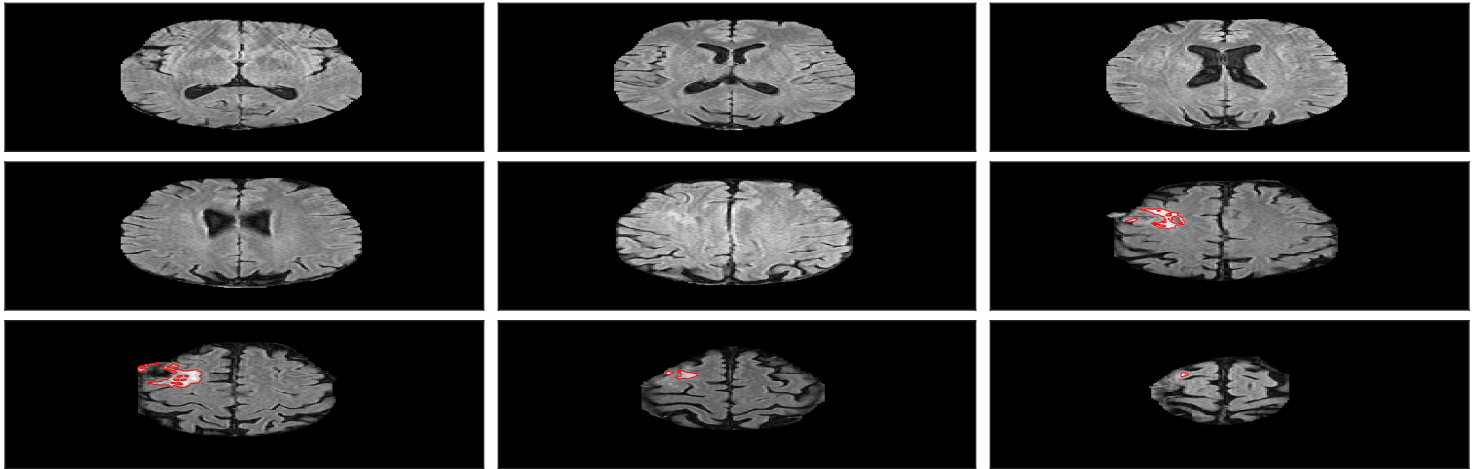

Supplement: S1 Data — The .zip file includes .pdf files labeled as follows, using case number 4224 as an example: 4224_t0.pdf includes the MRI data with segmentation of case number 4224 at baseline. 4224_t1_CAD_G_VC_S.pdf includes the MRI data with segmentation of case number 4224 at time 1, i.e., the time point of growth detection by the change-of-point method when the time point was different from the most recent MRI. 4224_tend_CAD_G_VC_G.pdf includes the MRI data with segmentation of case number 4224 at the most recent MRI. CAD_G and CAD_S mean that the change-of-point method predicted growth (G) or stability (S), respectively. VC_G and VC_S mean that the visual comparison detected growth (G) or stability (S), respectively. (ZIP) [file pmed.1002810.s001.zip › 7504_t0.pdf]

Case Number:7336; Time 0 (Baseline).

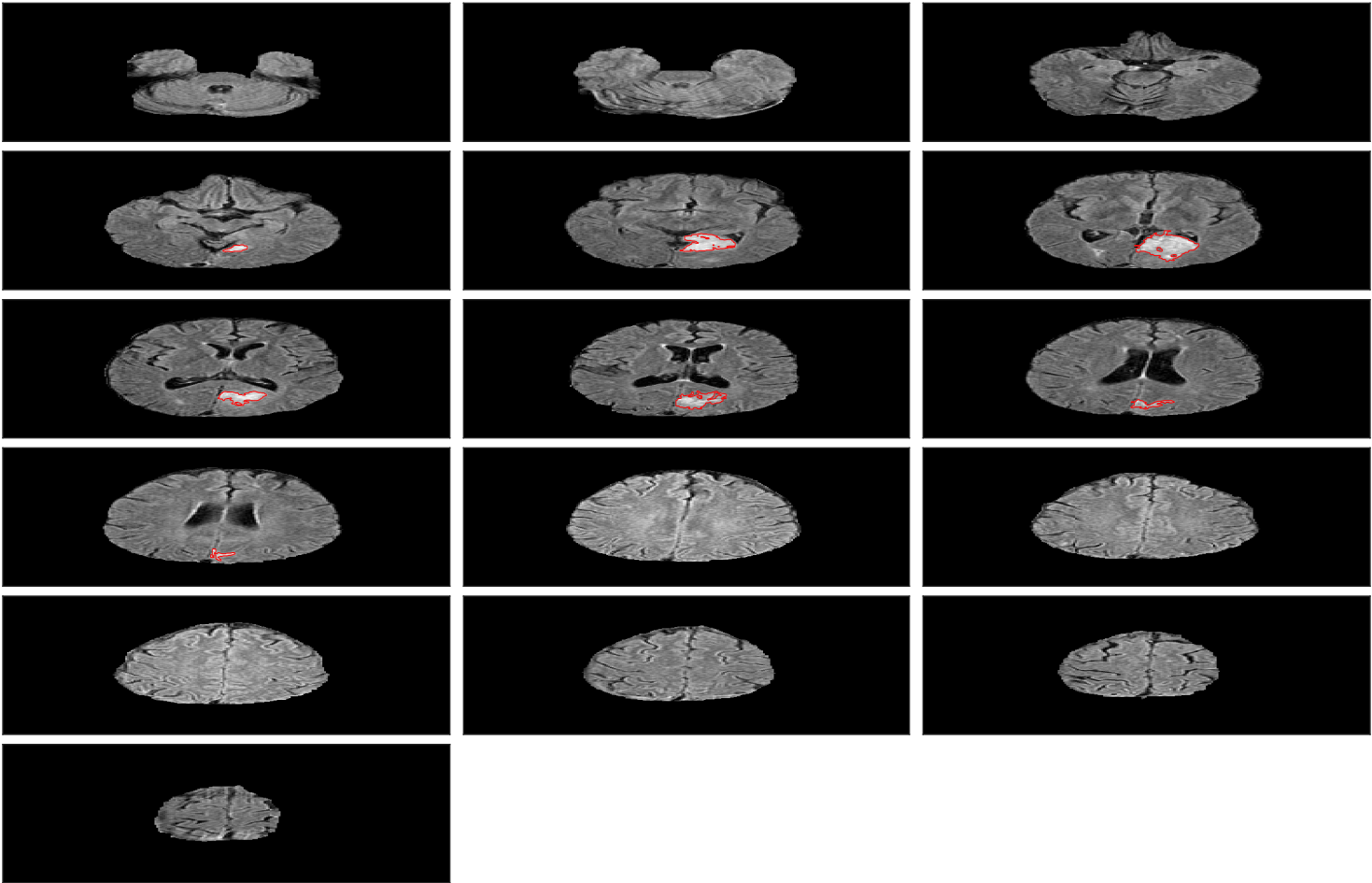

Supplement: S1 Data — The .zip file includes .pdf files labeled as follows, using case number 4224 as an example: 4224_t0.pdf includes the MRI data with segmentation of case number 4224 at baseline. 4224_t1_CAD_G_VC_S.pdf includes the MRI data with segmentation of case number 4224 at time 1, i.e., the time point of growth detection by the change-of-point method when the time point was different from the most recent MRI. 4224_tend_CAD_G_VC_G.pdf includes the MRI data with segmentation of case number 4224 at the most recent MRI. CAD_G and CAD_S mean that the change-of-point method predicted growth (G) or stability (S), respectively. VC_G and VC_S mean that the visual comparison detected growth (G) or stability (S), respectively. (ZIP) [file pmed.1002810.s001.zip › 7336_t0.pdf]

Case Number:7476; TIME 1; CAD --> GROWTH; VC --> STABLE; Interval From Baseline =21 months.

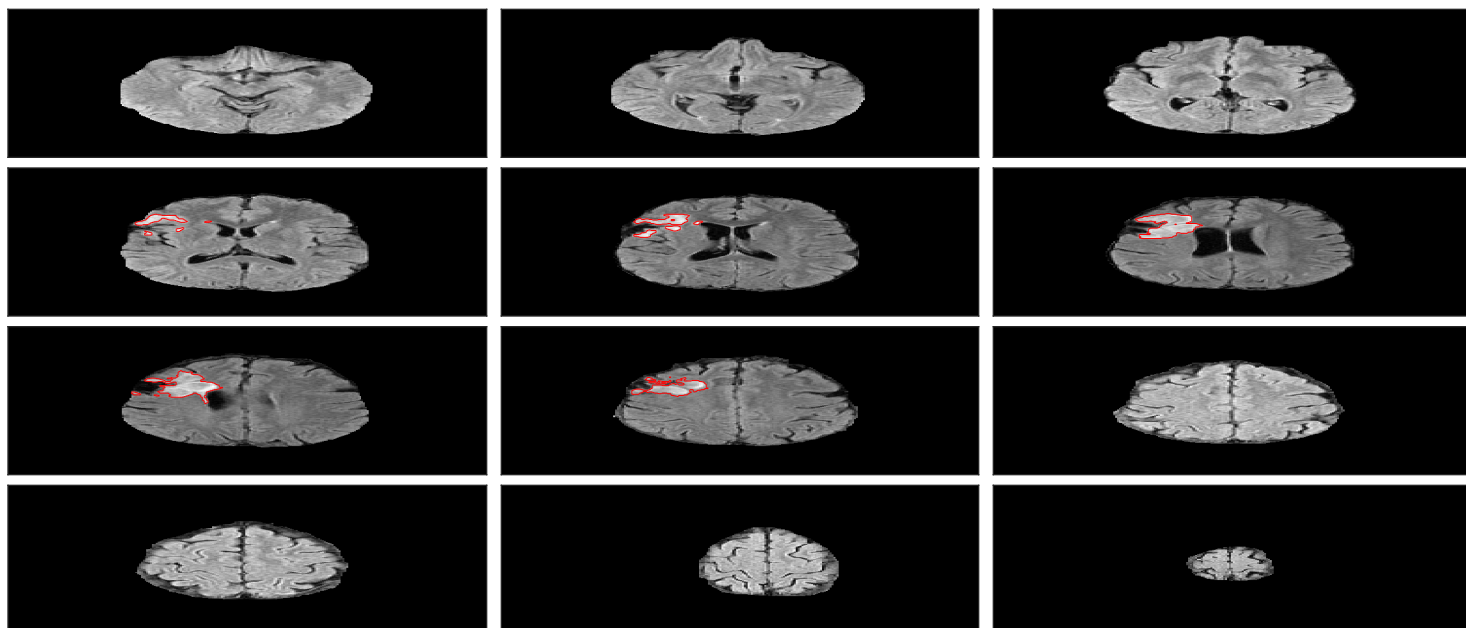

Supplement: S1 Data — The .zip file includes .pdf files labeled as follows, using case number 4224 as an example: 4224_t0.pdf includes the MRI data with segmentation of case number 4224 at baseline. 4224_t1_CAD_G_VC_S.pdf includes the MRI data with segmentation of case number 4224 at time 1, i.e., the time point of growth detection by the change-of-point method when the time point was different from the most recent MRI. 4224_tend_CAD_G_VC_G.pdf includes the MRI data with segmentation of case number 4224 at the most recent MRI. CAD_G and CAD_S mean that the change-of-point method predicted growth (G) or stability (S), respectively. VC_G and VC_S mean that the visual comparison detected growth (G) or stability (S), respectively. (ZIP) [file pmed.1002810.s001.zip › 7476_t1_CAD_G_VC_S.pdf]

Case Number:4387; TIME 1; CAD --> GROWTH; VC --> STABLE; Interval From Baseline =11 months.

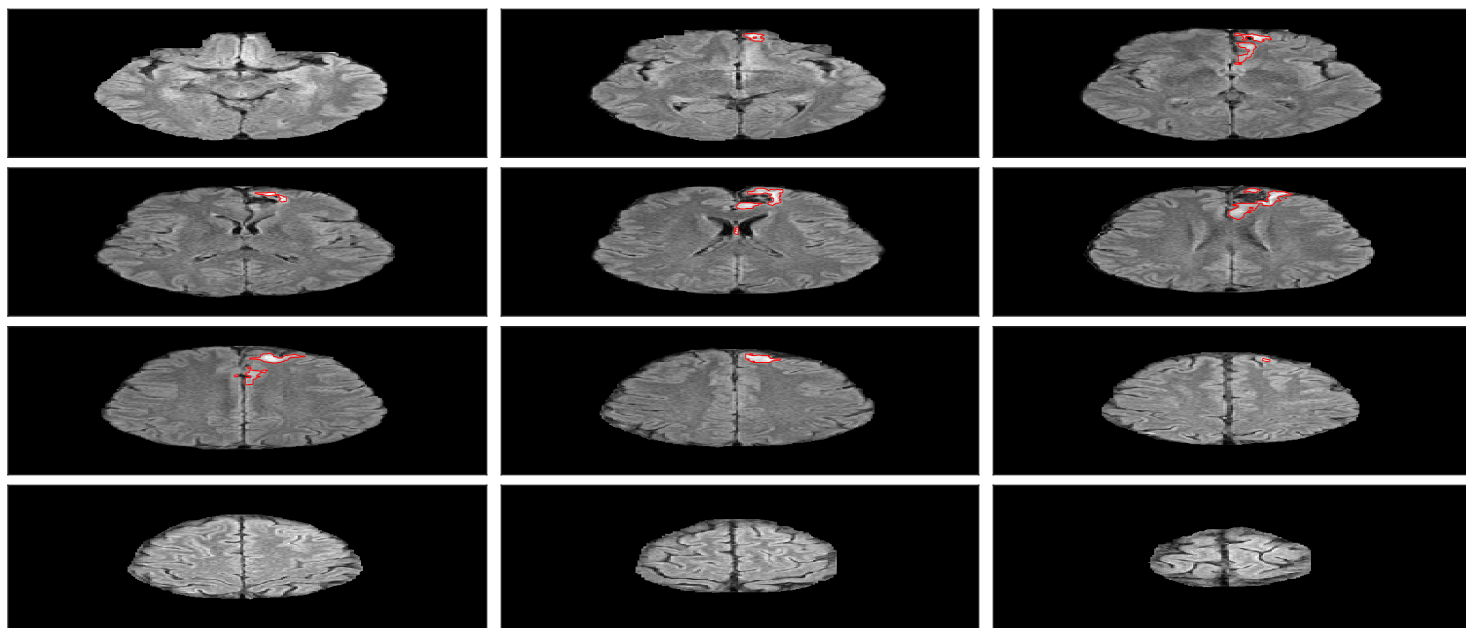

Supplement: S1 Data — The .zip file includes .pdf files labeled as follows, using case number 4224 as an example: 4224_t0.pdf includes the MRI data with segmentation of case number 4224 at baseline. 4224_t1_CAD_G_VC_S.pdf includes the MRI data with segmentation of case number 4224 at time 1, i.e., the time point of growth detection by the change-of-point method when the time point was different from the most recent MRI. 4224_tend_CAD_G_VC_G.pdf includes the MRI data with segmentation of case number 4224 at the most recent MRI. CAD_G and CAD_S mean that the change-of-point method predicted growth (G) or stability (S), respectively. VC_G and VC_S mean that the visual comparison detected growth (G) or stability (S), respectively. (ZIP) [file pmed.1002810.s001.zip › 4387_t1_CAD_G_VC_S.pdf]

Case Number:7334; Time 0 (Baseline).

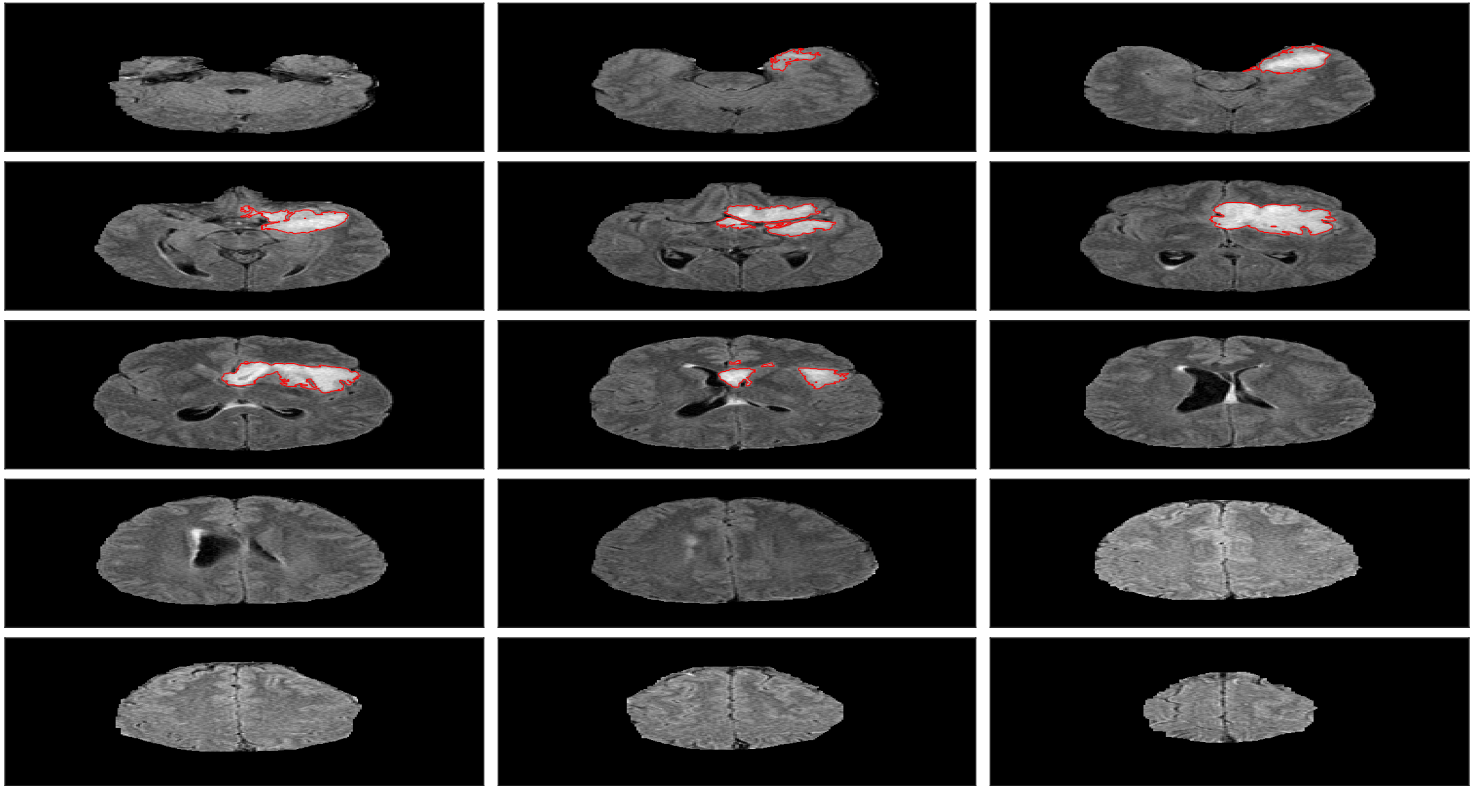

Supplement: S1 Data — The .zip file includes .pdf files labeled as follows, using case number 4224 as an example: 4224_t0.pdf includes the MRI data with segmentation of case number 4224 at baseline. 4224_t1_CAD_G_VC_S.pdf includes the MRI data with segmentation of case number 4224 at time 1, i.e., the time point of growth detection by the change-of-point method when the time point was different from the most recent MRI. 4224_tend_CAD_G_VC_G.pdf includes the MRI data with segmentation of case number 4224 at the most recent MRI. CAD_G and CAD_S mean that the change-of-point method predicted growth (G) or stability (S), respectively. VC_G and VC_S mean that the visual comparison detected growth (G) or stability (S), respectively. (ZIP) [file pmed.1002810.s001.zip › 7334_t0.pdf]

Case Number:7333; LAST MRI; CAD --> GROWTH; VC --> GROWTH; Interval From Baseline =44 months.

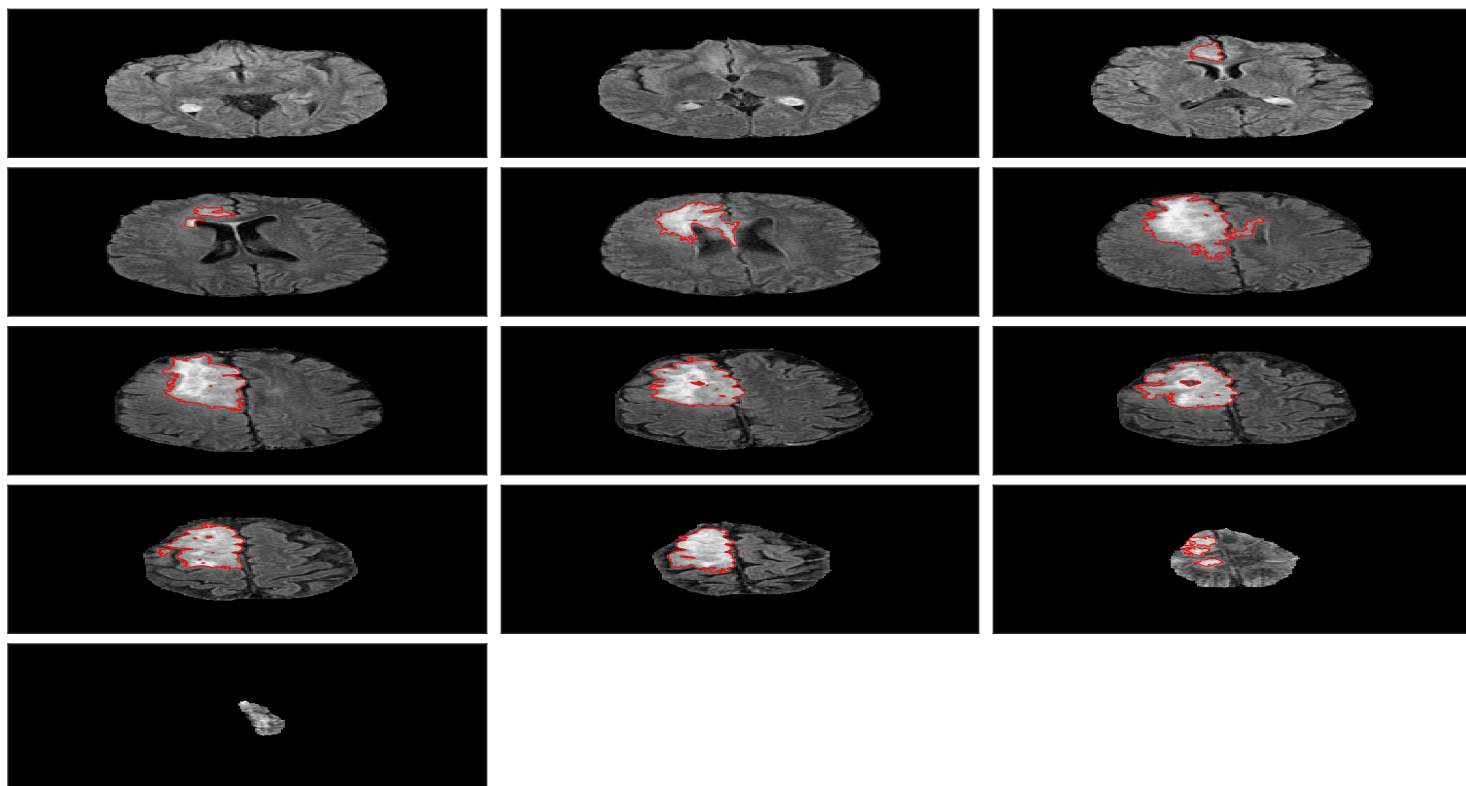

Supplement: S1 Data — The .zip file includes .pdf files labeled as follows, using case number 4224 as an example: 4224_t0.pdf includes the MRI data with segmentation of case number 4224 at baseline. 4224_t1_CAD_G_VC_S.pdf includes the MRI data with segmentation of case number 4224 at time 1, i.e., the time point of growth detection by the change-of-point method when the time point was different from the most recent MRI. 4224_tend_CAD_G_VC_G.pdf includes the MRI data with segmentation of case number 4224 at the most recent MRI. CAD_G and CAD_S mean that the change-of-point method predicted growth (G) or stability (S), respectively. VC_G and VC_S mean that the visual comparison detected growth (G) or stability (S), respectively. (ZIP) [file pmed.1002810.s001.zip › 7333_tend_CAD_G_VC_G.pdf]

Case Number:7506; Time 0 (Baseline).

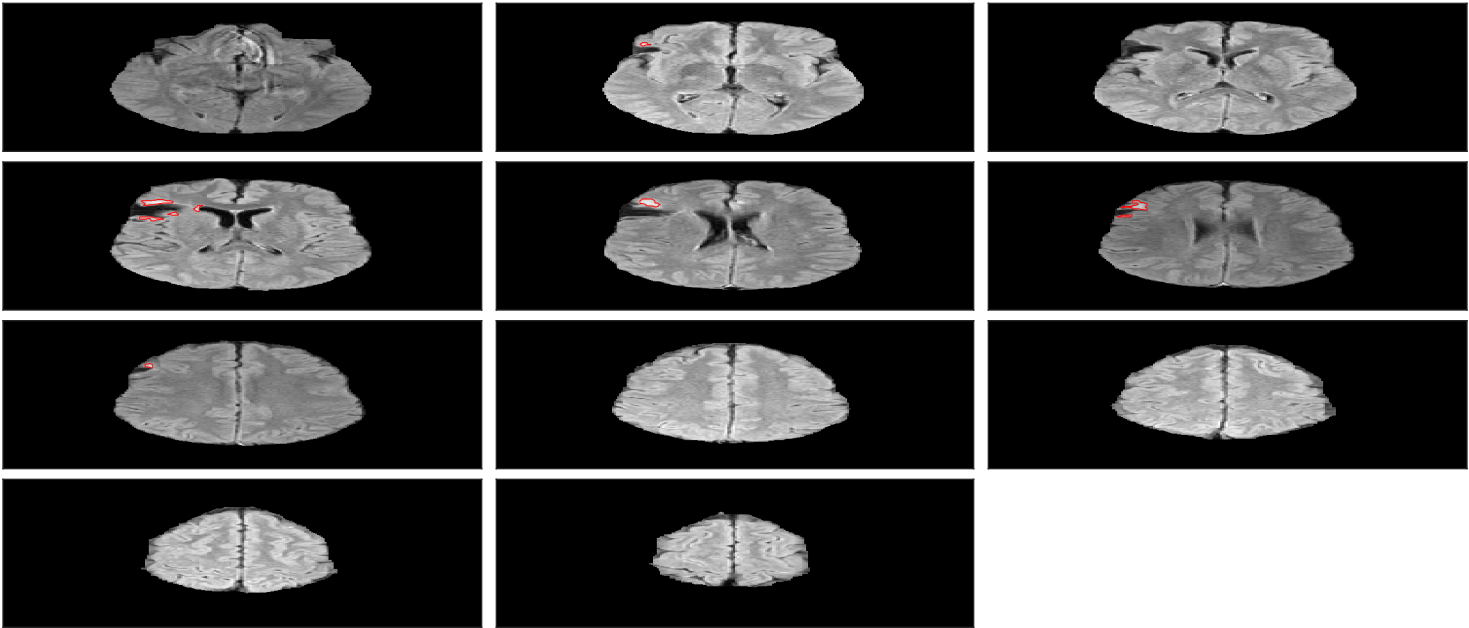

Supplement: S1 Data — The .zip file includes .pdf files labeled as follows, using case number 4224 as an example: 4224_t0.pdf includes the MRI data with segmentation of case number 4224 at baseline. 4224_t1_CAD_G_VC_S.pdf includes the MRI data with segmentation of case number 4224 at time 1, i.e., the time point of growth detection by the change-of-point method when the time point was different from the most recent MRI. 4224_tend_CAD_G_VC_G.pdf includes the MRI data with segmentation of case number 4224 at the most recent MRI. CAD_G and CAD_S mean that the change-of-point method predicted growth (G) or stability (S), respectively. VC_G and VC_S mean that the visual comparison detected growth (G) or stability (S), respectively. (ZIP) [file pmed.1002810.s001.zip › 7506_t0.pdf]

Case Number:7477; Time 0 (Baseline).

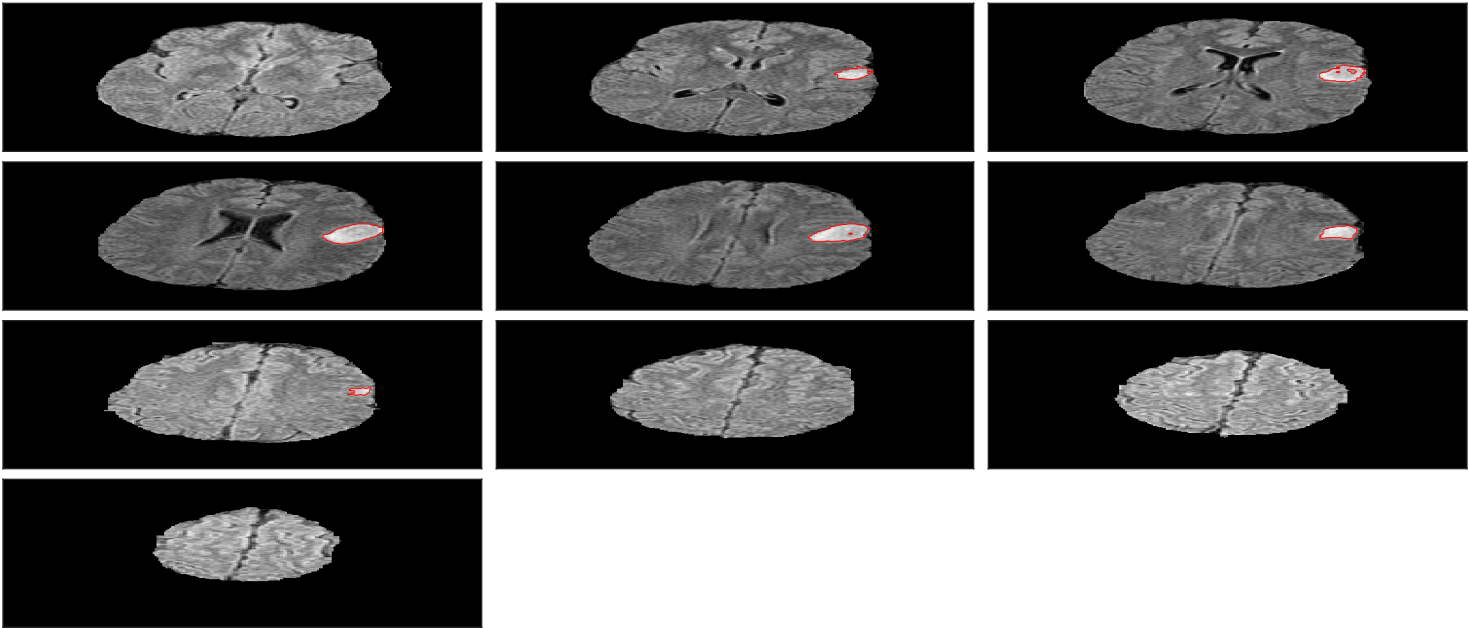

Supplement: S1 Data — The .zip file includes .pdf files labeled as follows, using case number 4224 as an example: 4224_t0.pdf includes the MRI data with segmentation of case number 4224 at baseline. 4224_t1_CAD_G_VC_S.pdf includes the MRI data with segmentation of case number 4224 at time 1, i.e., the time point of growth detection by the change-of-point method when the time point was different from the most recent MRI. 4224_tend_CAD_G_VC_G.pdf includes the MRI data with segmentation of case number 4224 at the most recent MRI. CAD_G and CAD_S mean that the change-of-point method predicted growth (G) or stability (S), respectively. VC_G and VC_S mean that the visual comparison detected growth (G) or stability (S), respectively. (ZIP) [file pmed.1002810.s001.zip › 7477_t0.pdf]

Case Number:7472; TIME 1; CAD --> GROWTH; VC --> STABLE; Interval From Baseline =16 months.

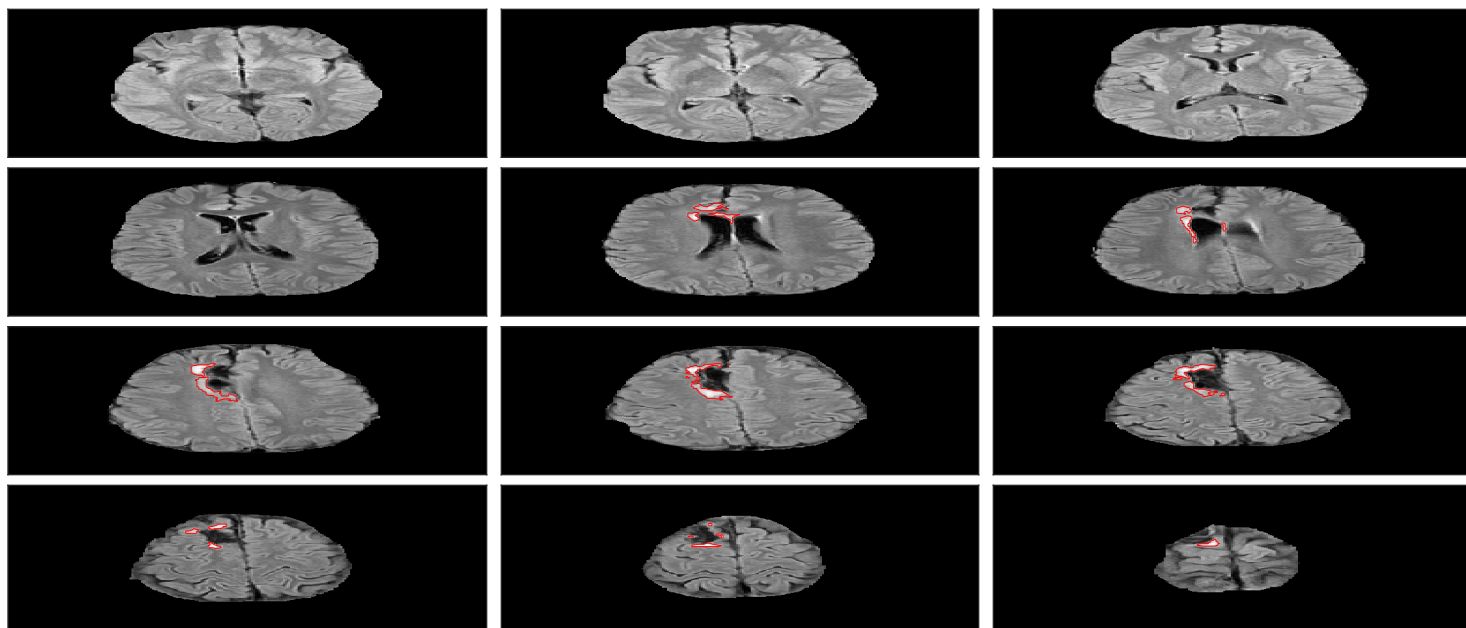

Supplement: S1 Data — The .zip file includes .pdf files labeled as follows, using case number 4224 as an example: 4224_t0.pdf includes the MRI data with segmentation of case number 4224 at baseline. 4224_t1_CAD_G_VC_S.pdf includes the MRI data with segmentation of case number 4224 at time 1, i.e., the time point of growth detection by the change-of-point method when the time point was different from the most recent MRI. 4224_tend_CAD_G_VC_G.pdf includes the MRI data with segmentation of case number 4224 at the most recent MRI. CAD_G and CAD_S mean that the change-of-point method predicted growth (G) or stability (S), respectively. VC_G and VC_S mean that the visual comparison detected growth (G) or stability (S), respectively. (ZIP) [file pmed.1002810.s001.zip › 7472_t1_CAD_G_VC_S.pdf]

Case Number:7469; Time 0 (Baseline).

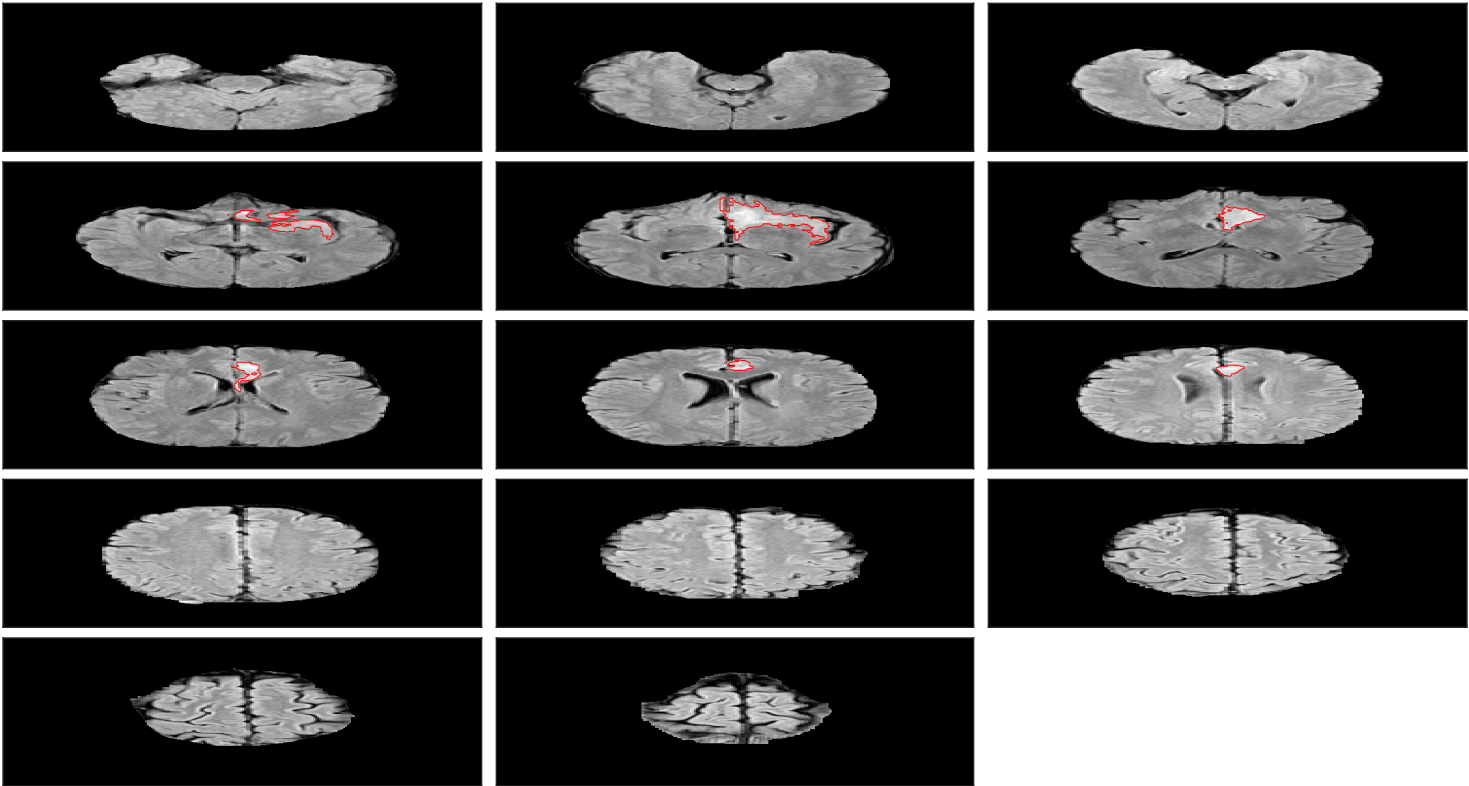

Supplement: S1 Data — The .zip file includes .pdf files labeled as follows, using case number 4224 as an example: 4224_t0.pdf includes the MRI data with segmentation of case number 4224 at baseline. 4224_t1_CAD_G_VC_S.pdf includes the MRI data with segmentation of case number 4224 at time 1, i.e., the time point of growth detection by the change-of-point method when the time point was different from the most recent MRI. 4224_tend_CAD_G_VC_G.pdf includes the MRI data with segmentation of case number 4224 at the most recent MRI. CAD_G and CAD_S mean that the change-of-point method predicted growth (G) or stability (S), respectively. VC_G and VC_S mean that the visual comparison detected growth (G) or stability (S), respectively. (ZIP) [file pmed.1002810.s001.zip › 7469_t0.pdf]

Case Number:7709; Time 0 (Baseline).

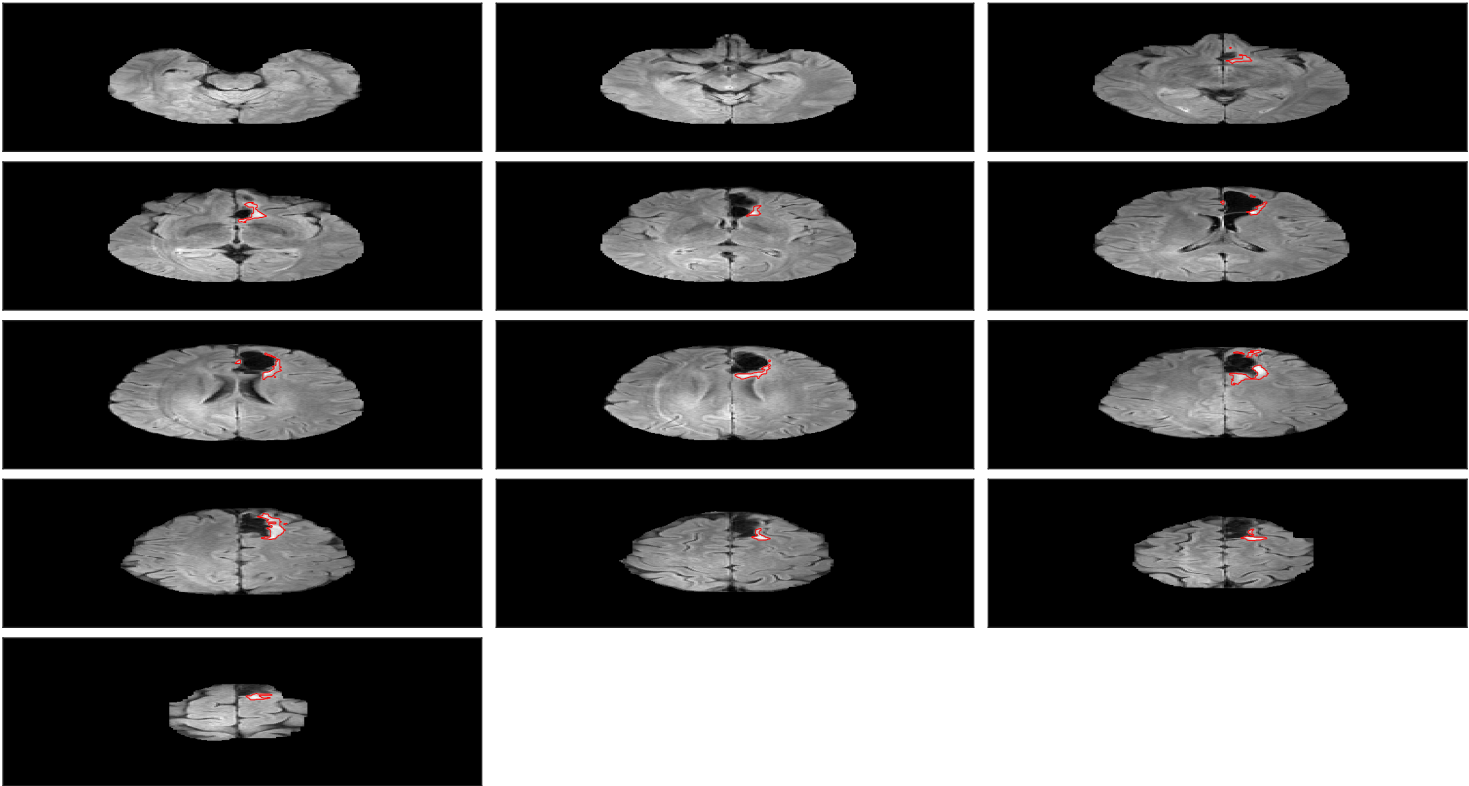

Supplement: S1 Data — The .zip file includes .pdf files labeled as follows, using case number 4224 as an example: 4224_t0.pdf includes the MRI data with segmentation of case number 4224 at baseline. 4224_t1_CAD_G_VC_S.pdf includes the MRI data with segmentation of case number 4224 at time 1, i.e., the time point of growth detection by the change-of-point method when the time point was different from the most recent MRI. 4224_tend_CAD_G_VC_G.pdf includes the MRI data with segmentation of case number 4224 at the most recent MRI. CAD_G and CAD_S mean that the change-of-point method predicted growth (G) or stability (S), respectively. VC_G and VC_S mean that the visual comparison detected growth (G) or stability (S), respectively. (ZIP) [file pmed.1002810.s001.zip › 7709_t0.pdf]

Case Number:7717; Time 0 (Baseline).

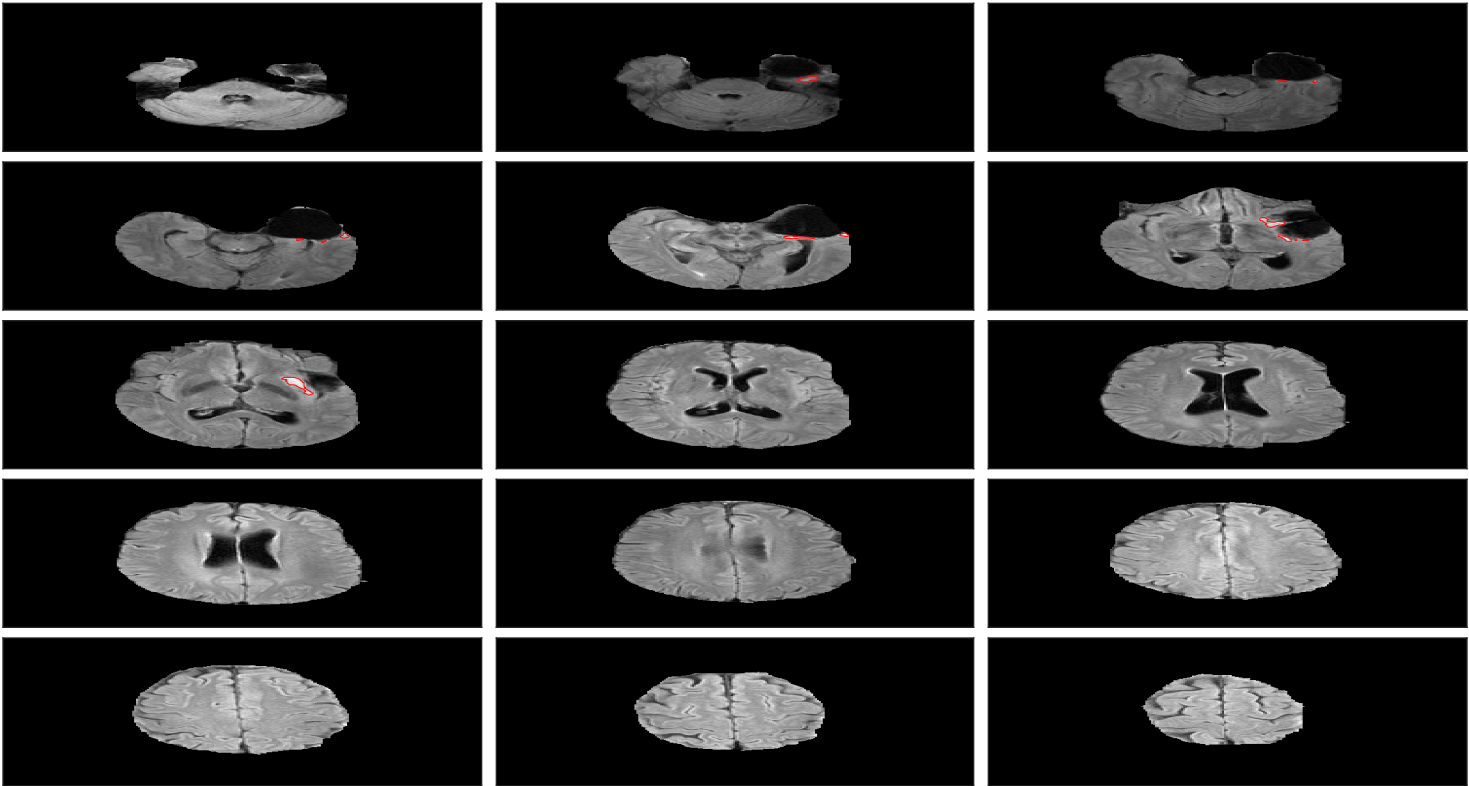

Supplement: S1 Data — The .zip file includes .pdf files labeled as follows, using case number 4224 as an example: 4224_t0.pdf includes the MRI data with segmentation of case number 4224 at baseline. 4224_t1_CAD_G_VC_S.pdf includes the MRI data with segmentation of case number 4224 at time 1, i.e., the time point of growth detection by the change-of-point method when the time point was different from the most recent MRI. 4224_tend_CAD_G_VC_G.pdf includes the MRI data with segmentation of case number 4224 at the most recent MRI. CAD_G and CAD_S mean that the change-of-point method predicted growth (G) or stability (S), respectively. VC_G and VC_S mean that the visual comparison detected growth (G) or stability (S), respectively. (ZIP) [file pmed.1002810.s001.zip › 7717_t0.pdf]

Case Number:7493; TIME 1; CAD --> GROWTH; VC --> STABLE; Interval From Baseline =10 months.

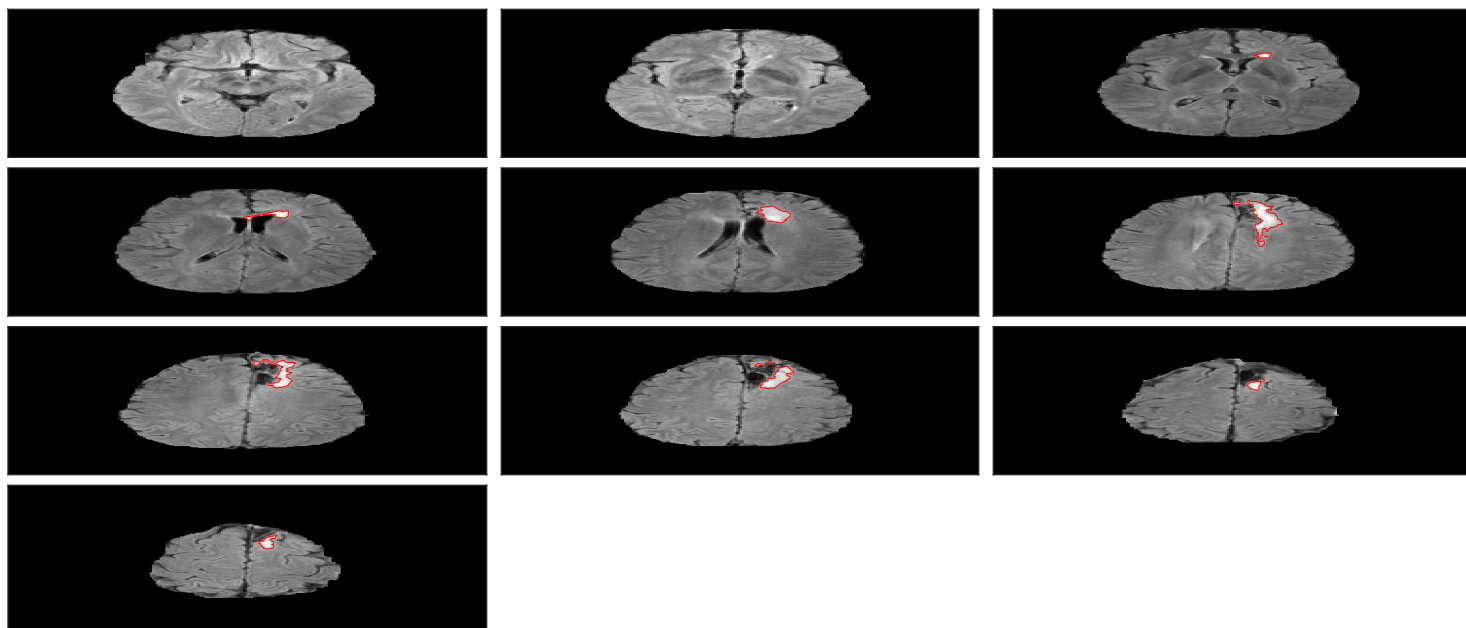

Supplement: S1 Data — The .zip file includes .pdf files labeled as follows, using case number 4224 as an example: 4224_t0.pdf includes the MRI data with segmentation of case number 4224 at baseline. 4224_t1_CAD_G_VC_S.pdf includes the MRI data with segmentation of case number 4224 at time 1, i.e., the time point of growth detection by the change-of-point method when the time point was different from the most recent MRI. 4224_tend_CAD_G_VC_G.pdf includes the MRI data with segmentation of case number 4224 at the most recent MRI. CAD_G and CAD_S mean that the change-of-point method predicted growth (G) or stability (S), respectively. VC_G and VC_S mean that the visual comparison detected growth (G) or stability (S), respectively. (ZIP) [file pmed.1002810.s001.zip › 7493_t1_CAD_G_VC_S.pdf]

Case Number:7708; TIME 1; CAD --> GROWTH; VC --> STABLE; Interval From Baseline =8 months.

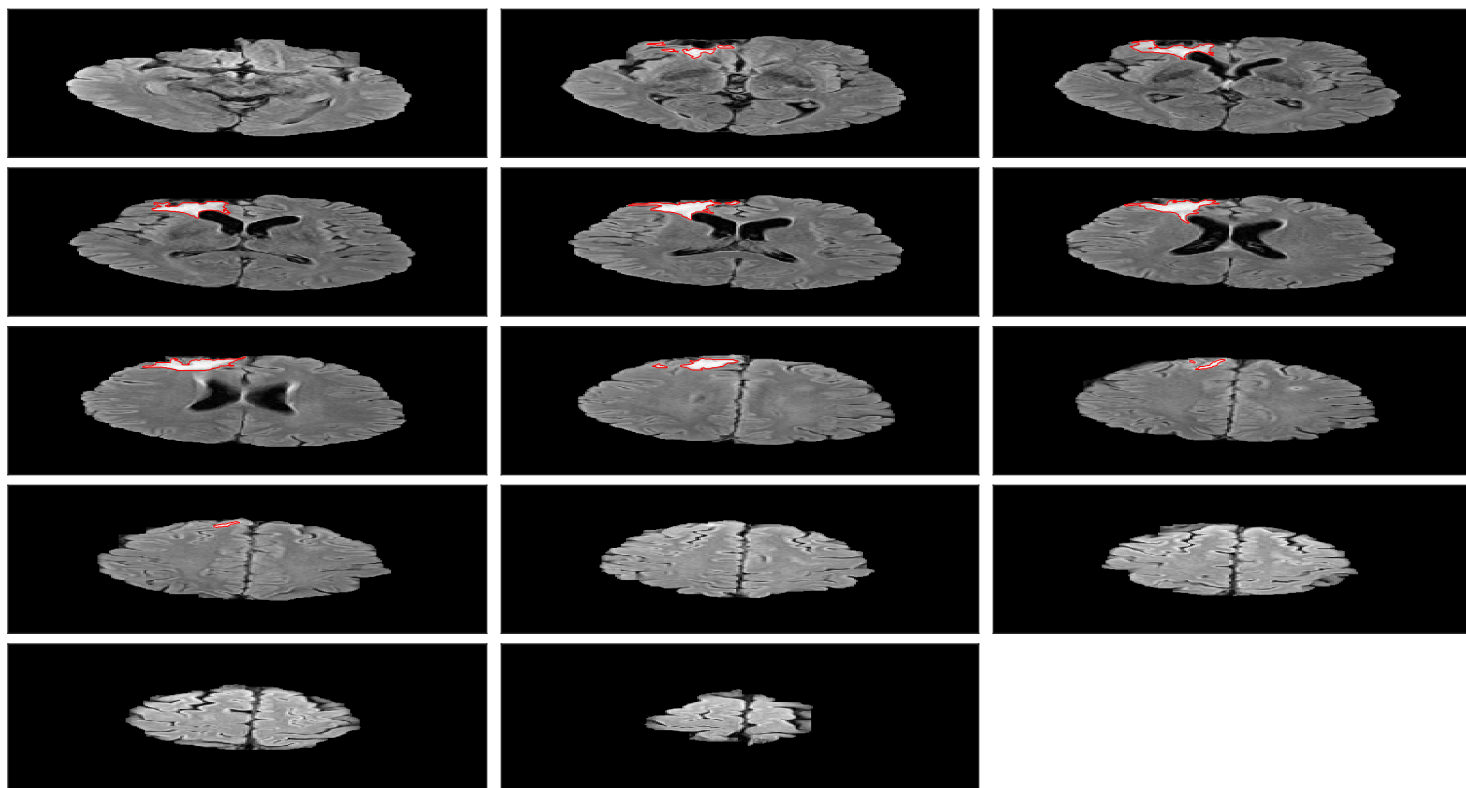

Supplement: S1 Data — The .zip file includes .pdf files labeled as follows, using case number 4224 as an example: 4224_t0.pdf includes the MRI data with segmentation of case number 4224 at baseline. 4224_t1_CAD_G_VC_S.pdf includes the MRI data with segmentation of case number 4224 at time 1, i.e., the time point of growth detection by the change-of-point method when the time point was different from the most recent MRI. 4224_tend_CAD_G_VC_G.pdf includes the MRI data with segmentation of case number 4224 at the most recent MRI. CAD_G and CAD_S mean that the change-of-point method predicted growth (G) or stability (S), respectively. VC_G and VC_S mean that the visual comparison detected growth (G) or stability (S), respectively. (ZIP) [file pmed.1002810.s001.zip › 7708_t1_CAD_G_VC_S.pdf]

Case Number:4387; Time 0 (Baseline).

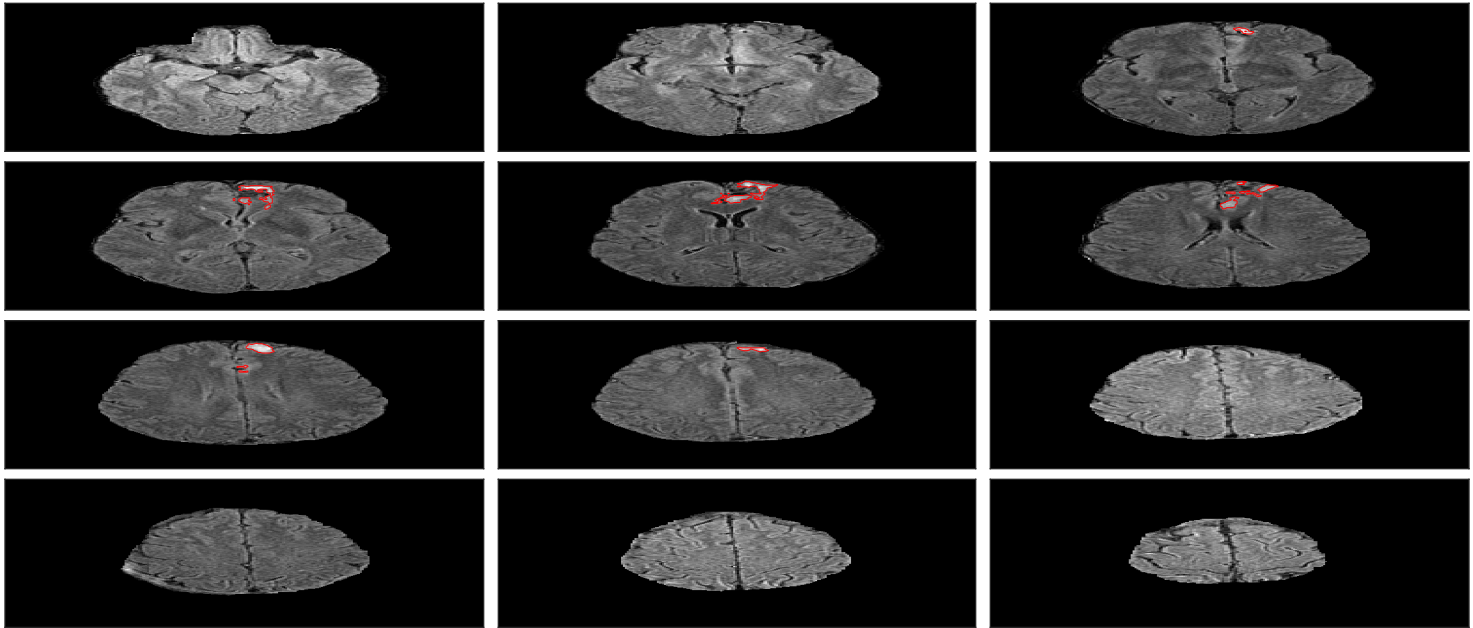

Supplement: S1 Data — The .zip file includes .pdf files labeled as follows, using case number 4224 as an example: 4224_t0.pdf includes the MRI data with segmentation of case number 4224 at baseline. 4224_t1_CAD_G_VC_S.pdf includes the MRI data with segmentation of case number 4224 at time 1, i.e., the time point of growth detection by the change-of-point method when the time point was different from the most recent MRI. 4224_tend_CAD_G_VC_G.pdf includes the MRI data with segmentation of case number 4224 at the most recent MRI. CAD_G and CAD_S mean that the change-of-point method predicted growth (G) or stability (S), respectively. VC_G and VC_S mean that the visual comparison detected growth (G) or stability (S), respectively. (ZIP) [file pmed.1002810.s001.zip › 4387_t0.pdf]

Case Number:7733; Time 0 (Baseline).

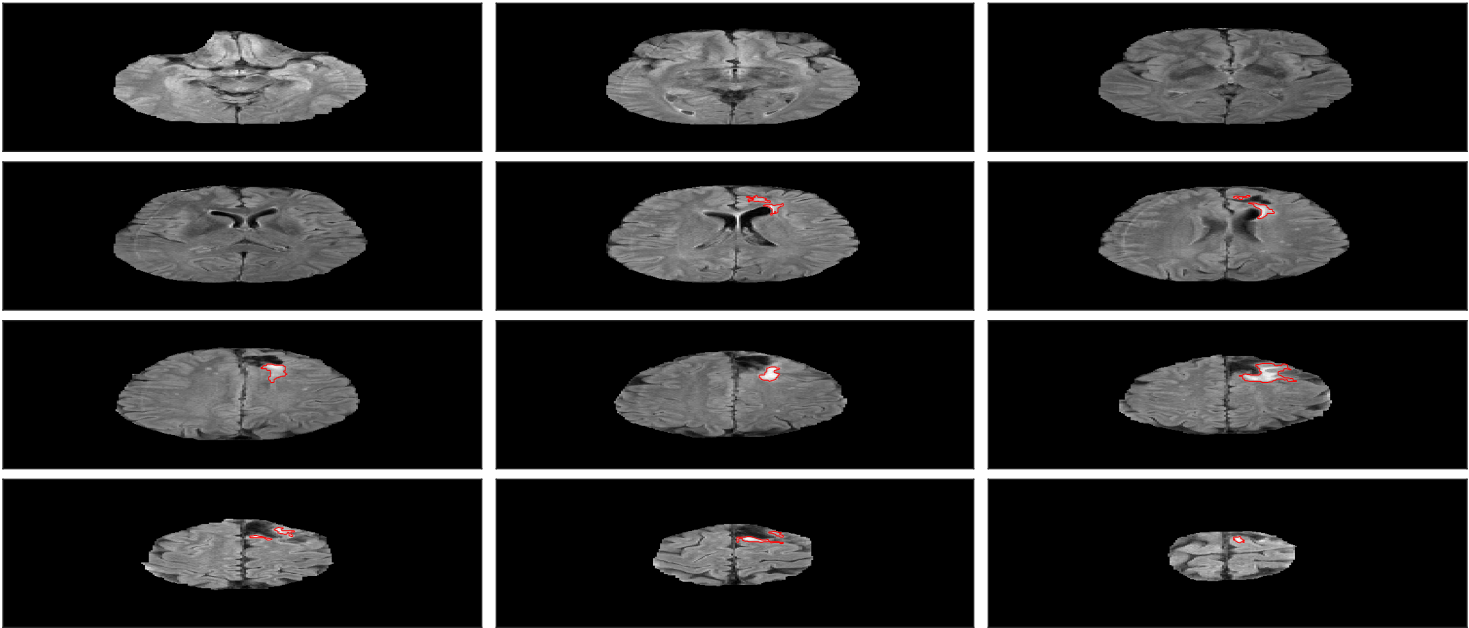

Supplement: S1 Data — The .zip file includes .pdf files labeled as follows, using case number 4224 as an example: 4224_t0.pdf includes the MRI data with segmentation of case number 4224 at baseline. 4224_t1_CAD_G_VC_S.pdf includes the MRI data with segmentation of case number 4224 at time 1, i.e., the time point of growth detection by the change-of-point method when the time point was different from the most recent MRI. 4224_tend_CAD_G_VC_G.pdf includes the MRI data with segmentation of case number 4224 at the most recent MRI. CAD_G and CAD_S mean that the change-of-point method predicted growth (G) or stability (S), respectively. VC_G and VC_S mean that the visual comparison detected growth (G) or stability (S), respectively. (ZIP) [file pmed.1002810.s001.zip › 7733_t0.pdf]

Case Number:7477; TIME 1; CAD --> GROWTH; VC --> STABLE; Interval From Baseline =7 months.

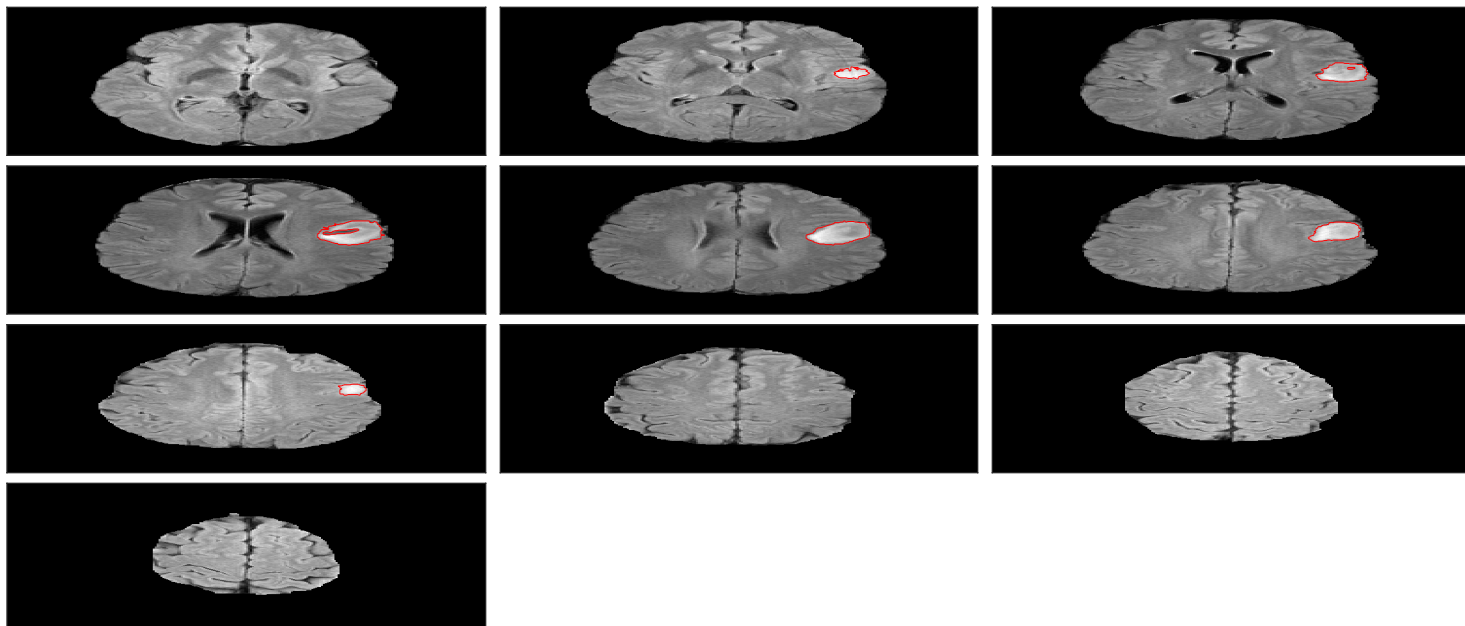

Supplement: S1 Data — The .zip file includes .pdf files labeled as follows, using case number 4224 as an example: 4224_t0.pdf includes the MRI data with segmentation of case number 4224 at baseline. 4224_t1_CAD_G_VC_S.pdf includes the MRI data with segmentation of case number 4224 at time 1, i.e., the time point of growth detection by the change-of-point method when the time point was different from the most recent MRI. 4224_tend_CAD_G_VC_G.pdf includes the MRI data with segmentation of case number 4224 at the most recent MRI. CAD_G and CAD_S mean that the change-of-point method predicted growth (G) or stability (S), respectively. VC_G and VC_S mean that the visual comparison detected growth (G) or stability (S), respectively. (ZIP) [file pmed.1002810.s001.zip › 7477_t1_CAD_G_VC_S.pdf]

Case Number:7070; Time 0 (Baseline).

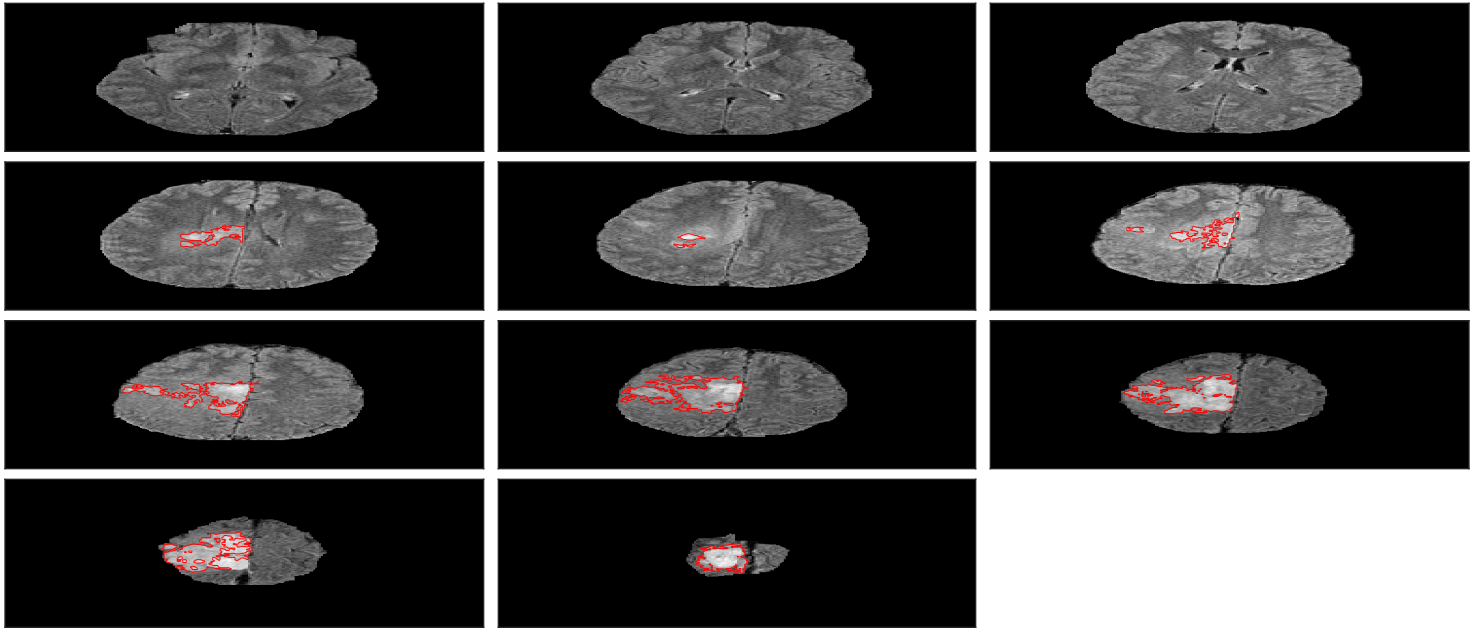

Supplement: S1 Data — The .zip file includes .pdf files labeled as follows, using case number 4224 as an example: 4224_t0.pdf includes the MRI data with segmentation of case number 4224 at baseline. 4224_t1_CAD_G_VC_S.pdf includes the MRI data with segmentation of case number 4224 at time 1, i.e., the time point of growth detection by the change-of-point method when the time point was different from the most recent MRI. 4224_tend_CAD_G_VC_G.pdf includes the MRI data with segmentation of case number 4224 at the most recent MRI. CAD_G and CAD_S mean that the change-of-point method predicted growth (G) or stability (S), respectively. VC_G and VC_S mean that the visual comparison detected growth (G) or stability (S), respectively. (ZIP) [file pmed.1002810.s001.zip › 7070_t0.pdf]

**Case Number:7070; TIME 1; CAD --> GROWTH; VC --> STABLE; Interval From Baseline =13 months.**

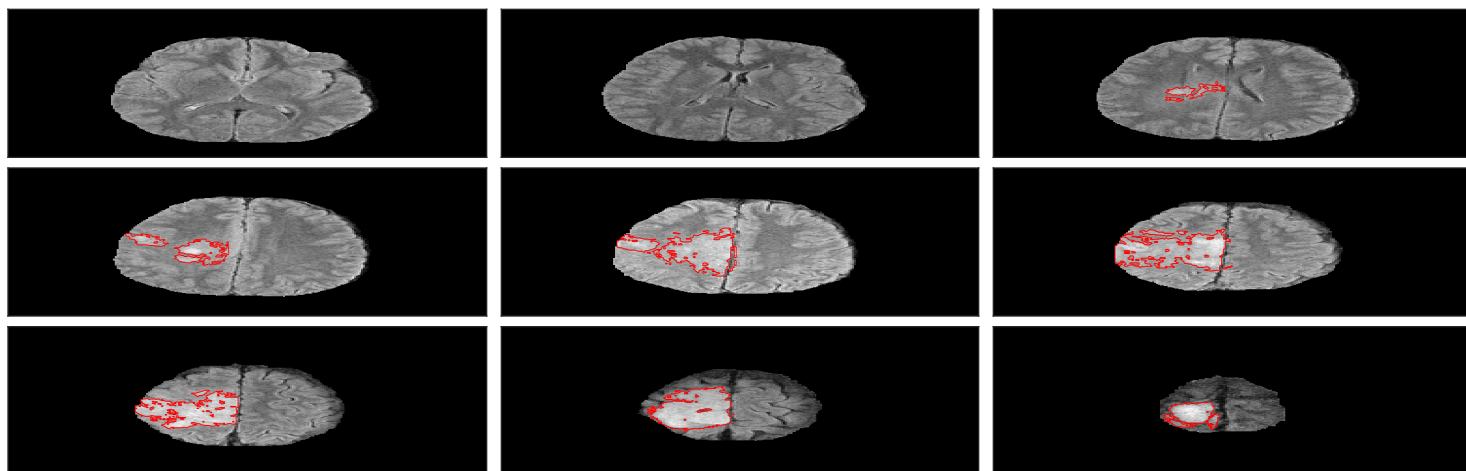

Supplement: S1 Data — The .zip file includes .pdf files labeled as follows, using case number 4224 as an example: 4224_t0.pdf includes the MRI data with segmentation of case number 4224 at baseline. 4224_t1_CAD_G_VC_S.pdf includes the MRI data with segmentation of case number 4224 at time 1, i.e., the time point of growth detection by the change-of-point method when the time point was different from the most recent MRI. 4224_tend_CAD_G_VC_G.pdf includes the MRI data with segmentation of case number 4224 at the most recent MRI. CAD_G and CAD_S mean that the change-of-point method predicted growth (G) or stability (S), respectively. VC_G and VC_S mean that the visual comparison detected growth (G) or stability (S), respectively. (ZIP) [file pmed.1002810.s001.zip › 7070_t1_CAD_G_VC_S.pdf]

Case Number:6937; LAST MRI; CAD --> GROWTH; VC --> GROWTH; Interval From Baseline =47 months.

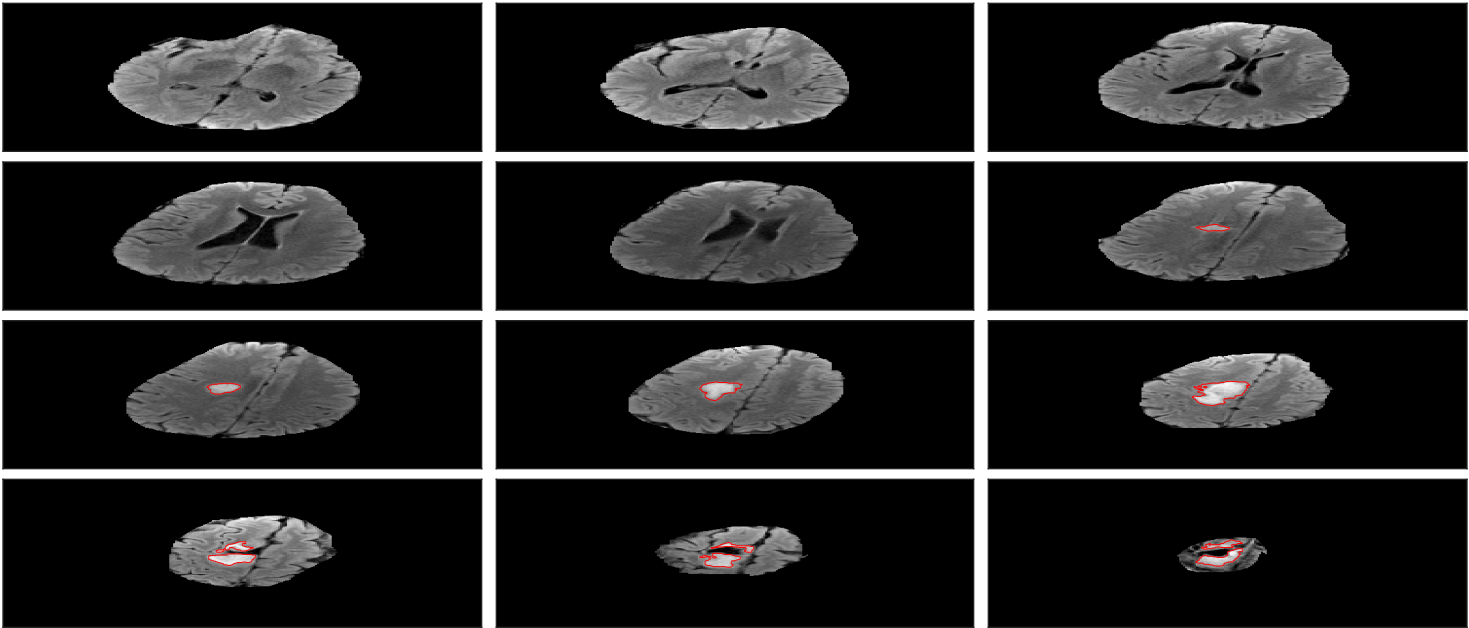

Supplement: S1 Data — The .zip file includes .pdf files labeled as follows, using case number 4224 as an example: 4224_t0.pdf includes the MRI data with segmentation of case number 4224 at baseline. 4224_t1_CAD_G_VC_S.pdf includes the MRI data with segmentation of case number 4224 at time 1, i.e., the time point of growth detection by the change-of-point method when the time point was different from the most recent MRI. 4224_tend_CAD_G_VC_G.pdf includes the MRI data with segmentation of case number 4224 at the most recent MRI. CAD_G and CAD_S mean that the change-of-point method predicted growth (G) or stability (S), respectively. VC_G and VC_S mean that the visual comparison detected growth (G) or stability (S), respectively. (ZIP) [file pmed.1002810.s001.zip › 6937_tend_CAD_G_VC_G.pdf]

Case Number:4384; TIME 1; CAD --> GROWTH; VC --> STABLE; Interval From Baseline =20 months.

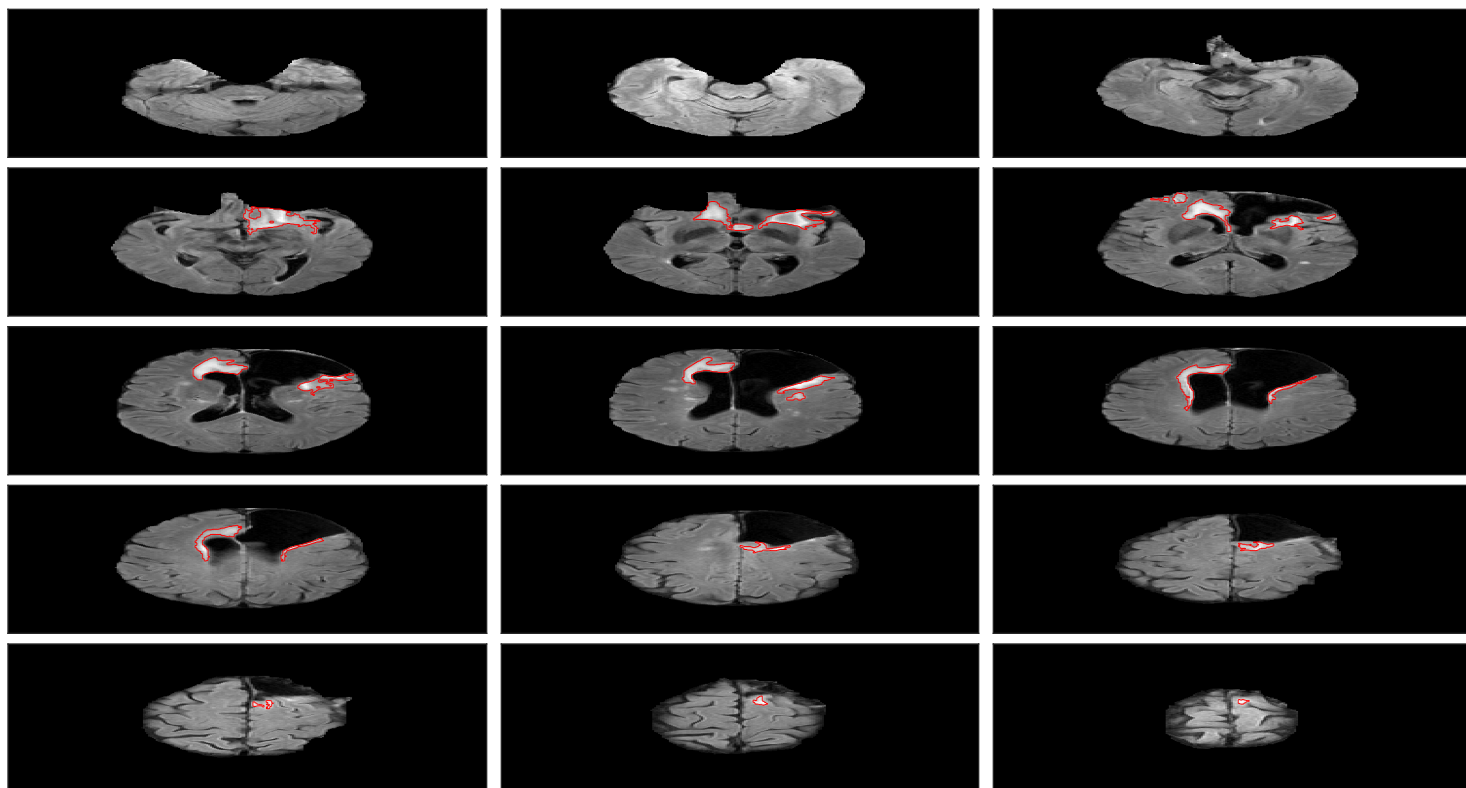

Supplement: S1 Data — The .zip file includes .pdf files labeled as follows, using case number 4224 as an example: 4224_t0.pdf includes the MRI data with segmentation of case number 4224 at baseline. 4224_t1_CAD_G_VC_S.pdf includes the MRI data with segmentation of case number 4224 at time 1, i.e., the time point of growth detection by the change-of-point method when the time point was different from the most recent MRI. 4224_tend_CAD_G_VC_G.pdf includes the MRI data with segmentation of case number 4224 at the most recent MRI. CAD_G and CAD_S mean that the change-of-point method predicted growth (G) or stability (S), respectively. VC_G and VC_S mean that the visual comparison detected growth (G) or stability (S), respectively. (ZIP) [file pmed.1002810.s001.zip › 4384_t1_CAD_G_VC_S.pdf]

Case Number:7489; TIME 1; CAD --> GROWTH; VC --> STABLE; Interval From Baseline =11 months.

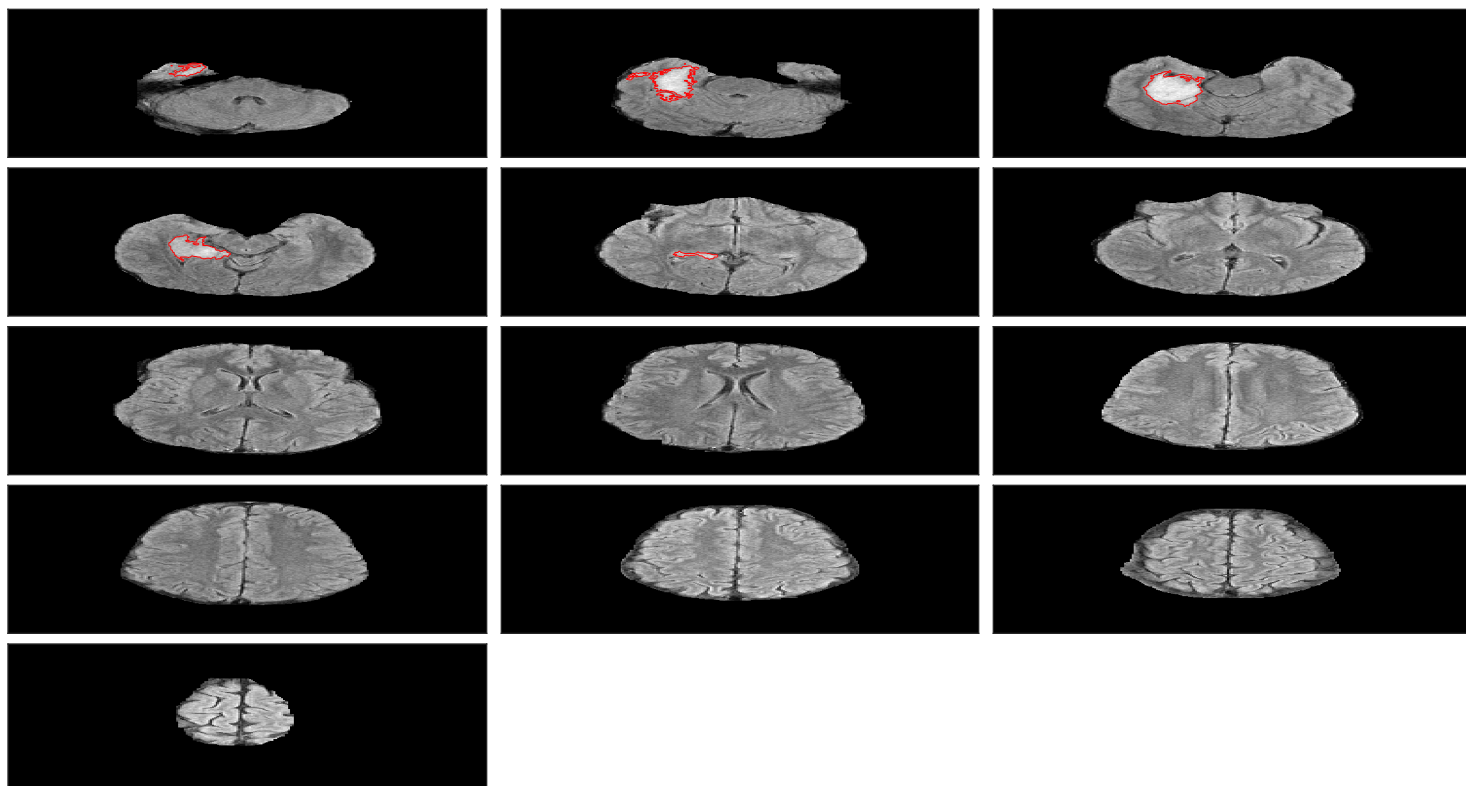

Supplement: S1 Data — The .zip file includes .pdf files labeled as follows, using case number 4224 as an example: 4224_t0.pdf includes the MRI data with segmentation of case number 4224 at baseline. 4224_t1_CAD_G_VC_S.pdf includes the MRI data with segmentation of case number 4224 at time 1, i.e., the time point of growth detection by the change-of-point method when the time point was different from the most recent MRI. 4224_tend_CAD_G_VC_G.pdf includes the MRI data with segmentation of case number 4224 at the most recent MRI. CAD_G and CAD_S mean that the change-of-point method predicted growth (G) or stability (S), respectively. VC_G and VC_S mean that the visual comparison detected growth (G) or stability (S), respectively. (ZIP) [file pmed.1002810.s001.zip › 7489_t1_CAD_G_VC_S.pdf]

Case Number:7094; TIME 1; CAD --> GROWTH; VC --> STABLE; Interval From Baseline =11 months.

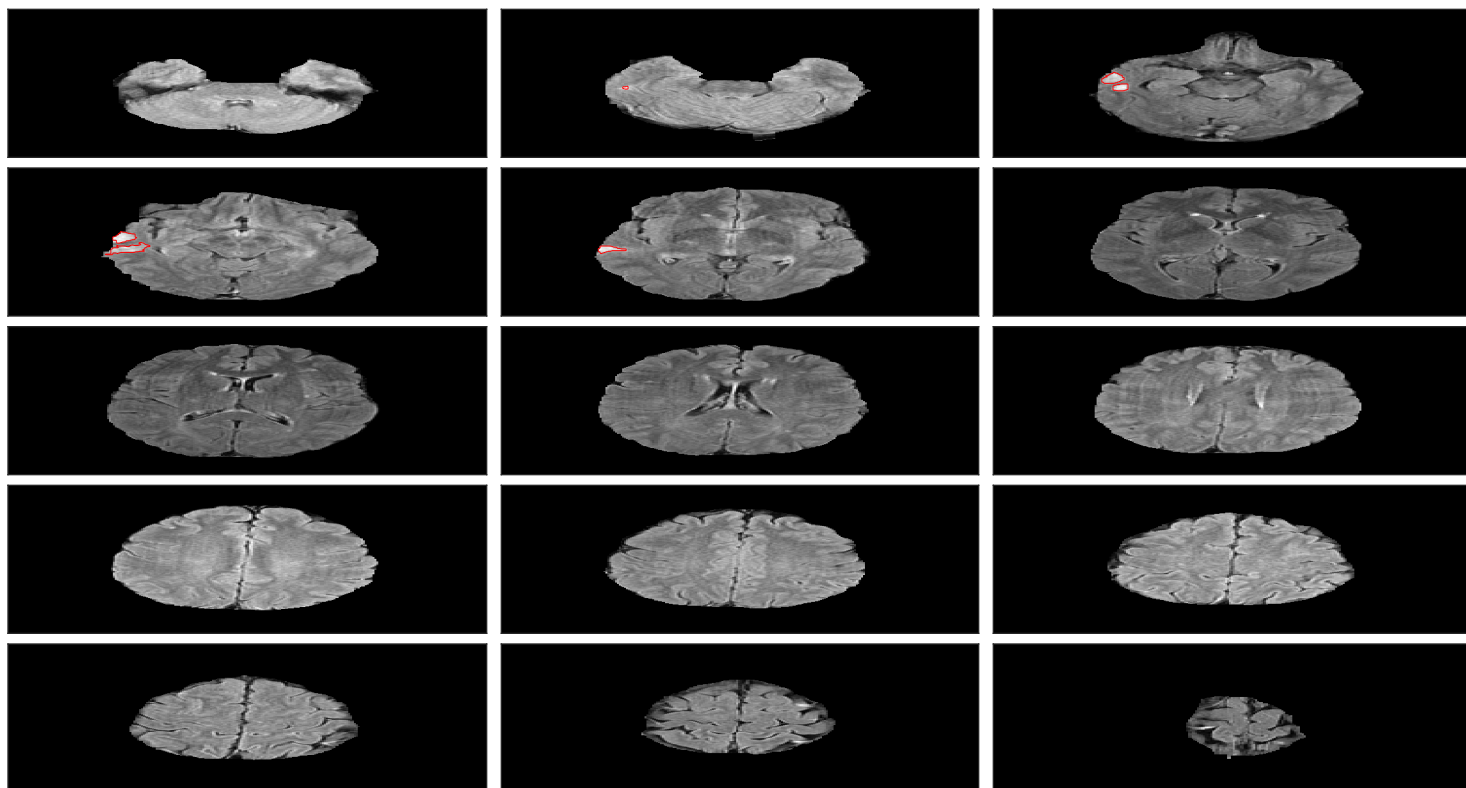

Supplement: S1 Data — The .zip file includes .pdf files labeled as follows, using case number 4224 as an example: 4224_t0.pdf includes the MRI data with segmentation of case number 4224 at baseline. 4224_t1_CAD_G_VC_S.pdf includes the MRI data with segmentation of case number 4224 at time 1, i.e., the time point of growth detection by the change-of-point method when the time point was different from the most recent MRI. 4224_tend_CAD_G_VC_G.pdf includes the MRI data with segmentation of case number 4224 at the most recent MRI. CAD_G and CAD_S mean that the change-of-point method predicted growth (G) or stability (S), respectively. VC_G and VC_S mean that the visual comparison detected growth (G) or stability (S), respectively. (ZIP) [file pmed.1002810.s001.zip › 7094_t1_CAD_G_VC_S.pdf]

Case Number:4225; Time 0 (Baseline).

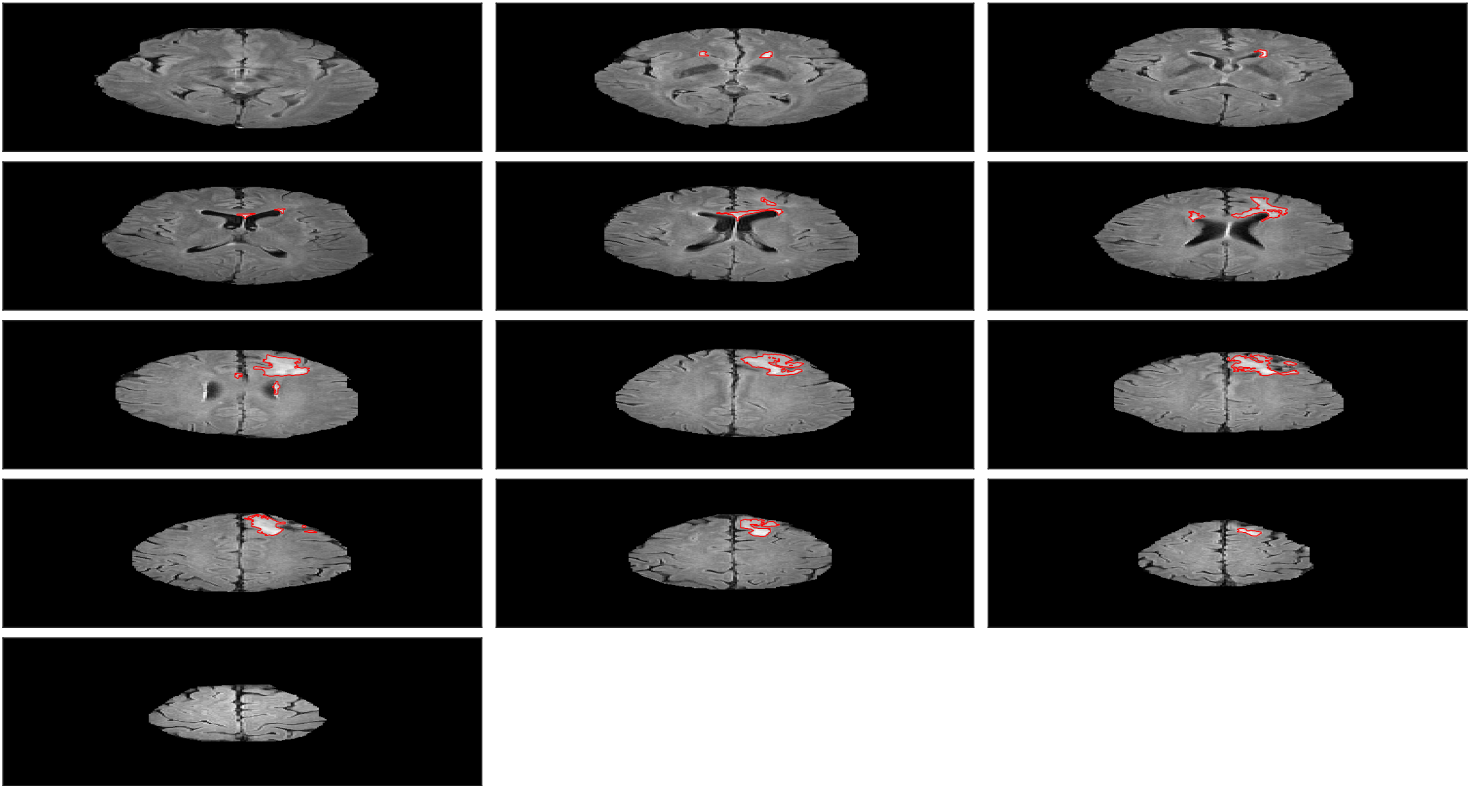

Supplement: S1 Data — The .zip file includes .pdf files labeled as follows, using case number 4224 as an example: 4224_t0.pdf includes the MRI data with segmentation of case number 4224 at baseline. 4224_t1_CAD_G_VC_S.pdf includes the MRI data with segmentation of case number 4224 at time 1, i.e., the time point of growth detection by the change-of-point method when the time point was different from the most recent MRI. 4224_tend_CAD_G_VC_G.pdf includes the MRI data with segmentation of case number 4224 at the most recent MRI. CAD_G and CAD_S mean that the change-of-point method predicted growth (G) or stability (S), respectively. VC_G and VC_S mean that the visual comparison detected growth (G) or stability (S), respectively. (ZIP) [file pmed.1002810.s001.zip › 4225_t0.pdf]

Case Number:7736; LAST MRI; CAD --> GROWTH; VC --> STABLE; Interval From Baseline =88 months.

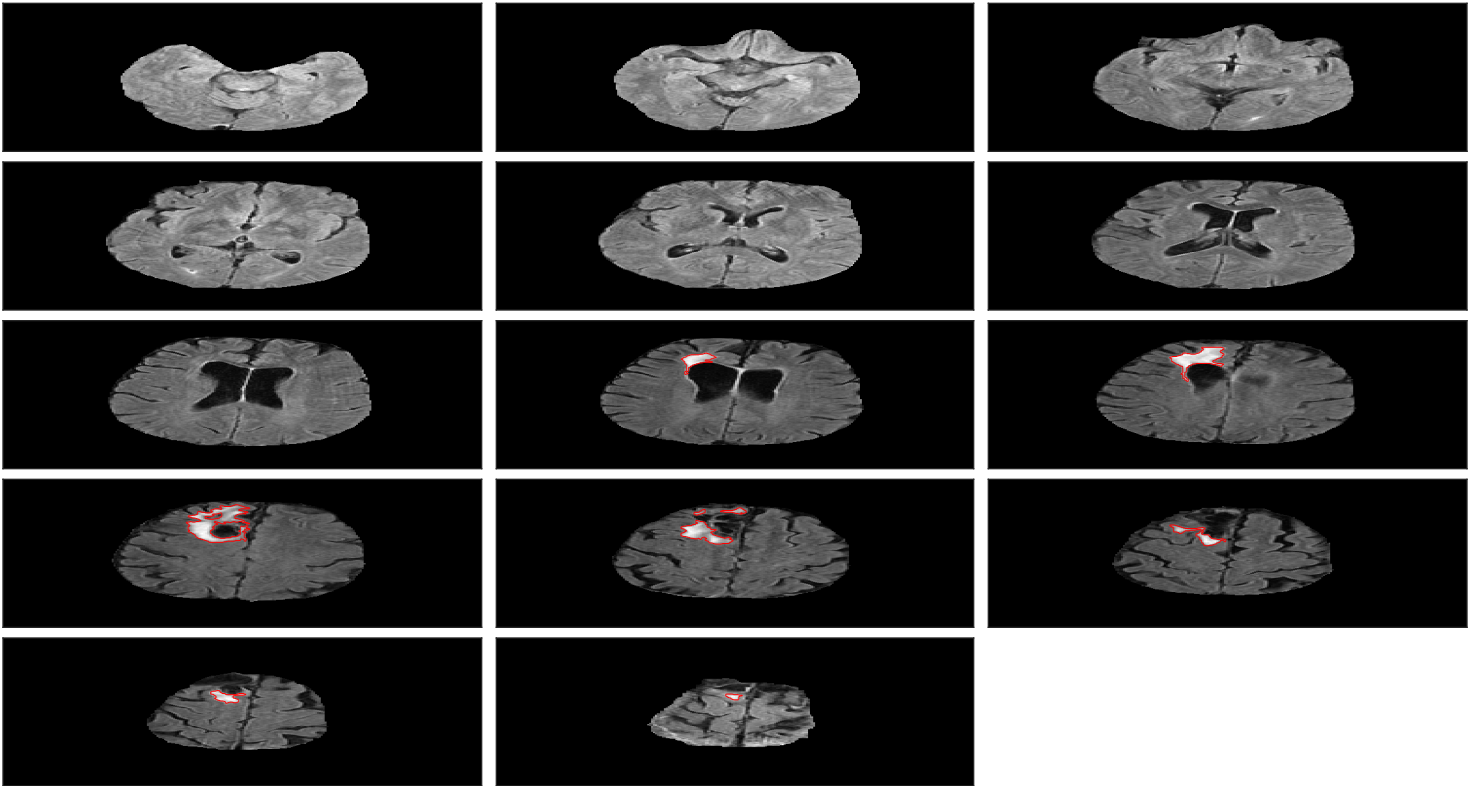

Supplement: S1 Data — The .zip file includes .pdf files labeled as follows, using case number 4224 as an example: 4224_t0.pdf includes the MRI data with segmentation of case number 4224 at baseline. 4224_t1_CAD_G_VC_S.pdf includes the MRI data with segmentation of case number 4224 at time 1, i.e., the time point of growth detection by the change-of-point method when the time point was different from the most recent MRI. 4224_tend_CAD_G_VC_G.pdf includes the MRI data with segmentation of case number 4224 at the most recent MRI. CAD_G and CAD_S mean that the change-of-point method predicted growth (G) or stability (S), respectively. VC_G and VC_S mean that the visual comparison detected growth (G) or stability (S), respectively. (ZIP) [file pmed.1002810.s001.zip › 7736_tend_CAD_G_VC_S.pdf]

Case Number:6936; LAST MRI; CAD --> GROWTH; VC --> GROWTH; Interval From Baseline =13 months.

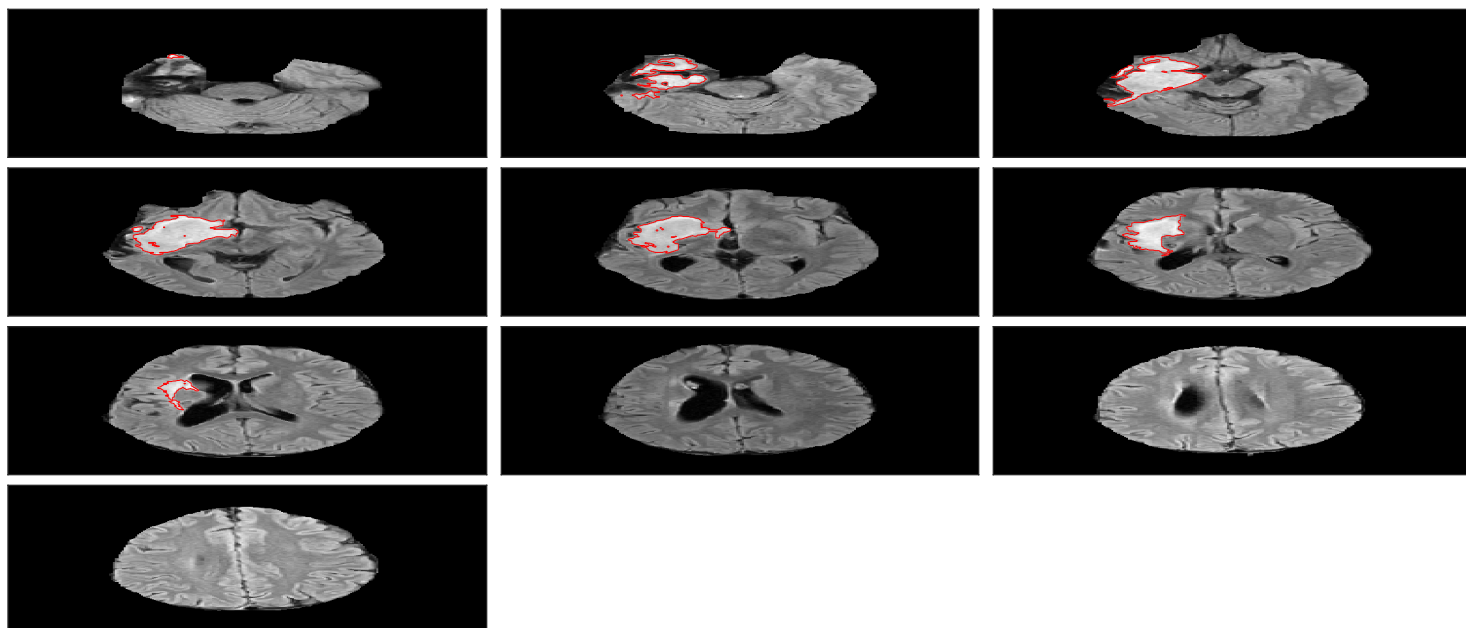

Supplement: S1 Data — The .zip file includes .pdf files labeled as follows, using case number 4224 as an example: 4224_t0.pdf includes the MRI data with segmentation of case number 4224 at baseline. 4224_t1_CAD_G_VC_S.pdf includes the MRI data with segmentation of case number 4224 at time 1, i.e., the time point of growth detection by the change-of-point method when the time point was different from the most recent MRI. 4224_tend_CAD_G_VC_G.pdf includes the MRI data with segmentation of case number 4224 at the most recent MRI. CAD_G and CAD_S mean that the change-of-point method predicted growth (G) or stability (S), respectively. VC_G and VC_S mean that the visual comparison detected growth (G) or stability (S), respectively. (ZIP) [file pmed.1002810.s001.zip › 6936_tend_CAD_G_VC_G.pdf]

Case Number:6936; Time 0 (Baseline).

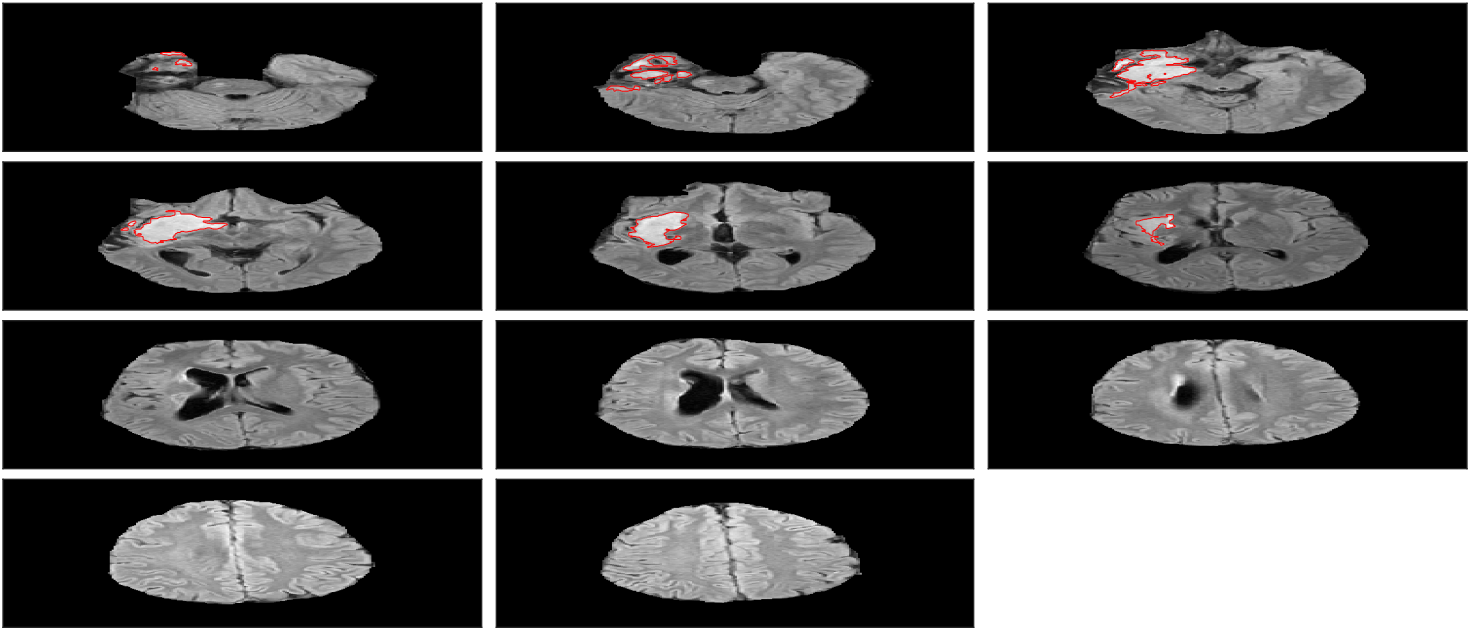

Supplement: S1 Data — The .zip file includes .pdf files labeled as follows, using case number 4224 as an example: 4224_t0.pdf includes the MRI data with segmentation of case number 4224 at baseline. 4224_t1_CAD_G_VC_S.pdf includes the MRI data with segmentation of case number 4224 at time 1, i.e., the time point of growth detection by the change-of-point method when the time point was different from the most recent MRI. 4224_tend_CAD_G_VC_G.pdf includes the MRI data with segmentation of case number 4224 at the most recent MRI. CAD_G and CAD_S mean that the change-of-point method predicted growth (G) or stability (S), respectively. VC_G and VC_S mean that the visual comparison detected growth (G) or stability (S), respectively. (ZIP) [file pmed.1002810.s001.zip › 6936_t0.pdf]

Case Number:7471; TIME 1; CAD --> GROWTH; VC --> STABLE; Interval From Baseline =9 months.

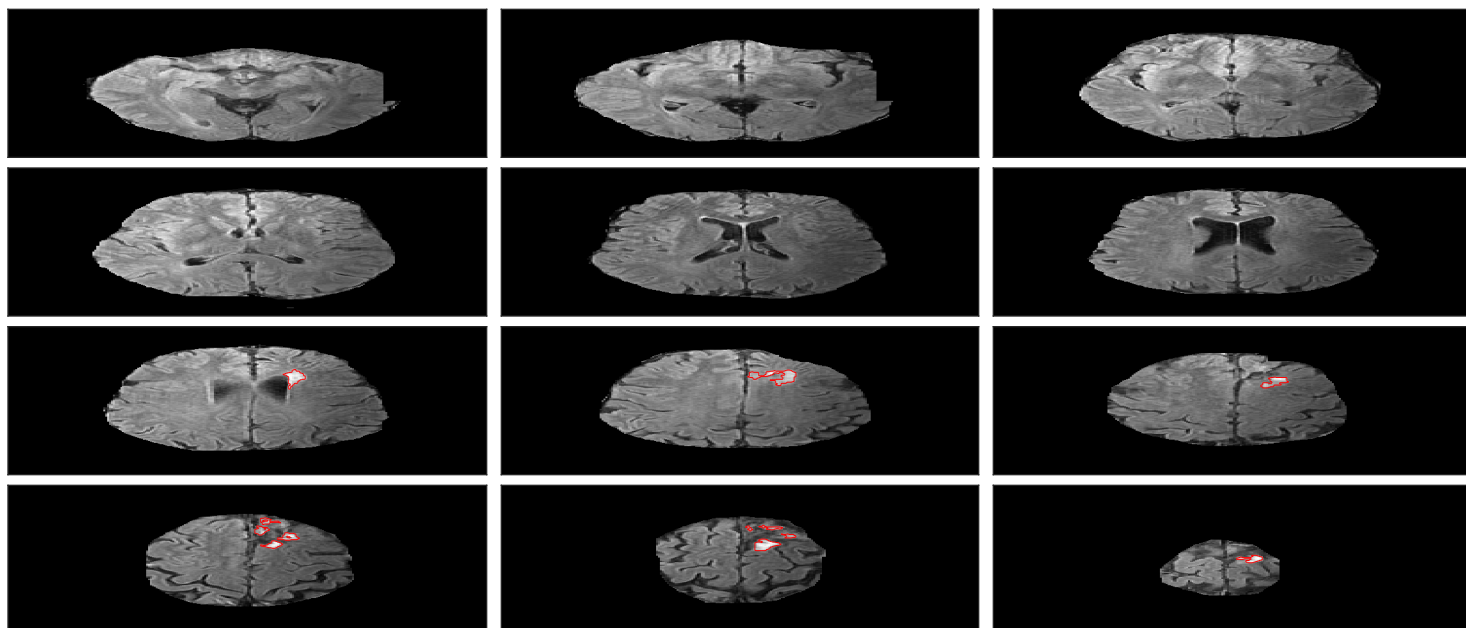

Supplement: S1 Data — The .zip file includes .pdf files labeled as follows, using case number 4224 as an example: 4224_t0.pdf includes the MRI data with segmentation of case number 4224 at baseline. 4224_t1_CAD_G_VC_S.pdf includes the MRI data with segmentation of case number 4224 at time 1, i.e., the time point of growth detection by the change-of-point method when the time point was different from the most recent MRI. 4224_tend_CAD_G_VC_G.pdf includes the MRI data with segmentation of case number 4224 at the most recent MRI. CAD_G and CAD_S mean that the change-of-point method predicted growth (G) or stability (S), respectively. VC_G and VC_S mean that the visual comparison detected growth (G) or stability (S), respectively. (ZIP) [file pmed.1002810.s001.zip › 7471_t1_CAD_G_VC_S.pdf]

Case Number:7732; LAST MRI; CAD --> STABLE; VC --> STABLE; Interval From Baseline =29 months.

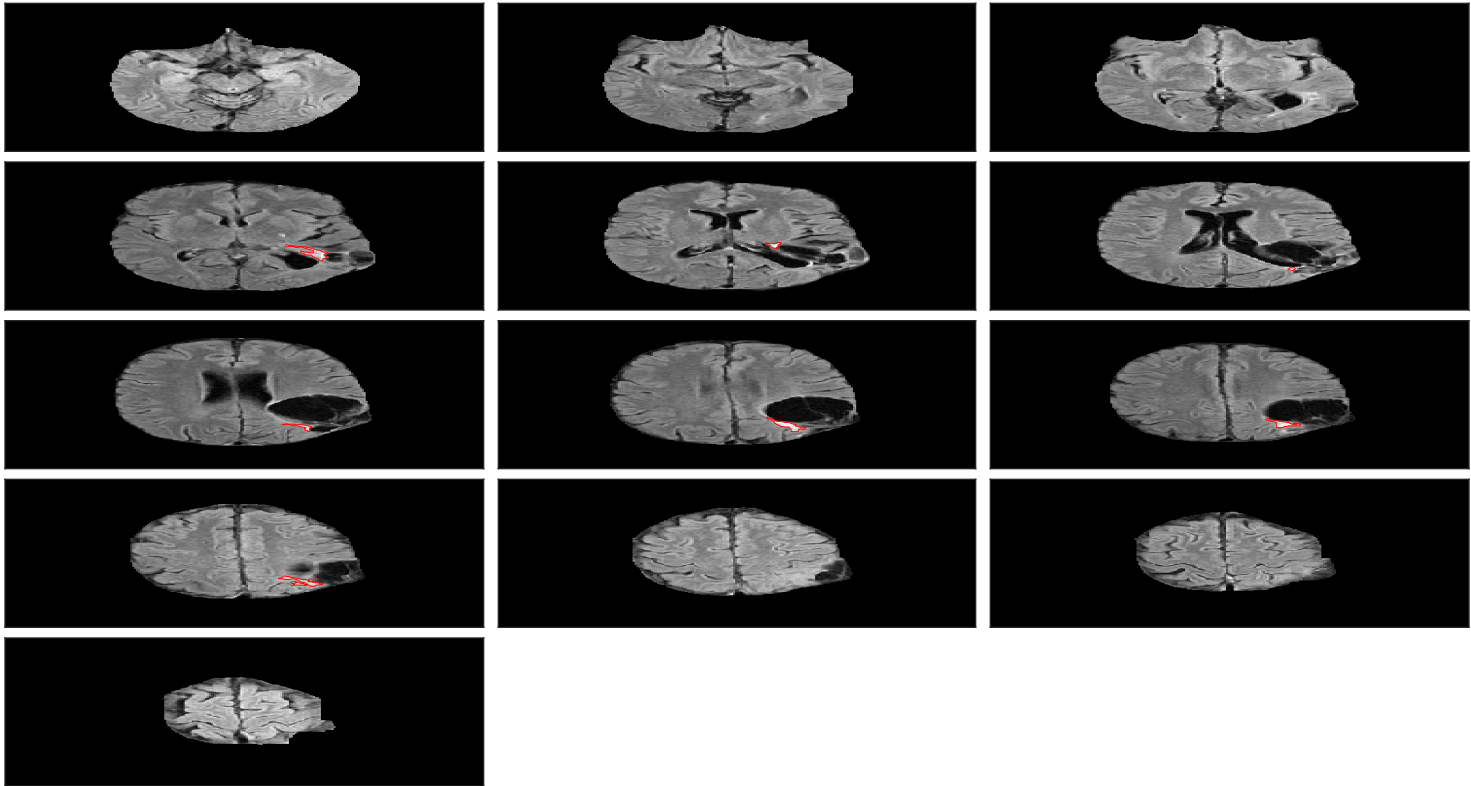

Supplement: S1 Data — The .zip file includes .pdf files labeled as follows, using case number 4224 as an example: 4224_t0.pdf includes the MRI data with segmentation of case number 4224 at baseline. 4224_t1_CAD_G_VC_S.pdf includes the MRI data with segmentation of case number 4224 at time 1, i.e., the time point of growth detection by the change-of-point method when the time point was different from the most recent MRI. 4224_tend_CAD_G_VC_G.pdf includes the MRI data with segmentation of case number 4224 at the most recent MRI. CAD_G and CAD_S mean that the change-of-point method predicted growth (G) or stability (S), respectively. VC_G and VC_S mean that the visual comparison detected growth (G) or stability (S), respectively. (ZIP) [file pmed.1002810.s001.zip › 7732_tend_CAD_S_VC_S.pdf]

Case Number:6935; LAST MRI; CAD --> GROWTH; VC --> GROWTH; Interval From Baseline =112 months.

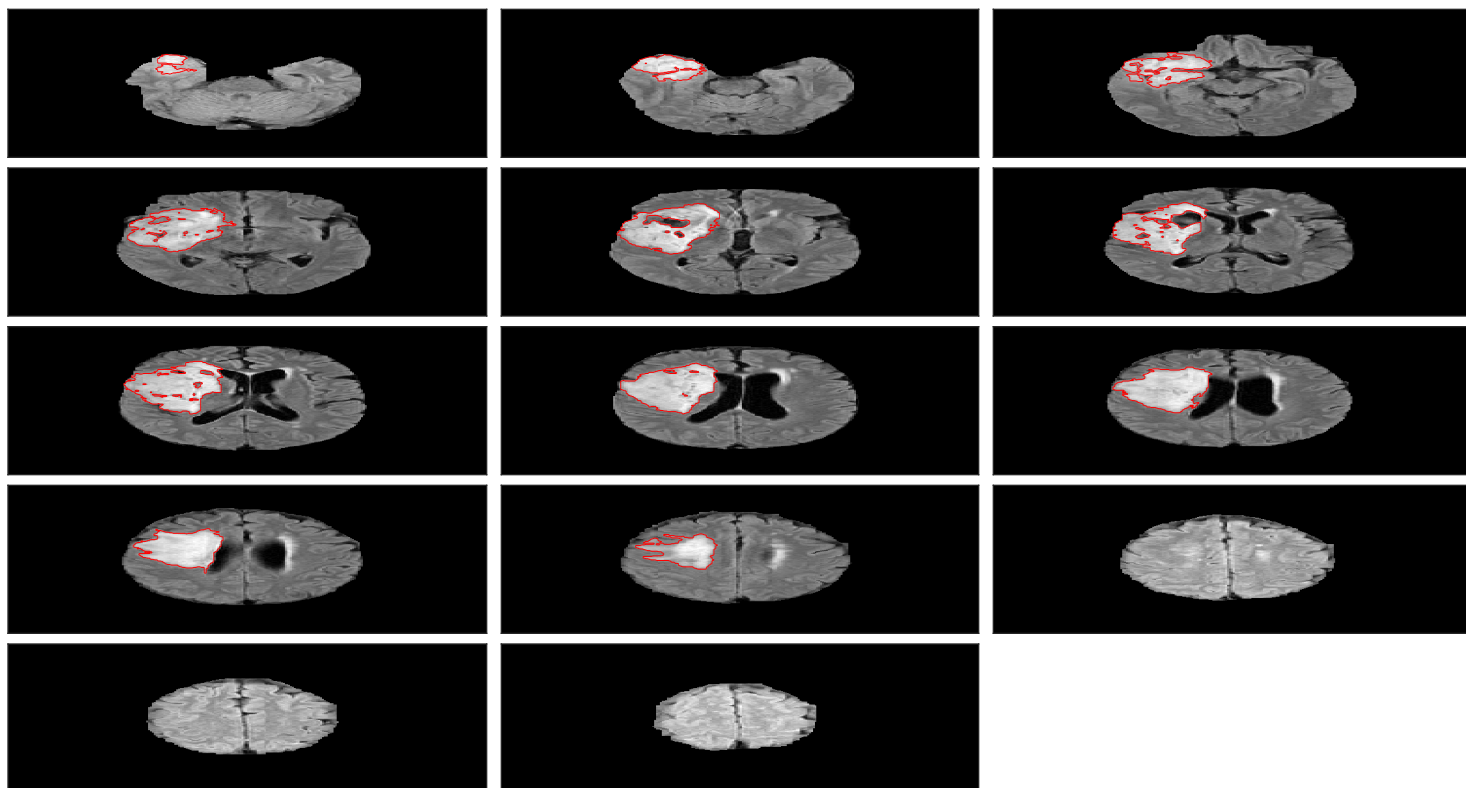

Supplement: S1 Data — The .zip file includes .pdf files labeled as follows, using case number 4224 as an example: 4224_t0.pdf includes the MRI data with segmentation of case number 4224 at baseline. 4224_t1_CAD_G_VC_S.pdf includes the MRI data with segmentation of case number 4224 at time 1, i.e., the time point of growth detection by the change-of-point method when the time point was different from the most recent MRI. 4224_tend_CAD_G_VC_G.pdf includes the MRI data with segmentation of case number 4224 at the most recent MRI. CAD_G and CAD_S mean that the change-of-point method predicted growth (G) or stability (S), respectively. VC_G and VC_S mean that the visual comparison detected growth (G) or stability (S), respectively. (ZIP) [file pmed.1002810.s001.zip › 6935_tend_CAD_G_VC_G.pdf]

Case Number:7492; Time 0 (Baseline).

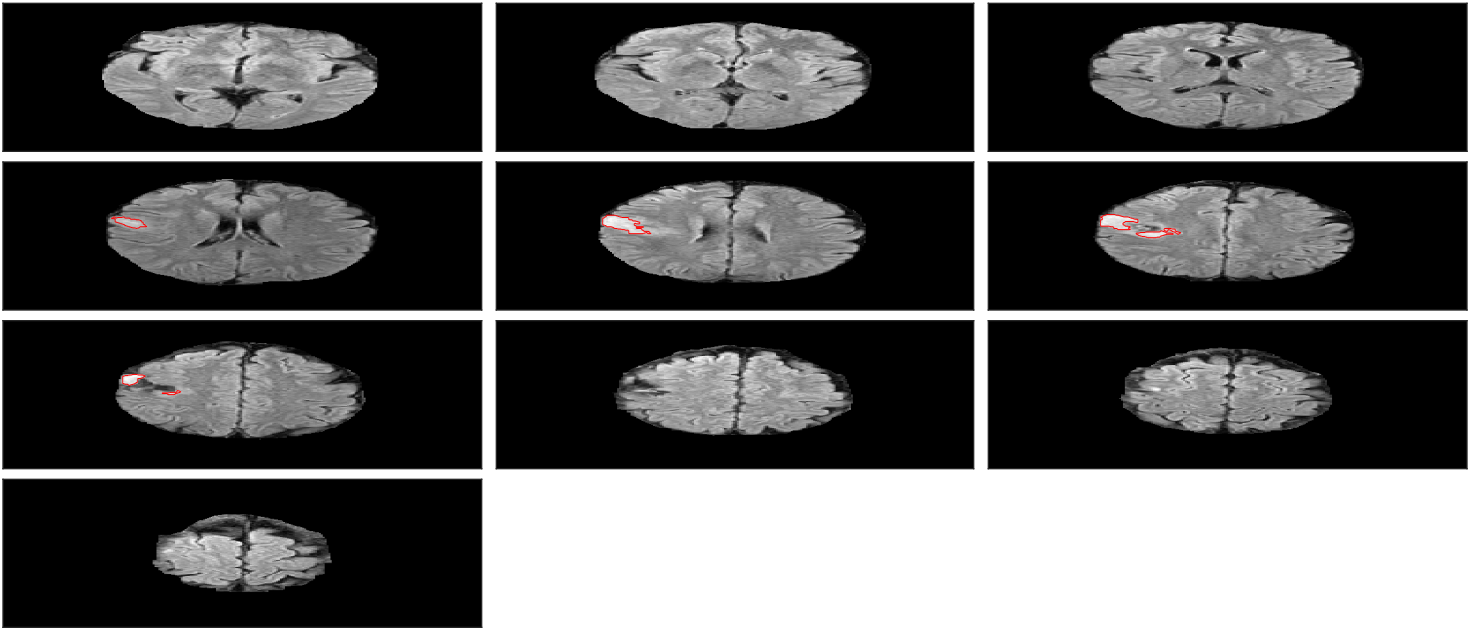

Supplement: S1 Data — The .zip file includes .pdf files labeled as follows, using case number 4224 as an example: 4224_t0.pdf includes the MRI data with segmentation of case number 4224 at baseline. 4224_t1_CAD_G_VC_S.pdf includes the MRI data with segmentation of case number 4224 at time 1, i.e., the time point of growth detection by the change-of-point method when the time point was different from the most recent MRI. 4224_tend_CAD_G_VC_G.pdf includes the MRI data with segmentation of case number 4224 at the most recent MRI. CAD_G and CAD_S mean that the change-of-point method predicted growth (G) or stability (S), respectively. VC_G and VC_S mean that the visual comparison detected growth (G) or stability (S), respectively. (ZIP) [file pmed.1002810.s001.zip › 7492_t0.pdf]

Case Number:7729; Time 0 (Baseline).

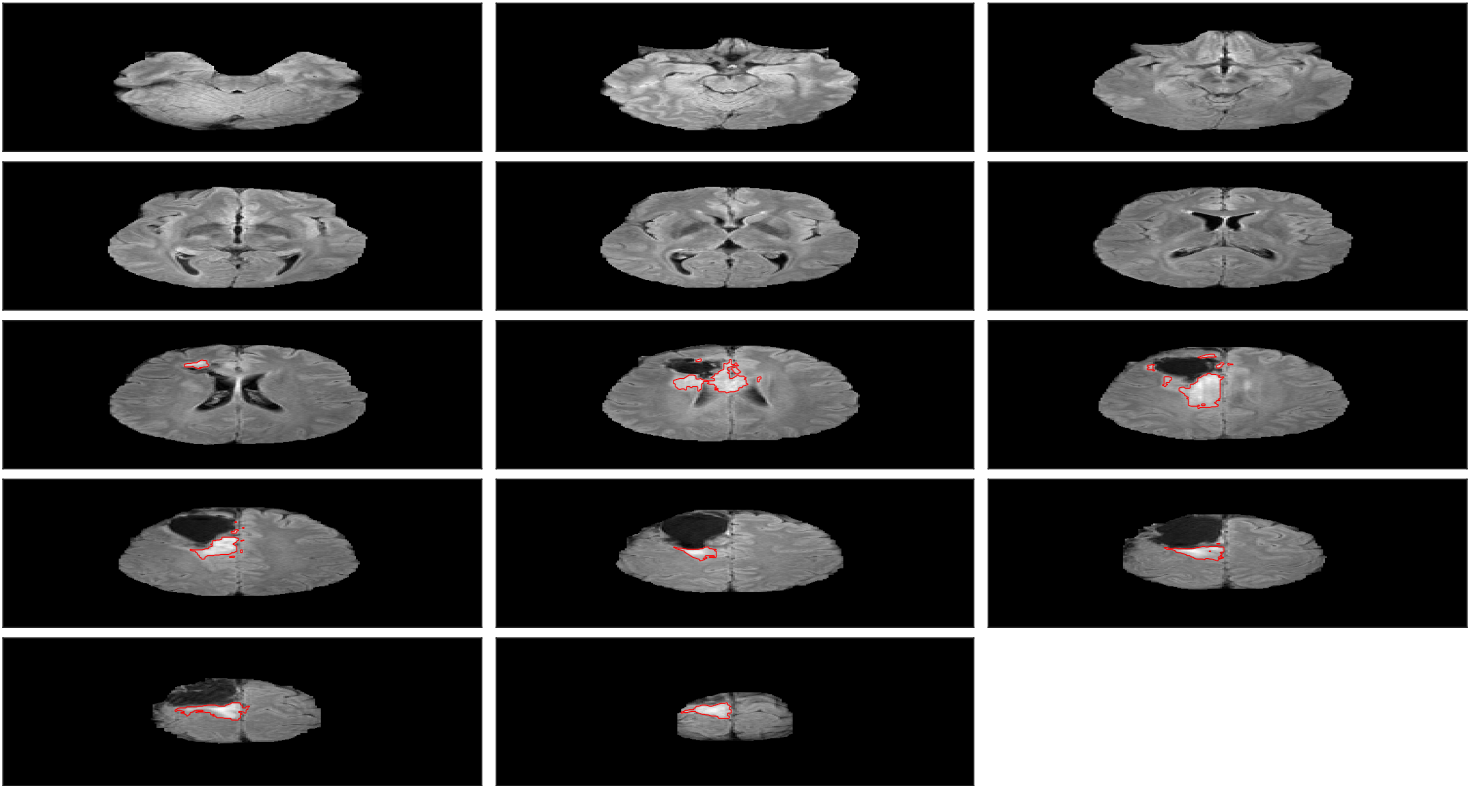

Supplement: S1 Data — The .zip file includes .pdf files labeled as follows, using case number 4224 as an example: 4224_t0.pdf includes the MRI data with segmentation of case number 4224 at baseline. 4224_t1_CAD_G_VC_S.pdf includes the MRI data with segmentation of case number 4224 at time 1, i.e., the time point of growth detection by the change-of-point method when the time point was different from the most recent MRI. 4224_tend_CAD_G_VC_G.pdf includes the MRI data with segmentation of case number 4224 at the most recent MRI. CAD_G and CAD_S mean that the change-of-point method predicted growth (G) or stability (S), respectively. VC_G and VC_S mean that the visual comparison detected growth (G) or stability (S), respectively. (ZIP) [file pmed.1002810.s001.zip › 7729_t0.pdf]

Case Number:7490; TIME 1; CAD --> GROWTH; VC --> STABLE; Interval From Baseline =23 months.

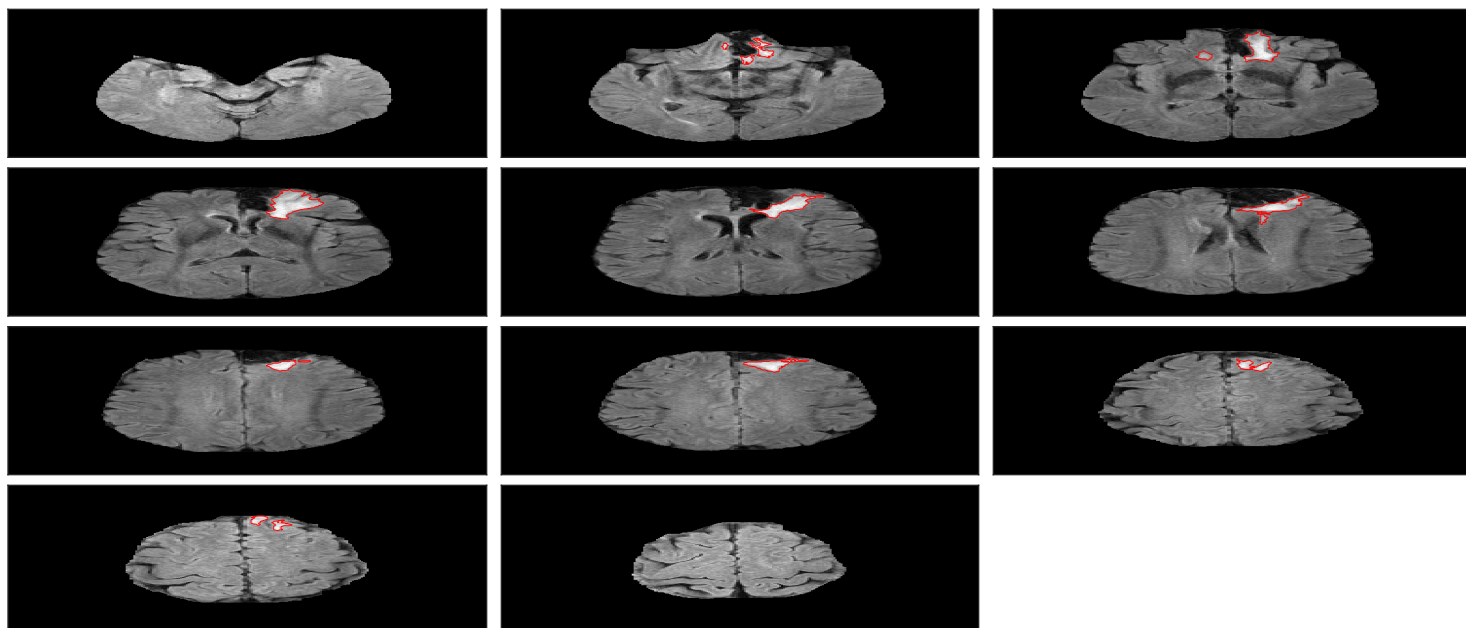

Supplement: S1 Data — The .zip file includes .pdf files labeled as follows, using case number 4224 as an example: 4224_t0.pdf includes the MRI data with segmentation of case number 4224 at baseline. 4224_t1_CAD_G_VC_S.pdf includes the MRI data with segmentation of case number 4224 at time 1, i.e., the time point of growth detection by the change-of-point method when the time point was different from the most recent MRI. 4224_tend_CAD_G_VC_G.pdf includes the MRI data with segmentation of case number 4224 at the most recent MRI. CAD_G and CAD_S mean that the change-of-point method predicted growth (G) or stability (S), respectively. VC_G and VC_S mean that the visual comparison detected growth (G) or stability (S), respectively. (ZIP) [file pmed.1002810.s001.zip › 7490_t1_CAD_G_VC_S.pdf]

Case Number:7754; Time 0 (Baseline).

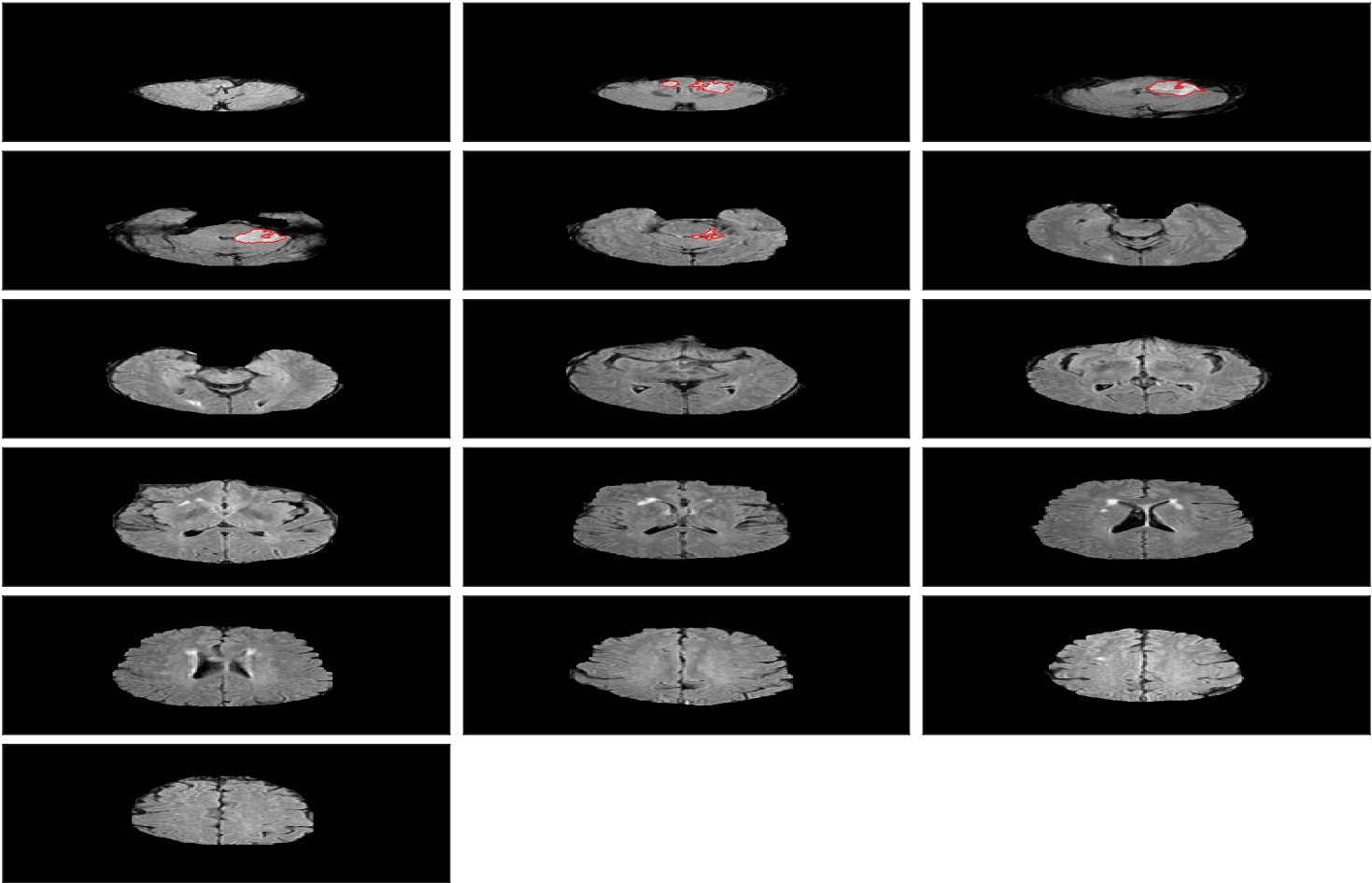

Supplement: S1 Data — The .zip file includes .pdf files labeled as follows, using case number 4224 as an example: 4224_t0.pdf includes the MRI data with segmentation of case number 4224 at baseline. 4224_t1_CAD_G_VC_S.pdf includes the MRI data with segmentation of case number 4224 at time 1, i.e., the time point of growth detection by the change-of-point method when the time point was different from the most recent MRI. 4224_tend_CAD_G_VC_G.pdf includes the MRI data with segmentation of case number 4224 at the most recent MRI. CAD_G and CAD_S mean that the change-of-point method predicted growth (G) or stability (S), respectively. VC_G and VC_S mean that the visual comparison detected growth (G) or stability (S), respectively. (ZIP) [file pmed.1002810.s001.zip › 7754_t0.pdf]

Case Number:7717; TIME 1; CAD --> GROWTH; VC --> STABLE; Interval From Baseline =9 months.

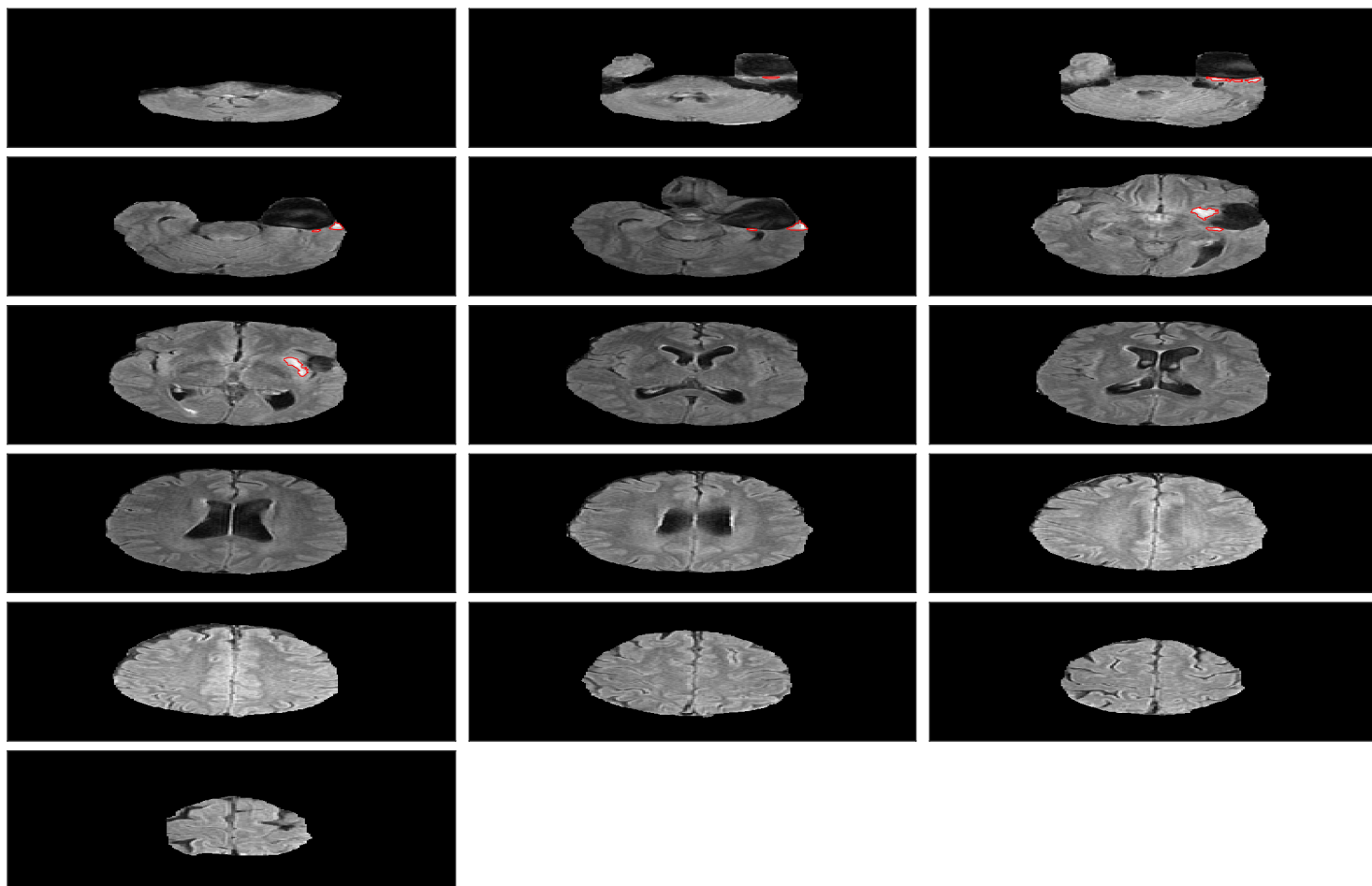

Supplement: S1 Data — The .zip file includes .pdf files labeled as follows, using case number 4224 as an example: 4224_t0.pdf includes the MRI data with segmentation of case number 4224 at baseline. 4224_t1_CAD_G_VC_S.pdf includes the MRI data with segmentation of case number 4224 at time 1, i.e., the time point of growth detection by the change-of-point method when the time point was different from the most recent MRI. 4224_tend_CAD_G_VC_G.pdf includes the MRI data with segmentation of case number 4224 at the most recent MRI. CAD_G and CAD_S mean that the change-of-point method predicted growth (G) or stability (S), respectively. VC_G and VC_S mean that the visual comparison detected growth (G) or stability (S), respectively. (ZIP) [file pmed.1002810.s001.zip › 7717_t1_CAD_G_VC_S.pdf]

Case Number:7730; LAST MRI; CAD --> STABLE; VC --> STABLE; Interval From Baseline =47 months.

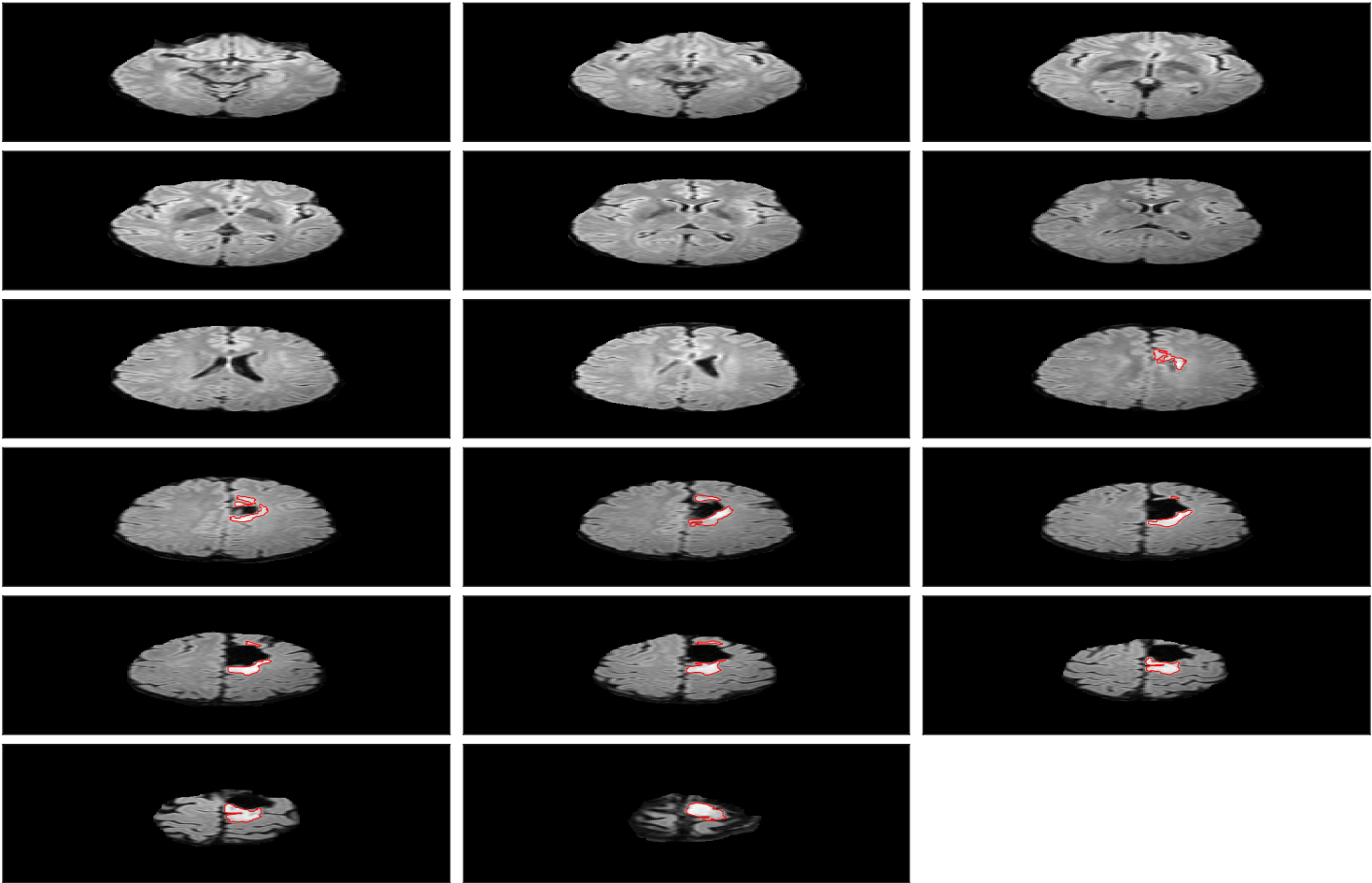

Supplement: S1 Data — The .zip file includes .pdf files labeled as follows, using case number 4224 as an example: 4224_t0.pdf includes the MRI data with segmentation of case number 4224 at baseline. 4224_t1_CAD_G_VC_S.pdf includes the MRI data with segmentation of case number 4224 at time 1, i.e., the time point of growth detection by the change-of-point method when the time point was different from the most recent MRI. 4224_tend_CAD_G_VC_G.pdf includes the MRI data with segmentation of case number 4224 at the most recent MRI. CAD_G and CAD_S mean that the change-of-point method predicted growth (G) or stability (S), respectively. VC_G and VC_S mean that the visual comparison detected growth (G) or stability (S), respectively. (ZIP) [file pmed.1002810.s001.zip › 7730_tend_CAD_S_VC_S.pdf]

Case Number:6934; LAST MRI; CAD --> GROWTH; VC --> GROWTH; Interval From Baseline =79 months.

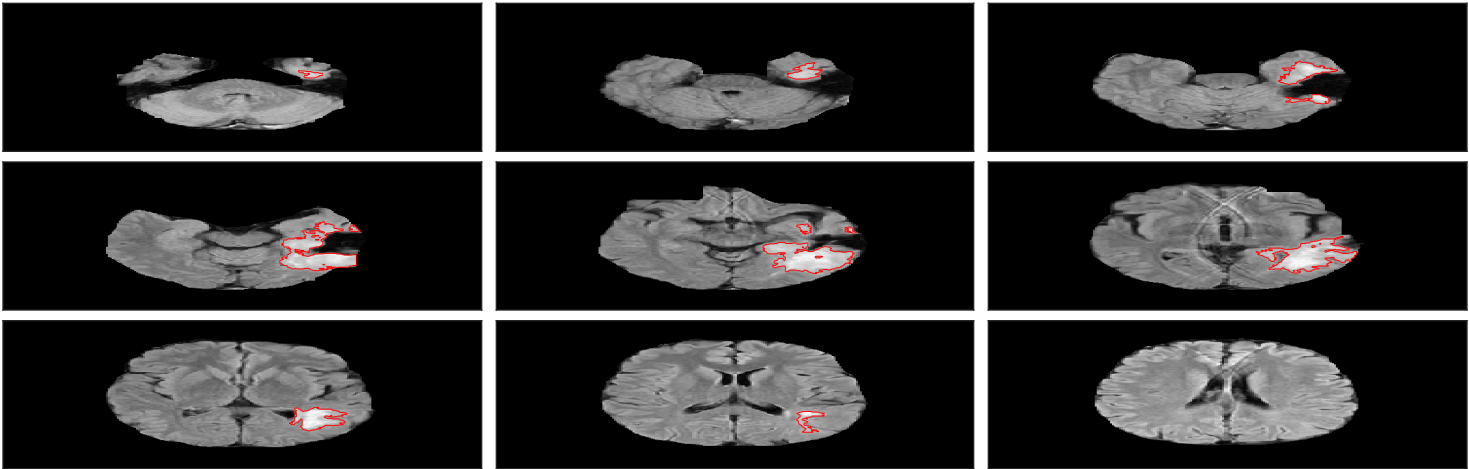

Supplement: S1 Data — The .zip file includes .pdf files labeled as follows, using case number 4224 as an example: 4224_t0.pdf includes the MRI data with segmentation of case number 4224 at baseline. 4224_t1_CAD_G_VC_S.pdf includes the MRI data with segmentation of case number 4224 at time 1, i.e., the time point of growth detection by the change-of-point method when the time point was different from the most recent MRI. 4224_tend_CAD_G_VC_G.pdf includes the MRI data with segmentation of case number 4224 at the most recent MRI. CAD_G and CAD_S mean that the change-of-point method predicted growth (G) or stability (S), respectively. VC_G and VC_S mean that the visual comparison detected growth (G) or stability (S), respectively. (ZIP) [file pmed.1002810.s001.zip › 6934_tend_CAD_G_VC_G.pdf]

Case Number:7713; Time 0 (Baseline).

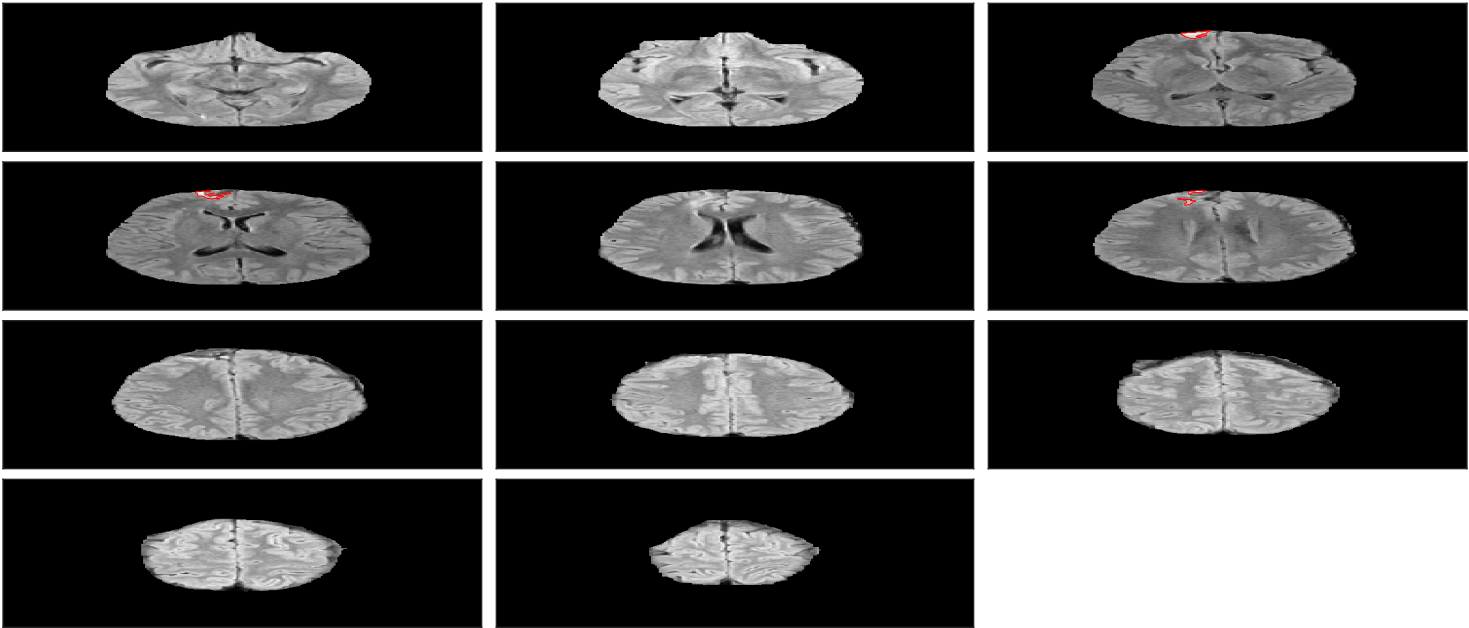

Supplement: S1 Data — The .zip file includes .pdf files labeled as follows, using case number 4224 as an example: 4224_t0.pdf includes the MRI data with segmentation of case number 4224 at baseline. 4224_t1_CAD_G_VC_S.pdf includes the MRI data with segmentation of case number 4224 at time 1, i.e., the time point of growth detection by the change-of-point method when the time point was different from the most recent MRI. 4224_tend_CAD_G_VC_G.pdf includes the MRI data with segmentation of case number 4224 at the most recent MRI. CAD_G and CAD_S mean that the change-of-point method predicted growth (G) or stability (S), respectively. VC_G and VC_S mean that the visual comparison detected growth (G) or stability (S), respectively. (ZIP) [file pmed.1002810.s001.zip › 7713_t0.pdf]

Case Number:7729; LAST MRI; CAD --> STABLE; VC --> STABLE; Interval From Baseline =8 months.

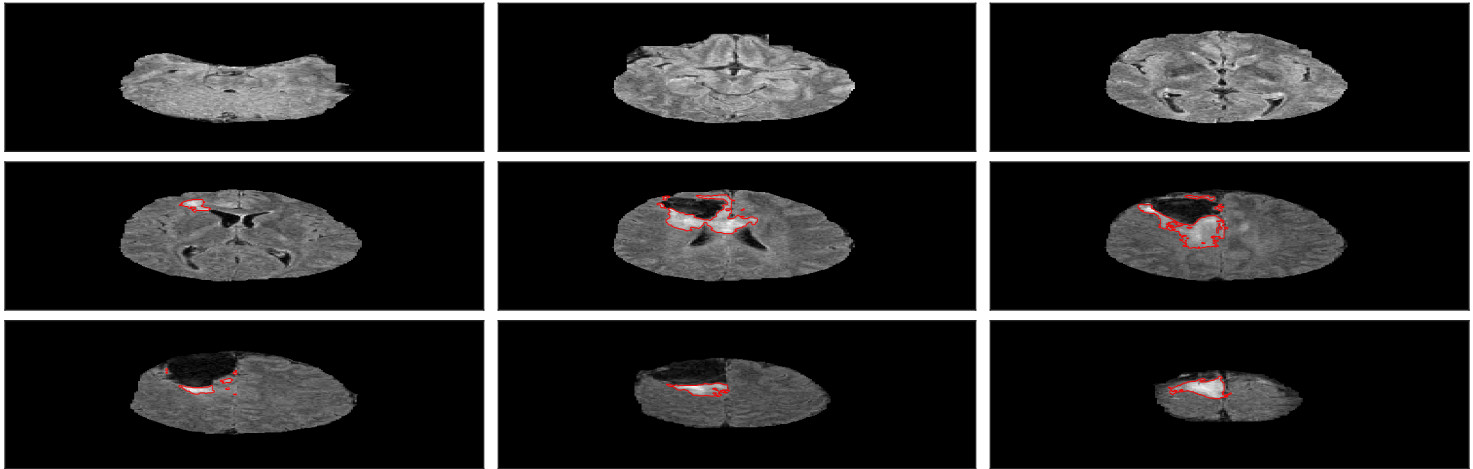

Supplement: S1 Data — The .zip file includes .pdf files labeled as follows, using case number 4224 as an example: 4224_t0.pdf includes the MRI data with segmentation of case number 4224 at baseline. 4224_t1_CAD_G_VC_S.pdf includes the MRI data with segmentation of case number 4224 at time 1, i.e., the time point of growth detection by the change-of-point method when the time point was different from the most recent MRI. 4224_tend_CAD_G_VC_G.pdf includes the MRI data with segmentation of case number 4224 at the most recent MRI. CAD_G and CAD_S mean that the change-of-point method predicted growth (G) or stability (S), respectively. VC_G and VC_S mean that the visual comparison detected growth (G) or stability (S), respectively. (ZIP) [file pmed.1002810.s001.zip › 7729_tend_CAD_S_VC_S.pdf]

Case Number:7474; TIME 1; CAD --> GROWTH; VC --> STABLE; Interval From Baseline =9 months.

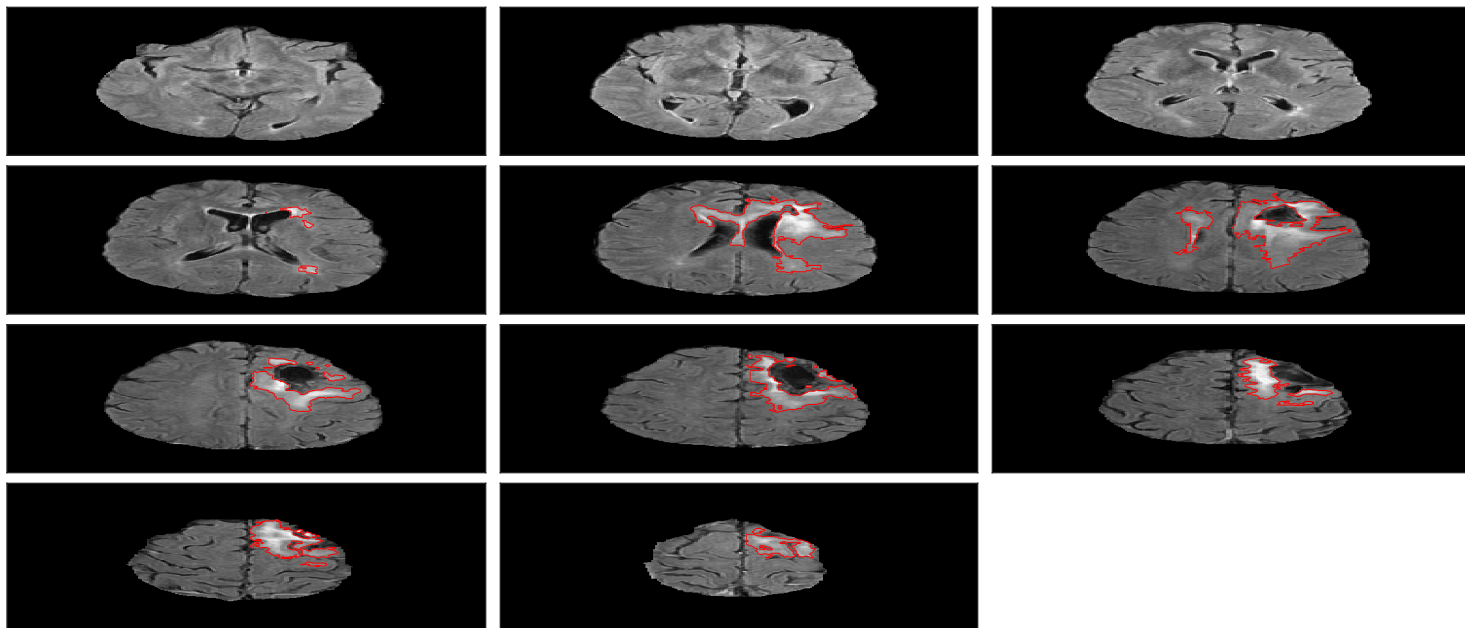

Supplement: S1 Data — The .zip file includes .pdf files labeled as follows, using case number 4224 as an example: 4224_t0.pdf includes the MRI data with segmentation of case number 4224 at baseline. 4224_t1_CAD_G_VC_S.pdf includes the MRI data with segmentation of case number 4224 at time 1, i.e., the time point of growth detection by the change-of-point method when the time point was different from the most recent MRI. 4224_tend_CAD_G_VC_G.pdf includes the MRI data with segmentation of case number 4224 at the most recent MRI. CAD_G and CAD_S mean that the change-of-point method predicted growth (G) or stability (S), respectively. VC_G and VC_S mean that the visual comparison detected growth (G) or stability (S), respectively. (ZIP) [file pmed.1002810.s001.zip › 7474_t1_CAD_G_VC_S.pdf]

Case Number:7735; Time 0 (Baseline).

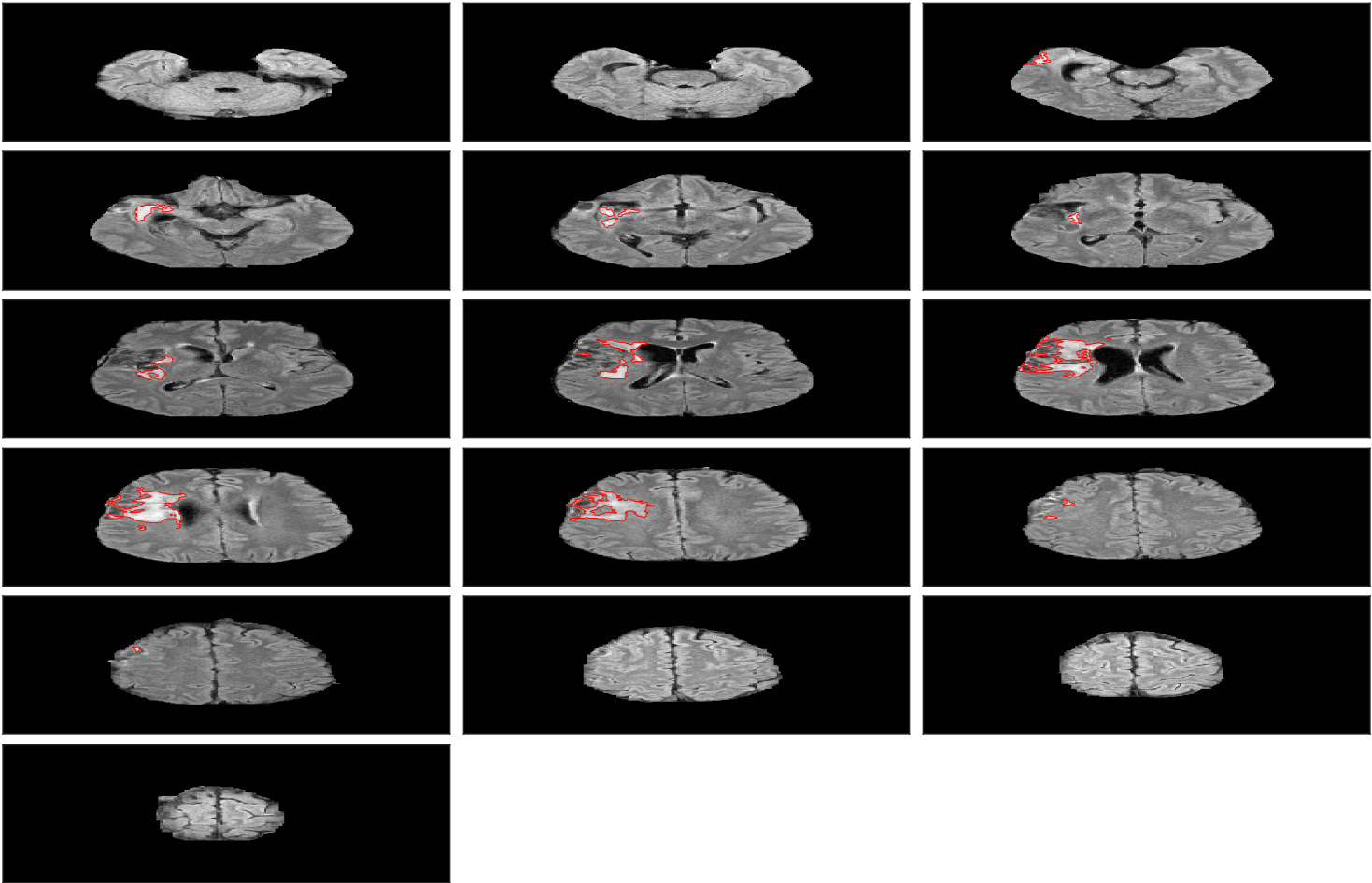

Supplement: S1 Data — The .zip file includes .pdf files labeled as follows, using case number 4224 as an example: 4224_t0.pdf includes the MRI data with segmentation of case number 4224 at baseline. 4224_t1_CAD_G_VC_S.pdf includes the MRI data with segmentation of case number 4224 at time 1, i.e., the time point of growth detection by the change-of-point method when the time point was different from the most recent MRI. 4224_tend_CAD_G_VC_G.pdf includes the MRI data with segmentation of case number 4224 at the most recent MRI. CAD_G and CAD_S mean that the change-of-point method predicted growth (G) or stability (S), respectively. VC_G and VC_S mean that the visual comparison detected growth (G) or stability (S), respectively. (ZIP) [file pmed.1002810.s001.zip › 7735_t0.pdf]

Case Number:7756; Time 0 (Baseline).

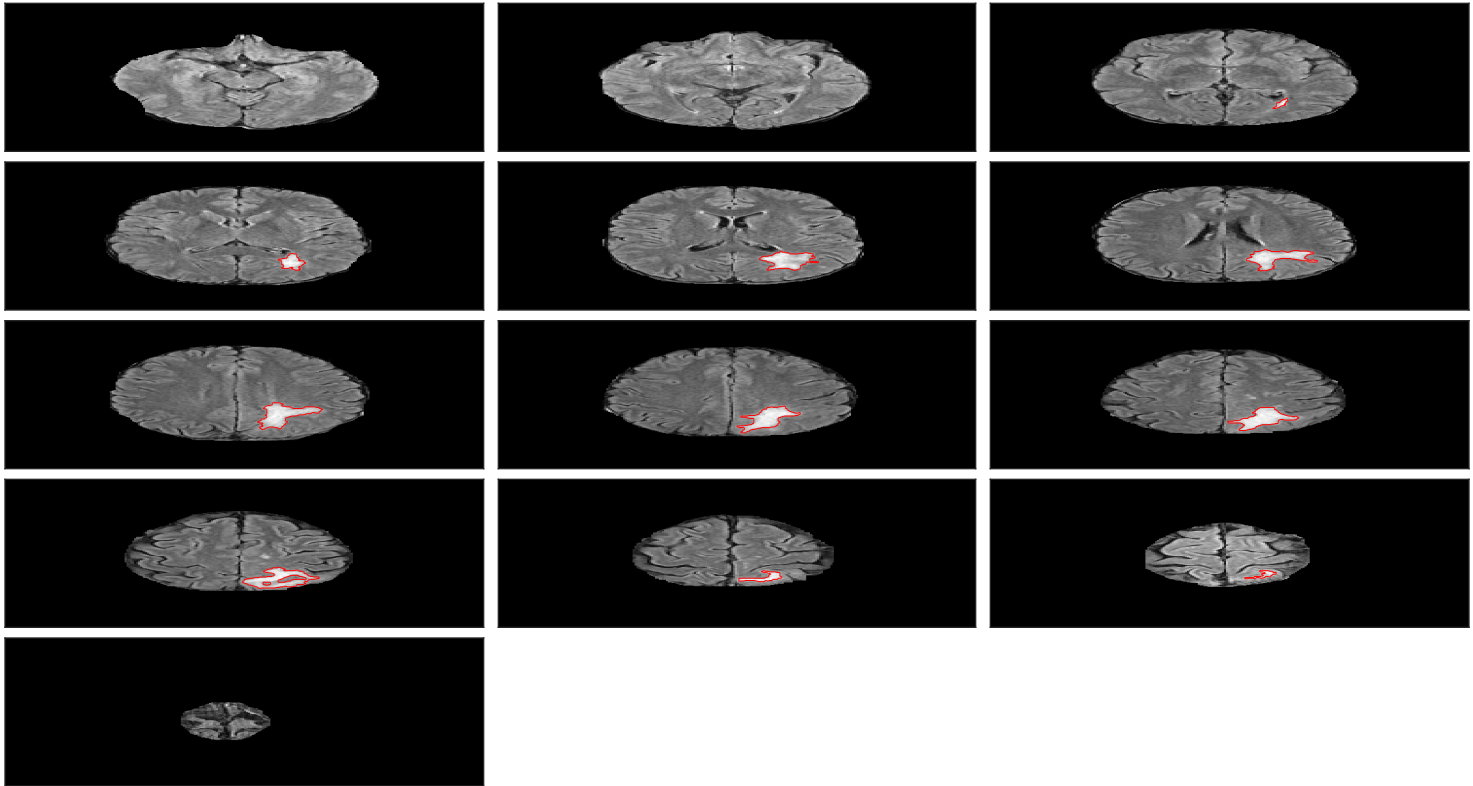

Supplement: S1 Data — The .zip file includes .pdf files labeled as follows, using case number 4224 as an example: 4224_t0.pdf includes the MRI data with segmentation of case number 4224 at baseline. 4224_t1_CAD_G_VC_S.pdf includes the MRI data with segmentation of case number 4224 at time 1, i.e., the time point of growth detection by the change-of-point method when the time point was different from the most recent MRI. 4224_tend_CAD_G_VC_G.pdf includes the MRI data with segmentation of case number 4224 at the most recent MRI. CAD_G and CAD_S mean that the change-of-point method predicted growth (G) or stability (S), respectively. VC_G and VC_S mean that the visual comparison detected growth (G) or stability (S), respectively. (ZIP) [file pmed.1002810.s001.zip › 7756_t0.pdf]

Case Number:7727; Time 0 (Baseline).

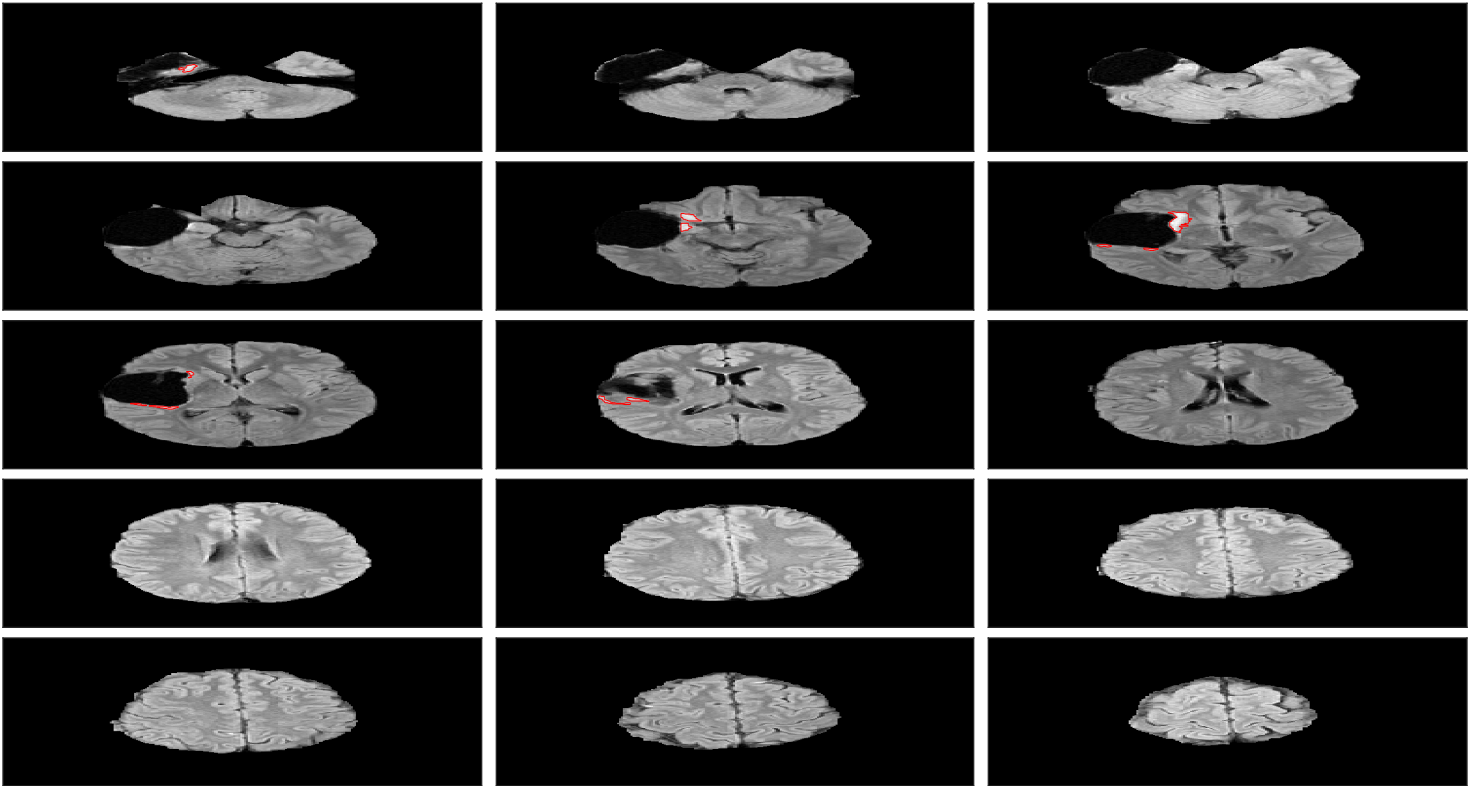

Supplement: S1 Data — The .zip file includes .pdf files labeled as follows, using case number 4224 as an example: 4224_t0.pdf includes the MRI data with segmentation of case number 4224 at baseline. 4224_t1_CAD_G_VC_S.pdf includes the MRI data with segmentation of case number 4224 at time 1, i.e., the time point of growth detection by the change-of-point method when the time point was different from the most recent MRI. 4224_tend_CAD_G_VC_G.pdf includes the MRI data with segmentation of case number 4224 at the most recent MRI. CAD_G and CAD_S mean that the change-of-point method predicted growth (G) or stability (S), respectively. VC_G and VC_S mean that the visual comparison detected growth (G) or stability (S), respectively. (ZIP) [file pmed.1002810.s001.zip › 7727_t0.pdf]

Case Number:7713; TIME 1; CAD --> GROWTH; VC --> STABLE; Interval From Baseline =8 months.

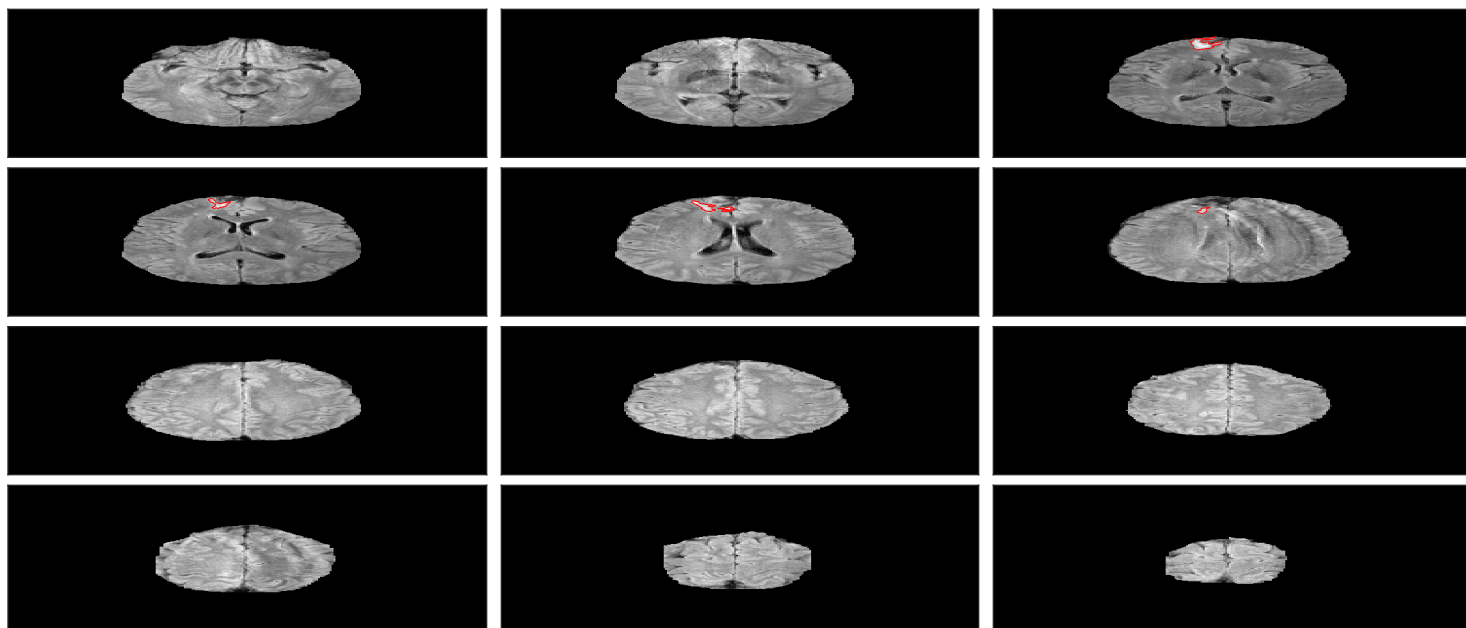

Supplement: S1 Data — The .zip file includes .pdf files labeled as follows, using case number 4224 as an example: 4224_t0.pdf includes the MRI data with segmentation of case number 4224 at baseline. 4224_t1_CAD_G_VC_S.pdf includes the MRI data with segmentation of case number 4224 at time 1, i.e., the time point of growth detection by the change-of-point method when the time point was different from the most recent MRI. 4224_tend_CAD_G_VC_G.pdf includes the MRI data with segmentation of case number 4224 at the most recent MRI. CAD_G and CAD_S mean that the change-of-point method predicted growth (G) or stability (S), respectively. VC_G and VC_S mean that the visual comparison detected growth (G) or stability (S), respectively. (ZIP) [file pmed.1002810.s001.zip › 7713_t1_CAD_G_VC_S.pdf]

Case Number:7490; Time 0 (Baseline).

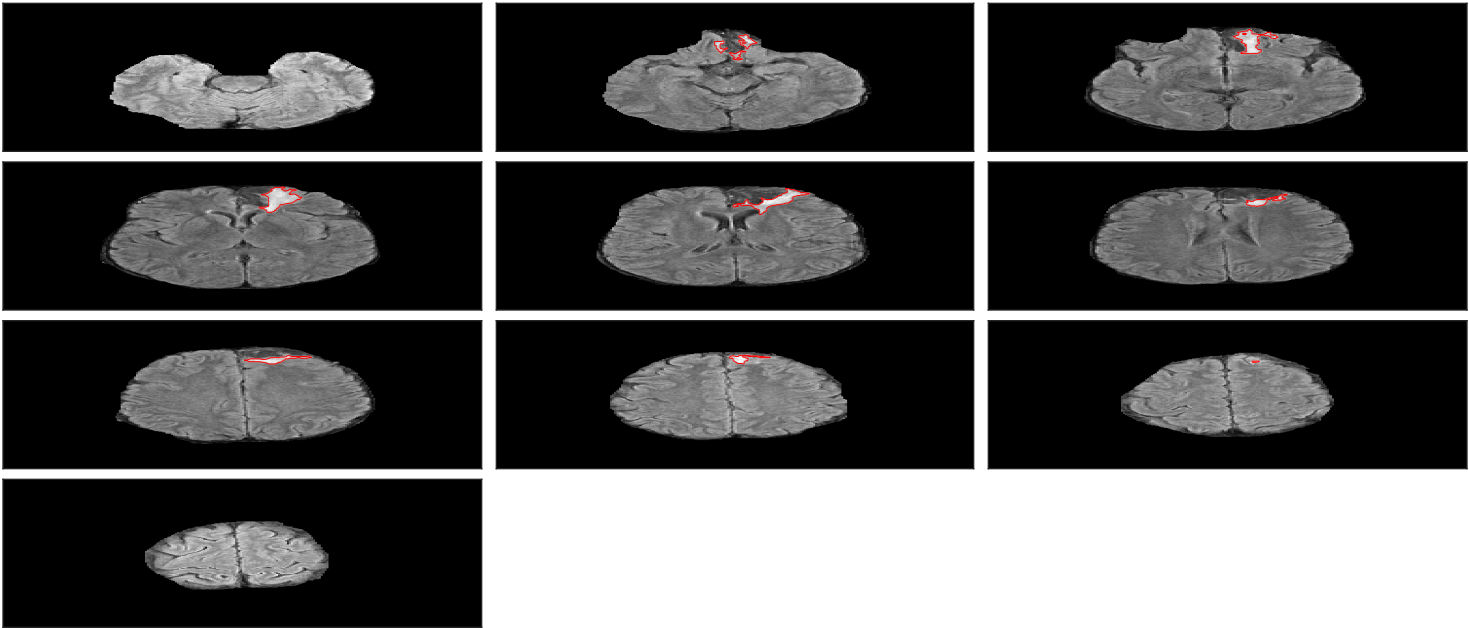

Supplement: S1 Data — The .zip file includes .pdf files labeled as follows, using case number 4224 as an example: 4224_t0.pdf includes the MRI data with segmentation of case number 4224 at baseline. 4224_t1_CAD_G_VC_S.pdf includes the MRI data with segmentation of case number 4224 at time 1, i.e., the time point of growth detection by the change-of-point method when the time point was different from the most recent MRI. 4224_tend_CAD_G_VC_G.pdf includes the MRI data with segmentation of case number 4224 at the most recent MRI. CAD_G and CAD_S mean that the change-of-point method predicted growth (G) or stability (S), respectively. VC_G and VC_S mean that the visual comparison detected growth (G) or stability (S), respectively. (ZIP) [file pmed.1002810.s001.zip › 7490_t0.pdf]

Case Number:4385; LAST MRI; CAD --> GROWTH; VC --> GROWTH; Interval From Baseline =24 months.

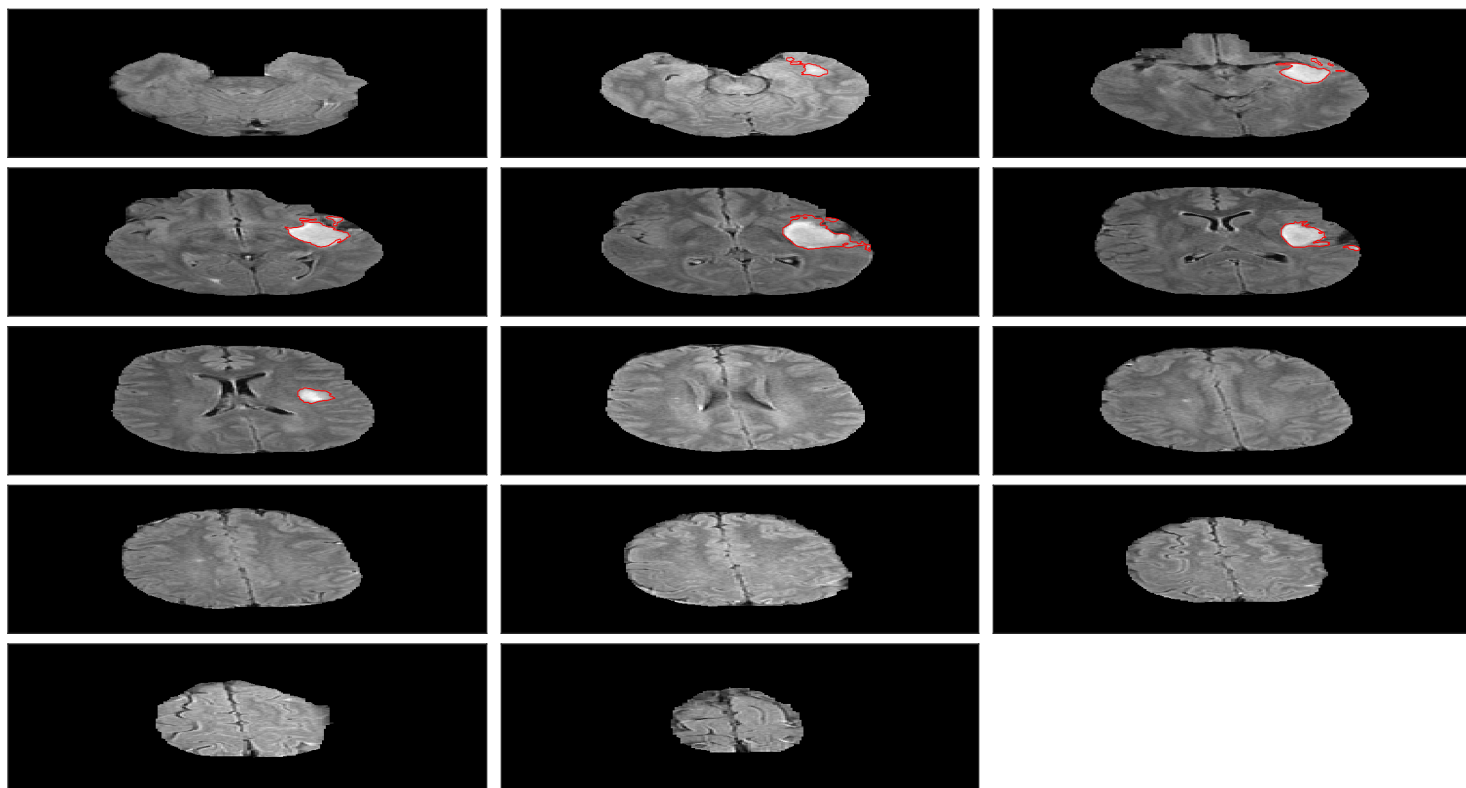

Supplement: S1 Data — The .zip file includes .pdf files labeled as follows, using case number 4224 as an example: 4224_t0.pdf includes the MRI data with segmentation of case number 4224 at baseline. 4224_t1_CAD_G_VC_S.pdf includes the MRI data with segmentation of case number 4224 at time 1, i.e., the time point of growth detection by the change-of-point method when the time point was different from the most recent MRI. 4224_tend_CAD_G_VC_G.pdf includes the MRI data with segmentation of case number 4224 at the most recent MRI. CAD_G and CAD_S mean that the change-of-point method predicted growth (G) or stability (S), respectively. VC_G and VC_S mean that the visual comparison detected growth (G) or stability (S), respectively. (ZIP) [file pmed.1002810.s001.zip › 4385_tend_CAD_G_VC_G.pdf]

Case Number:4385; TIME 1; CAD --> GROWTH; VC --> STABLE; Interval From Baseline =9 months.

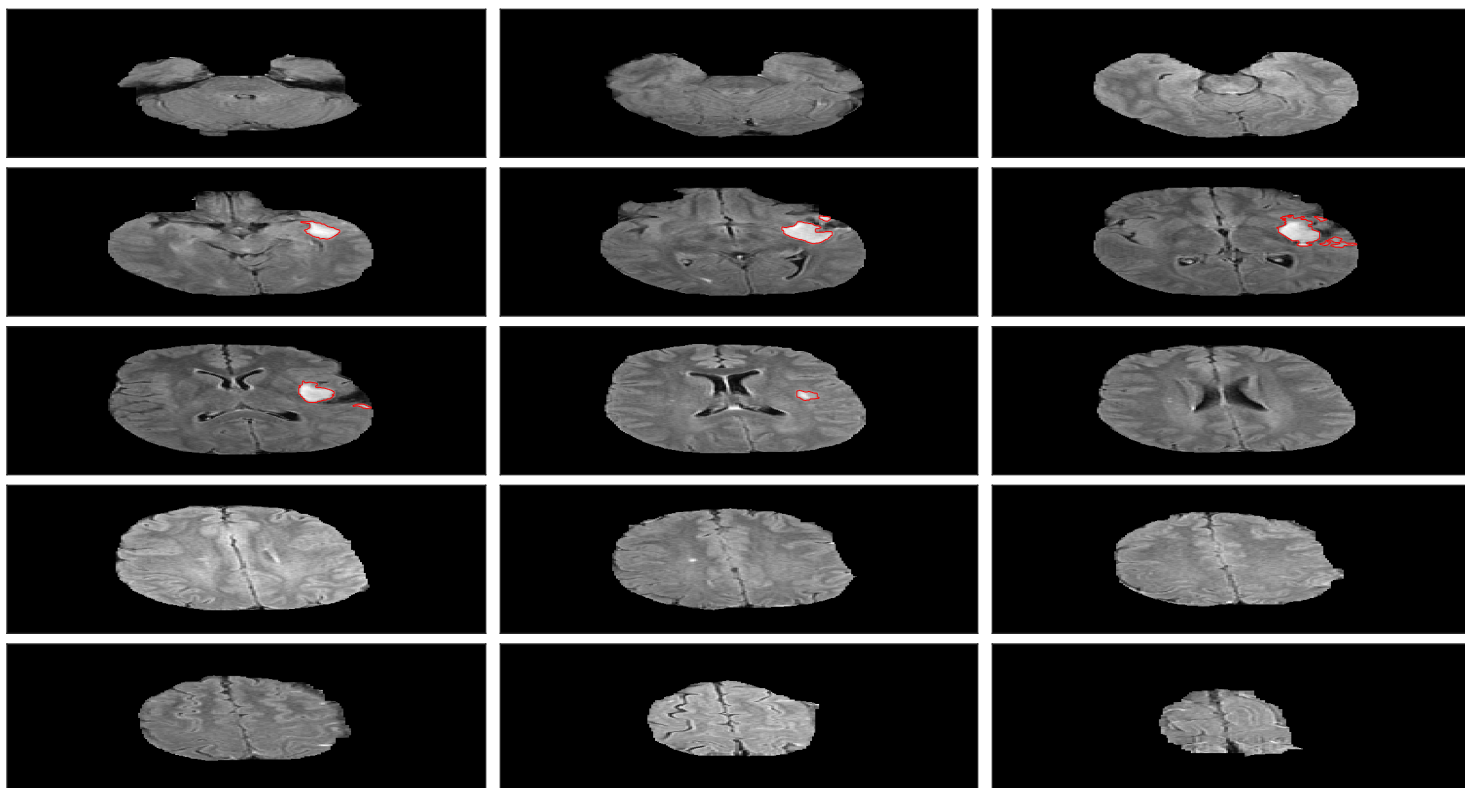

Supplement: S1 Data — The .zip file includes .pdf files labeled as follows, using case number 4224 as an example: 4224_t0.pdf includes the MRI data with segmentation of case number 4224 at baseline. 4224_t1_CAD_G_VC_S.pdf includes the MRI data with segmentation of case number 4224 at time 1, i.e., the time point of growth detection by the change-of-point method when the time point was different from the most recent MRI. 4224_tend_CAD_G_VC_G.pdf includes the MRI data with segmentation of case number 4224 at the most recent MRI. CAD_G and CAD_S mean that the change-of-point method predicted growth (G) or stability (S), respectively. VC_G and VC_S mean that the visual comparison detected growth (G) or stability (S), respectively. (ZIP) [file pmed.1002810.s001.zip › 4385_t1_CAD_G_VC_S.pdf]

Case Number:7494; TIME 1; CAD --> GROWTH; VC --> STABLE; Interval From Baseline =13 months.

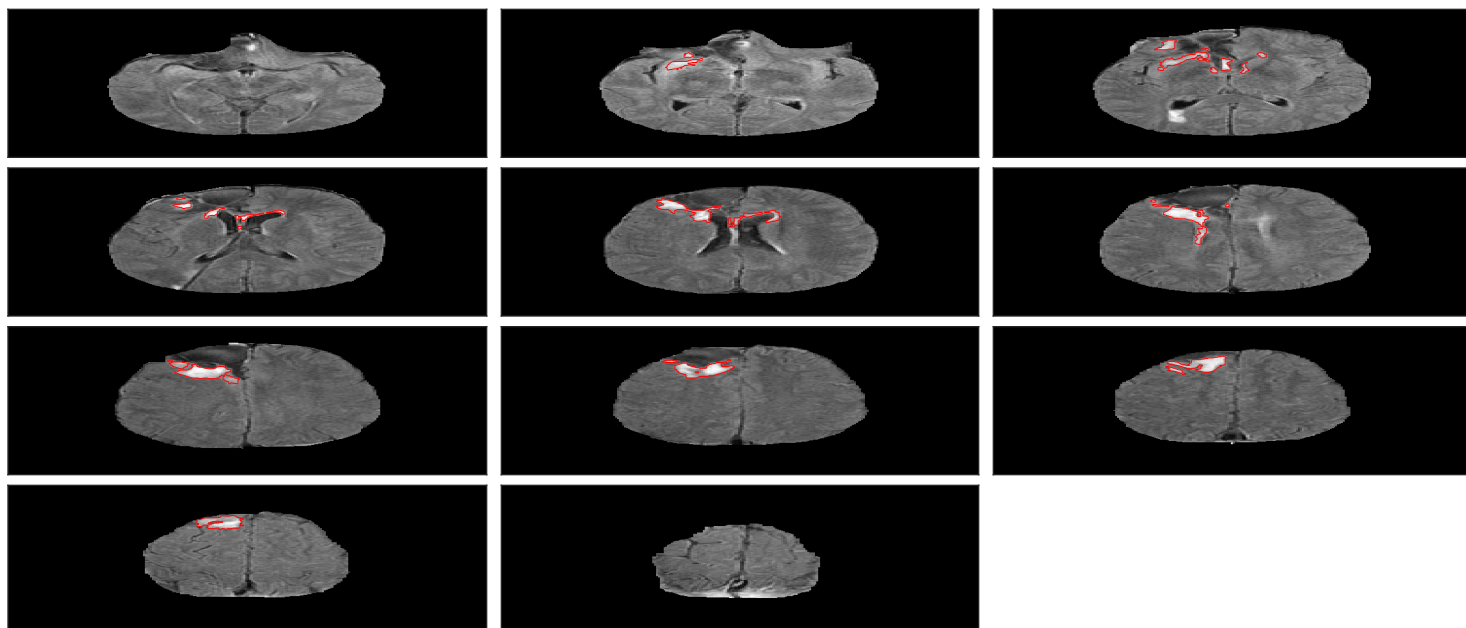

Supplement: S1 Data — The .zip file includes .pdf files labeled as follows, using case number 4224 as an example: 4224_t0.pdf includes the MRI data with segmentation of case number 4224 at baseline. 4224_t1_CAD_G_VC_S.pdf includes the MRI data with segmentation of case number 4224 at time 1, i.e., the time point of growth detection by the change-of-point method when the time point was different from the most recent MRI. 4224_tend_CAD_G_VC_G.pdf includes the MRI data with segmentation of case number 4224 at the most recent MRI. CAD_G and CAD_S mean that the change-of-point method predicted growth (G) or stability (S), respectively. VC_G and VC_S mean that the visual comparison detected growth (G) or stability (S), respectively. (ZIP) [file pmed.1002810.s001.zip › 7494_t1_CAD_G_VC_S.pdf]

Case Number:7711; Time 0 (Baseline).

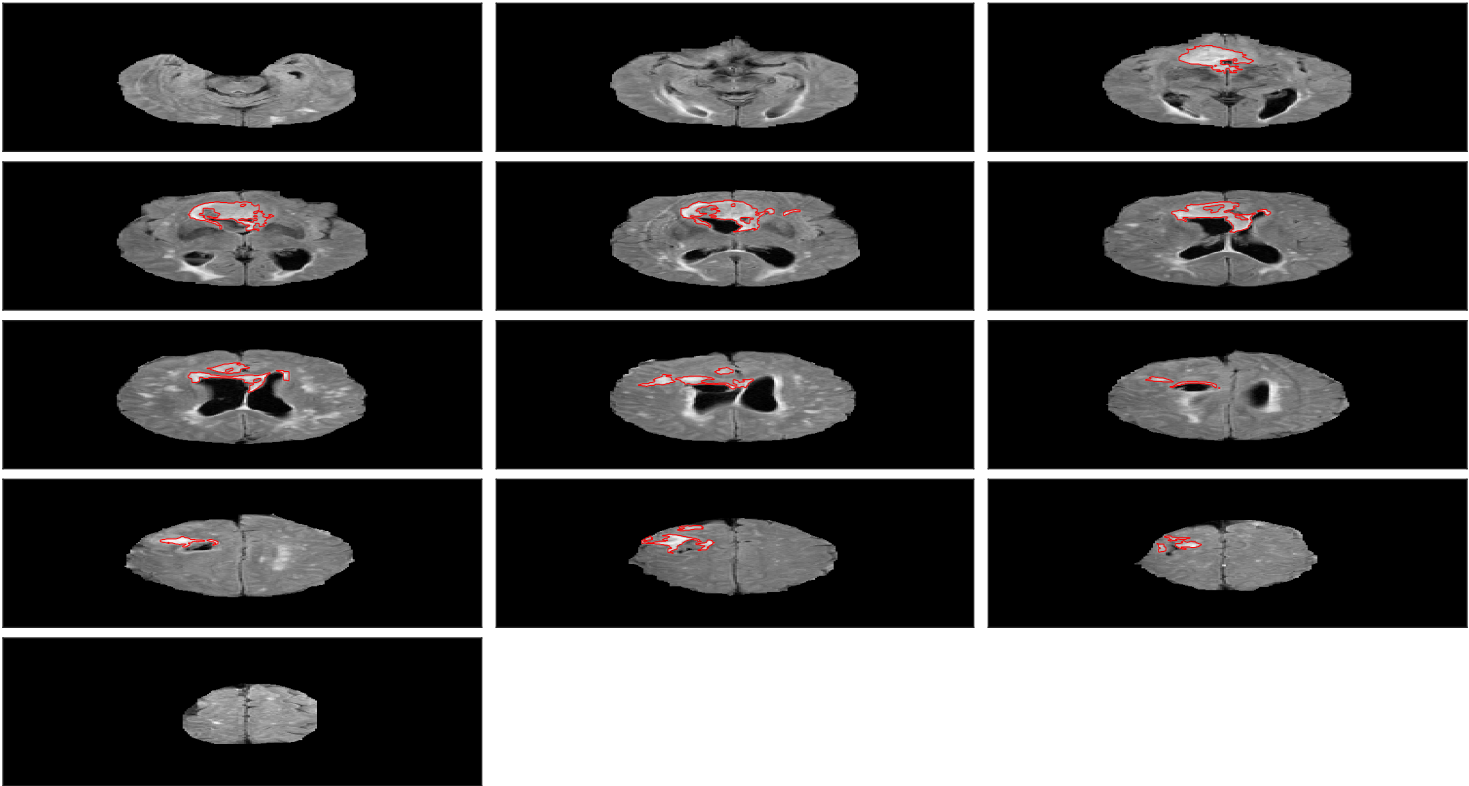

Supplement: S1 Data — The .zip file includes .pdf files labeled as follows, using case number 4224 as an example: 4224_t0.pdf includes the MRI data with segmentation of case number 4224 at baseline. 4224_t1_CAD_G_VC_S.pdf includes the MRI data with segmentation of case number 4224 at time 1, i.e., the time point of growth detection by the change-of-point method when the time point was different from the most recent MRI. 4224_tend_CAD_G_VC_G.pdf includes the MRI data with segmentation of case number 4224 at the most recent MRI. CAD_G and CAD_S mean that the change-of-point method predicted growth (G) or stability (S), respectively. VC_G and VC_S mean that the visual comparison detected growth (G) or stability (S), respectively. (ZIP) [file pmed.1002810.s001.zip › 7711_t0.pdf]

Case Number:7733; LAST MRI; CAD --> GROWTH; VC --> STABLE; Interval From Baseline =89 months.

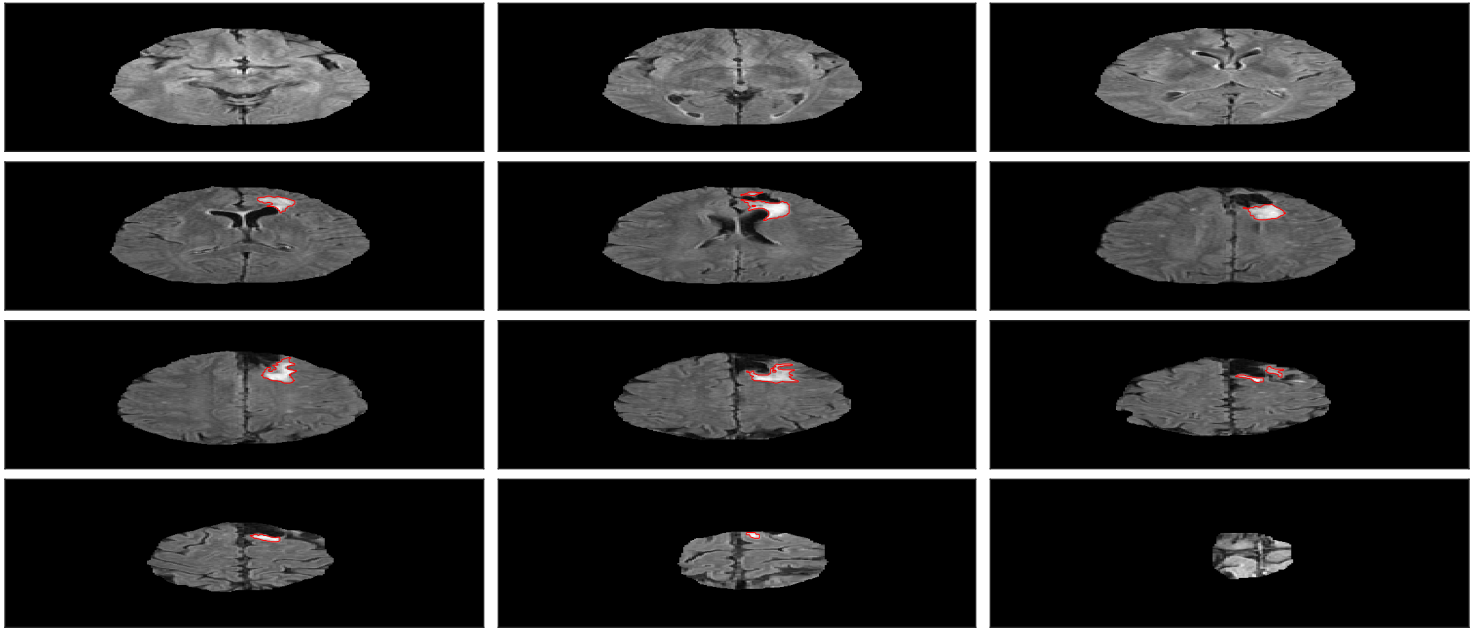

Supplement: S1 Data — The .zip file includes .pdf files labeled as follows, using case number 4224 as an example: 4224_t0.pdf includes the MRI data with segmentation of case number 4224 at baseline. 4224_t1_CAD_G_VC_S.pdf includes the MRI data with segmentation of case number 4224 at time 1, i.e., the time point of growth detection by the change-of-point method when the time point was different from the most recent MRI. 4224_tend_CAD_G_VC_G.pdf includes the MRI data with segmentation of case number 4224 at the most recent MRI. CAD_G and CAD_S mean that the change-of-point method predicted growth (G) or stability (S), respectively. VC_G and VC_S mean that the visual comparison detected growth (G) or stability (S), respectively. (ZIP) [file pmed.1002810.s001.zip › 7733_tend_CAD_G_VC_S.pdf]

Case Number:7741; TIME 1; CAD --> GROWTH; VC --> STABLE; Interval From Baseline =45 months.

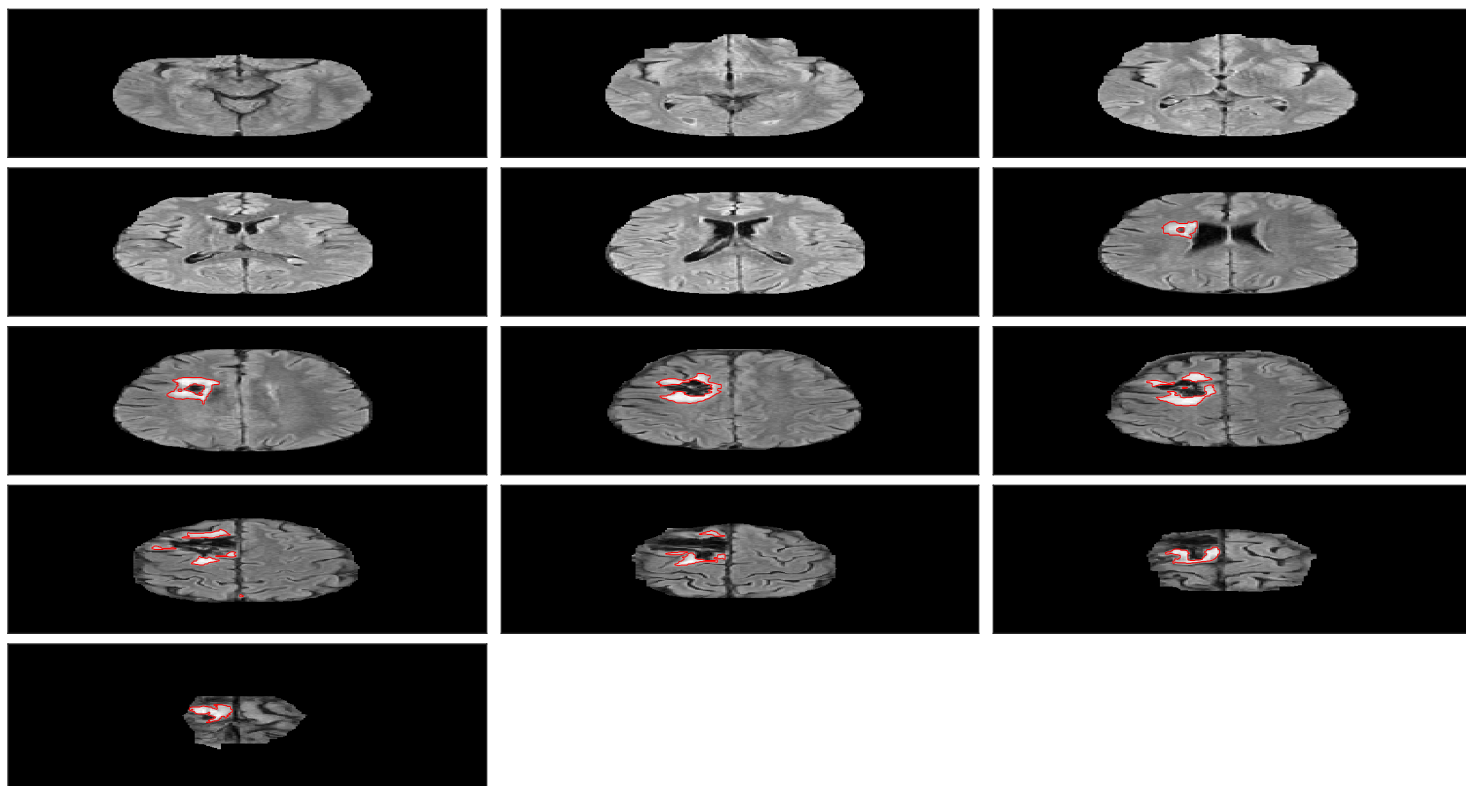

Supplement: S1 Data — The .zip file includes .pdf files labeled as follows, using case number 4224 as an example: 4224_t0.pdf includes the MRI data with segmentation of case number 4224 at baseline. 4224_t1_CAD_G_VC_S.pdf includes the MRI data with segmentation of case number 4224 at time 1, i.e., the time point of growth detection by the change-of-point method when the time point was different from the most recent MRI. 4224_tend_CAD_G_VC_G.pdf includes the MRI data with segmentation of case number 4224 at the most recent MRI. CAD_G and CAD_S mean that the change-of-point method predicted growth (G) or stability (S), respectively. VC_G and VC_S mean that the visual comparison detected growth (G) or stability (S), respectively. (ZIP) [file pmed.1002810.s001.zip › 7741_t1_CAD_G_VC_S.pdf]

Case Number:7470; TIME 1; CAD --> GROWTH; VC --> STABLE; Interval From Baseline =10 months.

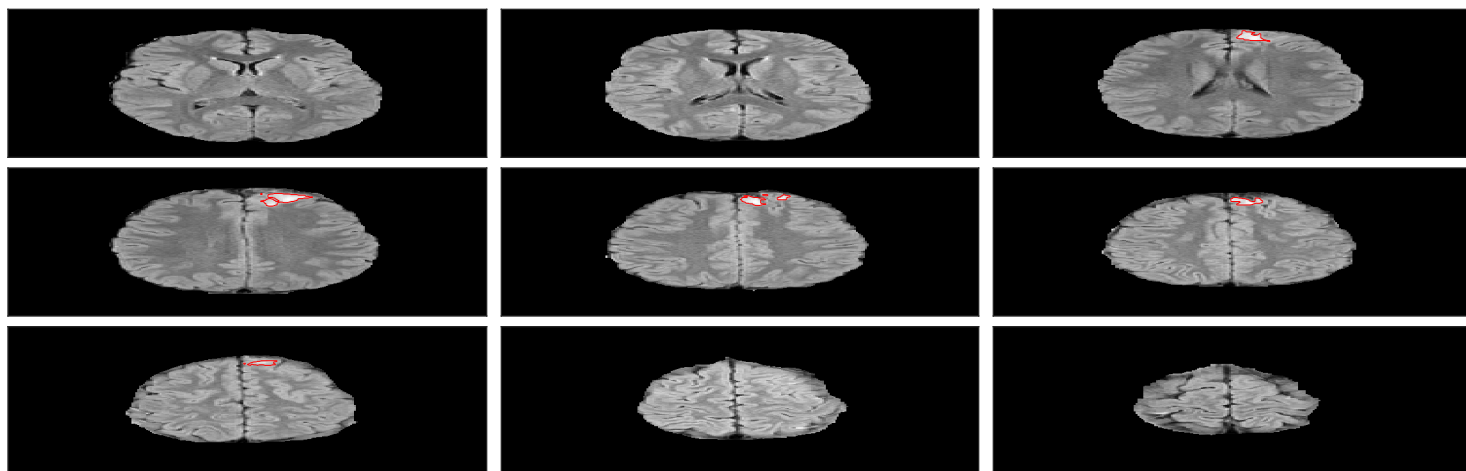

Supplement: S1 Data — The .zip file includes .pdf files labeled as follows, using case number 4224 as an example: 4224_t0.pdf includes the MRI data with segmentation of case number 4224 at baseline. 4224_t1_CAD_G_VC_S.pdf includes the MRI data with segmentation of case number 4224 at time 1, i.e., the time point of growth detection by the change-of-point method when the time point was different from the most recent MRI. 4224_tend_CAD_G_VC_G.pdf includes the MRI data with segmentation of case number 4224 at the most recent MRI. CAD_G and CAD_S mean that the change-of-point method predicted growth (G) or stability (S), respectively. VC_G and VC_S mean that the visual comparison detected growth (G) or stability (S), respectively. (ZIP) [file pmed.1002810.s001.zip › 7470_t1_CAD_G_VC_S.pdf]

Case Number:4384; LAST MRI; CAD --> GROWTH; VC --> GROWTH; Interval From Baseline =47 months.

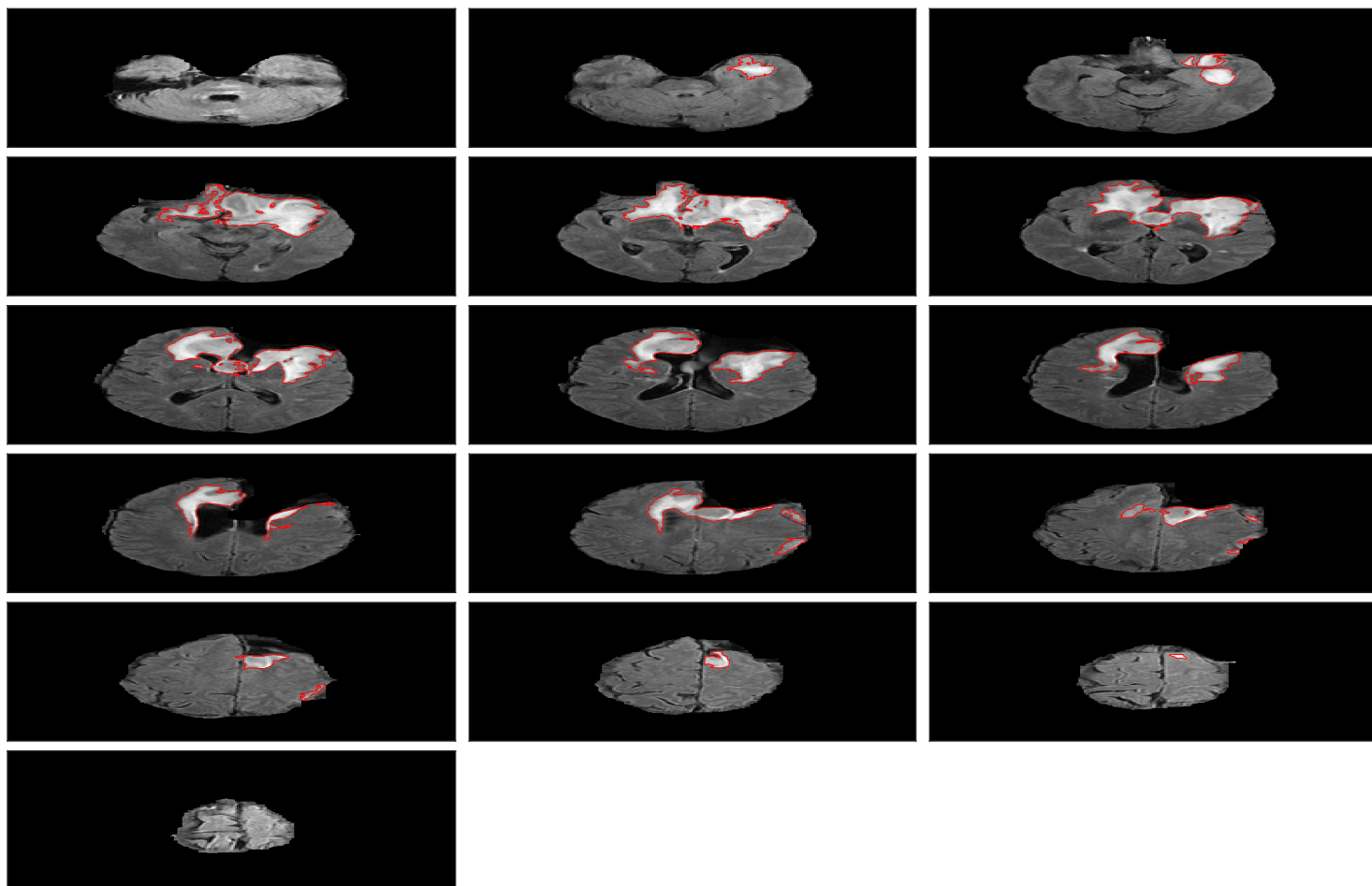

Supplement: S1 Data — The .zip file includes .pdf files labeled as follows, using case number 4224 as an example: 4224_t0.pdf includes the MRI data with segmentation of case number 4224 at baseline. 4224_t1_CAD_G_VC_S.pdf includes the MRI data with segmentation of case number 4224 at time 1, i.e., the time point of growth detection by the change-of-point method when the time point was different from the most recent MRI. 4224_tend_CAD_G_VC_G.pdf includes the MRI data with segmentation of case number 4224 at the most recent MRI. CAD_G and CAD_S mean that the change-of-point method predicted growth (G) or stability (S), respectively. VC_G and VC_S mean that the visual comparison detected growth (G) or stability (S), respectively. (ZIP) [file pmed.1002810.s001.zip › 4384_tend_CAD_G_VC_G.pdf]

Case Number:7469; TIME 1; CAD --> GROWTH; VC --> STABLE; Interval From Baseline =20 months.

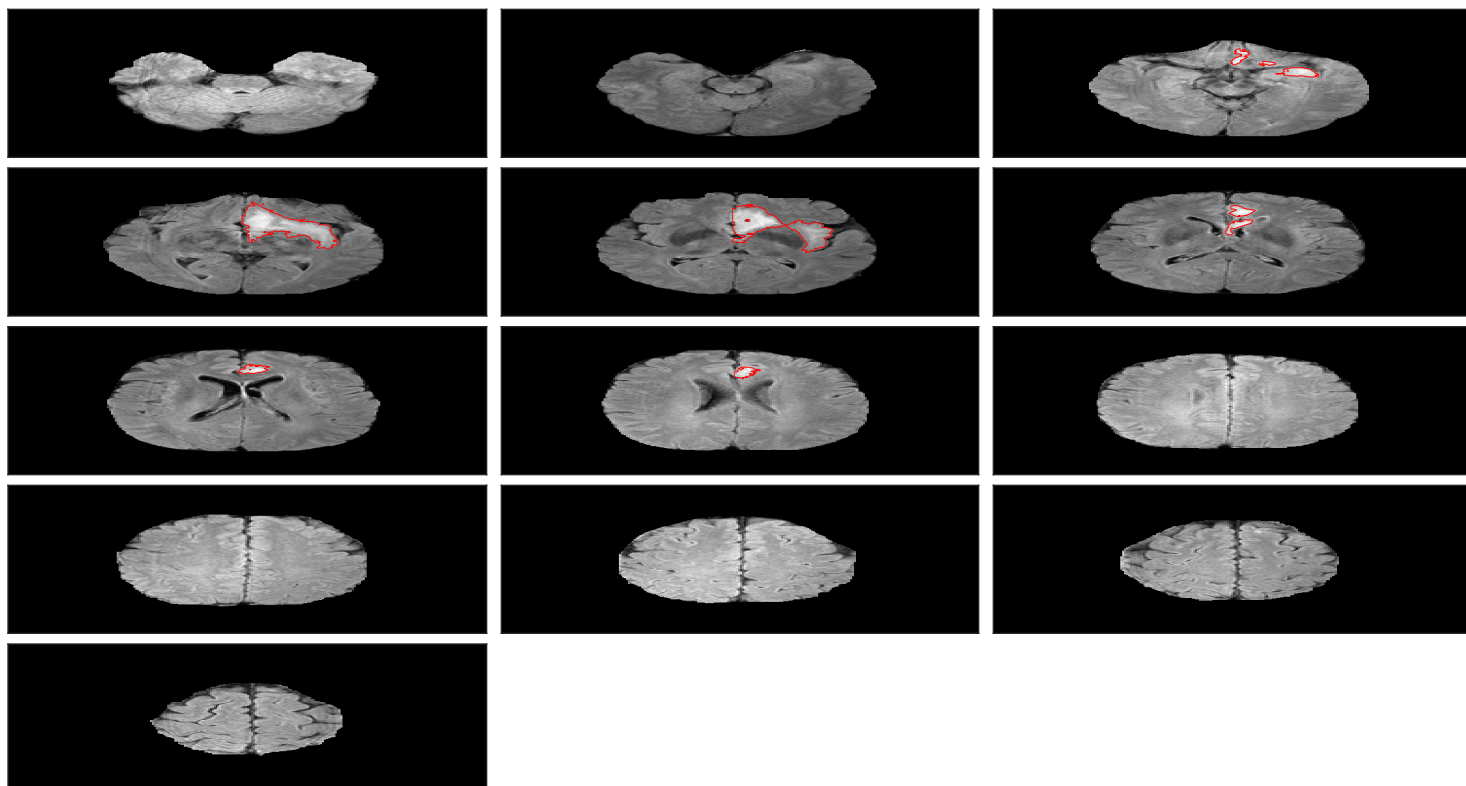

Supplement: S1 Data — The .zip file includes .pdf files labeled as follows, using case number 4224 as an example: 4224_t0.pdf includes the MRI data with segmentation of case number 4224 at baseline. 4224_t1_CAD_G_VC_S.pdf includes the MRI data with segmentation of case number 4224 at time 1, i.e., the time point of growth detection by the change-of-point method when the time point was different from the most recent MRI. 4224_tend_CAD_G_VC_G.pdf includes the MRI data with segmentation of case number 4224 at the most recent MRI. CAD_G and CAD_S mean that the change-of-point method predicted growth (G) or stability (S), respectively. VC_G and VC_S mean that the visual comparison detected growth (G) or stability (S), respectively. (ZIP) [file pmed.1002810.s001.zip › 7469_t1_CAD_G_VC_S.pdf]

Case Number:4387; LAST MRI; CAD --> GROWTH; VC --> GROWTH; Interval From Baseline =73 months.

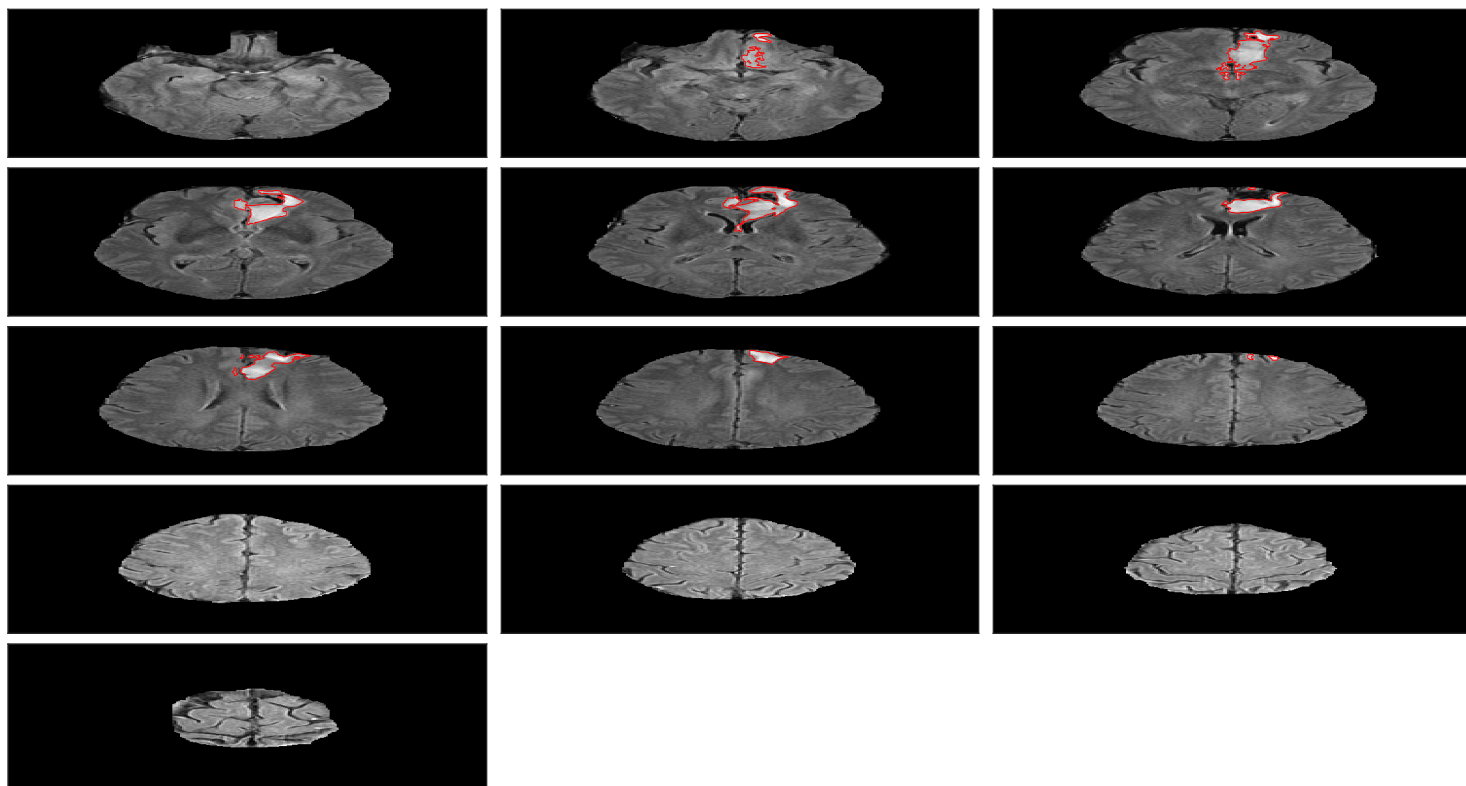

Supplement: S1 Data — The .zip file includes .pdf files labeled as follows, using case number 4224 as an example: 4224_t0.pdf includes the MRI data with segmentation of case number 4224 at baseline. 4224_t1_CAD_G_VC_S.pdf includes the MRI data with segmentation of case number 4224 at time 1, i.e., the time point of growth detection by the change-of-point method when the time point was different from the most recent MRI. 4224_tend_CAD_G_VC_G.pdf includes the MRI data with segmentation of case number 4224 at the most recent MRI. CAD_G and CAD_S mean that the change-of-point method predicted growth (G) or stability (S), respectively. VC_G and VC_S mean that the visual comparison detected growth (G) or stability (S), respectively. (ZIP) [file pmed.1002810.s001.zip › 4387_tend_CAD_G_VC_G.pdf]

Case Number:7734; LAST MRI; CAD --> STABLE; VC --> STABLE; Interval From Baseline =19 months.

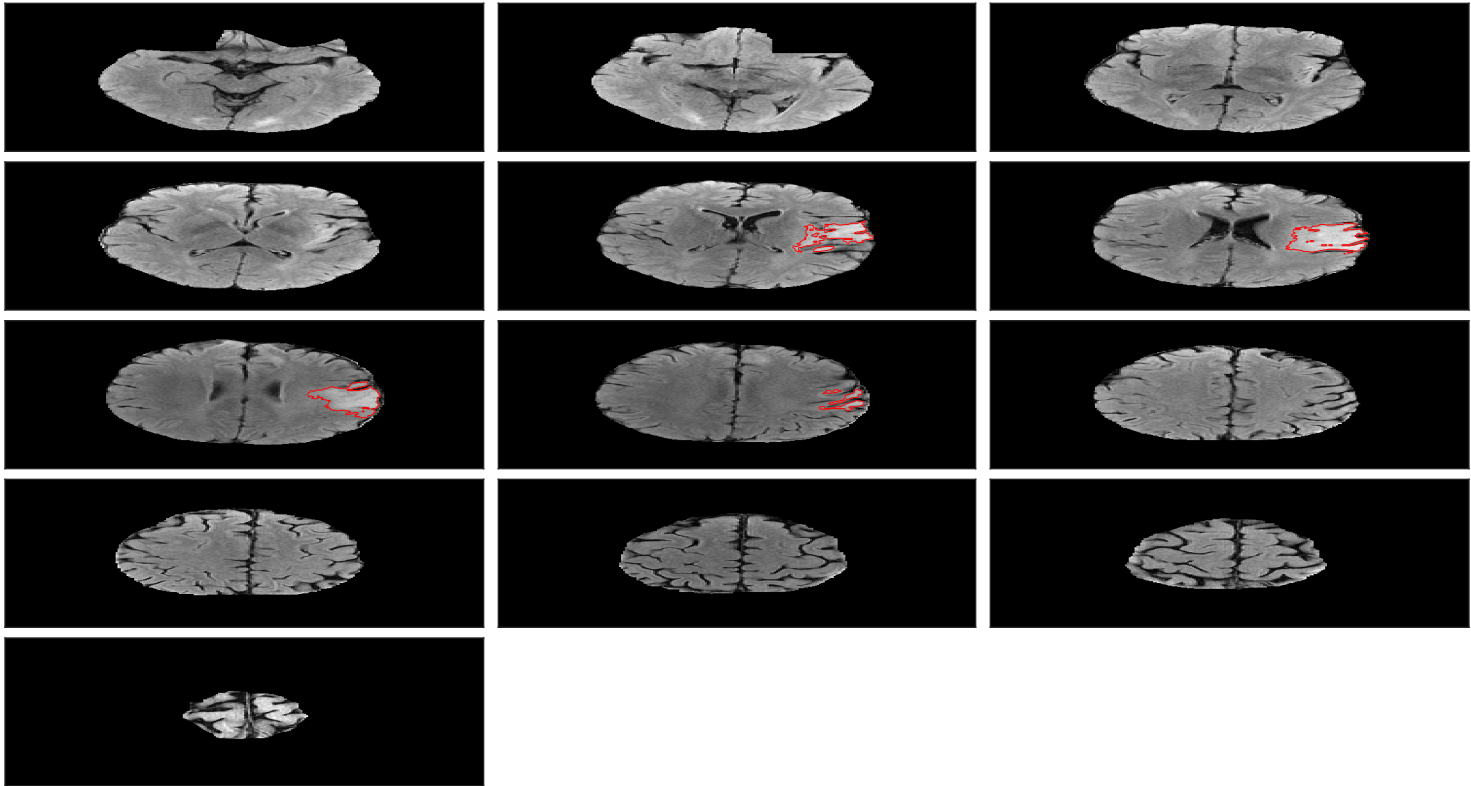

Supplement: S1 Data — The .zip file includes .pdf files labeled as follows, using case number 4224 as an example: 4224_t0.pdf includes the MRI data with segmentation of case number 4224 at baseline. 4224_t1_CAD_G_VC_S.pdf includes the MRI data with segmentation of case number 4224 at time 1, i.e., the time point of growth detection by the change-of-point method when the time point was different from the most recent MRI. 4224_tend_CAD_G_VC_G.pdf includes the MRI data with segmentation of case number 4224 at the most recent MRI. CAD_G and CAD_S mean that the change-of-point method predicted growth (G) or stability (S), respectively. VC_G and VC_S mean that the visual comparison detected growth (G) or stability (S), respectively. (ZIP) [file pmed.1002810.s001.zip › 7734_tend_CAD_S_VC_S.pdf]

Case Number:7491; TIME 1; CAD --> GROWTH; VC --> STABLE; Interval From Baseline =30 months.

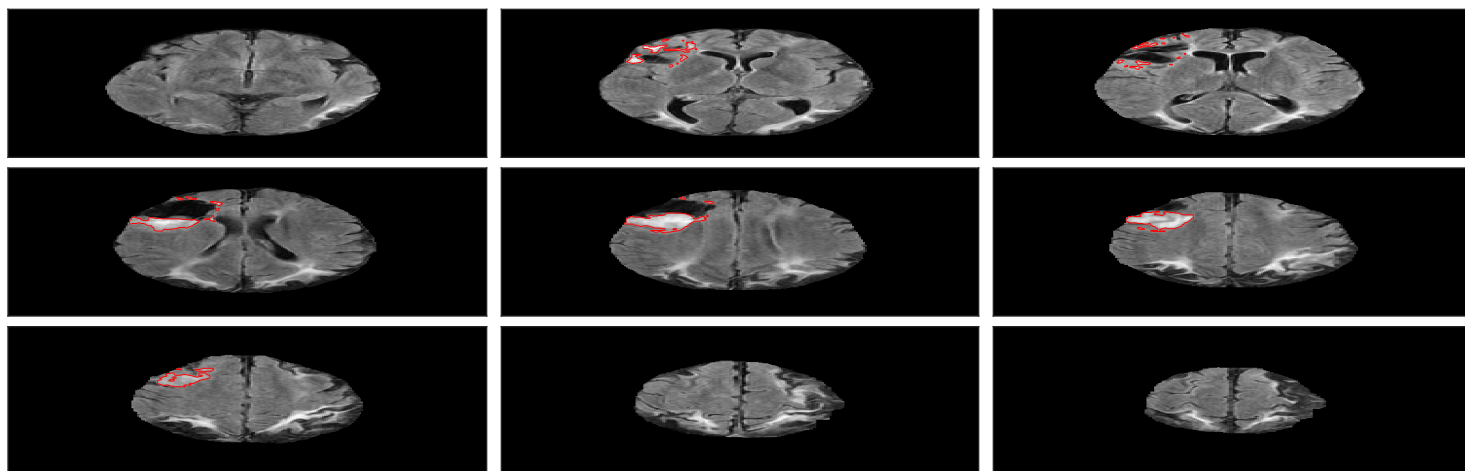

Supplement: S1 Data — The .zip file includes .pdf files labeled as follows, using case number 4224 as an example: 4224_t0.pdf includes the MRI data with segmentation of case number 4224 at baseline. 4224_t1_CAD_G_VC_S.pdf includes the MRI data with segmentation of case number 4224 at time 1, i.e., the time point of growth detection by the change-of-point method when the time point was different from the most recent MRI. 4224_tend_CAD_G_VC_G.pdf includes the MRI data with segmentation of case number 4224 at the most recent MRI. CAD_G and CAD_S mean that the change-of-point method predicted growth (G) or stability (S), respectively. VC_G and VC_S mean that the visual comparison detected growth (G) or stability (S), respectively. (ZIP) [file pmed.1002810.s001.zip › 7491_t1_CAD_G_VC_S.pdf]

Case Number:7471; Time 0 (Baseline).

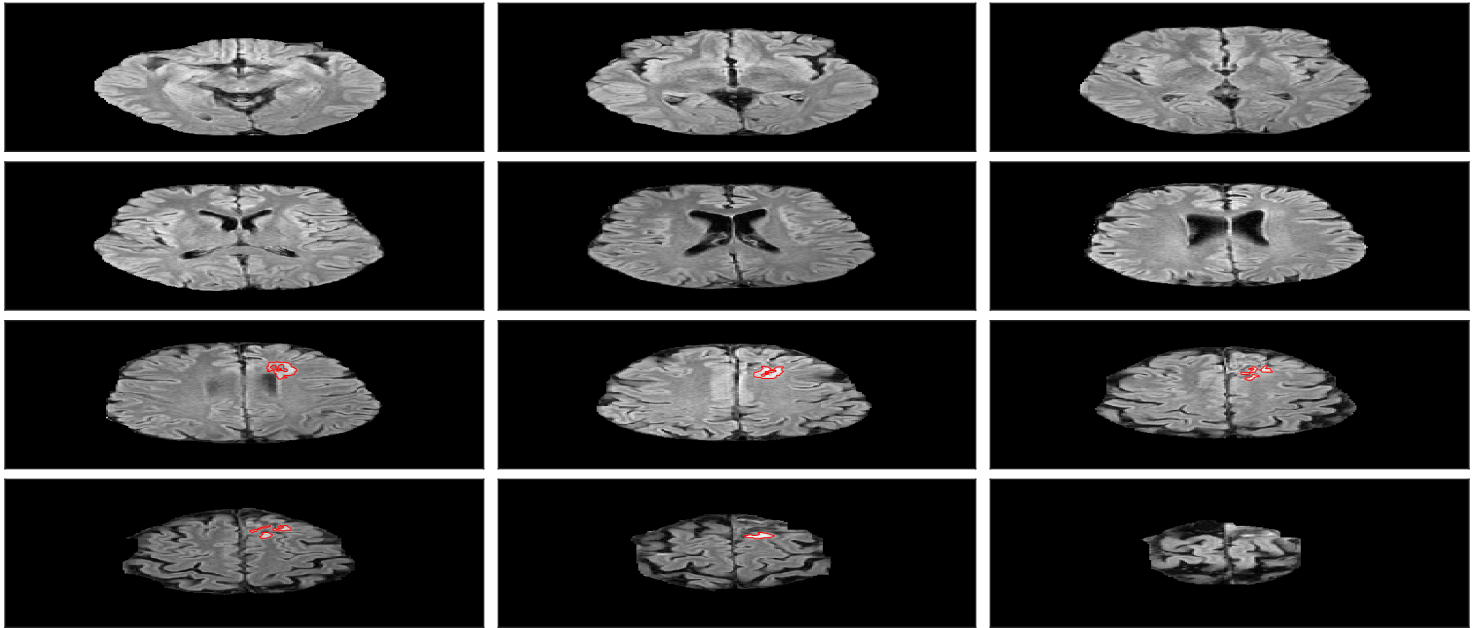

Supplement: S1 Data — The .zip file includes .pdf files labeled as follows, using case number 4224 as an example: 4224_t0.pdf includes the MRI data with segmentation of case number 4224 at baseline. 4224_t1_CAD_G_VC_S.pdf includes the MRI data with segmentation of case number 4224 at time 1, i.e., the time point of growth detection by the change-of-point method when the time point was different from the most recent MRI. 4224_tend_CAD_G_VC_G.pdf includes the MRI data with segmentation of case number 4224 at the most recent MRI. CAD_G and CAD_S mean that the change-of-point method predicted growth (G) or stability (S), respectively. VC_G and VC_S mean that the visual comparison detected growth (G) or stability (S), respectively. (ZIP) [file pmed.1002810.s001.zip › 7471_t0.pdf]

Case Number:4386; LAST MRI; CAD --> GROWTH; VC --> GROWTH; Interval From Baseline =32 months.

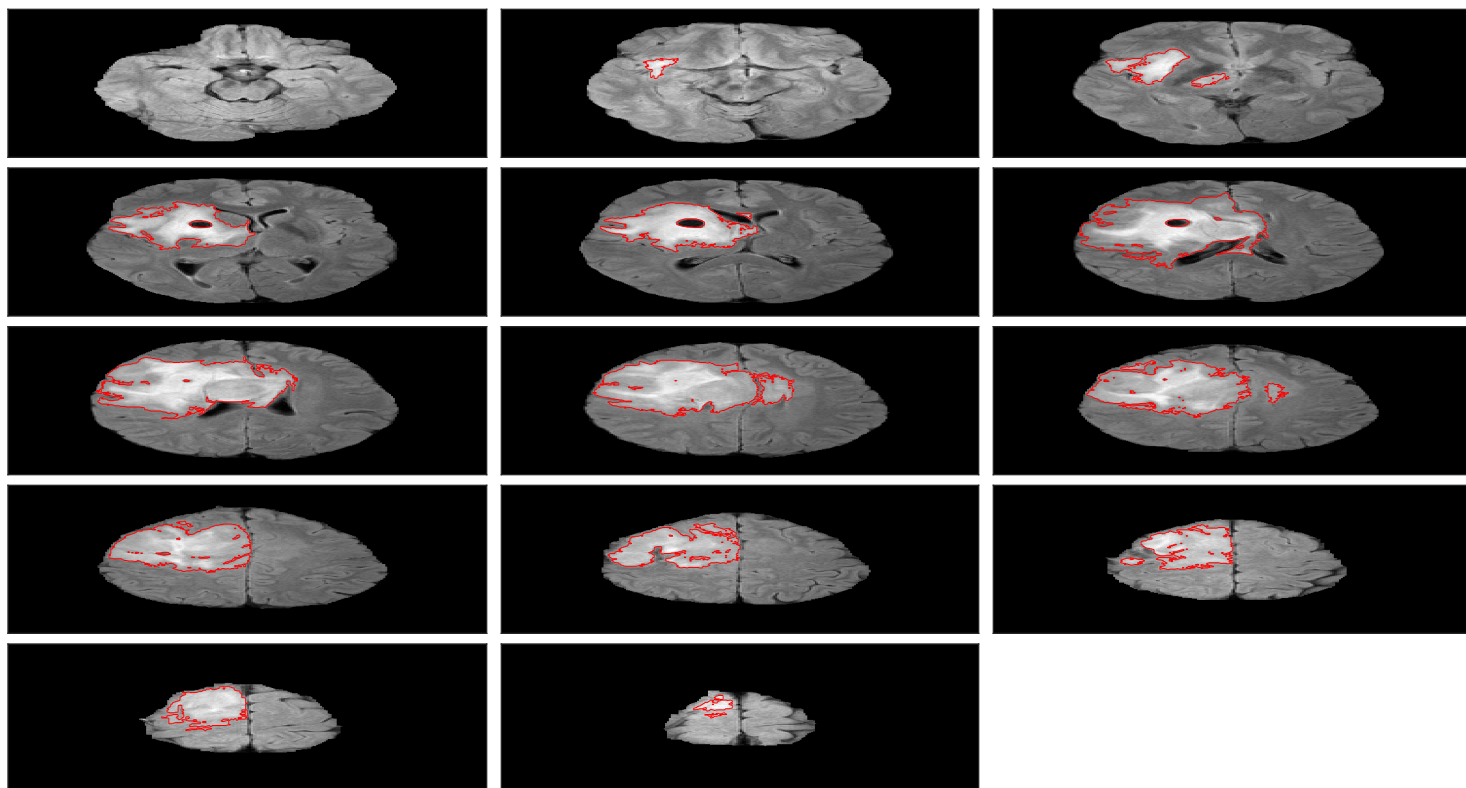

Supplement: S1 Data — The .zip file includes .pdf files labeled as follows, using case number 4224 as an example: 4224_t0.pdf includes the MRI data with segmentation of case number 4224 at baseline. 4224_t1_CAD_G_VC_S.pdf includes the MRI data with segmentation of case number 4224 at time 1, i.e., the time point of growth detection by the change-of-point method when the time point was different from the most recent MRI. 4224_tend_CAD_G_VC_G.pdf includes the MRI data with segmentation of case number 4224 at the most recent MRI. CAD_G and CAD_S mean that the change-of-point method predicted growth (G) or stability (S), respectively. VC_G and VC_S mean that the visual comparison detected growth (G) or stability (S), respectively. (ZIP) [file pmed.1002810.s001.zip › 4386_tend_CAD_G_VC_G.pdf]

Case Number:6934; Time 0 (Baseline).

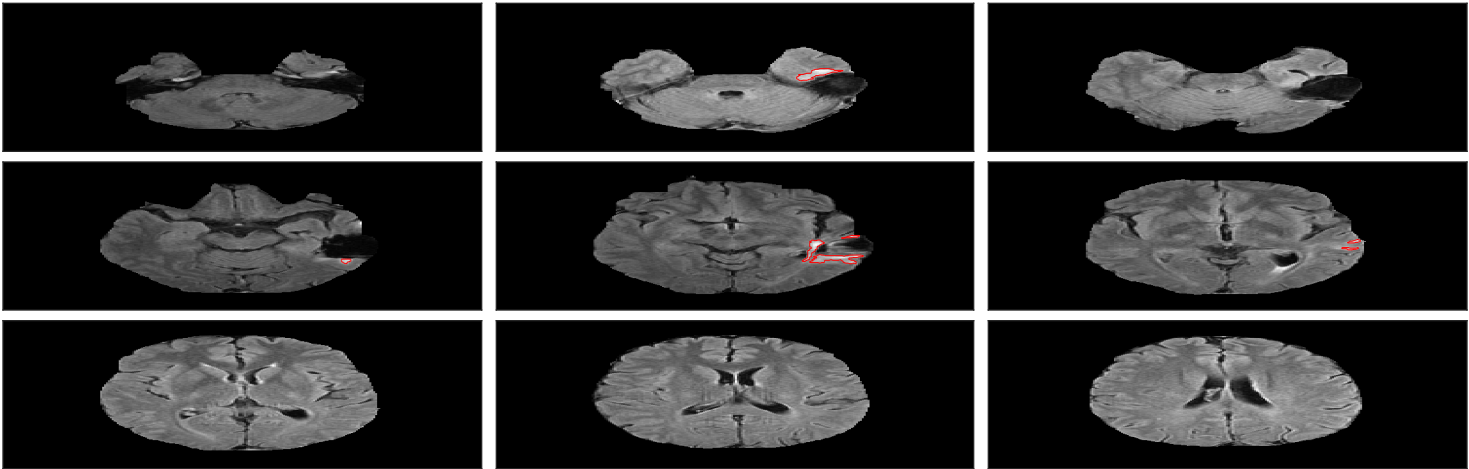

Supplement: S1 Data — The .zip file includes .pdf files labeled as follows, using case number 4224 as an example: 4224_t0.pdf includes the MRI data with segmentation of case number 4224 at baseline. 4224_t1_CAD_G_VC_S.pdf includes the MRI data with segmentation of case number 4224 at time 1, i.e., the time point of growth detection by the change-of-point method when the time point was different from the most recent MRI. 4224_tend_CAD_G_VC_G.pdf includes the MRI data with segmentation of case number 4224 at the most recent MRI. CAD_G and CAD_S mean that the change-of-point method predicted growth (G) or stability (S), respectively. VC_G and VC_S mean that the visual comparison detected growth (G) or stability (S), respectively. (ZIP) [file pmed.1002810.s001.zip › 6934_t0.pdf]

Case Number:7735; LAST MRI; CAD --> STABLE; VC --> STABLE; Interval From Baseline =19 months.

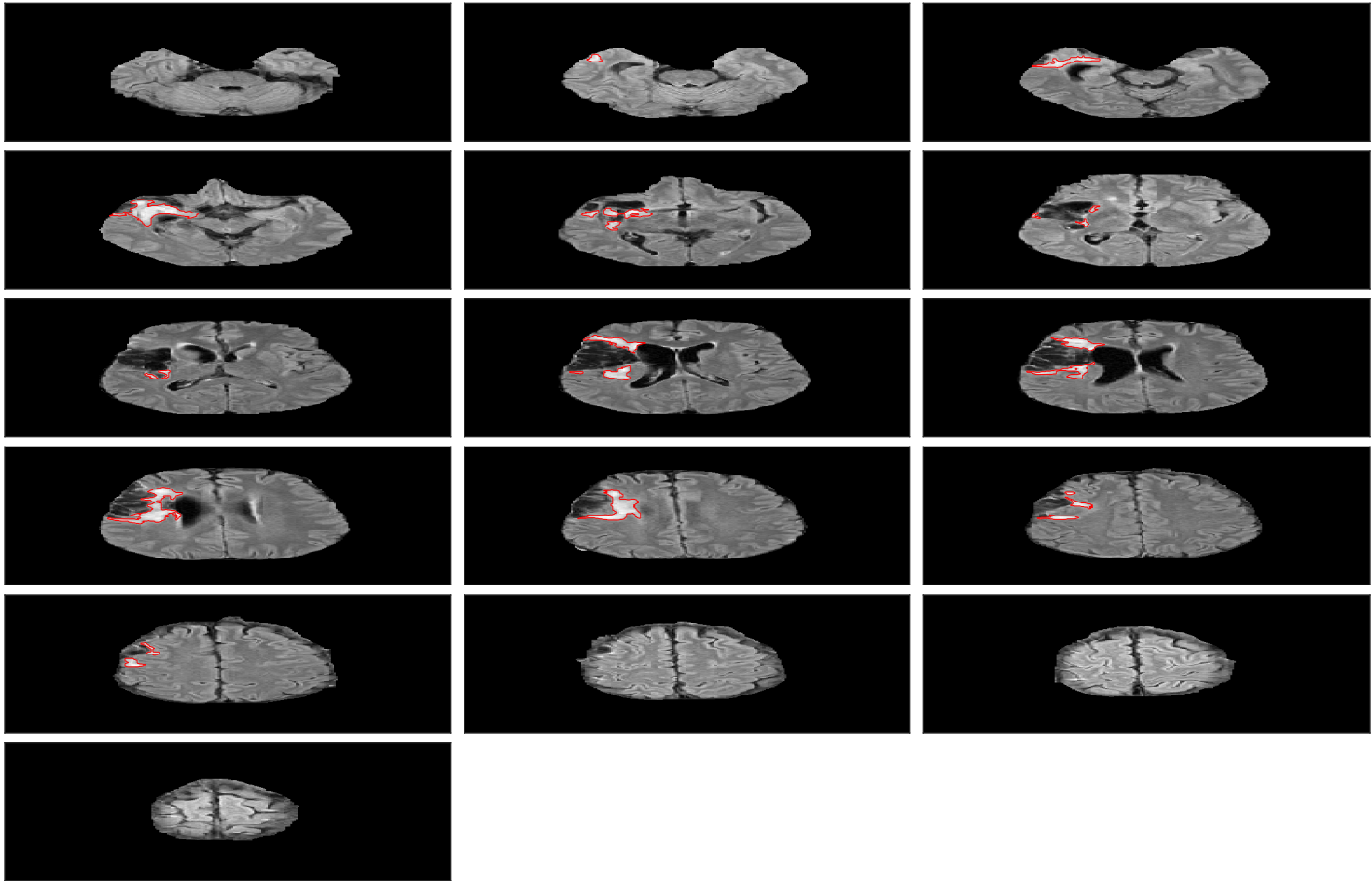

Supplement: S1 Data — The .zip file includes .pdf files labeled as follows, using case number 4224 as an example: 4224_t0.pdf includes the MRI data with segmentation of case number 4224 at baseline. 4224_t1_CAD_G_VC_S.pdf includes the MRI data with segmentation of case number 4224 at time 1, i.e., the time point of growth detection by the change-of-point method when the time point was different from the most recent MRI. 4224_tend_CAD_G_VC_G.pdf includes the MRI data with segmentation of case number 4224 at the most recent MRI. CAD_G and CAD_S mean that the change-of-point method predicted growth (G) or stability (S), respectively. VC_G and VC_S mean that the visual comparison detected growth (G) or stability (S), respectively. (ZIP) [file pmed.1002810.s001.zip › 7735_tend_CAD_S_VC_S.pdf]

Case Number:4389; TIME 1; CAD --> GROWTH; VC --> STABLE; Interval From Baseline =14 months.

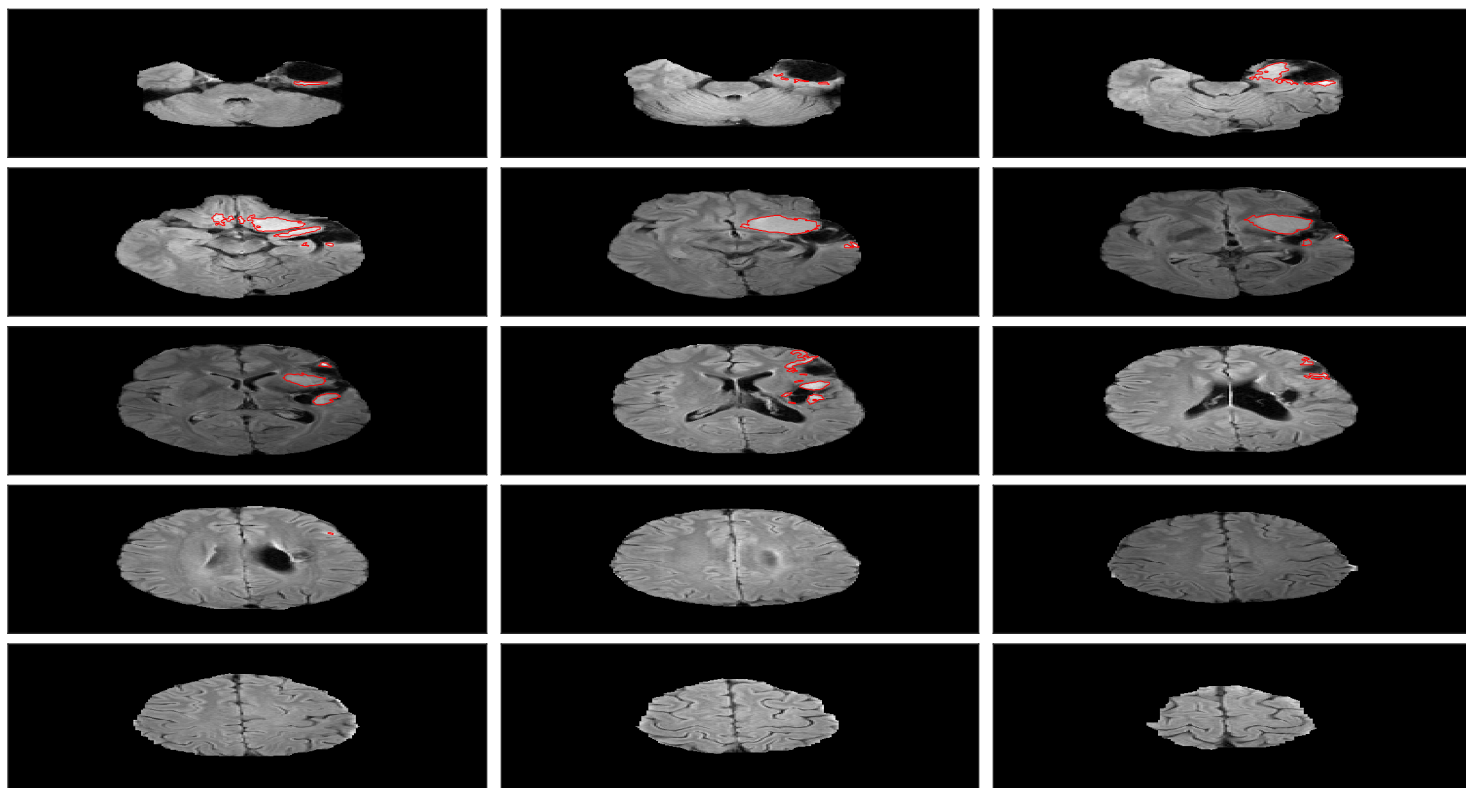

Supplement: S1 Data — The .zip file includes .pdf files labeled as follows, using case number 4224 as an example: 4224_t0.pdf includes the MRI data with segmentation of case number 4224 at baseline. 4224_t1_CAD_G_VC_S.pdf includes the MRI data with segmentation of case number 4224 at time 1, i.e., the time point of growth detection by the change-of-point method when the time point was different from the most recent MRI. 4224_tend_CAD_G_VC_G.pdf includes the MRI data with segmentation of case number 4224 at the most recent MRI. CAD_G and CAD_S mean that the change-of-point method predicted growth (G) or stability (S), respectively. VC_G and VC_S mean that the visual comparison detected growth (G) or stability (S), respectively. (ZIP) [file pmed.1002810.s001.zip › 4389_t1_CAD_G_VC_S.pdf]

Case Number:7472; LAST MRI; CAD --> GROWTH; VC --> STABLE; Interval From Baseline =22 months.

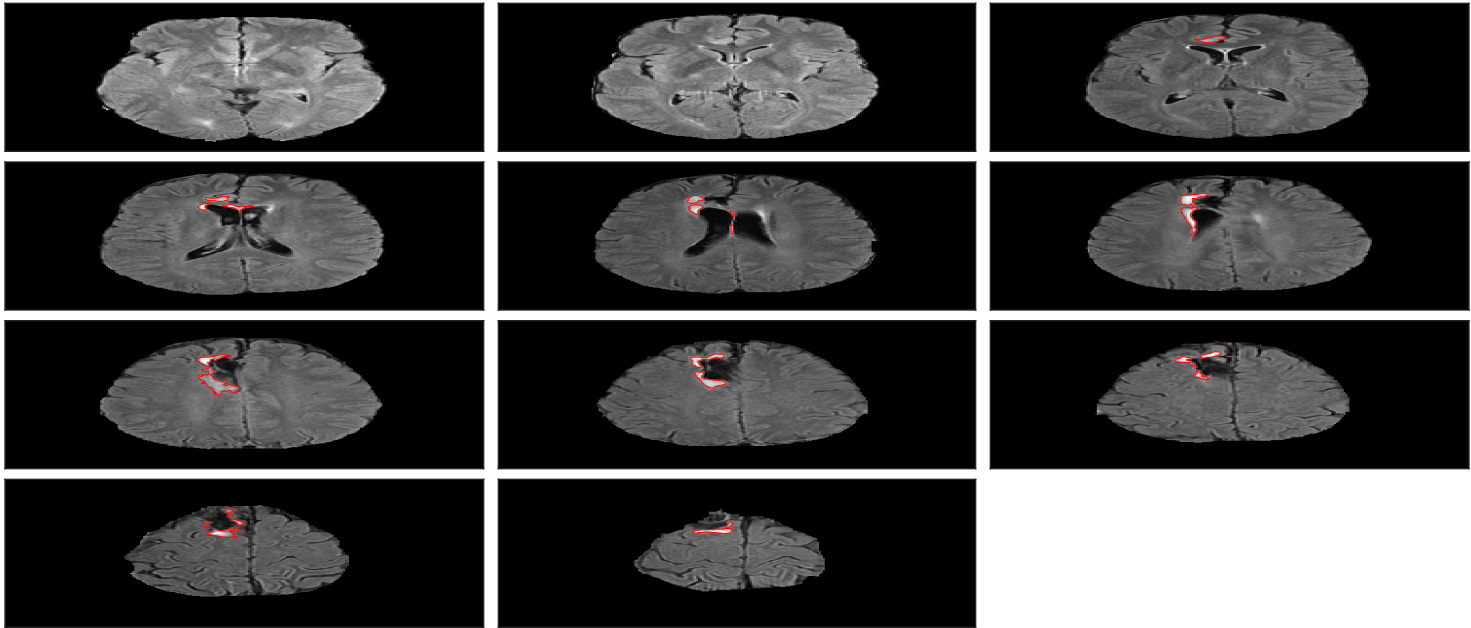

Supplement: S1 Data — The .zip file includes .pdf files labeled as follows, using case number 4224 as an example: 4224_t0.pdf includes the MRI data with segmentation of case number 4224 at baseline. 4224_t1_CAD_G_VC_S.pdf includes the MRI data with segmentation of case number 4224 at time 1, i.e., the time point of growth detection by the change-of-point method when the time point was different from the most recent MRI. 4224_tend_CAD_G_VC_G.pdf includes the MRI data with segmentation of case number 4224 at the most recent MRI. CAD_G and CAD_S mean that the change-of-point method predicted growth (G) or stability (S), respectively. VC_G and VC_S mean that the visual comparison detected growth (G) or stability (S), respectively. (ZIP) [file pmed.1002810.s001.zip › 7472_tend_CAD_G_VC_S.pdf]

Case Number:7469; LAST MRI; CAD --> GROWTH; VC --> GROWTH; Interval From Baseline =40 months.

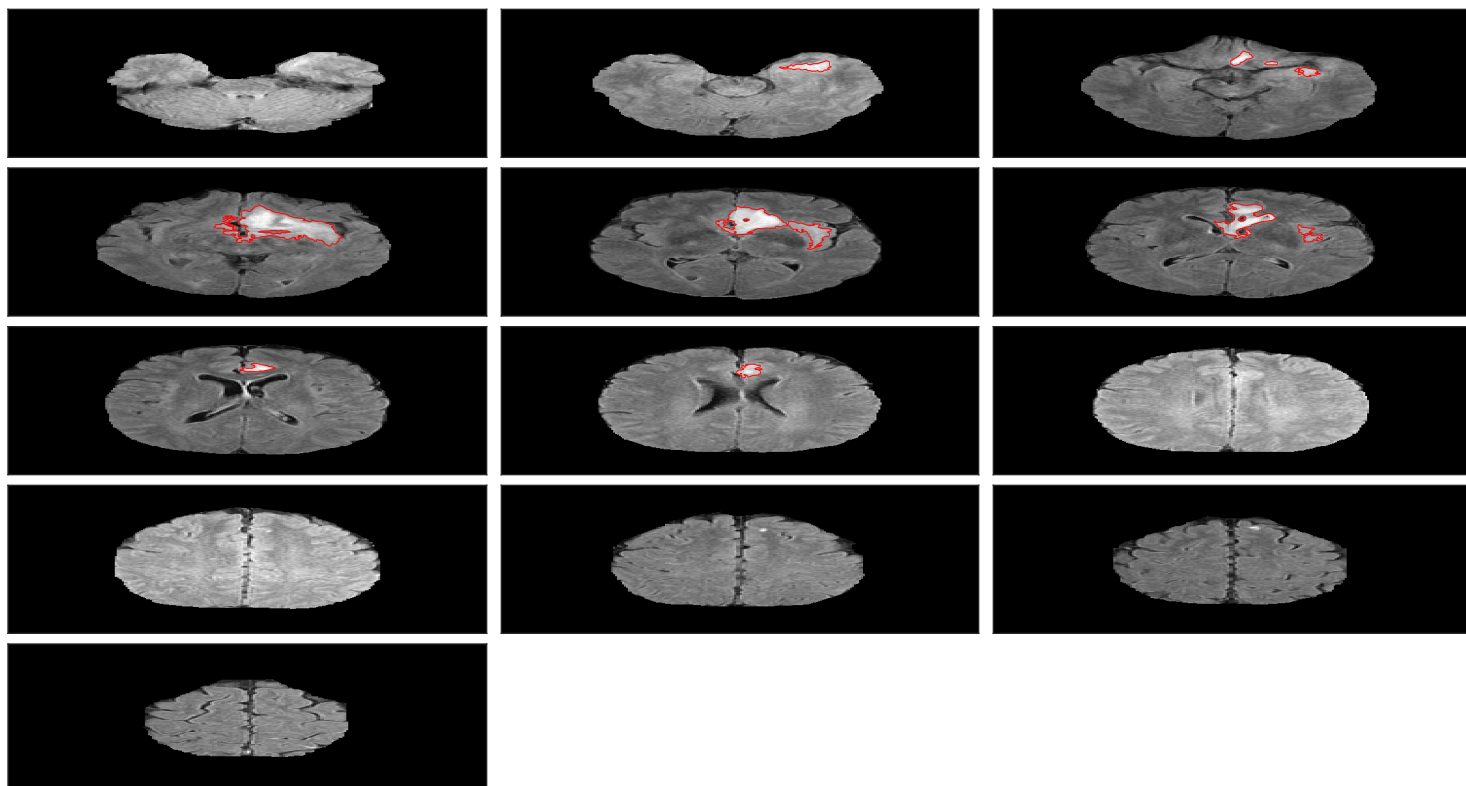

Supplement: S1 Data — The .zip file includes .pdf files labeled as follows, using case number 4224 as an example: 4224_t0.pdf includes the MRI data with segmentation of case number 4224 at baseline. 4224_t1_CAD_G_VC_S.pdf includes the MRI data with segmentation of case number 4224 at time 1, i.e., the time point of growth detection by the change-of-point method when the time point was different from the most recent MRI. 4224_tend_CAD_G_VC_G.pdf includes the MRI data with segmentation of case number 4224 at the most recent MRI. CAD_G and CAD_S mean that the change-of-point method predicted growth (G) or stability (S), respectively. VC_G and VC_S mean that the visual comparison detected growth (G) or stability (S), respectively. (ZIP) [file pmed.1002810.s001.zip › 7469_tend_CAD_G_VC_G.pdf]

Case Number:7474; Time 0 (Baseline).

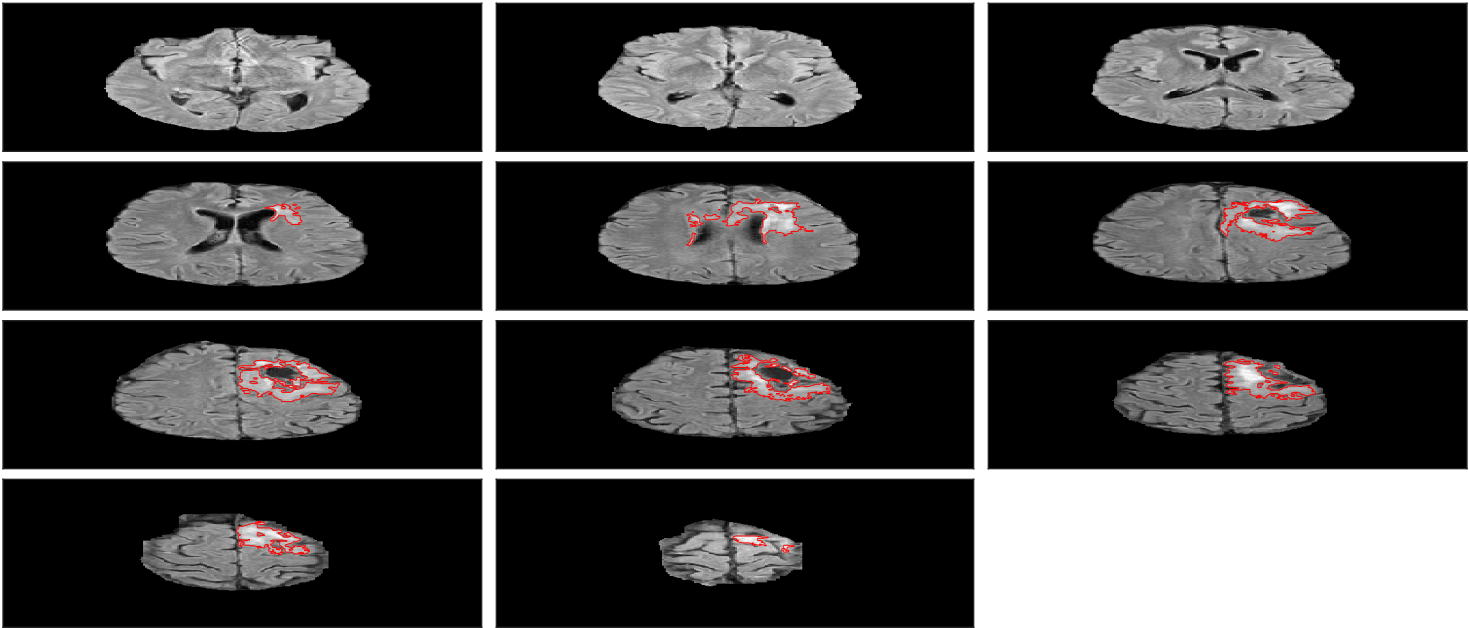

Supplement: S1 Data — The .zip file includes .pdf files labeled as follows, using case number 4224 as an example: 4224_t0.pdf includes the MRI data with segmentation of case number 4224 at baseline. 4224_t1_CAD_G_VC_S.pdf includes the MRI data with segmentation of case number 4224 at time 1, i.e., the time point of growth detection by the change-of-point method when the time point was different from the most recent MRI. 4224_tend_CAD_G_VC_G.pdf includes the MRI data with segmentation of case number 4224 at the most recent MRI. CAD_G and CAD_S mean that the change-of-point method predicted growth (G) or stability (S), respectively. VC_G and VC_S mean that the visual comparison detected growth (G) or stability (S), respectively. (ZIP) [file pmed.1002810.s001.zip › 7474_t0.pdf]

**Case Number:7718; LAST MRI; CAD --> GROWTH; VC --> STABLE; Interval From Baseline =73 months.**

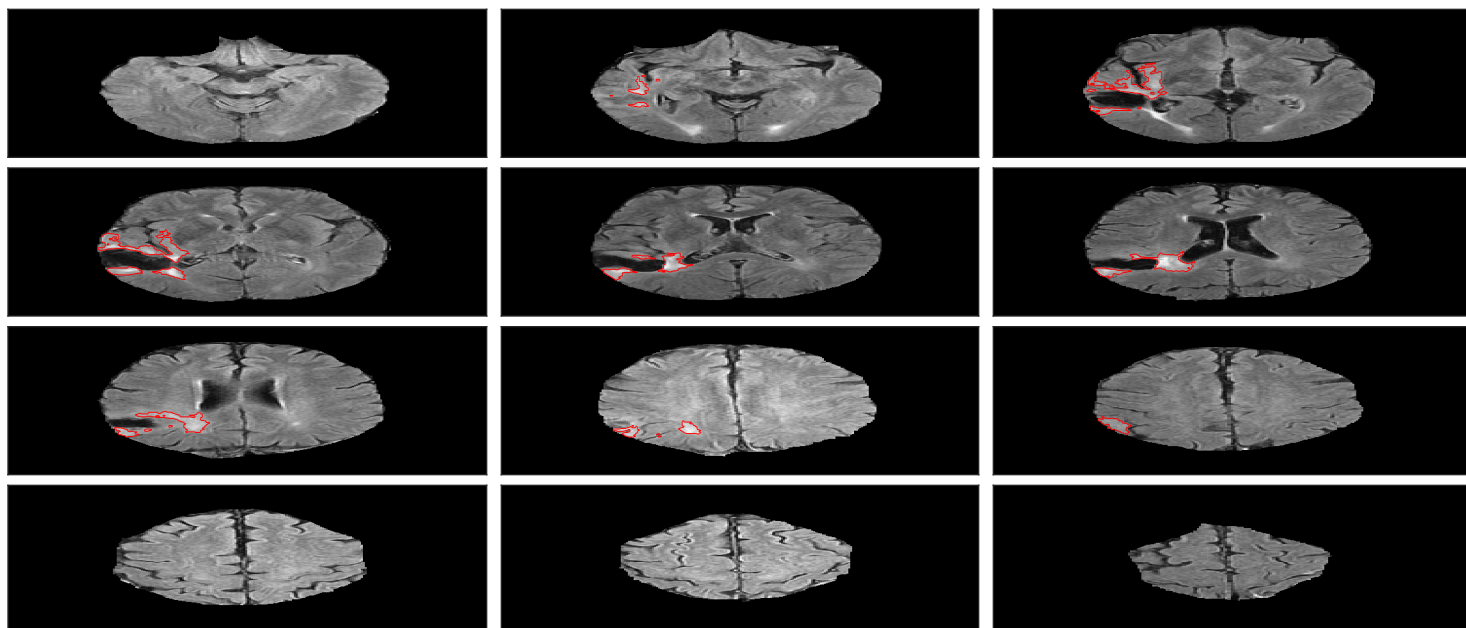

Supplement: S1 Data — The .zip file includes .pdf files labeled as follows, using case number 4224 as an example: 4224_t0.pdf includes the MRI data with segmentation of case number 4224 at baseline. 4224_t1_CAD_G_VC_S.pdf includes the MRI data with segmentation of case number 4224 at time 1, i.e., the time point of growth detection by the change-of-point method when the time point was different from the most recent MRI. 4224_tend_CAD_G_VC_G.pdf includes the MRI data with segmentation of case number 4224 at the most recent MRI. CAD_G and CAD_S mean that the change-of-point method predicted growth (G) or stability (S), respectively. VC_G and VC_S mean that the visual comparison detected growth (G) or stability (S), respectively. (ZIP) [file pmed.1002810.s001.zip › 7718_tend_CAD_G_VC_S.pdf]

Case Number:7505; Time 0 (Baseline).

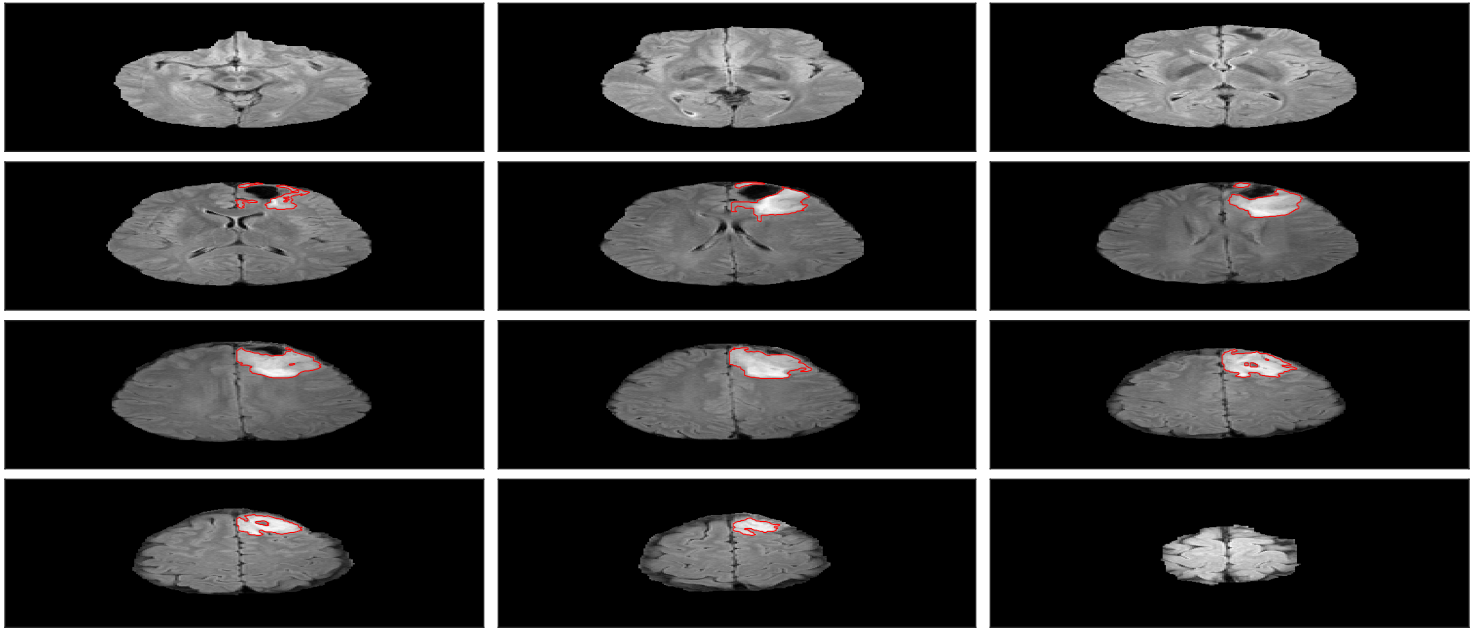

Supplement: S1 Data — The .zip file includes .pdf files labeled as follows, using case number 4224 as an example: 4224_t0.pdf includes the MRI data with segmentation of case number 4224 at baseline. 4224_t1_CAD_G_VC_S.pdf includes the MRI data with segmentation of case number 4224 at time 1, i.e., the time point of growth detection by the change-of-point method when the time point was different from the most recent MRI. 4224_tend_CAD_G_VC_G.pdf includes the MRI data with segmentation of case number 4224 at the most recent MRI. CAD_G and CAD_S mean that the change-of-point method predicted growth (G) or stability (S), respectively. VC_G and VC_S mean that the visual comparison detected growth (G) or stability (S), respectively. (ZIP) [file pmed.1002810.s001.zip › 7505_t0.pdf]

Case Number:7470; LAST MRI; CAD --> GROWTH; VC --> GROWTH; Interval From Baseline =21 months.

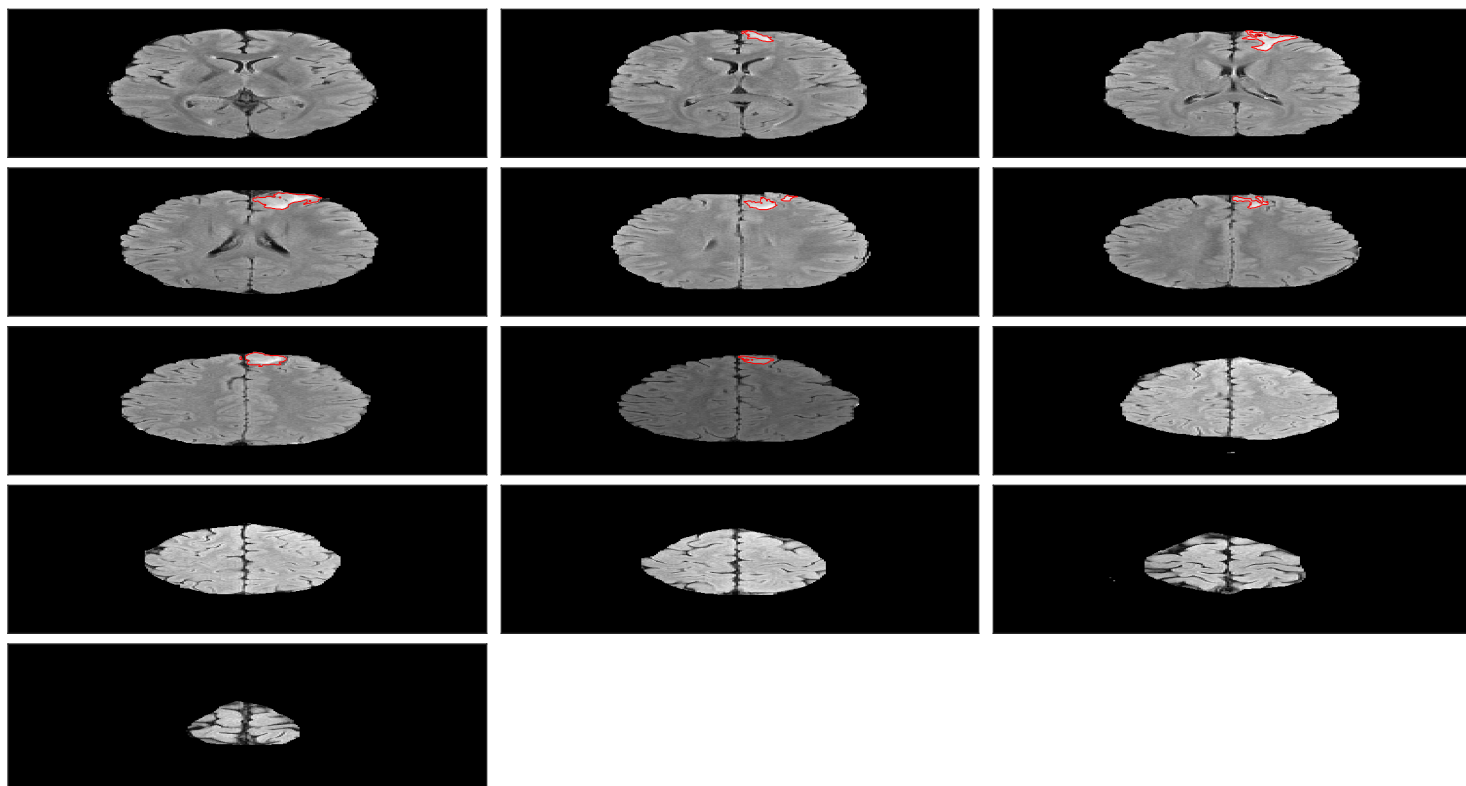

Supplement: S1 Data — The .zip file includes .pdf files labeled as follows, using case number 4224 as an example: 4224_t0.pdf includes the MRI data with segmentation of case number 4224 at baseline. 4224_t1_CAD_G_VC_S.pdf includes the MRI data with segmentation of case number 4224 at time 1, i.e., the time point of growth detection by the change-of-point method when the time point was different from the most recent MRI. 4224_tend_CAD_G_VC_G.pdf includes the MRI data with segmentation of case number 4224 at the most recent MRI. CAD_G and CAD_S mean that the change-of-point method predicted growth (G) or stability (S), respectively. VC_G and VC_S mean that the visual comparison detected growth (G) or stability (S), respectively. (ZIP) [file pmed.1002810.s001.zip › 7470_tend_CAD_G_VC_G.pdf]

Case Number:7754; LAST MRI; CAD --> STABLE; VC --> STABLE; Interval From Baseline =79 months.

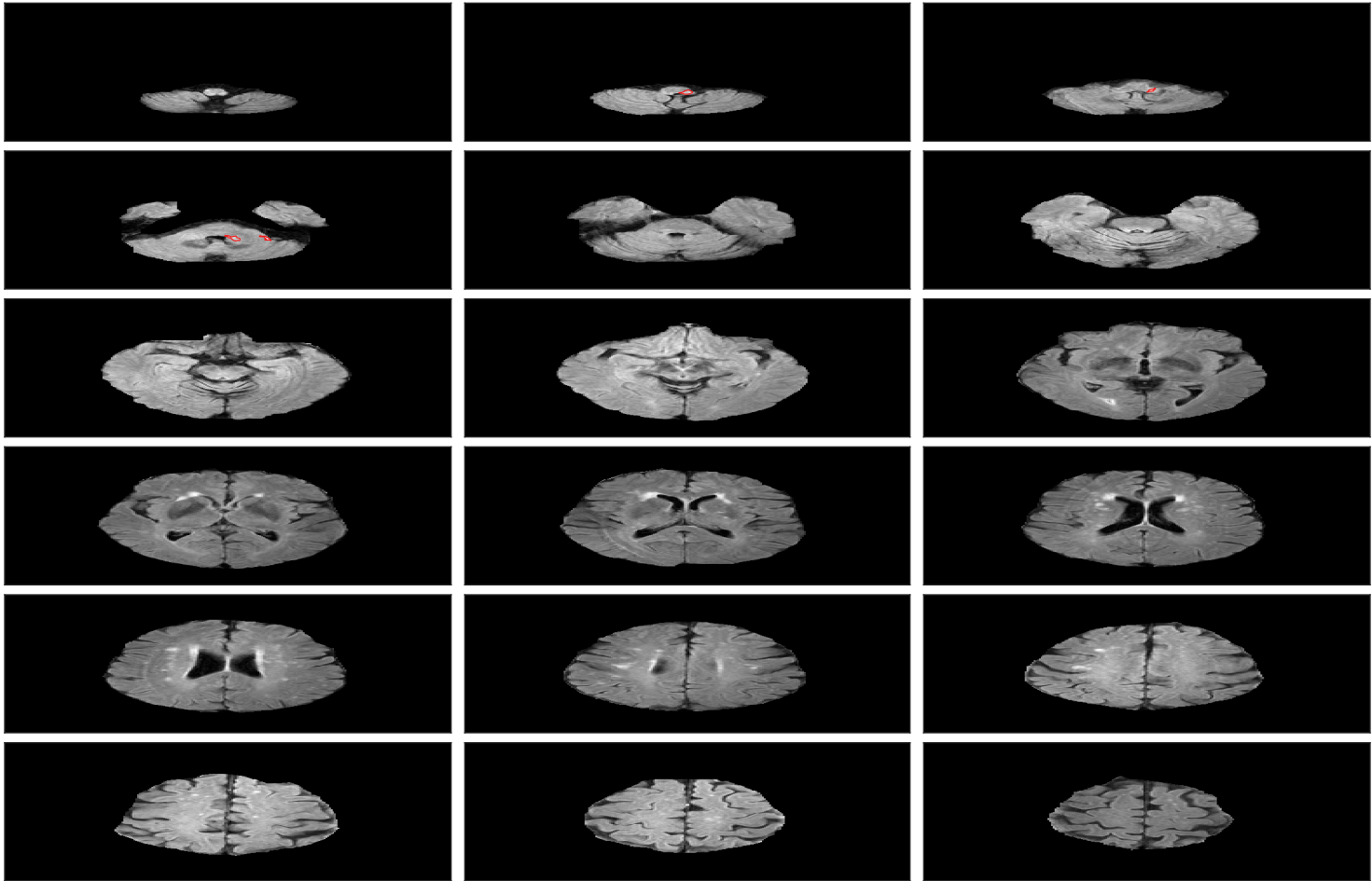

Supplement: S1 Data — The .zip file includes .pdf files labeled as follows, using case number 4224 as an example: 4224_t0.pdf includes the MRI data with segmentation of case number 4224 at baseline. 4224_t1_CAD_G_VC_S.pdf includes the MRI data with segmentation of case number 4224 at time 1, i.e., the time point of growth detection by the change-of-point method when the time point was different from the most recent MRI. 4224_tend_CAD_G_VC_G.pdf includes the MRI data with segmentation of case number 4224 at the most recent MRI. CAD_G and CAD_S mean that the change-of-point method predicted growth (G) or stability (S), respectively. VC_G and VC_S mean that the visual comparison detected growth (G) or stability (S), respectively. (ZIP) [file pmed.1002810.s001.zip › 7754_tend_CAD_S_VC_S.pdf]

Case Number:7478; Time 0 (Baseline).

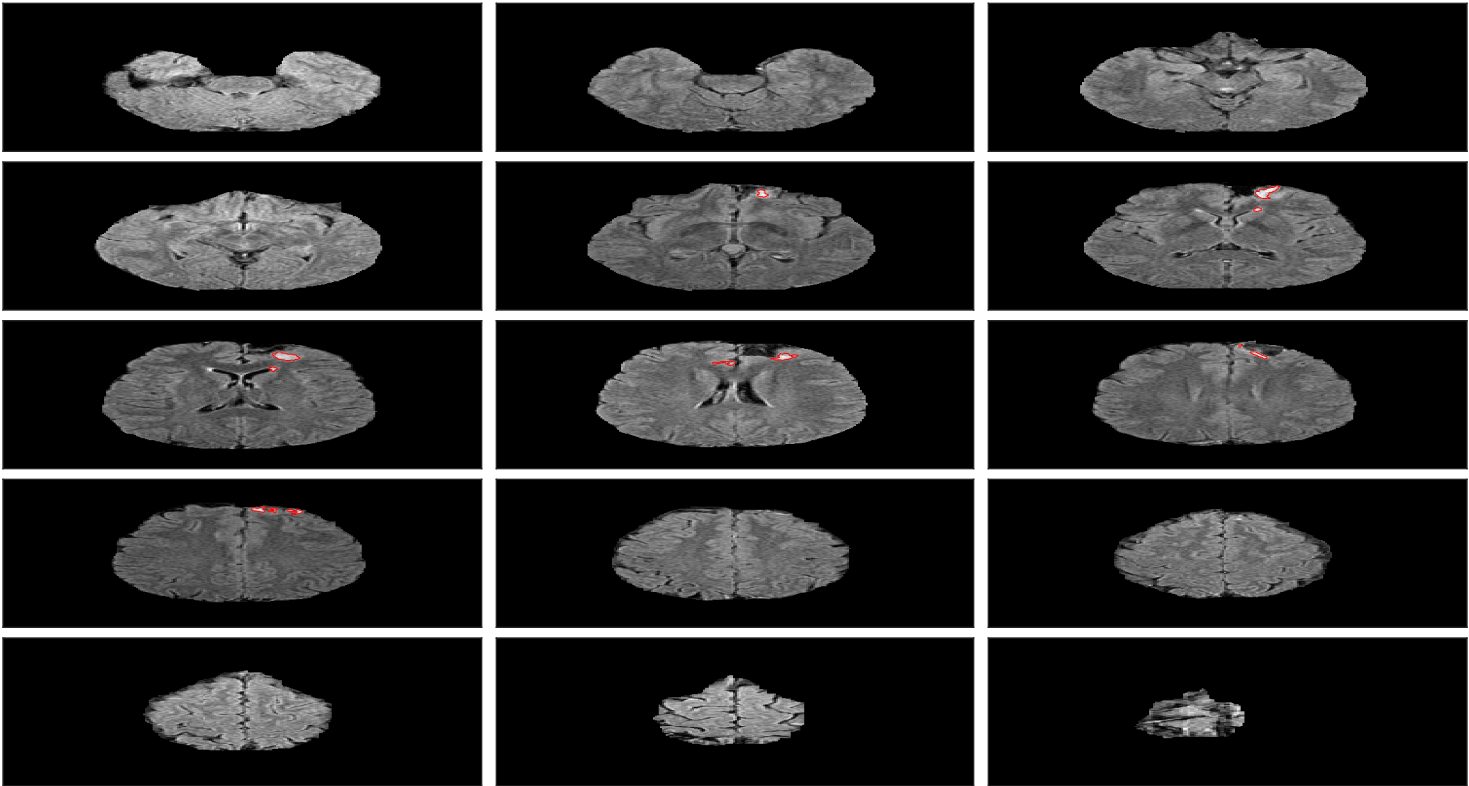

Supplement: S1 Data — The .zip file includes .pdf files labeled as follows, using case number 4224 as an example: 4224_t0.pdf includes the MRI data with segmentation of case number 4224 at baseline. 4224_t1_CAD_G_VC_S.pdf includes the MRI data with segmentation of case number 4224 at time 1, i.e., the time point of growth detection by the change-of-point method when the time point was different from the most recent MRI. 4224_tend_CAD_G_VC_G.pdf includes the MRI data with segmentation of case number 4224 at the most recent MRI. CAD_G and CAD_S mean that the change-of-point method predicted growth (G) or stability (S), respectively. VC_G and VC_S mean that the visual comparison detected growth (G) or stability (S), respectively. (ZIP) [file pmed.1002810.s001.zip › 7478_t0.pdf]

Case Number:7336; TIME 1; CAD --> GROWTH; VC --> STABLE; Interval From Baseline =19 months.

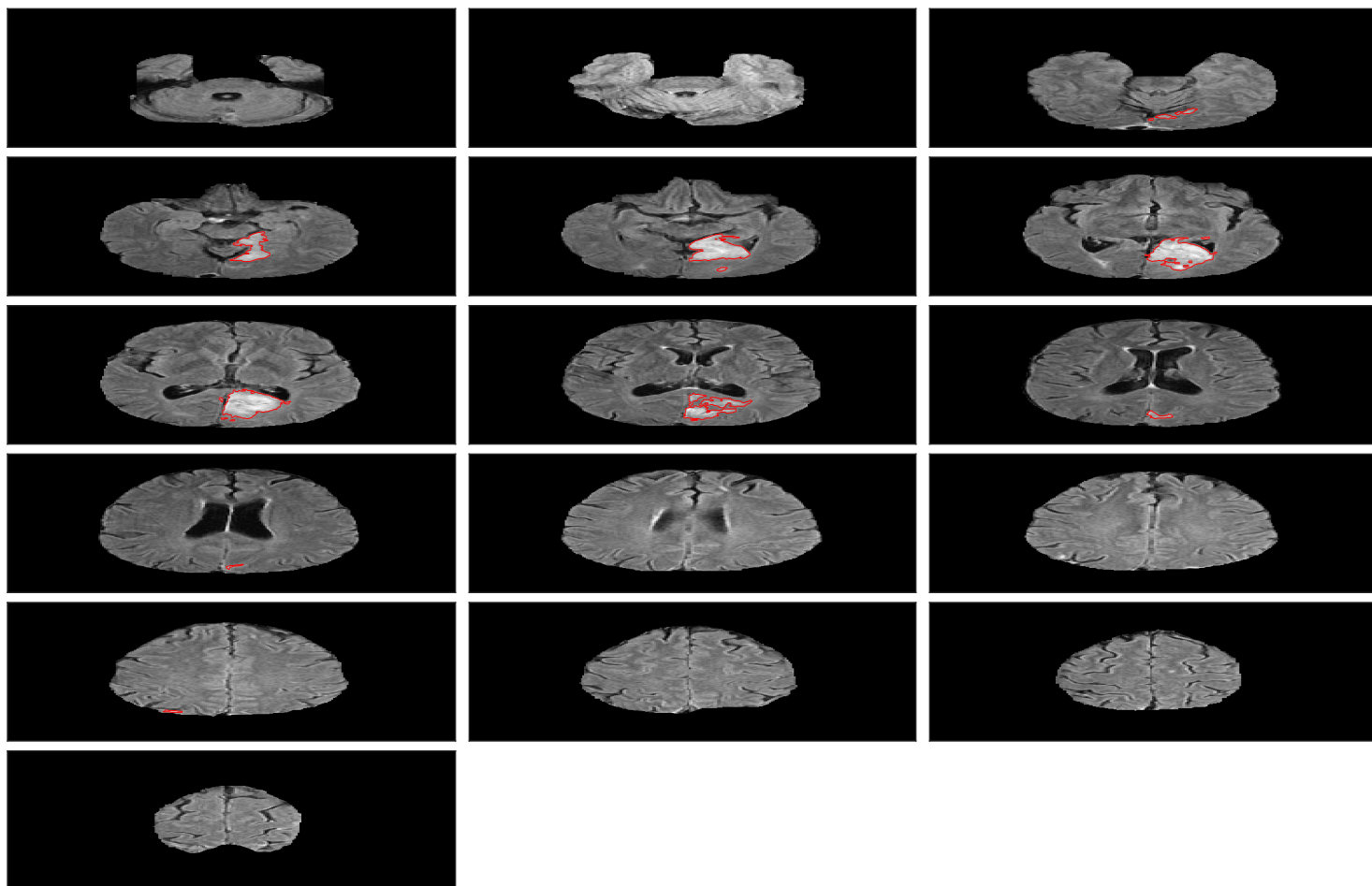

Supplement: S1 Data — The .zip file includes .pdf files labeled as follows, using case number 4224 as an example: 4224_t0.pdf includes the MRI data with segmentation of case number 4224 at baseline. 4224_t1_CAD_G_VC_S.pdf includes the MRI data with segmentation of case number 4224 at time 1, i.e., the time point of growth detection by the change-of-point method when the time point was different from the most recent MRI. 4224_tend_CAD_G_VC_G.pdf includes the MRI data with segmentation of case number 4224 at the most recent MRI. CAD_G and CAD_S mean that the change-of-point method predicted growth (G) or stability (S), respectively. VC_G and VC_S mean that the visual comparison detected growth (G) or stability (S), respectively. (ZIP) [file pmed.1002810.s001.zip › 7336_t1_CAD_G_VC_S.pdf]

Case Number:7504; LAST MRI; CAD --> GROWTH; VC --> STABLE; Interval From Baseline =90 months.

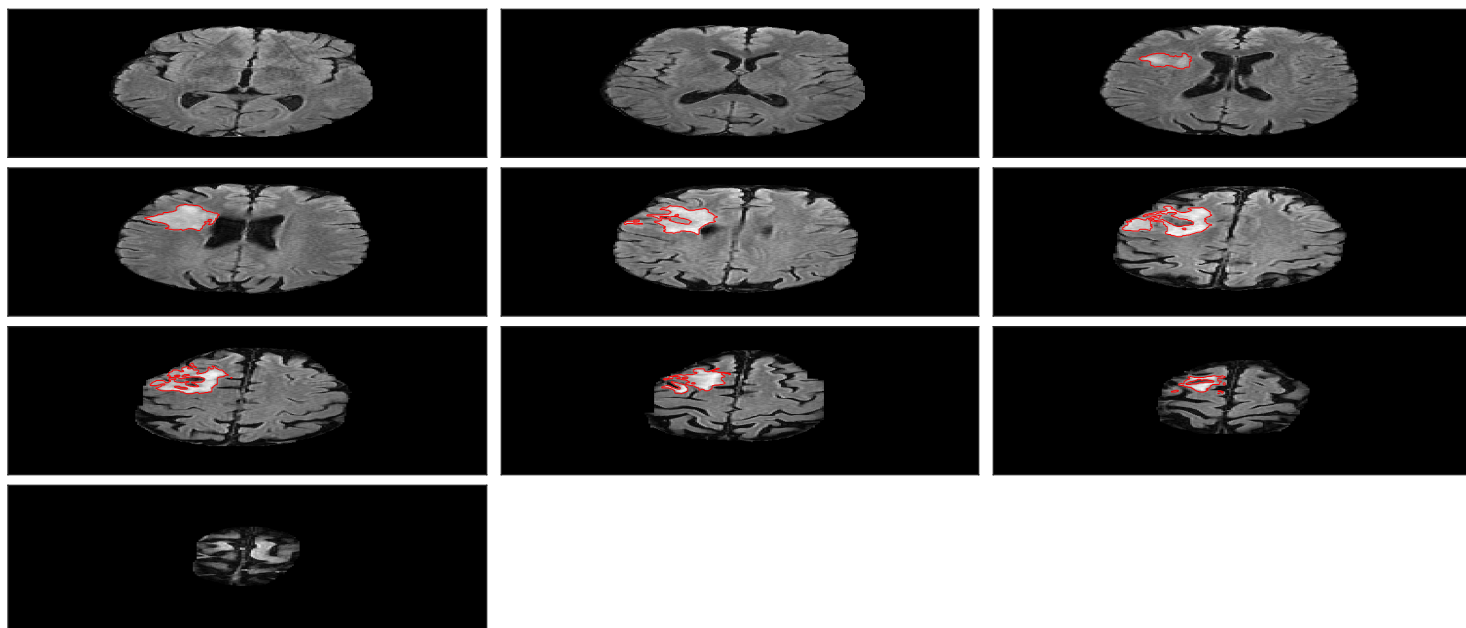

Supplement: S1 Data — The .zip file includes .pdf files labeled as follows, using case number 4224 as an example: 4224_t0.pdf includes the MRI data with segmentation of case number 4224 at baseline. 4224_t1_CAD_G_VC_S.pdf includes the MRI data with segmentation of case number 4224 at time 1, i.e., the time point of growth detection by the change-of-point method when the time point was different from the most recent MRI. 4224_tend_CAD_G_VC_G.pdf includes the MRI data with segmentation of case number 4224 at the most recent MRI. CAD_G and CAD_S mean that the change-of-point method predicted growth (G) or stability (S), respectively. VC_G and VC_S mean that the visual comparison detected growth (G) or stability (S), respectively. (ZIP) [file pmed.1002810.s001.zip › 7504_tend_CAD_G_VC_S.pdf]

Case Number:7755; LAST MRI; CAD --> STABLE; VC --> STABLE; Interval From Baseline =86 months.

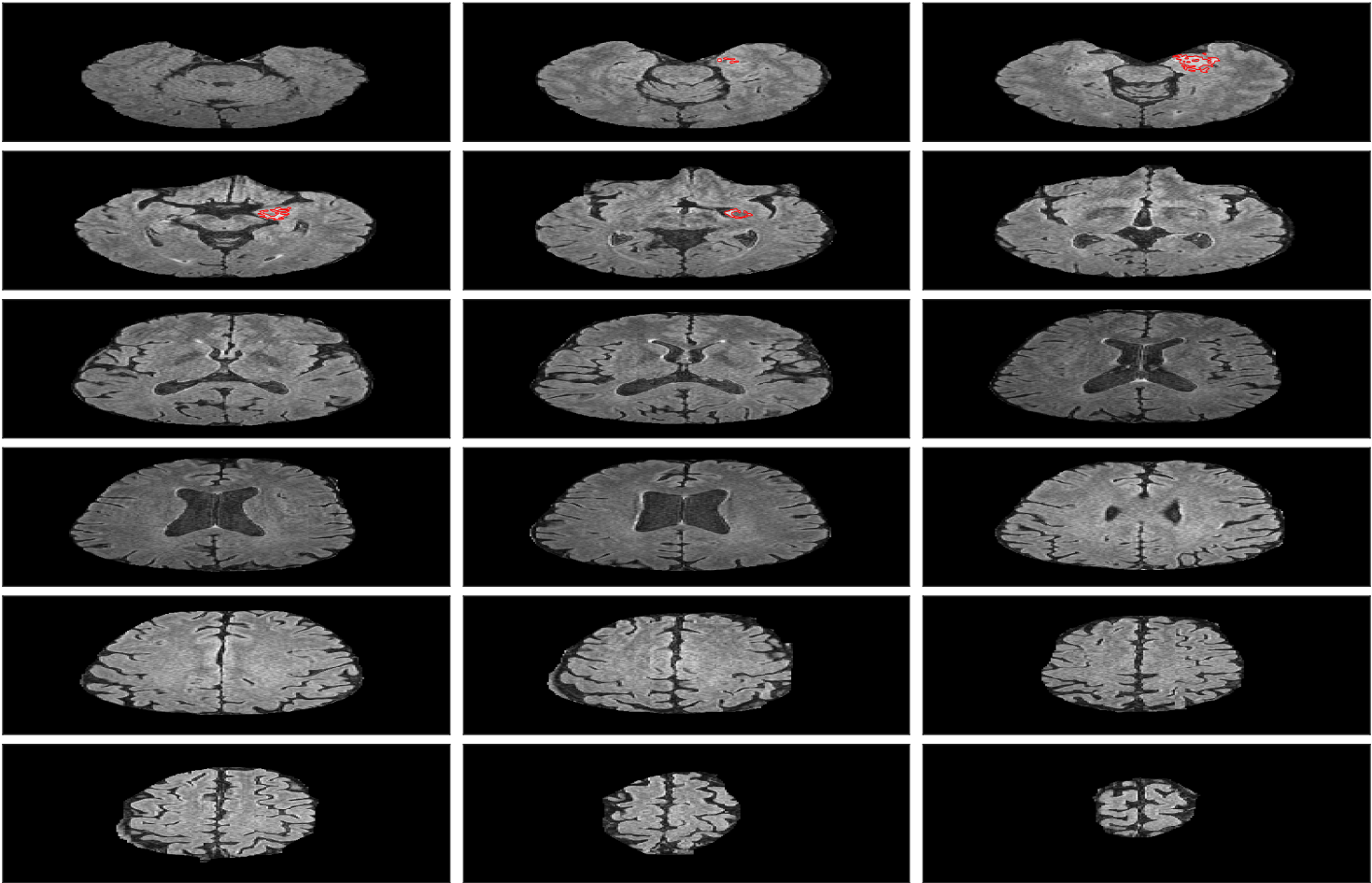

Supplement: S1 Data — The .zip file includes .pdf files labeled as follows, using case number 4224 as an example: 4224_t0.pdf includes the MRI data with segmentation of case number 4224 at baseline. 4224_t1_CAD_G_VC_S.pdf includes the MRI data with segmentation of case number 4224 at time 1, i.e., the time point of growth detection by the change-of-point method when the time point was different from the most recent MRI. 4224_tend_CAD_G_VC_G.pdf includes the MRI data with segmentation of case number 4224 at the most recent MRI. CAD_G and CAD_S mean that the change-of-point method predicted growth (G) or stability (S), respectively. VC_G and VC_S mean that the visual comparison detected growth (G) or stability (S), respectively. (ZIP) [file pmed.1002810.s001.zip › 7755_tend_CAD_S_VC_S.pdf]

Case Number:7756; LAST MRI; CAD --> STABLE; VC --> STABLE; Interval From Baseline =13 months.

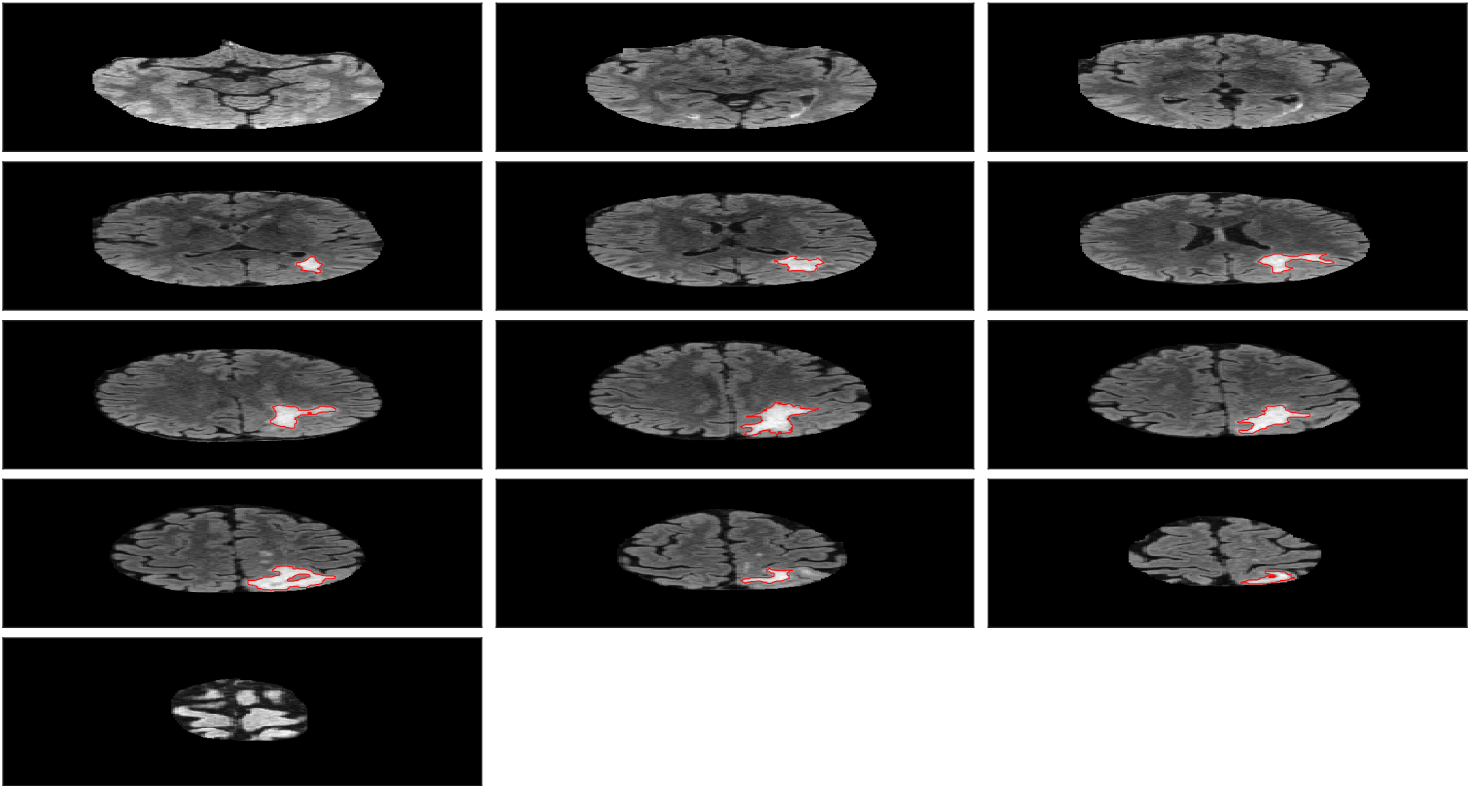

Supplement: S1 Data — The .zip file includes .pdf files labeled as follows, using case number 4224 as an example: 4224_t0.pdf includes the MRI data with segmentation of case number 4224 at baseline. 4224_t1_CAD_G_VC_S.pdf includes the MRI data with segmentation of case number 4224 at time 1, i.e., the time point of growth detection by the change-of-point method when the time point was different from the most recent MRI. 4224_tend_CAD_G_VC_G.pdf includes the MRI data with segmentation of case number 4224 at the most recent MRI. CAD_G and CAD_S mean that the change-of-point method predicted growth (G) or stability (S), respectively. VC_G and VC_S mean that the visual comparison detected growth (G) or stability (S), respectively. (ZIP) [file pmed.1002810.s001.zip › 7756_tend_CAD_S_VC_S.pdf]

Case Number:7505; LAST MRI; CAD --> GROWTH; VC --> GROWTH; Interval From Baseline =31 months.

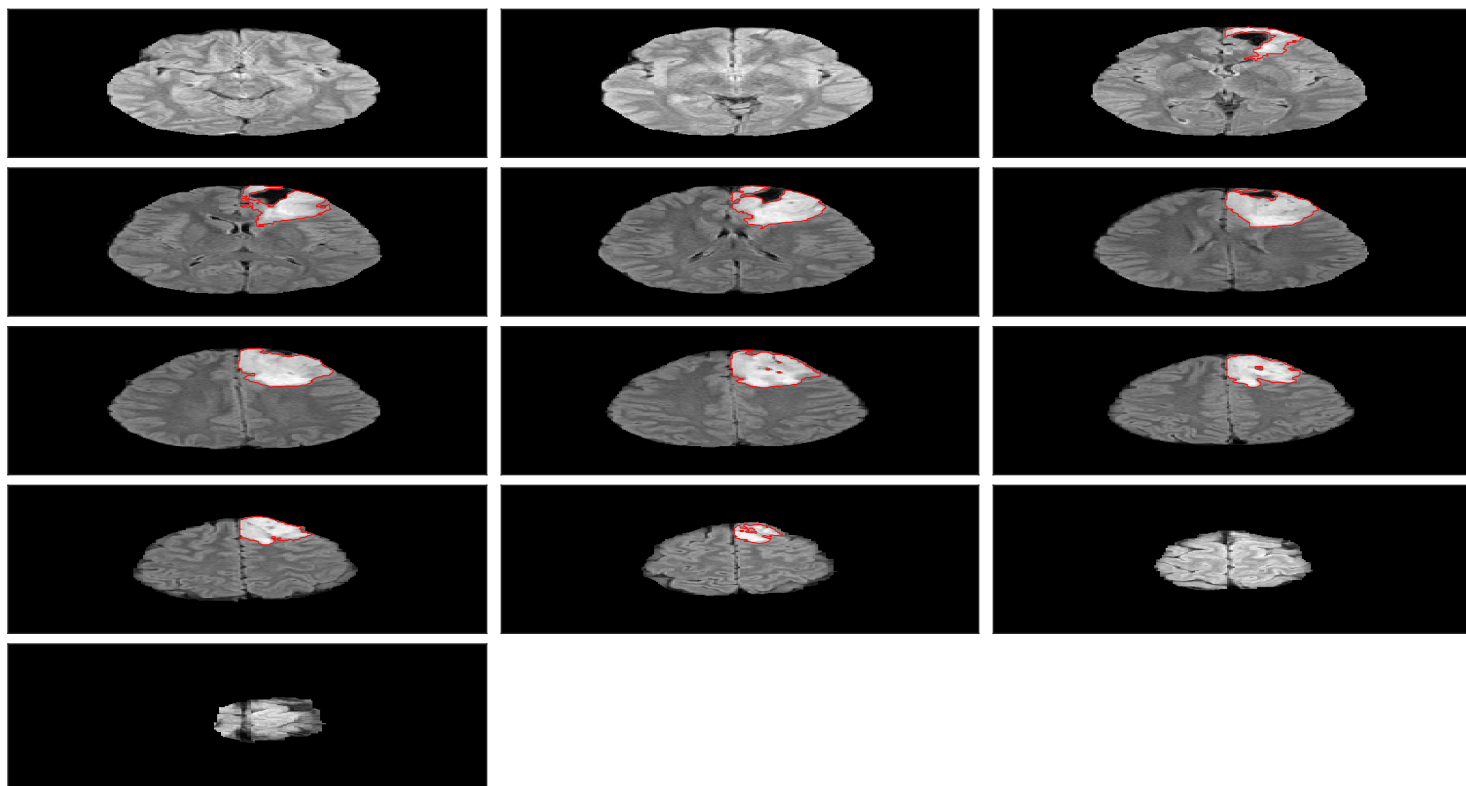

Supplement: S1 Data — The .zip file includes .pdf files labeled as follows, using case number 4224 as an example: 4224_t0.pdf includes the MRI data with segmentation of case number 4224 at baseline. 4224_t1_CAD_G_VC_S.pdf includes the MRI data with segmentation of case number 4224 at time 1, i.e., the time point of growth detection by the change-of-point method when the time point was different from the most recent MRI. 4224_tend_CAD_G_VC_G.pdf includes the MRI data with segmentation of case number 4224 at the most recent MRI. CAD_G and CAD_S mean that the change-of-point method predicted growth (G) or stability (S), respectively. VC_G and VC_S mean that the visual comparison detected growth (G) or stability (S), respectively. (ZIP) [file pmed.1002810.s001.zip › 7505_tend_CAD_G_VC_G.pdf]

Case Number:7741; Time 0 (Baseline).

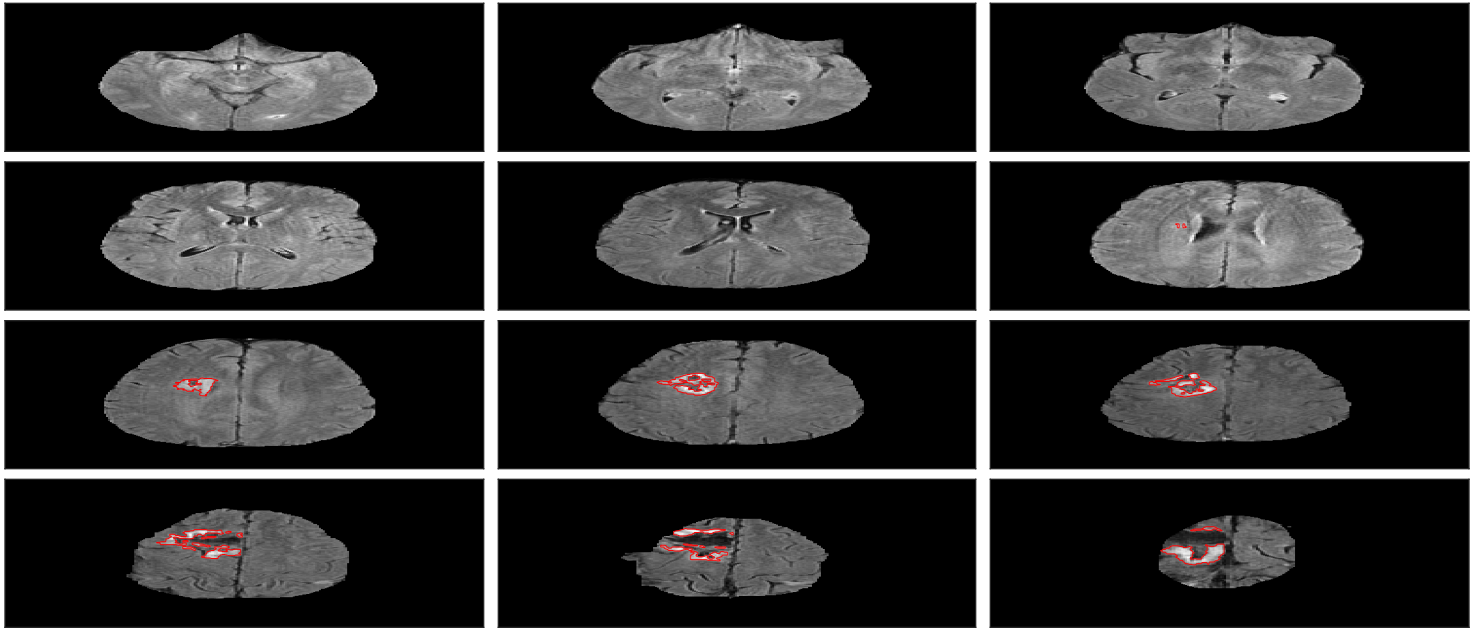

Supplement: S1 Data — The .zip file includes .pdf files labeled as follows, using case number 4224 as an example: 4224_t0.pdf includes the MRI data with segmentation of case number 4224 at baseline. 4224_t1_CAD_G_VC_S.pdf includes the MRI data with segmentation of case number 4224 at time 1, i.e., the time point of growth detection by the change-of-point method when the time point was different from the most recent MRI. 4224_tend_CAD_G_VC_G.pdf includes the MRI data with segmentation of case number 4224 at the most recent MRI. CAD_G and CAD_S mean that the change-of-point method predicted growth (G) or stability (S), respectively. VC_G and VC_S mean that the visual comparison detected growth (G) or stability (S), respectively. (ZIP) [file pmed.1002810.s001.zip › 7741_t0.pdf]

Case Number:4388; Time 0 (Baseline).

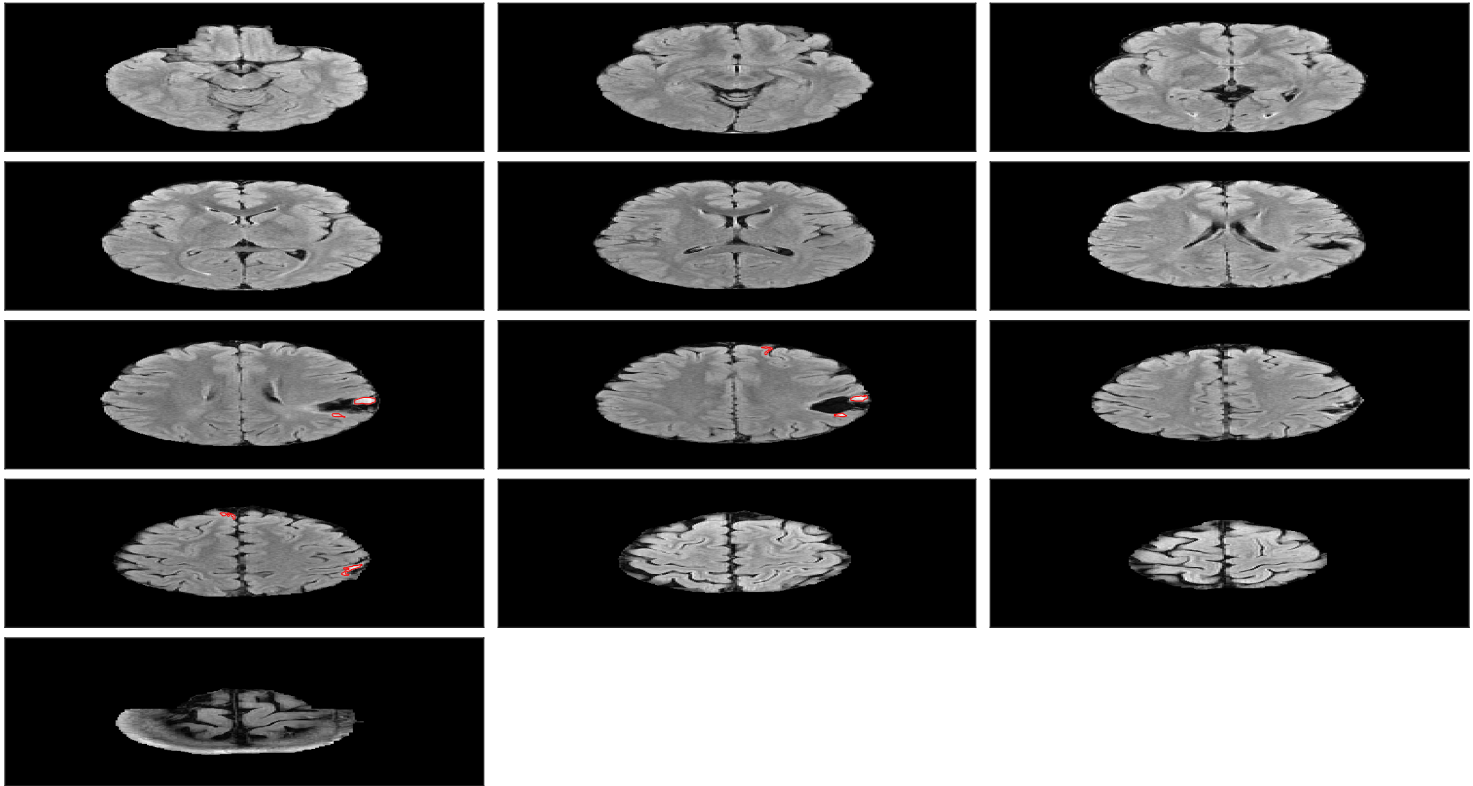

Supplement: S1 Data — The .zip file includes .pdf files labeled as follows, using case number 4224 as an example: 4224_t0.pdf includes the MRI data with segmentation of case number 4224 at baseline. 4224_t1_CAD_G_VC_S.pdf includes the MRI data with segmentation of case number 4224 at time 1, i.e., the time point of growth detection by the change-of-point method when the time point was different from the most recent MRI. 4224_tend_CAD_G_VC_G.pdf includes the MRI data with segmentation of case number 4224 at the most recent MRI. CAD_G and CAD_S mean that the change-of-point method predicted growth (G) or stability (S), respectively. VC_G and VC_S mean that the visual comparison detected growth (G) or stability (S), respectively. (ZIP) [file pmed.1002810.s001.zip › 4388_t0.pdf]

Case Number:6936; TIME 1; CAD --> GROWTH; VC --> STABLE; Interval From Baseline =10 months.

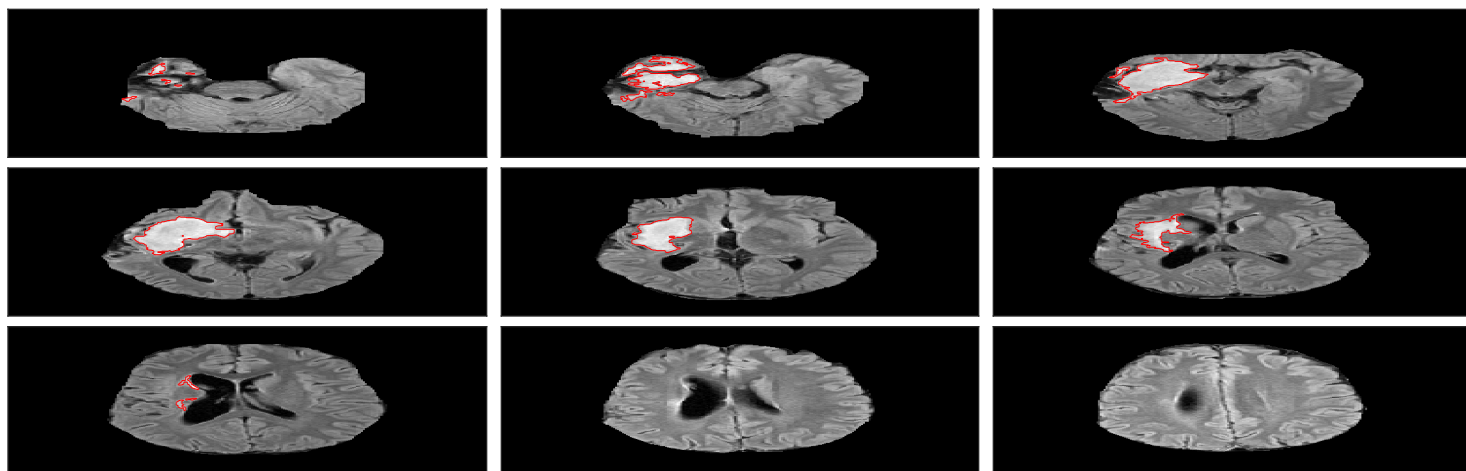

Supplement: S1 Data — The .zip file includes .pdf files labeled as follows, using case number 4224 as an example: 4224_t0.pdf includes the MRI data with segmentation of case number 4224 at baseline. 4224_t1_CAD_G_VC_S.pdf includes the MRI data with segmentation of case number 4224 at time 1, i.e., the time point of growth detection by the change-of-point method when the time point was different from the most recent MRI. 4224_tend_CAD_G_VC_G.pdf includes the MRI data with segmentation of case number 4224 at the most recent MRI. CAD_G and CAD_S mean that the change-of-point method predicted growth (G) or stability (S), respectively. VC_G and VC_S mean that the visual comparison detected growth (G) or stability (S), respectively. (ZIP) [file pmed.1002810.s001.zip › 6936_t1_CAD_G_VC_S.pdf]

Case Number:4384; Time 0 (Baseline).

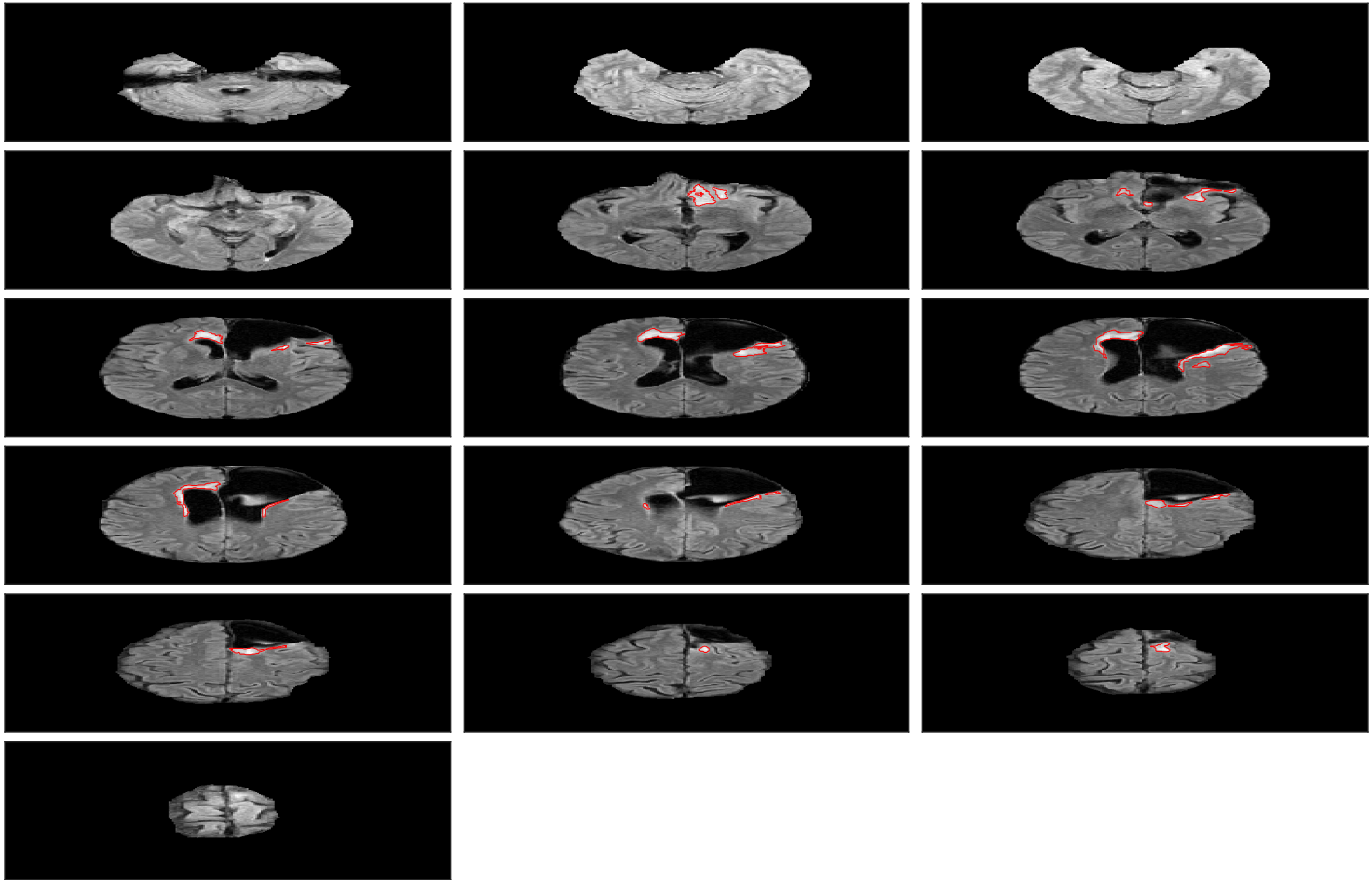

Supplement: S1 Data — The .zip file includes .pdf files labeled as follows, using case number 4224 as an example: 4224_t0.pdf includes the MRI data with segmentation of case number 4224 at baseline. 4224_t1_CAD_G_VC_S.pdf includes the MRI data with segmentation of case number 4224 at time 1, i.e., the time point of growth detection by the change-of-point method when the time point was different from the most recent MRI. 4224_tend_CAD_G_VC_G.pdf includes the MRI data with segmentation of case number 4224 at the most recent MRI. CAD_G and CAD_S mean that the change-of-point method predicted growth (G) or stability (S), respectively. VC_G and VC_S mean that the visual comparison detected growth (G) or stability (S), respectively. (ZIP) [file pmed.1002810.s001.zip › 4384_t0.pdf]

Case Number:7753; Time 0 (Baseline).

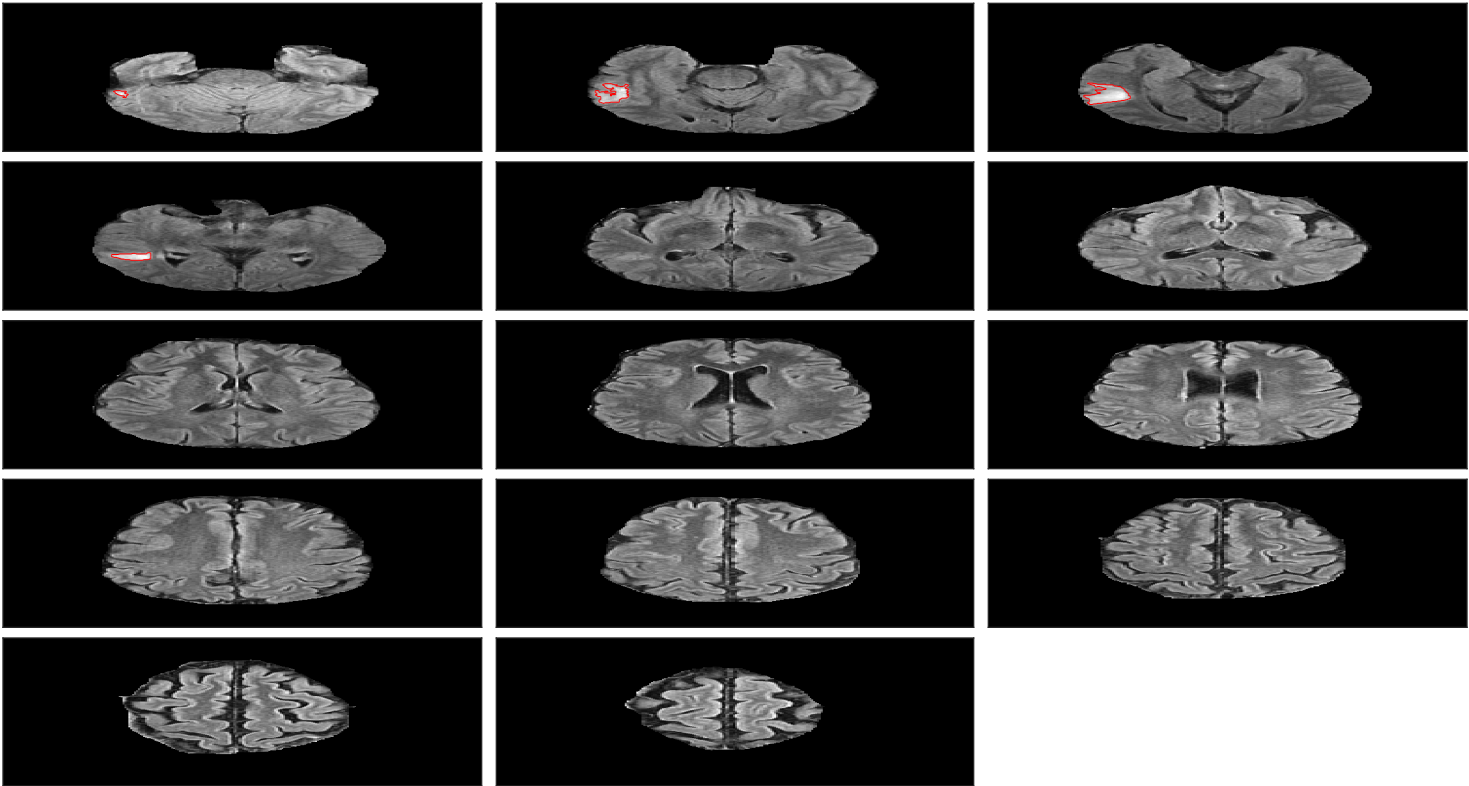

Supplement: S1 Data — The .zip file includes .pdf files labeled as follows, using case number 4224 as an example: 4224_t0.pdf includes the MRI data with segmentation of case number 4224 at baseline. 4224_t1_CAD_G_VC_S.pdf includes the MRI data with segmentation of case number 4224 at time 1, i.e., the time point of growth detection by the change-of-point method when the time point was different from the most recent MRI. 4224_tend_CAD_G_VC_G.pdf includes the MRI data with segmentation of case number 4224 at the most recent MRI. CAD_G and CAD_S mean that the change-of-point method predicted growth (G) or stability (S), respectively. VC_G and VC_S mean that the visual comparison detected growth (G) or stability (S), respectively. (ZIP) [file pmed.1002810.s001.zip › 7753_t0.pdf]

Case Number:7730; Time 0 (Baseline).

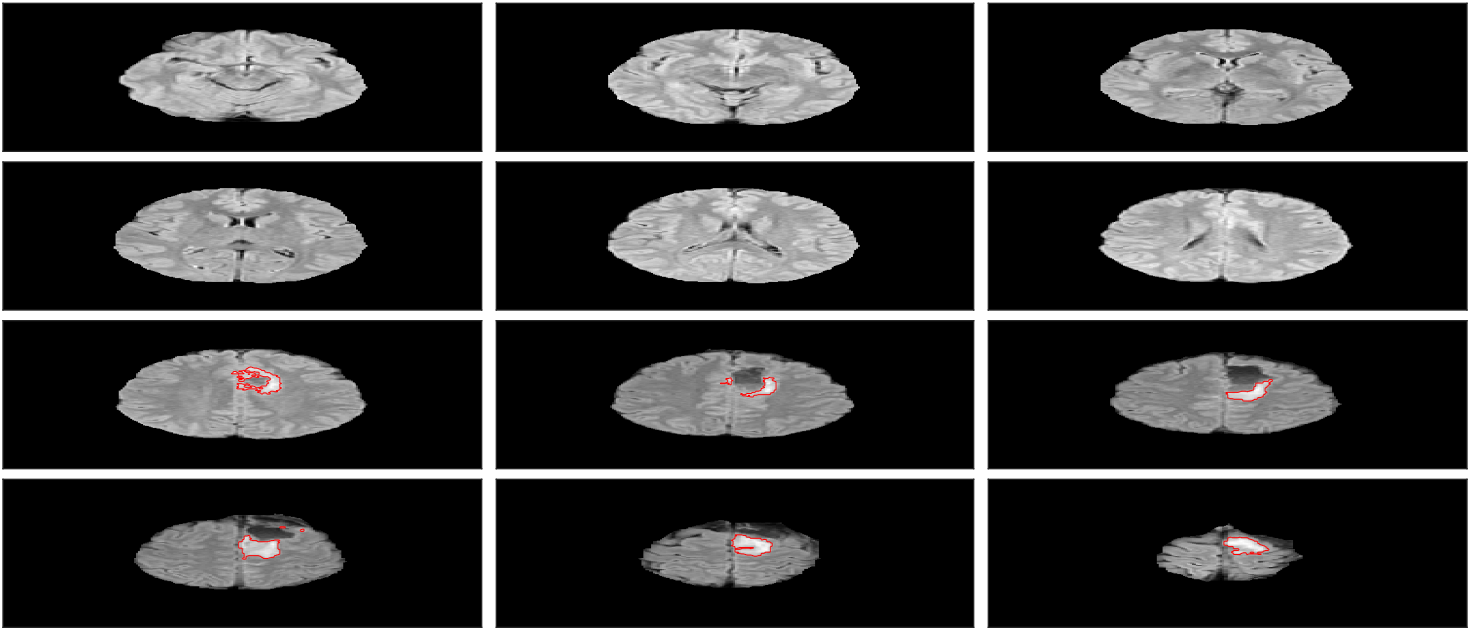

Supplement: S1 Data — The .zip file includes .pdf files labeled as follows, using case number 4224 as an example: 4224_t0.pdf includes the MRI data with segmentation of case number 4224 at baseline. 4224_t1_CAD_G_VC_S.pdf includes the MRI data with segmentation of case number 4224 at time 1, i.e., the time point of growth detection by the change-of-point method when the time point was different from the most recent MRI. 4224_tend_CAD_G_VC_G.pdf includes the MRI data with segmentation of case number 4224 at the most recent MRI. CAD_G and CAD_S mean that the change-of-point method predicted growth (G) or stability (S), respectively. VC_G and VC_S mean that the visual comparison detected growth (G) or stability (S), respectively. (ZIP) [file pmed.1002810.s001.zip › 7730_t0.pdf]

Case Number:7718; Time 0 (Baseline).

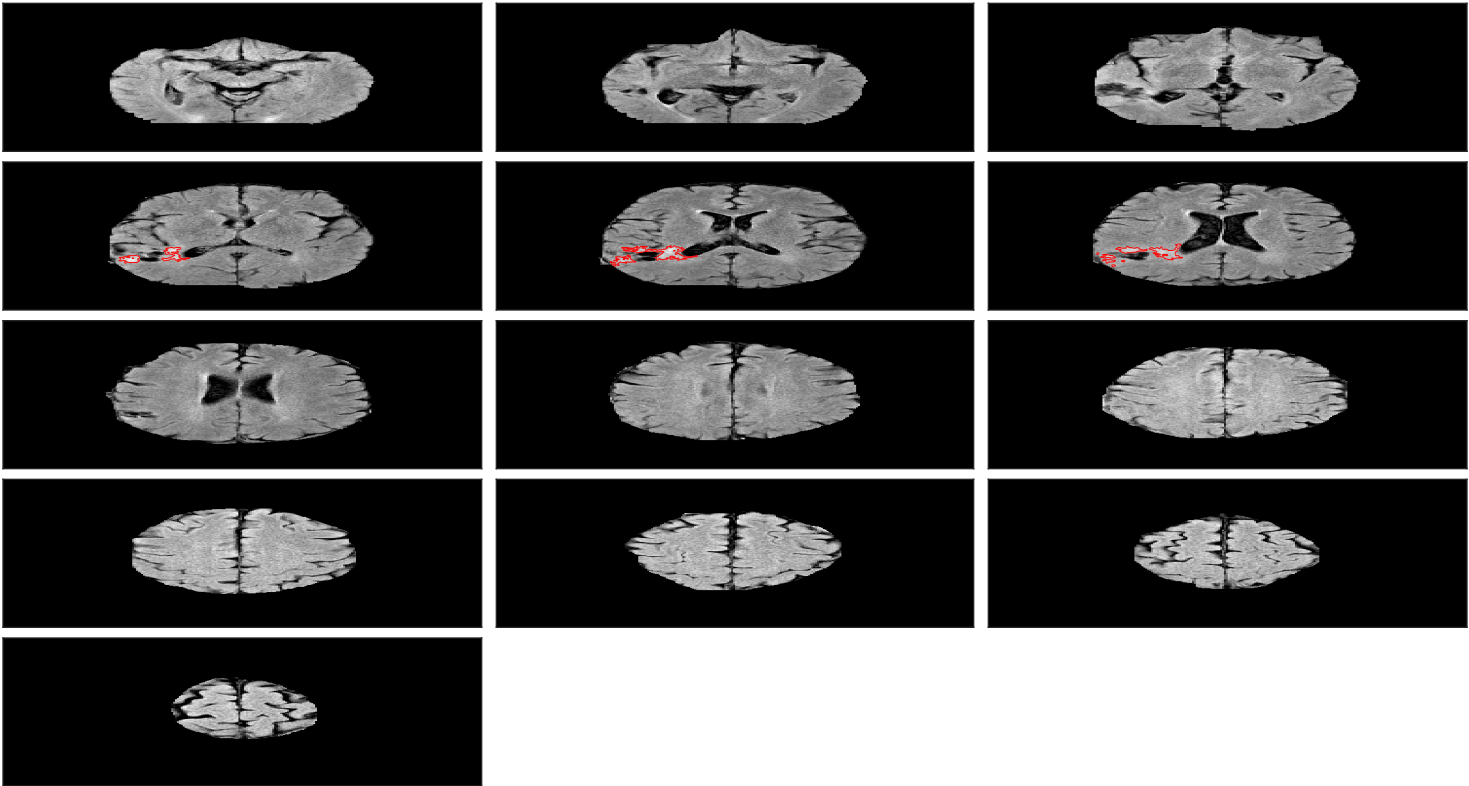

Supplement: S1 Data — The .zip file includes .pdf files labeled as follows, using case number 4224 as an example: 4224_t0.pdf includes the MRI data with segmentation of case number 4224 at baseline. 4224_t1_CAD_G_VC_S.pdf includes the MRI data with segmentation of case number 4224 at time 1, i.e., the time point of growth detection by the change-of-point method when the time point was different from the most recent MRI. 4224_tend_CAD_G_VC_G.pdf includes the MRI data with segmentation of case number 4224 at the most recent MRI. CAD_G and CAD_S mean that the change-of-point method predicted growth (G) or stability (S), respectively. VC_G and VC_S mean that the visual comparison detected growth (G) or stability (S), respectively. (ZIP) [file pmed.1002810.s001.zip › 7718_t0.pdf]

Case Number:7333; TIME 1; CAD --> GROWTH; VC --> STABLE; Interval From Baseline =19 months.

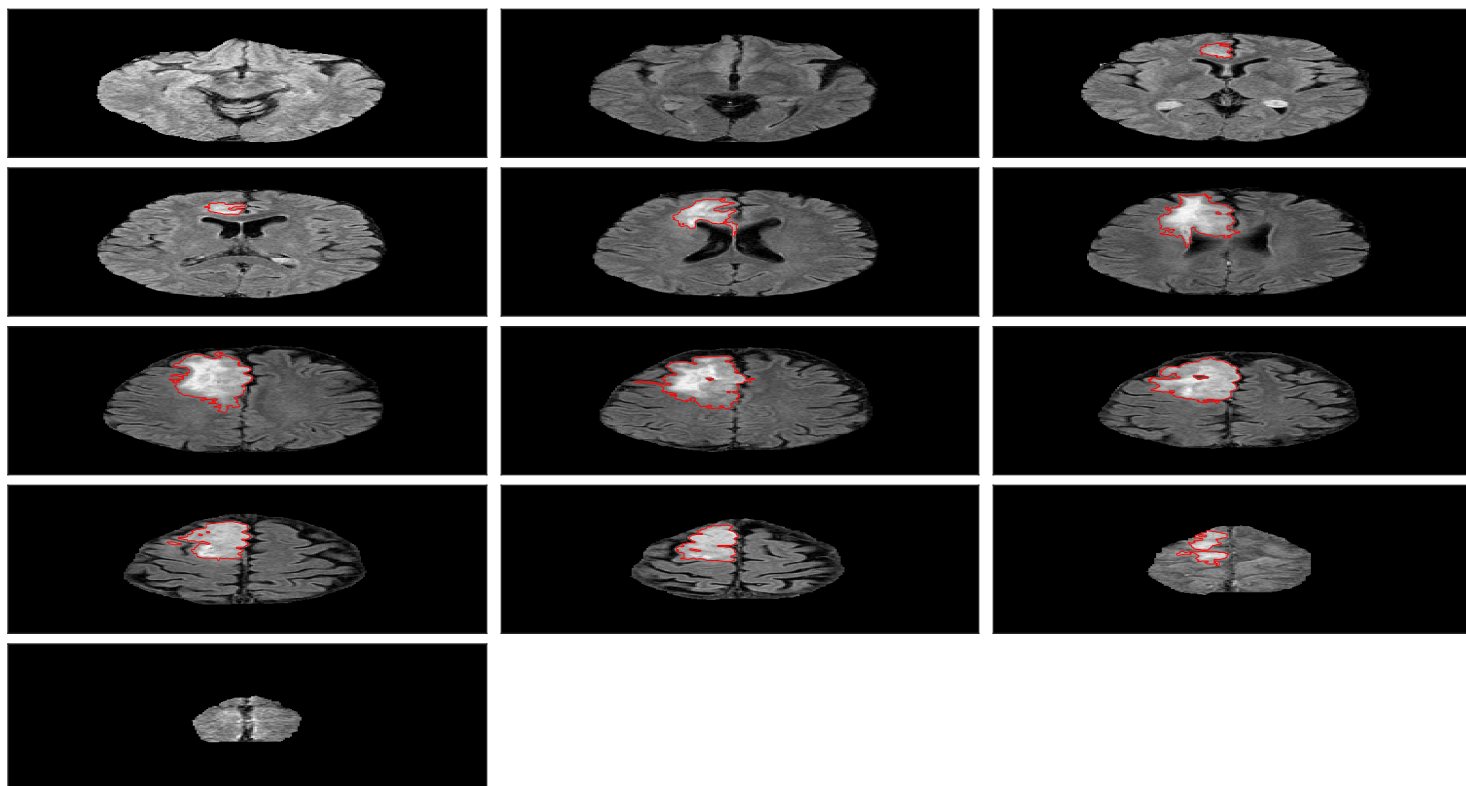

Supplement: S1 Data — The .zip file includes .pdf files labeled as follows, using case number 4224 as an example: 4224_t0.pdf includes the MRI data with segmentation of case number 4224 at baseline. 4224_t1_CAD_G_VC_S.pdf includes the MRI data with segmentation of case number 4224 at time 1, i.e., the time point of growth detection by the change-of-point method when the time point was different from the most recent MRI. 4224_tend_CAD_G_VC_G.pdf includes the MRI data with segmentation of case number 4224 at the most recent MRI. CAD_G and CAD_S mean that the change-of-point method predicted growth (G) or stability (S), respectively. VC_G and VC_S mean that the visual comparison detected growth (G) or stability (S), respectively. (ZIP) [file pmed.1002810.s001.zip › 7333_t1_CAD_G_VC_S.pdf]

Case Number:7471; LAST MRI; CAD --> GROWTH; VC --> STABLE; Interval From Baseline =101 months.

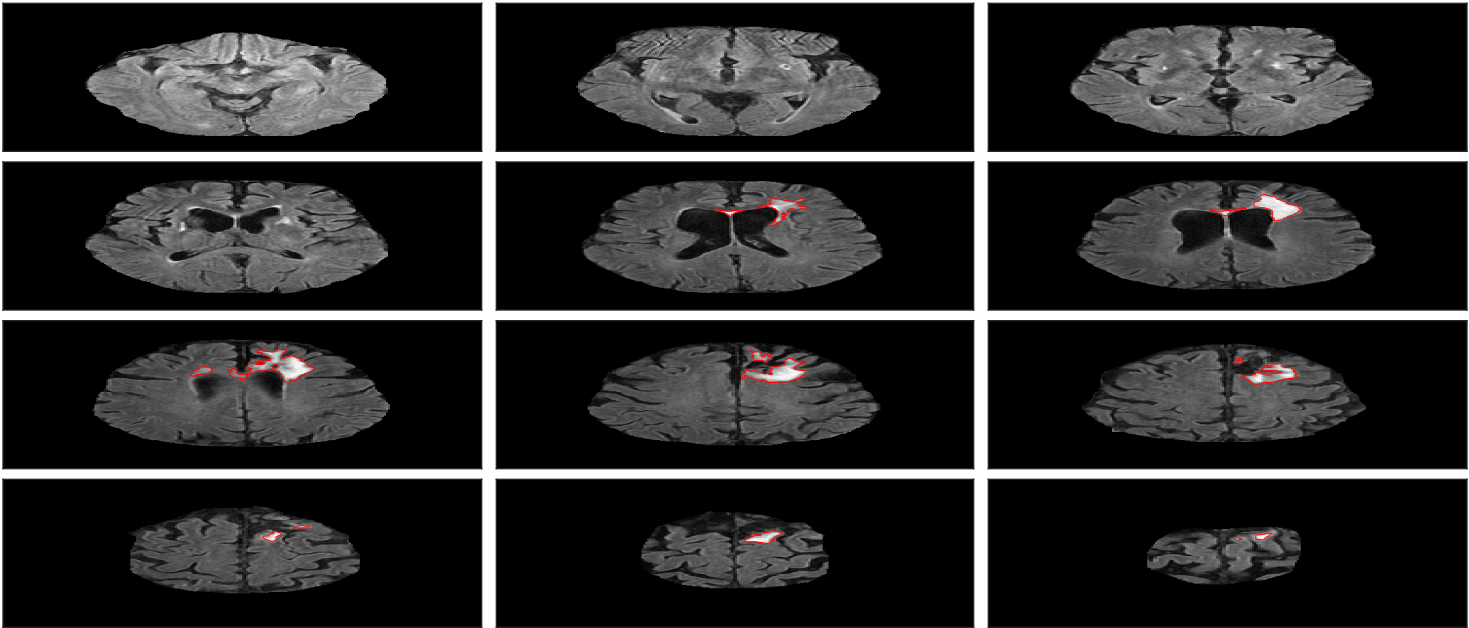

Supplement: S1 Data — The .zip file includes .pdf files labeled as follows, using case number 4224 as an example: 4224_t0.pdf includes the MRI data with segmentation of case number 4224 at baseline. 4224_t1_CAD_G_VC_S.pdf includes the MRI data with segmentation of case number 4224 at time 1, i.e., the time point of growth detection by the change-of-point method when the time point was different from the most recent MRI. 4224_tend_CAD_G_VC_G.pdf includes the MRI data with segmentation of case number 4224 at the most recent MRI. CAD_G and CAD_S mean that the change-of-point method predicted growth (G) or stability (S), respectively. VC_G and VC_S mean that the visual comparison detected growth (G) or stability (S), respectively. (ZIP) [file pmed.1002810.s001.zip › 7471_tend_CAD_G_VC_S.pdf]

Case Number:7714; Time 0 (Baseline).

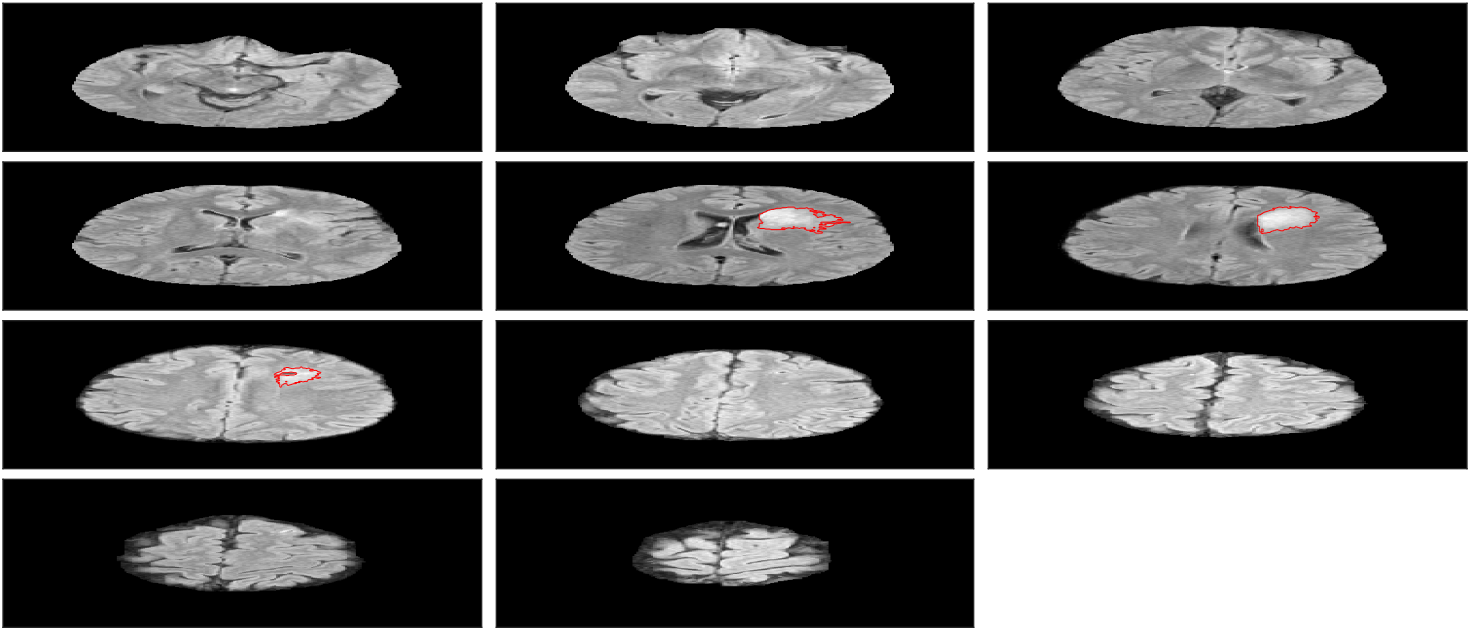

Supplement: S1 Data — The .zip file includes .pdf files labeled as follows, using case number 4224 as an example: 4224_t0.pdf includes the MRI data with segmentation of case number 4224 at baseline. 4224_t1_CAD_G_VC_S.pdf includes the MRI data with segmentation of case number 4224 at time 1, i.e., the time point of growth detection by the change-of-point method when the time point was different from the most recent MRI. 4224_tend_CAD_G_VC_G.pdf includes the MRI data with segmentation of case number 4224 at the most recent MRI. CAD_G and CAD_S mean that the change-of-point method predicted growth (G) or stability (S), respectively. VC_G and VC_S mean that the visual comparison detected growth (G) or stability (S), respectively. (ZIP) [file pmed.1002810.s001.zip › 7714_t0.pdf]
